# Gold-Catalyzed 1,3-Thiazine Formation and Uncommon Tautomer Isolation

**DOI:** 10.1021/acs.joc.2c00947

**Published:** 2022-08-09

**Authors:** Guillermo Canudo-Barreras, Daniel Salvador, Raquel P. Herrera, M. Concepción Gimeno

**Affiliations:** † Laboratorio de Organocatálisis Asimétrica, Departamento de Química Orgánica, Instituto de Síntesis Química y Catálisis Homogénea (ISQCH) CSIC-Universidad de Zaragoza, C/Pedro Cerbuna 12, 50009 Zaragoza, Spain; ‡ Departamento de Química Inorgánica, Instituto de Síntesis Química y Catálisis Homogénea (ISQCH) CSIC-Universidad de Zaragoza, C/Pedro Cerbuna 12, 50009 Zaragoza, Spain

## Abstract

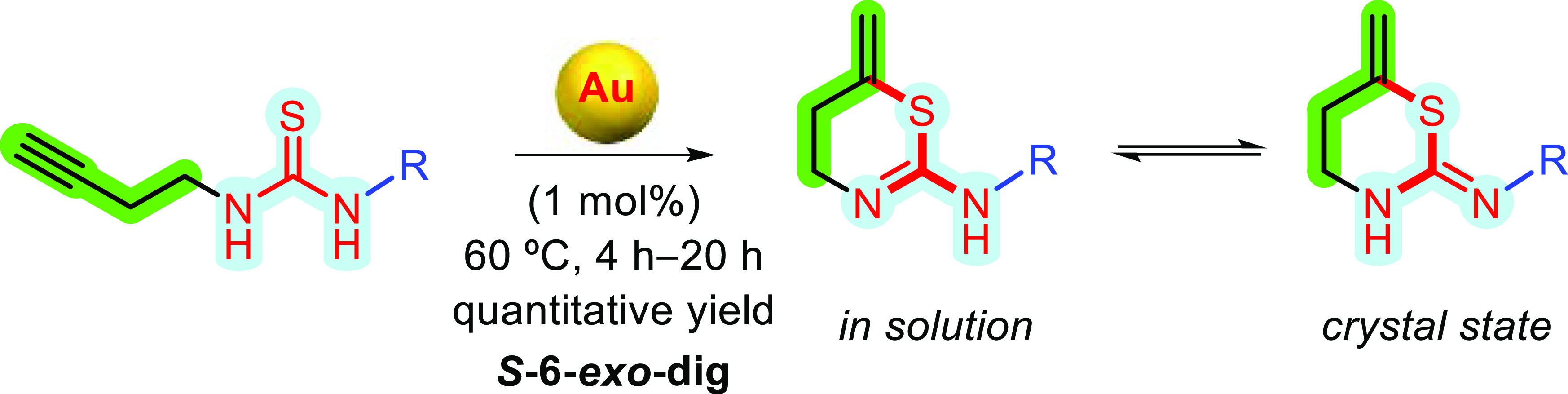

This work represents the first example of a gold-catalyzed formation of 1,3-thiazine/1,3-thiazinane by means of a catalytic approach and further uncommon isolation of the two tautomers. The developed protocol gives rise to a broad scope of 1,3-thiazine derivatives with excellent yields in short reaction times. Interestingly, different isomers could be obtained depending on the state of the compound, and in the crystal state the 1,3-thiazinane isomer is obtained, while in solution the 1,3-thiazine is the unique isomer. This work represents an interesting approach for the synthesis of potential biologically relevant molecules and a crucial precedent in tautomerism isolation and characterization.

## Introduction

The development of new protocols for the efficient synthesis of heterocyclic compounds^1^ has encouraged the efforts of chemists as a continuous challenging aim in the discovery of new strategies for diversity-oriented synthesis.^2^ Thiazines are considered as a privileged structural core among the plethora of heterocyclic scaffolds, existing in three different isomers depending on the position of the nitrogen and the sulfur atom in the six-membered ring. These species are important due to their biological properties such as antifungal, anticonvulsant, antitubercular, antibacterial, antimicrobial, antitumor, insecticidal, fungicidal, herbicidal agents, tranquilizers, and various antiviral.^3^ Among these isomers, those focused on benzo-1,3-thiazines have received major attention because they are the structural core of many pharmaceutically active molecules.^4^ In contrast, 1,3-thiazines or 1,3-thiazinanes have been less explored.

**Scheme 1:**
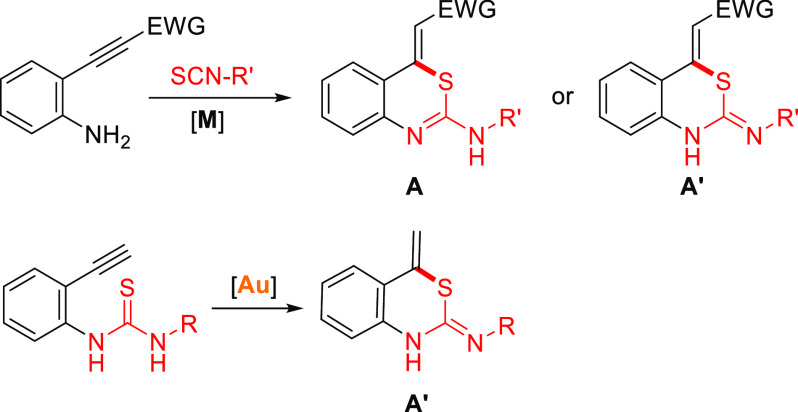
Synthesis of Benzo-1,3-thiazines

Some methodologies have succeeded in synthesizing benzo-1,3-thiazines by a tandem cyclization mainly using super-stoichiometric amounts of the promoter,^5^ and there are only scarce examples involving a metal catalyst in a tandem 6-*exo*-dig cyclization.^6^ The addition reaction of internal alkynylaniline derivatives substituted, with electron-withdrawing groups, to aryl isothiocyanates affords the benzothiazine derivatives usually as a mixture of the two isomers A and A′.^5,6^ Recently, we have reported on the synthesis of benzothiazines mediated by gold catalysis starting from thioureas containing terminal alkynyl groups (Scheme 1). Interestingly, in our developed method, only tautomer A′ was achieved.^7^

The importance of this family of compounds justifies the continuous search for developing novel synthetic approaches starting from simple and available substrates, as the known methodologies are limited in structural diversification. In addition, the enhancement of catalysis makes this work a pioneering approach to obtain these uncommon species.

**Scheme 2:**
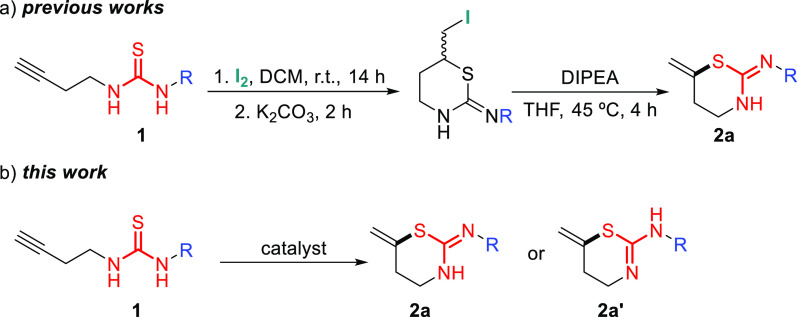
Use of Stoichiometric Amounts of I_2_ in the Synthesis of 1,3-Thiazinanes and Our Hypothesis of Work

It should be noted that none of the previous catalytic works have succeeded to obtain 1,3-thiazines or 1,3-thiazinane monocycles starting from alkynylamines, instead of using alkynylanilines, and the only example reported in the literature uses stoichiometric amounts of I_2_ following two reaction steps (Scheme 2a).^8^ Hence, based on our continue search for the discovery of new metal-catalyzed reactions, we have focused our investigation on the study of these interesting compounds by means of gold catalysts,^9^ and we have optimized the model process disclosed in Scheme 2b starting from butynyl thiourea derivatives.

## Results and Discussion

**Figure 1:**
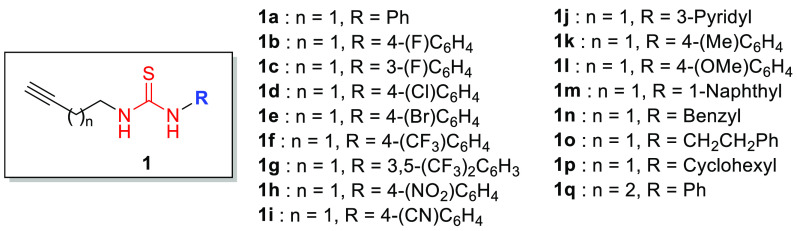
Synthesized thioureas **1a-q**.

To test this idea, a battery of thioureas **1** was first synthesized with excellent yields (Figure 1 and see the Supporting Information).

**Figure 2:**
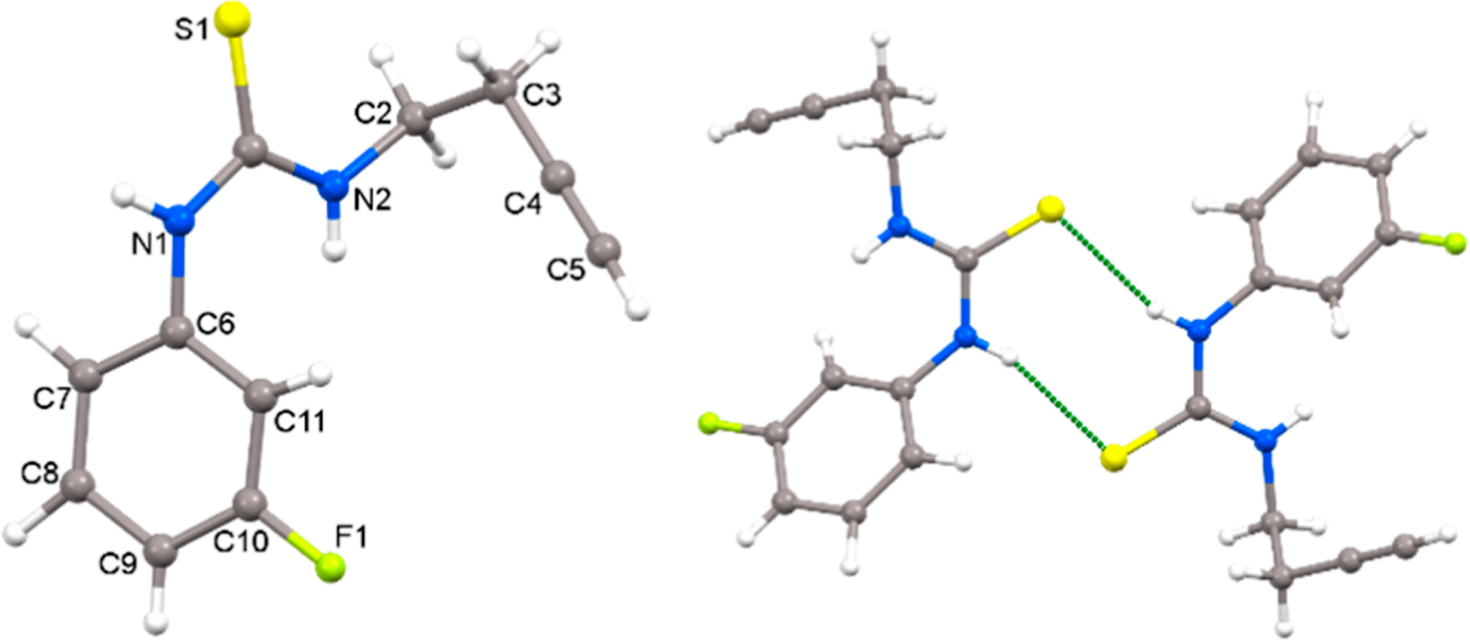
Crystal structure of thiourea **1c** and formation of dimers through hydrogen bonding.

The thioureas **1** have been characterized by NMR, and the structure of **1c** was confirmed by X-ray diffraction studies (Figure 2). The S1–C1 distance is 1.6917(18) Å, while the C–N distances are N1–C1 1.347(2) and N2–C1 1.339(2) Å, which are those expected for thiourea compounds. The presence of hydrogen bonds between the sulfur atom and the NH groups of adjacent molecules is also observed.^10^

**Figure 3:**
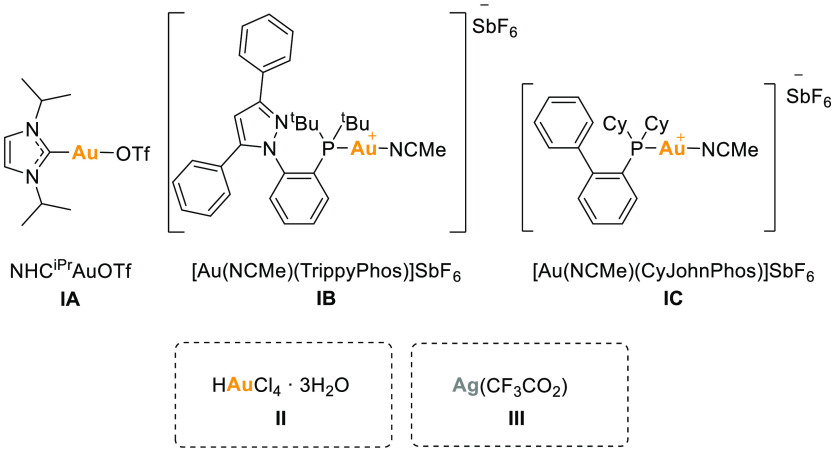
Catalysts tested in the model reaction.

In order to study the catalytic cyclization of these thioureas, some metal catalysts (Figure 3) and conditions were tested in the model reaction disclosed in Table 1. Several phosphine and N-heterocyclic carbene gold compounds, together with the most common salts such as chloroauric acid or silver trifluoroacetate, were chosen.

**Table 1:**
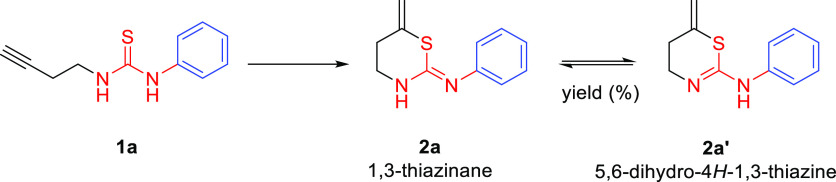
Screening of the Reaction Conditions to Obtain 1,3-Thiazinane **2a** or 1,3-Thiazine **2a′**^a^

entry	cat. (%)	solvent (mL)	temp. (°C)	time (h)^b^	yield (%)^c^
1	**IA** (5)	MeCN (0.5)	r.t.	47	n.d.
2	**IA** (5)	MeCN (0.5)	60	172	55
3	**IB** (3)	MeCN (0.5)	60	24	98
4	**IB** (3)	CH_2_Cl_2_ (1)	60	4	92
5	**IB** (1)	MeCN (0.5)	60	5	91
6	**IC** (1)	MeCN (0.5)	60	5	99
7	**II** (5)	MeCN (0.5)	r.t.	47	n.d.
8	**II** (5)	MeCN (0.5)	60	22	48
9	**II** (3)	MeCN (0.5)	60	71	n.d.
10	**II** (1)	MeCN (0.5)	60	71	n.d.
11	**II** (5)	Toluene (0.5)	r.t.	22	n.d.
12	**II** (5)	Toluene (0.5)	60	26	n.d.
13	**II** (5)	THF (0.5)	r.t.	22	12
14	**III** (10)	MeCN (0.5)	60	45	82

a Reaction: to a solution of the catalyst (amount indicated) in the solvent indicated (0.5 mL), the corresponding thiourea **1a** (0.1 mmol) was added. The reaction mixture was left stirring at different temperatures and the course of the reaction is followed by TLC (*n*-hexane/ethyl acetate 5:5). The catalyst was removed from the reaction mixture by silica gel, and the product was evaporated under vacuum. 1,3-Thiazinane **2a** was obtained as a white solid.

b Time until the TLC monitoring indicates either the full conversion of thiourea **1a** or no further progression of the reaction course.

c Isolated yield by column chromatography.

From all the combinations and parameters evaluated (catalysts, temperature, solvent, and catalytic concentration), it can be deduced that the best conditions are achieved with catalysts **IB** and **IC**, obtaining the highest yields (up to 99% for catalyst **IC**) after 5 h of reaction and using a catalyst loading of 1 mol % (in 0.5 mL of MeCN and at 60 °C) (entries 5 and 6, Table 1). Although catalyst **IB** offers similar yields and reaction times for the same catalyst loading and temperature than **IC**, the latter is chosen due to the lower synthetic complexity and availability of the phosphine. Additionally, we have also performed a proof testing the catalyst [Au(NCMe)(JohnPhos)]SbF_6_, bearing the analogous JohnPhos phosphine, under the best reaction conditions and we were able to get the same excellent results in the model reaction (5 h, 99%). In contrast, the Au(III) catalyst **II** (entry 8, Table 1) and the Ag(I) catalyst **III** (entry 14, Table 1), despite being capable of catalyzing the cyclization of thiourea, required higher catalytic loads—5 and 10 mol %, respectively—and longer reaction times—22 and 45 h—without achieving complete conversions. The screening of solvent afforded MeCN as the best choice, maybe due to the presence of a molecule of MeCN in the catalyst, and therefore, the necessity of an interchange between this solvent and the ligand in the catalytic cycle.

**Figure 4:**
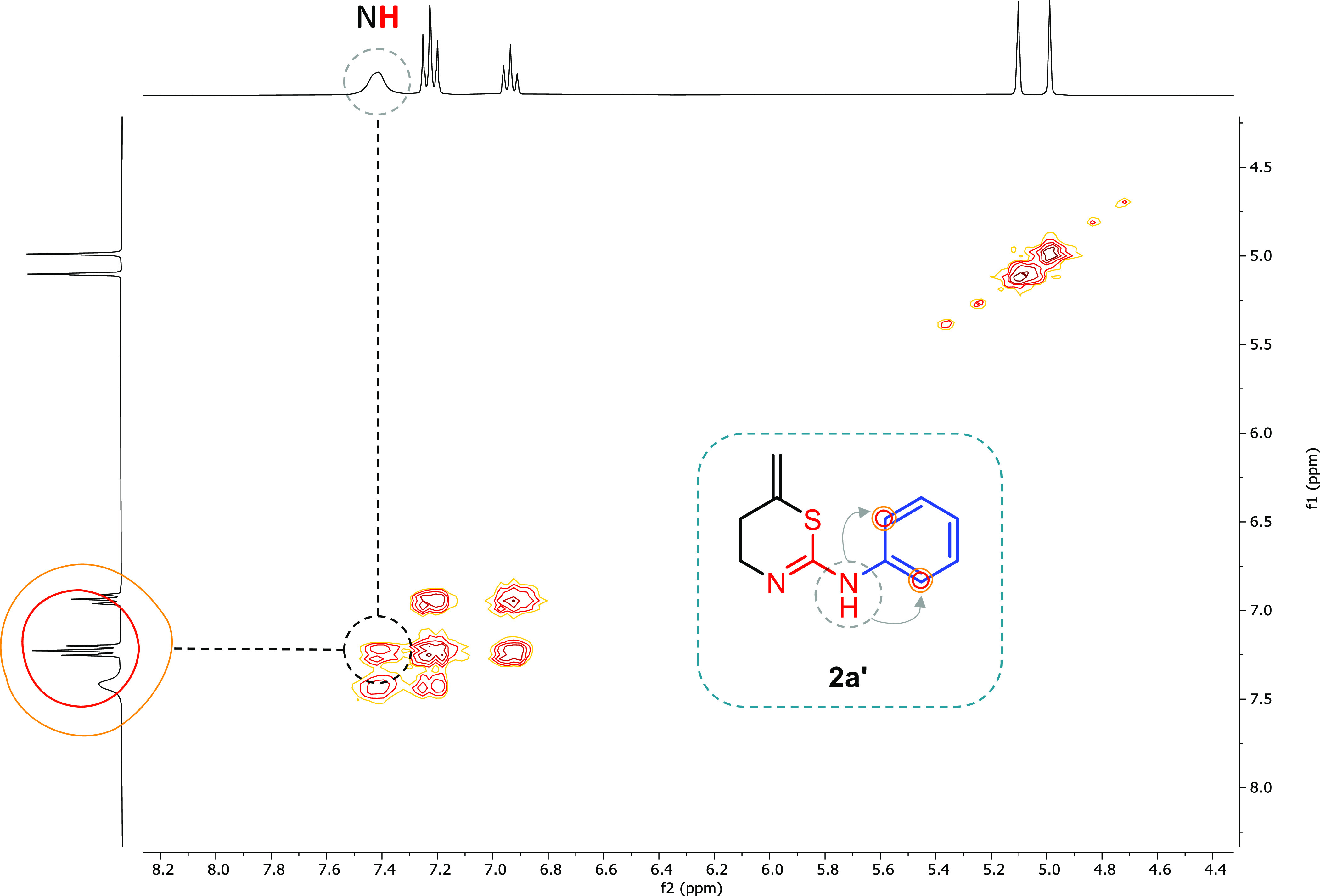
COSY NMR (300 MHz, CD_3_COCD_3_) spectrum for compound **2a′**.

**Figure 5:**
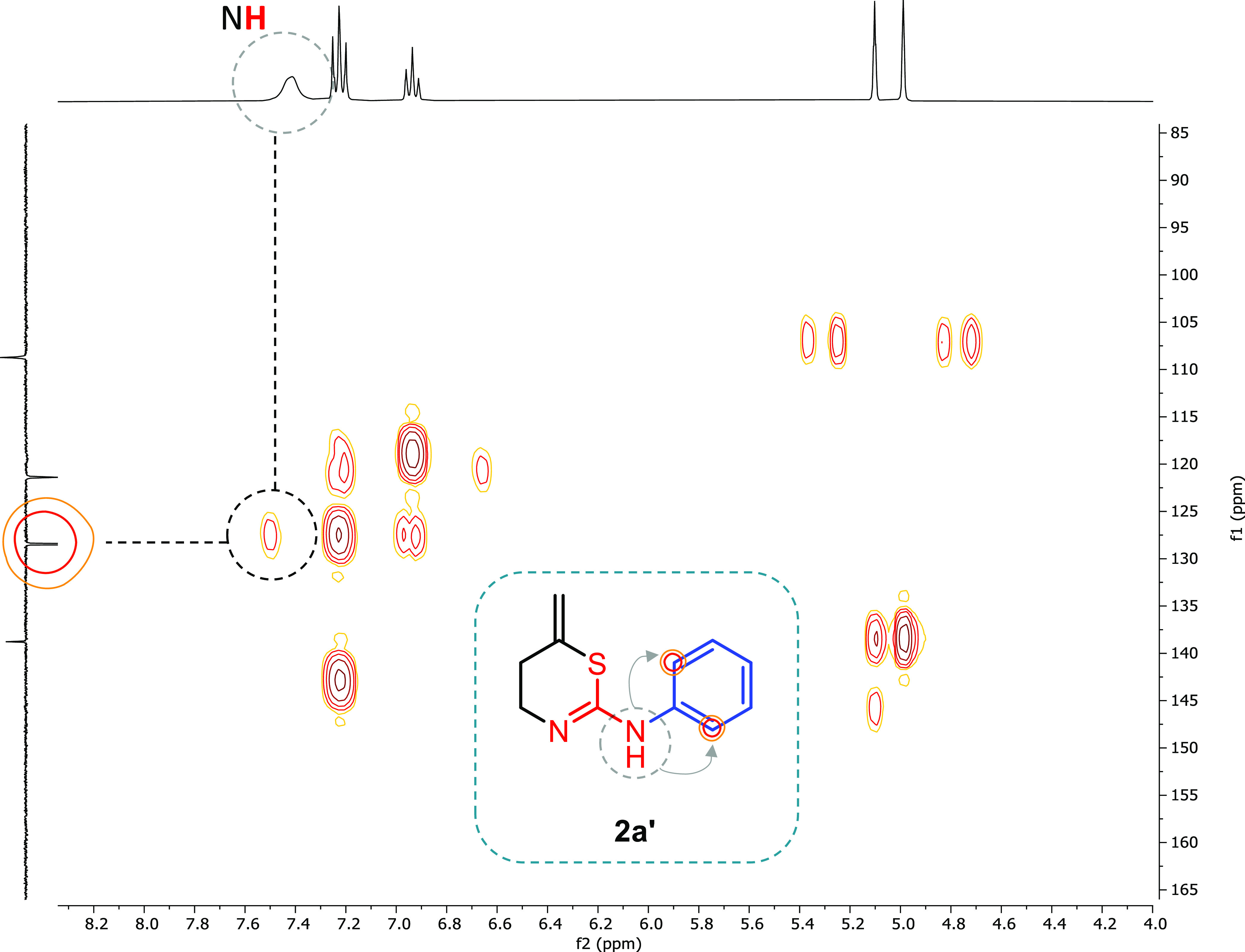
HMBC NMR (300 MHz, 75 MHz, CD_3_COCD_3_) spectrum for compound **2a′**.

Since an equilibrium exists between the tautomers **2a** (1,3-thiazinane) and **2a′** (5,6-dihydro-4*H*-1,3-thiazine), the first thing was to determine the species obtained in this procedure since surprisingly this important aspect has been overlooked in other published works. Because of the lack of literature about these compounds, multiple NMR experiments were carried out to elucidate the structure. The most indicative experiments were the COSY (Figure 4) and HMBC spectra (Figure 5).

If the 1,3-thiazinane **2a** were formed in solution, coupling between the CH_2_ and the **NH** within the ring would be expected. However, the coupling was only observed between the **NH** outside and aromatic protons for some of the final 1,3-thiazines **2′** (Figure 4) (see additional COSY spectra in the Supporting Information for more examples), but not with the CH_2_ of the ring. Therefore, it was assumed that **NH** is outside the cycle (as in **2a′** where at high concentration and long acquisition times for these experiments, we were able to find this key interaction). Moreover, in the HMBC spectra, coupling between the **NH** and aromatic C is also observed supporting the tautomer obtained (Figure 5). Therefore, in solution we can unambiguously conclude that we obtain **2a′** (5,6-dihydro-4*H*-1,3-thiazine) as the only product.

**Figure 6:**
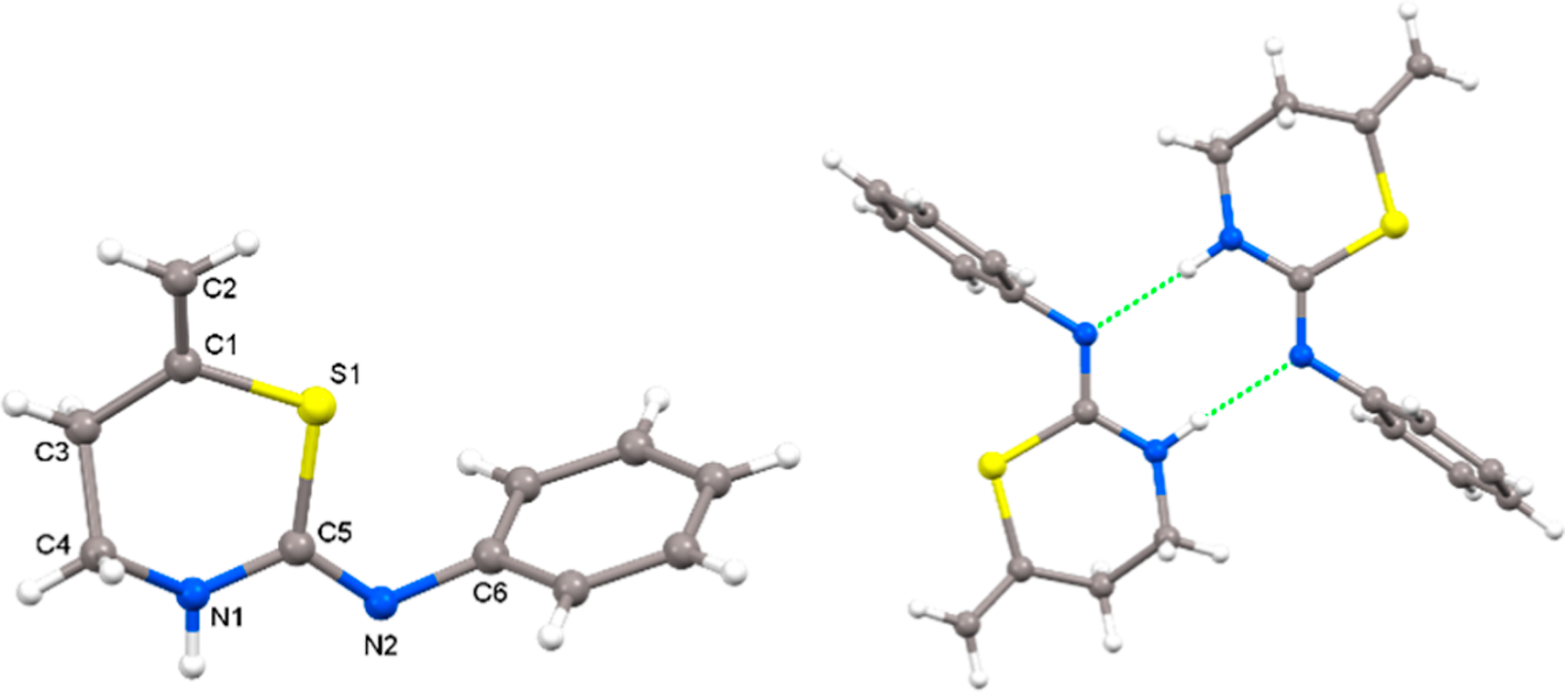
Crystal structure of 1,3-thiazinane **2a** and association through hydrogen bonding.

Interestingly, although the NMR data point toward the commented tautomer, a single crystal was grown from compound **2a**, and the structure was elucidated by X-ray diffraction studies (Figure 6).

Surprisingly, the crystal structure obtained highlights the presence of the **NH** group within the ring and the **N=C** outside the ring; in sharp contrast to that apparently observed in solution. Compound **2a** crystallizes with two independent molecules. Inside the ring, the N1–C distances are 1.451(4) and 1.348(3) Å, which although are dissimilar highlight the presence of the thiazinane moiety. The C5–N2 bond length is 1.285(3) Å, indicating the presence of the imine bond. The molecules associate in dimers through N–H···N hydrogen bonds of 2.012 Å.

This interesting phenomenon could be explained if the energetic difference between the crystalline packing of the solid state and the solvated state could be enough to stabilize one tautomer against the other in solution, giving rise to a different result when crystallizing in a certain solvent.^12^ It is worth mentioning that both tautomers have been isolated against the normal situation where even if the individual tautomers are isolated in the crystal state, in solution, they always exist as a mixture. Therefore, it is proposed that a tautomeric equilibrium can be modulated by inducing a phase change in the system, tuning some conditions of the medium. On the other hand, it could be appreciated that the equilibrium in solution between both tautomers is slow enough to analyze one of them. In this case, it seems that the crystal packing is favored for one of the tautomers, while the other is predominant in solution. The possibility to have two different building blocks in two distinct aggregation states or phases starting from the same single compound, may lead to the opportunity to work with each one separately to achieve a divergent synthetic step.

**Figure 7:**
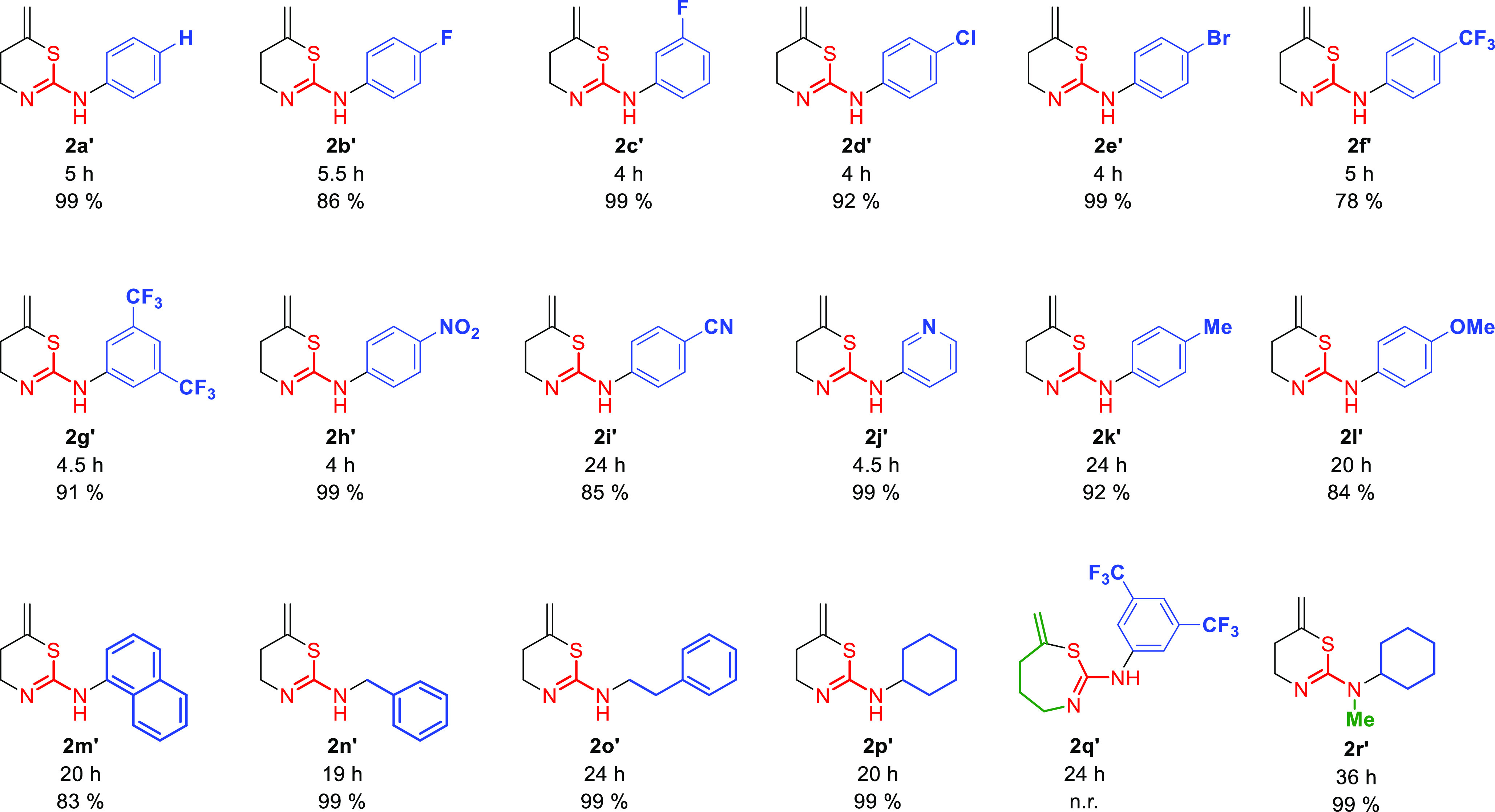
Synthesis of tautomers **2a′**–**p′** characterized in solution. N.r.: no reaction observed.

With the best reaction conditions in hand, we explored the scope of this approach for thioureas **1a**–**q**. In Figure 7, we represent the tautomers **2′** obtained and characterized for each product as found in solution.

**Scheme 3:**
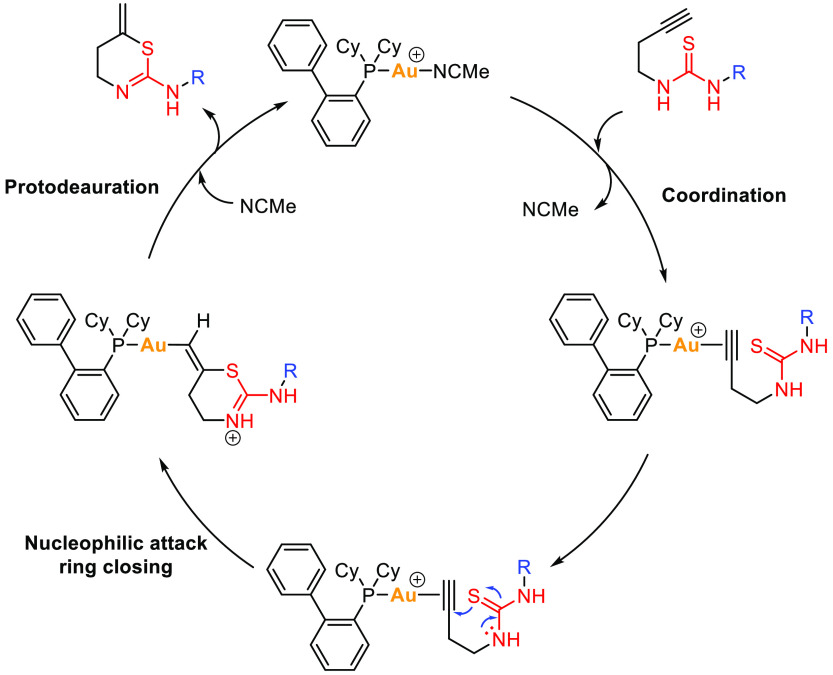
Gold-Catalyzed Formation of 5,6-Dihydro-4*H*-1,3-thiazines 2′

In all cases, excellent yields were obtained after short reaction times. Only, thioureas **1k**–**p** required longer reaction times than those with electron-withdrawing groups in the aromatic ring. However, the final products **2k′**–**p′** were also obtained with excellent yield. Thiourea **1i**, with a nitrile group, also required longer reaction times in contrast to the other activated substrates, but the final thiazine **2i′** was obtained with very good yields. As a limitation of this protocol, the reaction was set up to obtain **2q′**, a seven-membered ring. However, the reaction was unsuccessful under the same conditions. Interestingly, we tried to methylate **2p′** with MeI, and the final *N*-methyl-1,3-thiazine **2r′** was obtained with quantitative yield in a very clean reaction. Additionally, to prove the utility of this catalytic procedure, a scaled-up example has been performed. In this case, the procedure was conducted with 1 mmol of **1a** giving **2a′** in 83% (169.6 mg), although with longer reaction times (24 h). On the basis of the experimental results and in the chemistry of gold, we propose a plausible mechanism explaining the final products obtained (Scheme 3).

After coordination of thiourea **1** to the gold center of the catalyst, an intramolecular nucleophilic attack of the sulfur atom over the triple bond would give rise to the cyclization of the product. Final protodeauration would produce the final thiazine derivative and release of the catalyst to initiate the catalytic cycle again.

## Conclusions

In summary, we have shown the first example of a gold-catalyzed formation of 1,3-thiazine/1,3-thiazinane derivatives starting from a family of thiourea derivatives containing the butynyl moiety. The developed protocol gives rise to a broad scope of 1,3-thiazine derivatives with excellent yields in short reaction times and with low catalyst loading. The scope of this methodology may allow a great structural diversification, on one side using functionalized butynyl amines and on the other side modulated by the substituents of the isothiocyanate compounds. Although at this point the pentynyl amine derivate has not worked under the best reaction conditions, this opens the possibility of further studies to obtain new seven-membered ring scaffolds. Interestingly, the two different tautomers were identified depending on the state of the compound, and in the crystal state, the 1,3-thiazinane isomer was obtained, while in solution, the 1,3-thiazine was the unique isomer. This work represents an interesting procedure for the synthesis of potential biologically relevant molecules and an important precedent in tautomerism isolation and characterization.

## Supporting Information Available

General experimental methods and instrumentation, general procedure for the synthesis of thioureas **1a**–**q**, general procedure for the synthesis of 1,3-thiazines **2a′**–**p′** and **2r′**, synthesis of catalyst [Au(NCMe)(CyJohnPhos)]SbF_6_ (**IC**), and ^1^H, ^13^C{^1^H}-APT, COSY, HSQC, and HMBC NMR spectra (PDF)
