# Supplementary material for: Gold-Catalyzed 1,3-Thiazine Formation and Uncommon Tautomer Isolation
Source: J Org Chem. 2022 Aug 9;87(16):10747–54. doi: 10.1021/acs.joc.2c00947 (PMC9400392; doi:10.1021/acs.joc.2c00947)

Gold catalyzed 1,3-thiazine formation and uncommon tautomer isolation

Guillermo Canudo-Barreras,[‡ξ] Daniel Salvador,[ξ] Raquel P. Herrera[‡]* and M. Concepción Gimeno[ξ]*

[‡] Laboratorio de Organocatálisis Asimétrica, Departamento de Química Orgánica, Instituto de Síntesis Química y Catálisis Homogénea (ISQCH) CSIC-Universidad de Zaragoza, C/ Pedro Cerbuna 12, 50009 Zaragoza, Spain. Email: raquelph@unizar.es

[ξ] Departamento de Química Inorgánica, Instituto de Síntesis Química y Catálisis Homogénea (ISQCH) CSIC-Universidad de Zaragoza, C/ Pedro Cerbuna 12, 50009 Zaragoza, Spain. Email: gimeno@unizar.es

Supporting Information

1. General experimental methods and instrumentation S2

2. General procedure for the synthesis of thioureas 1a-q S2

3. General procedure for the synthesis of 1,3-thiazines 2a-r’...S8

4. Synthesis of catalyst [Au(NCMe)(CyJohnPhos)]SbF$_6$ (IC) S14

5. ^{1}H, ^{13}C{^{1}H}-APT, COSY, HSQC and HMBC NMR spectra S15

5.1. Spectra of thioureas 1a-q S15

5.2. Spectra of 5,6-dihydro-4*H*-1,3-thiazines 2’ S38

5.3. Spectra of gold(I) catalyst IC S68

1. General experimental methods and instrumentation

Analytical thin-layer chromatography was performed on 0.25 mm silica gel 60-F plates. ESI and MicroTof-Q mass analyzer were used for HRMS measurements. NMR spectroscopy was conducted using a Bruker AVANCE–II spectrometer. ^{1}H NMR spectra were recorded at 300 and 400 MHz; ^{13}C{^{1}H}-APT NMR spectra were recorded at 75 and 101 MHz; DMSO, CD_3CN, CD_3COCD_3, CD_2Cl_2 and $CDCl_3$ were used as the deuterated solvents. Chemical shifts were reported in the δ scale relative to residual DMSO (2.50 ppm), CH_3CN (1.94 ppm), CH_3COCH_3 (2.05 ppm), CH_2Cl_2 (5.32 ppm) and $CHCl_3$ (7.26 ppm) for ^{1}H-NMR and the central line of DMSO-d_6 (39.52 ppm), CD_3CN (1.32 pp), CD_3COCD_3 (29.84 ppm), CD_2Cl_2 (53.84 ppm) and $CDCl_3$ (77.16 ppm) for ^{13}C{^{1}H}-APT NMR.

All commercially available solvents and reagents were used as received. Structural assignments were made with additional information from gCOSY, gHSQC, and gHMBC experiments.

2. General procedure for the synthesis of thioureas 1a-q

Based on the common procedures on the literature, with some modification in the proportions of the reagents, thioureas **1a-q** have been synthesized as followed: to a solution of but-3-yn-1-amine hydrochloride (**3**) (1 equiv., 0.5 mmol, 0.5 M) or pent-4-yn-1-amine hydrochloride (**4**) and of Et_3N (1 equiv., 0.5 mmol, 70 μL) in CH_2Cl_2 (1.0 mL), the corresponding isothiocyanate **5** (1.1 equiv., 0.55 mmol) is added. The reaction mixture is stirred at room temperature between 1 and 5 days and followed by TLC (*n*-hexane:ethyl acetate 7:3) until the but-3-yn-1-amine (**3**) disappears and a white —also yellow— solid precipitates. The only exception is the thiourea **1j**, which is obtained as an orange gel instead of a solid.

The product is partially evaporated, precipitated with hexane and the excess of isothiocyanate **5** is removed by filtration and washed with hexane. The characterizations are done by ^{1}H-NMR, ^{13}C{^{1}H}-APT, COSY, HSQC and HRMS. The conversions are always quantitative. Yields after purification and reaction times are shown in **Table S1**.

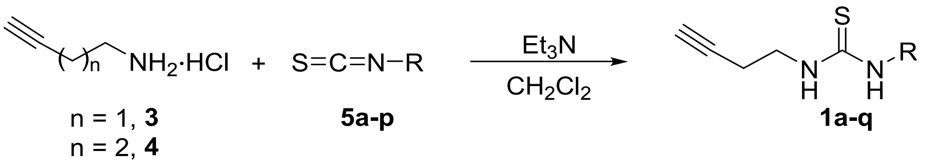

***Table S1**. Reaction conditions of the synthesis of thioureas **1a** to **1q**.*

Thiourea 1	Amine·HCl	R	time (days)	yield (%)	Conv. (%)[(a)]
**1a**	**3**	Ph	4	63	> 99
**1b**	**3**	4F-C_6H_4	2	92	> 99
**1c**	**3**	3F-C_6H_4	1	71	> 99
**1d**	**3**	4Cl-C_6H_4	4	89	> 99
**1e**	**3**	4Br-C_6H_4	4	95	> 99

**1f**	**3**	$4CF_3$-C_6H_4	1	98	> 99
**1g**	**3**	$3,5(CF_3)_2$-C_6H_3	4	65	> 99
**1h**	**3**	$4NO_2$-C_6H_4	2	98	> 99
**1i**	**3**	$4CN$-C_6H_4	1	85	> 99
**1j**	**3**	3-Py	2	52	> 99
**1k**	**3**	$4Me$-C_6H_4	5	90	> 99
**1l**	**3**	$4MeO$-C_6H_4	3	87	> 99
**1m**	**3**	1-Naphthyl	4	70	> 99
**1n**	**3**	Benzyl	1	97	> 99
**1o**	**3**	CH_2CH_2Ph	1	92	> 99
**1p**	**3**	Cy	1	82	> 99
**1q**	**4**	$3,5$-$(CF_3)_2$-C_6H_3	3	67	> 99

1-(But-3-yn-1-yl)-3-phenylthiourea (1a)

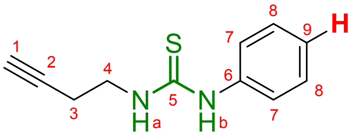

1a

Following the general procedure, thiourea **1a** was obtained after 4 days of reaction, at room temperature, as a yellow solid in 63 % yield (64.4 mg). ^{1}H NMR (400 MHz, $CDCl_3$): δ 7.88 (br s, 1H, NH_b), 7.50–7.38 (m, 2H, H-Ar), 7.37–7.21 (m, 3H, H-Ar), 6.41 (br s, 1H, NH_a), 3.80 (q, J = 5.9 Hz, 2H, H-C_4), 2.54 (td, J = 6.3, 2.6 Hz, 2H, H-C_3), 1.93 (t, J = 2.6 Hz, 1H, H-C_1). ^{13}C{^{1}H}-APT NMR (75 MHz, $CDCl_3$): δ 180.9 (s, 1C, C_5), 136.0 (s, 1C, C_6), 130.4 (s, 2C, C_8), 127.6 (s, 1C, C_9), 125.5 (s, 2C, C_7), 81.5 (s, 1C, C_2), 70.4 (s, 1C, C_1), 43.8 (s, 1C, C_4), 19.0 (s, 1C, C_3). HRMS (ESI+) calculated for $C_{11}H_{13}N_2S$ 205.0794; found 205.0794 $[M+H]^+$.

1-(But-3-yn-1-yl)-3-(4-fluorophenyl)thiourea (1b)

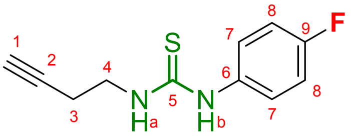

1b

Following the general procedure, thiourea **1b** was obtained after 2 days of reaction, at room temperature, as a yellow solid in 92 % yield (102.2 mg). ^{1}H NMR (400 MHz, $CDCl_3$): δ 7.97 (br s, 1H, NH_b), 7.30–7.20 (m, 2H, H-Ar), 7.18–7.06 (m, 2H, H-Ar), 6.25 (br s, 1H, NH_a), 3.78 (t, J = 6.2 Hz, 2H, H-C_4), 2.53 (td, J = 6.2, 2.6 Hz, 2H, H-C_3), 1.93 (t, J = 2.6 Hz, 1H, H-C_1). ^{13}C{^{1}H}-APT NMR (101 MHz, $CDCl_3$): δ 181.2 (s, 1C, C_5), 161.7 (d, J = 248.8 Hz, 1C, C_9), 131.8 (s, 1C, C_6), 128.1 (d, J = 8.7 Hz, 2C, C_7), 117.3 (d, J = 22.7 Hz, 2C, C_8), 81.5 (s, 1C, C_2), 70.5 (s, 1C, C_1), 43.6 (s, 1C, C_4), 19.0 (s, 1C, C_3). HRMS (ESI+) calculated for $C_{11}H_{12}FN_2S$ 223.0700; found 223.0701 $[M+H]^+$.

1-(But-3-yn-1-yl)-3-(3-fluorophenyl)thiourea (1c)

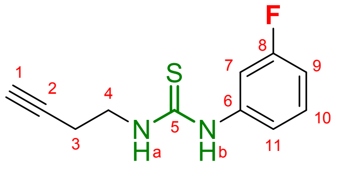

1c

Following the general procedure, thiourea **1c** was obtained after 1 day of reaction, at room temperature, as a yellow solid in 71 % yield (78.9 mg). ^{1}H NMR (300 MHz, $CDCl_3$): δ 8.15 (br s, 1H, NH_b), 7.46–7.32 (m, 1H, H-Ar), 7.07–6.94 (m, 3H, H-Ar), 6.54 (br s, 1H, NH_a), 3.81 (q, J = 6.1 Hz, 2H, H-C_4), 2.56 (td, J = 6.2, 2.6 Hz, 2H, H-C_3), 1.99 (t, J = 2.6 Hz, 1H, H-C_1). ^{13}C{^{1}H}-APT NMR (75 MHz, $CDCl_3$): δ 180.5 (s, 1C, C_5), 163.5 (d, J = 249.3 Hz, 1C, C_8), 137.7 (d, J = 9.6 Hz, 1C, C_6), 131.6 (d, J = 9.4 Hz, 1C, C_{10}), 120.4 (d, J = 3.0 Hz, 1C, C_{11}), 114.2 (d, J = 21.0 Hz, 1C, C_7), 112.2 (d, J = 23.5 Hz, 1C, C_9), 81.5 (s, 1C, C_2), 70.7 (s, 1C, C_1), 43.7 (s, 1C, C_4), 18.9 (s, 1C, C_3). HRMS (ESI+) calculated for $C_{11}H_{11}FN_2NaS$ 245.0519; found 245.0519 $[M+Na]^+$.

1-(But-3-yn-1-yl)-3-(4-chlorophenyl)thiourea (1d)

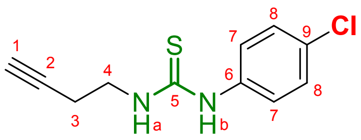

1d

Following the general procedure, thiourea **1d** was obtained after 4 days of reaction, at room temperature, as a yellow solid in 89 % yield (106.2 mg). ^{1}H NMR (300 MHz, $CDCl_3$): δ 8.02 (br s, 1H, NH_b), 7.45–7.34 (m, 2H, H-Ar), 7.26–7.14 (m, 2H, H-Ar), 6.34 (br s, 1H, NH_a), 3.79 (q, J = 6.0 Hz, 2H, H-C_4), 2.54 (td, J = 6.2, 2.6 Hz, 2H, H-C_3), 1.96 (t, J = 2.6 Hz, 1H, H-C_1). ^{13}C{^{1}H}-APT NMR (101 MHz, $CDCl_3$): δ 180.8 (s, 1C, C_5), 134.5 (s, 1C, C_6), 133.2 (s, 1C, C_9), 130.5 (s, 2C, C_7), 126.7 (s, 2C, C_8), 81.5 (s, 1C, C_2), 70.6 (s, 1C, C_1), 43.7 (s, 1C, C_4), 18.9 (s, 1C, C_3). HRMS (ESI+) calculated for $C_{11}H_{12}ClN_2S$ 239.0404; found 239.0409 $[M+H]^+$.

1-(But-3-yn-1-yl)-3-(4-bromophenyl)thiourea (1e)

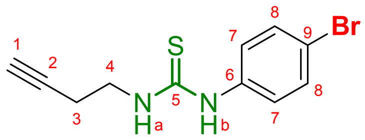

1e

Following the general procedure, thiourea **1e** was obtained after 4 days of reaction, at room temperature, as a yellow solid in 95 % yield (134.5 mg). ^{1}H NMR (400 MHz, CD_3COCD_3): δ 9.03 (br s, 1H, NH_b), 7.54–7.41 (m, 5H, H-Ar, NH_a), 3.76 (q, J = 6.6 Hz, 2H, H-C_4), 2.56 (td, J = 7.0, 2.7 Hz, 2H, H-C_3), 2.43 (t, J = 2.8 Hz, 1H, H-C_1). ^{13}C{^{1}H}-APT NMR (101 MHz, CD_3COCD_3): δ 182.4 (s, 1C, C_5), 139.2 (s, 1C, C_6), 132.8 (s, 2C, C_7), 126.6 (s, 2C, C_8), 118.2 (s, 1C, C_9), 82.6 (s, 1C, C_2), 71.2 (s, 1C, C_1), 44.1 (s, 1C, C_4), 19.1 (s, 1C, C_3). HRMS (ESI-) calculated for $C_{11}H_{10}BrN_2S$ 280.9743; found 280.9741 $[M-H]^-$.

1-(But-3-yn-1-yl)-3-(4-(trifluoromethyl)phenyl)thiourea (1f)

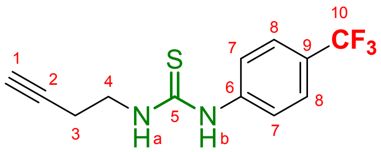

1f

Following the general procedure, thiourea **1f** was obtained after 1 day of reaction, at room temperature, as a yellow solid in 98 % yield (133.4 mg). ^{1}H NMR (300 MHz, $CDCl_3$): δ 8.72 (br s, 1H, NH_b), 7.66 (d, J = 8.0 Hz, 2H, H-Ar), 7.40 (d, J = 8.2 Hz, 2H, H-Ar), 6.58 (br s, 1H, NH_a), 3.81 (q, J = 6.1 Hz, 2H, H-C_4), 2.57 (td, J = 6.2, 2.6 Hz, 2H, H-C_3), 1.99 (t, J = 2.6 Hz, 1H,

H-C_1). $^{13}C\{^1H\}$-APT NMR (75 MHz, $CDCl_3$): δ 180.3 (s, 1C, C_5), 139.6 (s, 1C, C_6), 128.6 (q, J = 33.2 Hz, 1C, C_9), 127.4 (q, J = 3.6 Hz, 2C, C_8), 124.2 (s, 2C, C_7), 123.8 (q, J = 272.0 Hz, 1C, C_{10}), 81.5 (s, 1C, C_2), 70.7 (s, 1C, C_1), 43.7 (s, 1C, C_4), 18.8 (s, 1C, C_3). HRMS (ESI+) calculated for $C_{12}H_{12}F_3N_2S$ 273.0668; found 273.0662 $[M+H]^+$

1-(3,5-Bis(trifluoromethyl)phenyl)-3-(but-3-yn-1-yl)thiourea (1g)

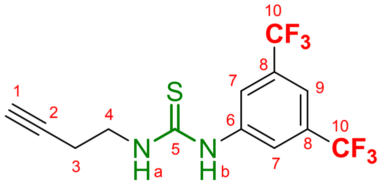

1g

Following the general procedure, thiourea **1g** was obtained after 4 days of reaction, at room temperature, as a yellow solid in 65 % yield (110.5 mg). ^1H NMR (300 MHz, $CDCl_3$): δ 8.22 (br s, 1H, NH_b), 7.79 (s, 2H, H-C_7), 7.76 (s, 1H, H-C_9), 6.47 (br s, 1H, NH_a), 3.83 (t, J = 6.1 Hz, 2H, H-C_4), 2.60 (td, J = 6.1, 2.6 Hz, 2H, H-C_3), 2.02 (t, J = 2.6 Hz, 1H, H-C_1). $^{13}C\{^1H\}$-APT NMR (75 MHz, $CDCl_3$): δ 180.6 (s, 1C, C_5), 138.3 (s, 1C, C_6), 133.7 (q, J = 34.0 Hz, 2C, C_8), 124.4 (s, 2C, C_7), 122.8 (q, J = 272.0 Hz, 2C, C_{10}), 120.2 (m, 1C, C_9), 81.2 (s, 1C, C_2), 71.1 (s, 1C, C_1), 43.8 (s, 1C, C_4), 18.8 (s, 1C, C_3). HRMS (ESI+) calculated for $C_{13}H_{11}F_6N_2S$ 341.0542; found 341.0542 $[M+H]^+$.

1-(But-3-yn-1-yl)-3-(4-nitrophenyl)thiourea (1h)

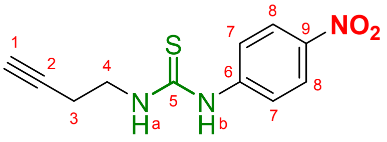

1h

Following the general procedure, thiourea **1h** was obtained after 2 day of reaction, at room temperature, as a yellow solid in 98 % yield (122.2 mg). ^1H NMR (300 MHz, CD_3COCD_3): δ 9.52 (br s, 1H NH_b), 8.24–8.16 (m, 2H, H-Ar), 7.97–7.88 (m, 2H, H-Ar), 7.84 (br s, 1H, NH_a), 3.80 (td, J = 6.9. 5.7 Hz, 2H, H-C_4), 2.60 (td, J = 6.9, 2.7 Hz, 2H, H-C_3), 2.47 (t, J = 2.7 Hz, 1H, H-C_1). $^{13}C\{^1H\}$-APT NMR (75 MHz, $CDCl_3$): δ 181.9 (s, 1C, C_5), 146.7 (s, 1C, C_6), 125.2 (s, 2C, C_8), 122.1 (s, 2C, C_7), 114.8 (s, 1C, C_9), 82.3 (s, 1C, C_2), 71.3 (s, 1C, C_1), 44.0 (s, 1C, C_4), 18.7 (s, 1C, C_3). HRMS (ESI+) calculated for $C_{11}H_{11}N_3NaO_2S$ 272.0464; found 272.0454 $[M+Na]^+$.

1-(But-3-yn-1-yl)-3-(4-cyanophenyl)thiourea (1i)

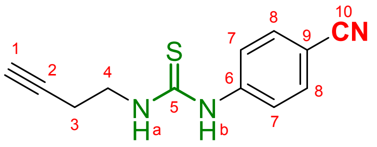

1i

Following the general procedure, thiourea **1i** was obtained after 1 day of reaction, at room temperature, as a yellow solid in 85 % yield (98.0 mg). ^1H NMR (300 MHz, CD_3COCD_3): δ 9.35 (br s, 1H, NH_b), 7.91–7.62 (m, 4H, H-Ar and 1H, NH_b), 3.88–3.71 (m, 2H, H-C_4), 2.58 (td, J = 6.9, 2.7 Hz, 2H, H-C_3), 2.46 (t, J = 2.7 Hz, 1H, H-C_1). $^{13}C\{^1H\}$-APT NMR (75 MHz, CD_3COCD_3): δ 182.0 (s, 1C, C_5), 144.7 (s, 1C, C_6), 133.6 (s, 2C, C_8), 123.1 (s, 2C, C_7), 119.4 (s, 1C, C_9), 107.4 (s, 1C, C_{10}), 82.4 (s, 1C, C_2), 71.3 (s, 1C, C_1), 44.0 (s, 1C, C_4), 18.8 (s, 1C, C_3). HRMS (ESI+) calculated for $C_{12}H_{12}N_3S$ 230.0746; found 230.0741 $[M+H]^+$.

1-(But-3-yn-1-yl)-3-(pyridin-3-yl)thiourea (1j)

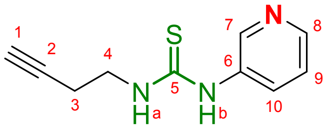

1j

Following the general procedure, thiourea **1j** was obtained after 2 days of reaction, at room temperature, as a yellow solid in 52 % yield (53.4 mg). ^{1}H NMR (300 MHz, $CDCl_3$): δ 8.78–8.34 (m, 3H, H-Ar and NH_a), 7.82 (d, J = 8.1 Hz, 1H, H-Ar), 7.36 (dd, J = 8.2, 4.7 Hz, 1H, H-Ar), 6.69 (br s, 1H, NH_b), 3.79 (q, J = 6.1 Hz, 2H, H-C_4), 2.55 (td, J = 6.3, 2.6 Hz, 2H, H-C_3), 1.99 (t, J = 2.6 Hz, 1H, H-C_1). ^{13}C{^{1}H}-APT NMR (75 MHz, $CDCl_3$): δ 181.3 (s, 1C, C_5), 147.3 (s, 1C, C_8), 145.8 (s, 1C, C_7), 134.0 (s, 1C, C_6), 132.9 (s, 1C, C_{10}), 124.5 (s, 1C, C_9), 81.4 (s, 1C, C_2), 70.7 (s, 1C, C_1), 43.6 (s, 1C, C_4), 18.9 (s, 1C, C_3). HRMS (ESI+) calculated for $C_{10}H_{12}N_3S$ 206.0746; found 206.0737 $[M+H]^+$.

1-(But-3-yn-1-yl)-3-(4-methylphenyl)thiourea (1k)

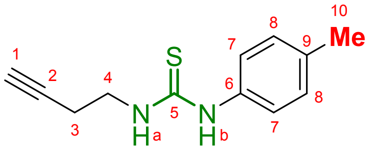

1k

Following the general procedure, thiourea **1k** was obtained after 5 days of reaction, at room temperature, as a yellow solid in 90 % yield (98.2 mg). ^{1}H NMR (300 MHz, CD_3CN): δ 8.06 (br s, 1H, NH_b), 7.28–7.11 (m, 4H, H-Ar), 6.65 (br s, 1H, NH_a), 3.65 (td, J = 6.9, 5.9 Hz, 2H, H-C_4), 2.49 (td, J = 6.9, 2.7 Hz, 2H, H-C_3), 2.33 (s, 3H, H-C_{10}), 2.21 (t, J = 2.7 Hz, 1H, H-C_1). ^{13}C{^{1}H}-APT NMR (75 MHz, CD_3CN): δ 182.4 (s, 1C, C_5), 137.4 (s, 1C, C_6), 131.5 (s, 1C, C_9), 131.0 (s, 2C, C_7), 126.2 (s, 2C, C_8), 82.6 (s, 1C, C_2), 71.0 (s, 1C, C_1), 44.1 (s, 1C, C_4), 21.0 (s, 1C, C_{10}), 19.1 (s, 1C, C_3). HRMS (ESI+) calculated for $C_{12}H_{14}N_2NaS$ 241.0770; found 241.0758 $[M+Na]^+$.

1-(But-3-yn-1-yl)-3-(4-methoxyphenyl)thiourea (1l)

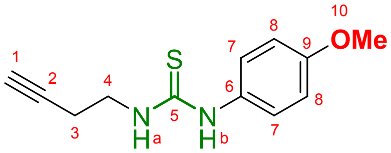

1j

Following the general procedure, thiourea **1l** was obtained after 3 days of reaction, at room temperature, as a yellow solid in 87 % yield (101.8 mg). ^{1}H NMR (300 MHz, $CDCl_3$): δ 7.71 (br s, 1H, NH_b), 7.23–7.12 (m, 2H, H-C_7), 6.99–6.85 (m, 2H, H-C_8), 6.20 (br s, 1H, NH_a), 3.82 (s, 3H, H-C_{10}), 3.77 (q, J = 6.2 Hz, 2H, H-C_4), 2.52 (td, J = 6.3, 2.6 Hz, 2H, H-C_3), 1.91 (t, J = 2.6 Hz, 1H, H-C_1). ^{13}C{^{1}H}-APT NMR (75 MHz, $CDCl_3$): δ 181.4 (s, 1C, C_5), 159.2 (s, 1C, C_9), 128.3 (s, 1C, C_6), 127.9 (s, 2C, C_7), 115.4 (s, 2C, C_8), 81.5 (s, 1C, C_2), 70.3 (s, 1C, C_1), 55.7 (s, 1C, C_{10}), 43.6 (s, 1C, C_4), 19.0 (s, 1C, C_3). HRMS (ESI+) calculated for $C_{12}H_{15}N_2OS$ 235.0900; found 235.0893 $[M+H]^+$.

1-(But-3-yn-1-yl)-3-(naphthalen-1-yl)thiourea (1m)

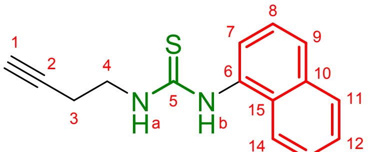

1m

Following the general procedure, thiourea **1m** was obtained after 4 days of reaction, at room temperature, as a yellow solid in 70 % yield (89.0 mg) . ^{1}H NMR (300 MHz, DMSO-d_6): δ 9.74 (br s, 1H, NH_b), 8.02–7.93 (m, 1H, H-Ar), 7.92–7.82 (m, 2H, H-Ar), 7.64 (br s, 1H, NH_a), 7.61–7.44 (m, 4H, H-Ar), 3.59 (td, J = 7.2, 5.7 Hz, 2H, H-C_4), 2.84 (t, J = 2.6 Hz, 1H, H-C_1), 2.45

(td, J = 7.2, 2.7 Hz, 2H, H-C_3). $^{13}C\{^1H\}$-APT NMR (75 MHz, DMSO-d_6): δ 181.8 (s, 1C, C_5), 134.0 (s, 1C, C_6), 129.8 (s, 2C, C_{10}, C_{15}), 128.1 (s, 1C, C_{13}), 126.8 (s, 1C, C_{12}), 126.2 (s, 1C, C_8), 126.2 (s, 1C, C_{11}), 125.7 (s, 1C, C_{14}), 125.1 (s, 1C, C_9), 122.9 (s, 1C, C_7), 82.1 (s, 1C, C_2), 72.2 (s, 1C, C_1), 42.9 (s, 1C, C_4), 18.2 (s, 1C, C_3). HRMS (ESI+) calculated for $C_{15}H_{15}N_2S$ 255.0950; found 255.0952 $[M+H]^+$.

1-Benzyl-3-(but-3-yn-1-yl)thiourea (1n)

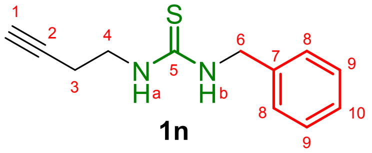

1n

Following the general procedure, thiourea **1n** was obtained after 1 day of reaction, at room temperature, as a yellow solid in 97 % yield (105.9 mg) . ^1H NMR (300 MHz, CD_3COCD_3): δ 7.39 (br s, 1H, NH_b), 7.37–7.20 (m, 5H, H-Ar), 7.13 (br s, 1H, NH_a), 4.78 (br d, J = 5.4 Hz, 2H, H-C_6), 3.70 (q, J = 6.6 Hz, 2H, H-C_4), 2.50 (td, J = 6.9, 2.7 Hz, 2H, H-C_3), 2.38 (t, J = 2.7 Hz, 1H, H-C_1). $^{13}C\{^1H\}$-APT NMR (75 MHz, $CDCl_3$): δ 182.3 (s, 1C, C_5), 136.5 (s, 1C, C_7), 129.1 (s, 2C, C_9), 128.2 (s, 1C, C_{10}), 127.7 (s, 2C, C_8), 81.3 (s, 1C, C_2), 70.6 (s, 1C, C_1), 48.3 (s, 1C, C_6), 43.3 (s, 1C, C_4), 19.1 (s, 1C, C_3). HRMS (ESI+) calculated for $C_{12}H_{15}N_2S$ 219.0950; found 219.0945 $[M+H]^+$.

1-(But-3-yn-1-yl)-3-phenethylthiourea (1o)

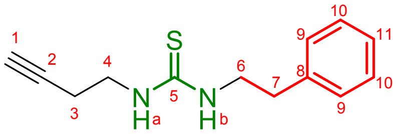

1o

Following the general procedure, thiourea **1o** was obtained after 1 day of reaction, at room temperature, as a yellow solid in 92 % yield (106.9 mg). ^1H NMR (300 MHz, CD_3COCD_3): δ 7.38–7.15 (m, 5H, H-Ar), 7.05 (br s, 2H, $NH_{a,b}$), 3.83–3.70 (m, 2H, H-C_6), 3.69–3.57 (m, 2H, H-C_4), 2.90 (dd, J = 8.2, 6.6 Hz, 2H, H-C_7), 2.48 (td, J = 6.9, 2.7 Hz, 2H, H-C_3), 2.39 (t, J = 2.7 Hz, 1H, H-C_1). $^{13}C\{^1H\}$-APT NMR (75 MHz, CD_3COCD_3): δ 172.6 (s, 1C, C_5), 140.4 (s, 1C, C_8), 129.6 (s, 2C, C_9), 129.2 (s, 2C, C_{10}), 127.0 (s, 1C, C_{11}), 82.6 (s, 1C, C_2), 71.0 (s, 1C, C_1), 46.2 (s, 1C, C_6), 43.7 (s, 1C, C_4), 36.0 (s, 1C, C_7), 19.5 (s, 1C, C_3). HRMS (ESI+) calculated for $C_{13}H_{17}N_2S$ 233.1107; found 233.1107 $[M+H]^+$.

1-(But-3-yn-1-yl)-3-cyclohexylthiourea (1p)

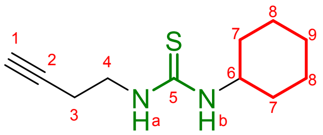

1p

Following the general procedure, thiourea **1p** was obtained after 1 day of reaction, at room temperature, as a yellow solid in 82 % yield (86.3 mg). ^1H NMR (400 MHz, CD_3COCD_3): δ 6.90 (br s, 1H, NH_a), 6.83 (br s, 1H, NH_b), 4.20–3.98 (m, 2H, H-C_6), 3.65 (td, J = 6.8, 5.6 Hz, 2H, H-C_4), 2.47 (td, J = 6.9, 2.7 Hz, 2H, H-C_3), 2.38 (t, J = 2.7 Hz, 1H, H-C_1), 2.03–1.92 (m, 2H, Cy), 1.763–1.65 (m, 2H, Cy), 1.64–1.54 (m, 1H, Cy), 1.42–1.10 (m, 5H, Cy). $^{13}C\{^1H\}$-APT NMR (101 MHz, CD_3COCD_3): δ 183.0 (s, 1C, C_5), 82.7 (s, 1C, C_2), 70.9 (s, 1C, C_1), 53.2 (s, 1C, C_6), 43.6 (s, 1C, C_4), 33.4 (s, 2C, C_y), 26.3 (s, 2C, C_y), 25.7 (s, 1C, C_y), 19.6 (s, 1C, C_3). HRMS (ESI+) calculated for $C_{11}H_{18}N_2NaS$ 233.1083; found 233.1071 $[M+Na]^+$.

1-(3,5-Bis(trifluoromethyl)phenyl)-3-(pent-4-yn-1-yl)thiourea (1q)

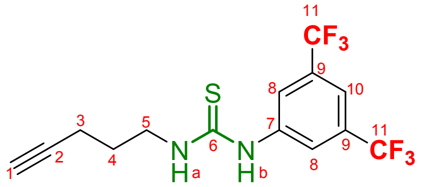

1q

Following the general procedure, thiourea **1q** was obtained after 3 day of reaction, at room temperature, as a yellow solid in 67 % yield (118.9 mg). ^{1}H NMR (400 MHz, CD_3COCD_3): δ 9.34 (br s, 1H, NH_b), 8.31 (s, 2H, H-C_8), 7.78 (br s, 1H, NH_a), 7.71 (s, 1H, H-C_{10}), 3.81–3.66 (m, 2H, H-C_5), 2.39 (t, J = 2.7 Hz, 1H, H-C_1), 2.29 (td, J = 7.2, 2.7 Hz, 2H, H-C_3), 1.88 (q, 2H, H-C_4). ^{13}C{^{1}H}-APT NMR (101 MHz, CD_3COCD_3): δ 182.6 (s, 1C, C_6), 142.9 (s, 1C, C_7), 131.9 (q, J = 28.3 Hz, 2C, C_9), 124.4 (q, J = 270.0 Hz, 2C, C_{11}), 123.8–123.1 (m, 1C, C_{10}), 117.7–117.3 (m, 2C, C_8), 84.1 (s, 1C, C_2), 70.4 (s, 1C, C_1), 44.2 (s, 1C, C_5), 28.4 (s, 2C, C_3), 16.4 (s, 1C, C_4). HRMS (ESI+) calculated for $C_{14}H_{13}F_6N_2S$ 355.0698; found 355.0694 $[M+H]^+$.

3. General procedure for the synthesis of 1,3-thiazines 2a-r'

To a solution of catalyst **IC** (0.01 equiv., 0.001 mmol, 0.8 mg) in CH_2Cl_2 (0.5 mL), the corresponding thiourea **1** (1 equiv., 0.1 mmol, 0.2 M) is added. The reaction mixture is stirred at room temperature between 4 and 5 hours —in most cases— and the course of the reaction if followed by TLC (*n*-hexane:ethyl acetate 5:5). All 1,3-thiazines **2'** are obtained as white solids after a column chromatography (*n*-hexane:ethyl acetate 7:3).

For a higher scale, to a solution of catalyst **IC** (0.01 equiv., 0.01 mmol, 8 mg) in CH_2Cl_2 (2.5 mL), the corresponding thiourea **1a** (1 equiv., 1 mmol, 0.4 M) is added. The reaction mixture is stirred at room temperature during 24 h, and the course of the reaction if followed by TLC (*n*-hexane:ethyl acetate 5:5). 1,3-thiazine **2a'** is obtained as white solid after a column chromatography (*n*-hexane:ethyl acetate 7:3) in 83 % (169.6 mg).

The characterizations are performed by ^{1}H NMR, ^{13}C{^{1}H}-APT, COSY, HSQC, HMBC and HRMS in solution. Indeed, the characterized species are the tautomers of **5,6-dihydro-4*H*-1,3-thiazine 2'**. All yields after purification and reaction times are shown in the **Figure 7**.

6-Methylene-*N*-phenyl-5,6-dihydro-4*H*-1,3-thiazin-2-amine (2a')

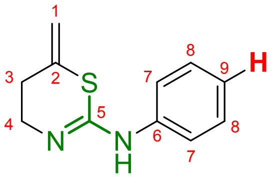

2a'

Following the general procedure, compound **2a'** was obtained after 5 hours of reaction, at 60 ºC, as a yellow solid in 99 % yield (20.2 mg). ^{1}H NMR (300 MHz, $CDCl_3$): δ 7.38–7.22 (m, 2H, H-Ar), 7.16–6.92 (m, 3H, H-Ar), 6.56 (br s, 1H, NH), 5.08–4.89 (m, 2H, H-C_1), 3.54–3.33 (m, 2H, H-C_4), 2.65–2.49 (m, 2H, H-C_3). ^{13}C{^{1}H}-APT NMR (75 MHz, $CDCl_3$): δ 151.1 (s, 1C, C_5), 145.5 (s, 1C, C_6), 138.1 (s, 1C, C_2), 129.0 (s, 2C, C_7), 123.2 (s, 2C, C_8), 121.9 (s, 1C, C_9), 109.7 (s, 1C, C_1),

43.5 (s, 1C, C4), 32.0 (s, 1C, C_3). HRMS (ESI+) calculated for $C_{11}H_{13}N_2S$ 205.0794; found 205.0790 $[M+H]^+$.

_N_-(4-Fluorophenyl)-6-methylene-5,6-dihydro-4_H_-1,3-thiazin-2-amine (**2b'**)

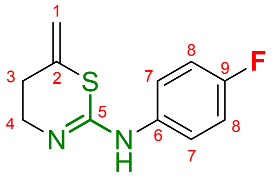

2b'

Following the general procedure, compound **2b'** was obtained after 5.5 hours of reaction, at 60 ºC, as a yellow solid in 86 % yield (19.1 mg). ^{1}H NMR (300 MHz, CD_3COCD_3): δ 7.61–7.22 (m, 2H, H-Ar), 7.04–6.92 (m, 2H, H-Ar), 5.10 (t, J = 1.6 Hz, 1H, H-C_1), 4.98 (t, J = 1.3 Hz, 1H, H-C_1), 3.68–3.43 (m, 2H, H-C_4), 2.84 (br s, 1H, NH), 2.61–2.45 (m, 2H, H-C_3). ^{13}C{^{1}H}-APT NMR (75 MHz, CD_3COCD_3): δ 158.9 (d, J = 238.9 Hz, 1C, C_9), 155.2 (s, 1C, C_5), 140.1 (s, 1C, C_6), 122.3 (m, 2C, C_7), 120.9 (s, 1C, C_2), 115.7 (d, J = 22.1 Hz, 2C, C_8), 109.0 (s, 1C, C_1), 45.3 (s, 1C, C_4), 31.5 (s, 1C, C_3). HRMS (ESI+) calculated for $C_{11}H_{12}FN_2S$ 223.0700; found 223.0692 $[M+H]^+$.

_N_-(3-Fluorophenyl)-6-methylene-5,6-dihydro-4_H_-1,3-thiazin-2-amine (**2c'**)

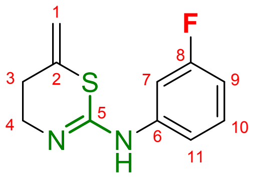

2c'

Following the general procedure, compound **2c'** was obtained after 4 hours of reaction, at 60 ºC, as a yellow solid in 99 % yield (22.0 mg). ^{1}H NMR (300 MHz, $CDCl_3$): δ 7.26–7.16 (m, 1H, H-Ar), 6.93 (dt, J = 10.8, 2.2 Hz, 1H, H-Ar), 6.84–6.68 (m, 2H, H-Ar), 5.23 (br s, 1H, NH), 5.02 (t, J = 1.2 Hz, 1H, H-C_1), 4.96 (q, J = 1.1 Hz, 1H, H-C_1), 3.53–3.44 (m, 2H, H-C_4), 2.65–2.56 (m, 2H, H-C_3). ^{13}C{^{1}H}-APT NMR (75 MHz, CD_3COCD_3): δ 163.9 (d, J = 241.1 Hz, 1C, C_8), 146.5 (s, 1C, C_5), 139.9 (s, 2C, C_6 and C_2), 130.6 (d, J = 9.7 Hz, 1C, C_{11}), 116.2 (br s, 1C, C_{10}), 109.2 (s, 1C, C_1), 108.7 (d, J = 21.6 Hz, 1C, C_7), 107.5 (d, J = 25.7 Hz, 1C, C_9), 45.9 (s, 1C, C_4), 31.2 (s, 1C, C_3). HRMS (ESI+) calculated for $C_{11}H_{12}FN_2S$ 223.0700; found 223.0693 $[M+H]^+$.

_N_-(4-Chlorophenyl)-6-methylene-5,6-dihydro-4_H_-1,3-thiazin-2-amine (**2d'**)

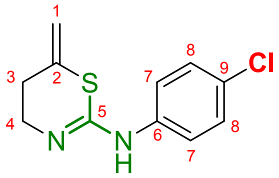

2d'

Following the general procedure, compound **2d'** was obtained after 4 hours of reaction, at 60 ºC, as a yellow solid in 92 % yield (22.0 mg). ^{1}H NMR (300 MHz, CD_3COCD_3): δ 7.45–7.36 (m, 2H, H-C_8), 7.32–7.23 (m, 2H, H-C_7), 5.18 (td, J = 1.5, 0.6 Hz, 1H, H-C_1), 5.07 (q, J = 1.1 Hz, 1H, H-C_1), 3.64–3.54 (m, 2H, H-C_4), 3.02 (br s, 1H, NH), 2.64–2.54 (m, 2H, H-C_3). ^{13}C{^{1}H}-APT NMR (75 MHz, $CDCl_3$): δ 152.2 (s, 1C, C_5), 143.5 (s, 1C, C_6), 137.2 (s, 1C, C_2), 129.2 (s, 2C, C_8), 128.7 (s, 1C, C_9), 123.5 (s, 2C, C_7), 110.5 (s, 1C, C_1), 43.2 (s, 1C, C_4), 31.9 (s, 1C, C_3). HRMS (ESI+) calculated for $C_{11}H_{12}ClN_2S$ 239.0404; found 239.0407 $[M+H]^+$.

N-(4-Bromophenyl)-6-methylene-5,6-dihydro-4H-1,3-thiazin-2-amine (**2e'**)

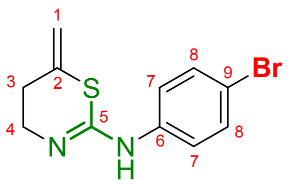

2e'

Following the general procedure, compound **2e'** was obtained after 4 hours of reaction, at 60 ºC, as a yellow solid in 99 % yield (28.0 mg). ^{1}H NMR (400 MHz, $CDCl_3$): δ 7.41–7.33 (m, 2H, H-C_8), 7.00–6.91 (m, 2H, H-C_7), 5.94 (br s, 1H, NH), 5.05 (s, 1H, H-C_1), 5.00 (s, 1H, H-C_1), 3.47–3.40 (m, 2H, H-C_4), 2.64–2.56 (m, 2H, H-C_3). ^{13}C{^{1}H}-APT NMR (101 MHz, $CDCl_3$): δ 151.6 (s, 1C, C_5), 144.9 (s, 1C, C_6), 137.6 (s, 1C, C_2), 132.0 (s, 2C, C_8), 123.8 (s, 2C, C_7), 115.9 (s, 1C, C_9), 110.2 (s, 1C, C_1), 43.1 (s, 1C, C_4), 32.1 (s, 1C, C_3). HRMS (ESI+) calculated for $C_{11}H_{12}BrN_2S$ 282.9899; found 282.9910 $[M+H]^+$.

6-Methylene-N-(4-(trifluoromethyl)phenyl)-5,6-dihydro-4H-1,3-thiazin-2-amine (**2f'**)

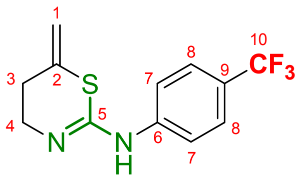

2f'

Following the general procedure, compound **2f'** was obtained after 5 hours of reaction, at 60 ºC, as a yellow solid in 78 % yield (21.2 mg). ^{1}H NMR (400 MHz, CD_3COCD_3): δ 7.76–7.58 (m, 2H, H-Ar), 7.55 (d, J = 8.7 Hz, 2H, H-Ar), 5.18–5.11 (m, 1H, H-C_1), 5.06–4.99 (m, 1H, H-C_1), 3.68–3.55 (m, 2H, H-C_4), 2.85 (br s, 1H, NH), 2.54 (ddt, J = 7.1, 3.2, 1.4 Hz, 2H, H-C_3). ^{13}C{^{1}H}-APT NMR (75 MHz, $CDCl_3$): δ 155.2 (s, 1C, C_5), 150.1 (d, J = 204.8 Hz, 1C, C_{10}), 137.3 (s, 1C, C_2), 130.2 (s, 1C, C_6), 126.3 (s, 2C, C_8), 123.8 (d, J = 151.0 Hz, 1C, C_9), 121.8 (s, 2C, C_7), 110.4 (s, 1C, C_1), 43.4 (s, 1C, C_4), 31.9 (s, 1C, C_3). HRMS (ESI+) calculated for $C_{12}H_{12}F_3N_2S$ 273.0668; found 273.0674 $[M+H]^+$.

N-(3,5-Bis(trifluoromethyl)phenyl)-6-methylene-5,6-dihydro-4H-1,3-thiazin-2-amine (**2g'**)

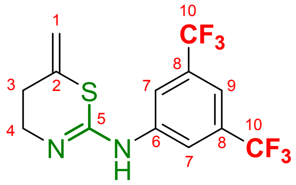

2g'

Following the general procedure, compound **2g'** was obtained after 4.5 hours of reaction, at 60 ºC, as a yellow solid in 91 % yield (31.0 mg). ^{1}H NMR (300 MHz, $CDCl_3$): δ 7.52 (s, 1H, H-C_9), 7.48 (d, J = 1.6 Hz, 2H, H-C_7), 5.76 (br s, 1H, NH), 5.13–5.11 (m, 1H, H-C_1), 5.06–5.04 (m, 1H, H-C_1), 3.51-3.42 (m, 2H, H-C_4), 2.71-2.62 (m, 2H, H-C_3). ^{13}C{^{1}H}-APT NMR (75 MHz, $CDCl_3$): δ 153.2 (s, 1C, C_5), 148.3 (s, 1C, C_6), 136.5 (s, 1C, C_2), 132.3 (q, J = 32.9 Hz, 2C, C_8), 125.3 (q, J = 272.0 Hz, 2C, C_{10}), 122.6 (s, 2C, C_7), 116.5 (hept, J = 4.0 Hz, 1C, C_9), 111.2 (s, 1C, C_1), 42.6 (s, 1C, C_4), 32.2 (s, 1C, C_3). HRMS (ESI+) calculated for $C_{13}H_{11}F_6N_2S$ 341.0542; found 341.0530 $[M+H]^+$.

6-Methylene-*N*-(4-nitrophenyl)-5,6-dihydro-4*H*-1,3-thiazin-2-amine (2h')

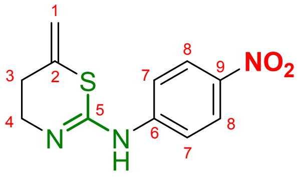

2h'

Following the general procedure, compound **2h'** was obtained after 4 hours of reaction, at 60 ºC, as a yellow solid in 99 % yield (26.1 mg). ^{1}H NMR (300 MHz, CD_3COCD_3): δ 8.13 (d, *J* = 9.3 Hz, 2H, H-Ar), 7.86–7.52 (m, 2H, H-Ar), 5.21–5.12 (m, 1H, H-C_1), 5.09–4.99 (m, 1H, H-C_1), 3.74–3.59 (m, 2H, H-C_4), 2.62–2.47 (m, 2H, H-C_3). $^{13}C\{^{1}H\}$-APT NMR (75 MHz, CD_3COCD_3): δ 148.5 (s, 1C, C_5), 142.4 (s, 1C, C_6), 139.3 (s, 1C, C_2), 125.6 (s, 2C, C_8), 120.1 (s, 2C, C_7), 113.3 (s, 1C, C_9), 109.8 (s, 1C, C_1), 46.0 (s, 1C, C_4), 30.8 (s, 1C, C_3). HRMS (ESI+) calculated for $C_{11}H_{11}N_3NaOS$ 272.0464; found 272.0448 $[M+Na]^+$.

4-((6-methylene-5,6-dihydro-4*H*-1,3-thiazin-2-yl)amino)benzonitrile (2i')

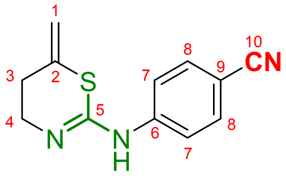

2i'

Following the general procedure, compound **2i'** was obtained after 24 hours of reaction, at 60 ºC, as a yellow solid in 85 % yield (19.0 mg). ^{1}H NMR (400 MHz, CD_3COCD_3): δ 7.71–7.48 (m, 4H, H-Ar), 5.15 (td, *J* = 1.5, 0.6 Hz, 1H, H-C_1), 5.03 (t, *J* = 0.9 Hz, 1H, H-C_1), 3.68–3.57 (m, 2H, H-C_4), 2.86 (br s, 1H, NH), 2.58–2.48 (m, 2H, H-C_3). $^{13}C\{^{1}H\}$-APT NMR (101 MHz, CD_3COCD_3): 139.4 (s, 1C, C_6), 133.7 (s, 2C, C_8), 120.8 (s, 2C, C_7), 119.9 (s, 1C, C_9), 114.8 (s, 1C, C_2), 109.6 (s, 1C, C_1), 104.9 (s, 1C, C_{10}), 45.9 (s, 1C, C_4), 30.9 (s, 1C, C_3). HRMS (ESI+) calculated for $C_{12}H_{12}N_3S$ 230.0746; found 230.0744 $[M+H]^+$.

6-Methylene-*N*-(pyridin-3-yl)-5,6-dihydro-4*H*-1,3-thiazin-2-amine (2j')

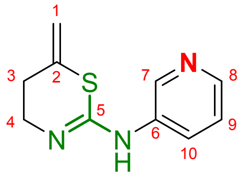

2j'

Following the general procedure, compound **2j'** was obtained after 4.5 hours of reaction, at 60 ºC, as a yellow solid in 99 % yield (20.3 mg). ^{1}H NMR (300 MHz, $CDCl_3$): δ 8.31 (d, *J* = 2.6 Hz, 1H, H-C_7), 8.28 (dd, *J* = 4.8, 1.5 Hz, 1H, H-C_8), 7.44 (ddd, *J* = 8.1, 2.6, 1.5 Hz, 1H, H-C_{10}), 7.20 (dd, *J* = 8.1, 4.7 Hz, 1H, H-C_9), 5.69 (br s, 1H, NH), 5.09–5.07 (m, 1H, H-C_1), 5.03-5.00 (m, 1H, H-C_1), 3.50–3.42 (m, 2H, H-C_4), 2.67–2.60 (m, 2H, H-C_3). $^{13}C\{^{1}H\}$-APT NMR (75 MHz, $CDCl_3$): δ 153.7 (s, 1C, C_5), 144.6 (s, 1C, C_7), 144.2 (s, 1C, C_8), 142.1 (s, 1C, C_6), 136.7 (s, 1C, C_2), 129.8 (s, 1C, C_{10}), 123.7 (s, 1C, C_9), 111.0 (s, 1C, C_1), 42.7 (s, 1C, C_4), 32.1 (s, 1C, C_3). HRMS (ESI+) calculated for $C_{10}H_{12}N_3S$ 206.0746; found 206.0746 $[M+H]^+$.

6-Methylene-*N*-(4-methylphenyl)-5,6-dihydro-4*H*-1,3-thiazin-2-amine (2k')

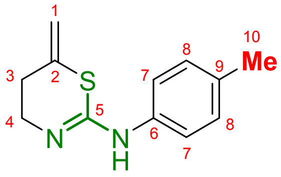

2k'

Following the general procedure, compound **2k'** was obtained after 24 hours of reaction, at 60 ºC, as a yellow solid in 92 % yield (20.1 mg). ^{1}H NMR (300 MHz, $CDCl_3$): δ 7.14–6.96 (m, 4H, H-Ar), 5.08–5.04 (m, 1H, H-C_1), 5.02–4.99 (m, 1H, H-C_1), 4.86 (br s, 1H, NH), 3.54–3.44 (m, 2H, H-C_4), 2.65–2.54 (m, 2H, H-C_3), 2.31 (s, 3H, H-C_{10}). $^{13}C\{^{1}H\}$-APT NMR (75 MHz, $CDCl_3$): δ

152.1 (s, 1C, C_5), 141.5 (s, 1C, C_6), 137.7 (s, 1C, C_2), 133.3 (s, 1C, C_9), 129.7 (s, 2C, C_8), 122.2 (s, 2C, C_7), 110.1 (s, 1C, C_1), 43.5 (s, 1C, C_4), 31.9 (s, 1C, C_3), 21.0 (s, 1C, C_{10}). HRMS (ESI+) calculated for $C_{12}H_{15}N_2S$ 219.0950; found 219.0948 $[M+H]^+$.

***N*-(4-Methoxyphenyl)-6-methylene-5,6-dihydro-4*H*-1,3-thiazin-2-amine (2l')**

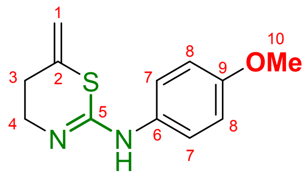

2l'

Following the general procedure, compound **2l'** was obtained after 20 hours of reaction, at 60 ºC, as a yellow solid in 84 % yield (19.4 mg). ^{1}H NMR (400 MHz, $CDCl_3$): δ 7.10–6.97 (m, 2H, H-Ar), 6.88–6.79 (m, 2H, H-Ar), 5.06–5.03 (m, 1H, H-C_1), 5.02–4.96 (m, 1H, H-C_1), 3.78 (s, 3H, H-C_{10}), 3.57–3.43 (m, 2H, H-C_4), 2.63–2.53 (m, 2H, H-C_3). $^{13}C\{^1H\}$-APT NMR (101 MHz, $CDCl_3$): δ 156.0 (s, 1C, C_5), 149.5 (s, 1C, C_9), 138.2 (s, 1C, C_2), 123.4 (s, 2C, C_7), 114.3 (s, 2C, C_8), 112.7 (s, 1C, C_6), 109.7 (s, 1C, C_1), 55.6 (s, 1C, C_{10}), 44.1 (s, 1C, C_4), 29.8 (s, 1C, C_3). HRMS (ESI+) calculated for $C_{12}H_{15}N_2OS$ 235.0900; found 235.0902 $[M+H]^+$.

6-Methylene-*N*-(naphthalen-1-yl)-5,6-dihydro-4*H*-1,3-thiazin-2-amine (2m')

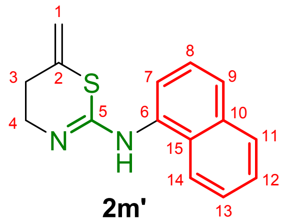

2m'

Following the general procedure, compound **2m'** was obtained after 20 hours of reaction, at 60 ºC, as a yellow solid in 83 % yield (21.1 mg). ^{1}H NMR (300 MHz, $CDCl_3$): δ 8.10–8.01 (m, 1H, H-Ar), 7.88–7.79 (m, 1H, H-Ar), 7.59 (d, J = 8.2 Hz, 1H, H-Ar), 7.52–7.40 (m, 3H, H-Ar), 7.08 (d, J = 7.3 Hz, 1H, H-Ar), 4.97 (s, 1H, H-C_1), 4.91 (s, 1H, H-C_1), 3.29 (t, J = 5.5 Hz, 2H, H-C_4), 2.63 (t, J = 5.5 Hz, 2H, H-C_3). $^{13}C\{^1H\}$-APT NMR (101 MHz, $CDCl_3$) δ 153.3 (s, 1C, C_5), 144.3 (s, 1C, C_6), 137.9 (s, 1C, C_2), 134.6 (s, 1C, C_{13}), 128.1 (s, 1C, C_{11}), 126.1 (s, 1C, C_{12}), 126.0 (s, 1C, C_8), 125.5 (s, 1C, C_7), 123.8 (s, 1C, C_{14}), 123.6 (s, 1C, C_9), 118.5 (br s, 2C, C_{10}, C_{15}), 110.0 (s, 1C, C_1), 41.9 (s, 1C, C_4), 33.1 (s, 1C, C_3). HRMS (ESI+) calculated for $C_{15}H_{15}N_2S$ 255.0950; found 255.0939 $[M+H]^+$.

***N*-Benzyl-6-methylene-5,6-dihydro-4*H*-1,3-thiazin-2-amine (2n')**

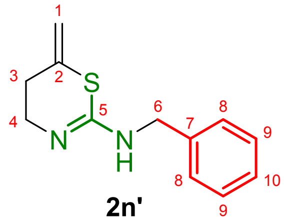

2n'

Following the general procedure, compound **2n'** was obtained after 19 hours of reaction, at 60 ºC, as a yellow solid in 99 % yield (21.6 mg). ^{1}H NMR (300 MHz, $CDCl_3$): δ 7.32–7.16 (m, 5H, H-Ar), 5.01 (t, J = 1.6 Hz, 1H, H-C_1), 4.97–4.91 (m, 1H, H-C_1), 4.38 (s, 2H, H-C_6), 4.15 (br s, 1H, NH), 3.60–3.49 (m, 2H, H-C_4), 2.41–2.31 (m, 2H, H-C_3). $^{13}C\{^1H\}$-APT NMR (75 MHz, $CDCl_3$): δ 150.9 (s, 1C, C_5), 138.6 (s, 1C, C_2), 138.5 (s, 1C, C_7), 128.8 (s, 2C, C_9), 128.0 (s, 2C, C_8), 127.6 (s, 1C, C_{10}), 108.7 (s, 1C, C_1), 46.6 (s, 1C, C_4), 30.5 (s, 1C, C_3), 29.8 (s, 1C, C_6). HRMS (ESI+) calculated for $C_{12}H_{15}N_2S$ 219.0950; found 219.0943 $[M+H]^+$.

6-Methylene-*N*-phenethyl-5,6-dihydro-4*H*-1,3-thiazin-2-amine (2o')

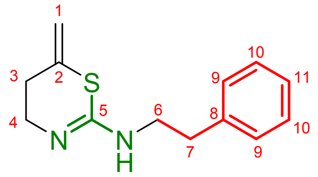

2o'

Following the general procedure, compound **2o'** was obtained after 24 hours of reaction, at 60 ºC, as a yellow solid in 99 % yield (22.4 mg). ^{1}H NMR (300 MHz, $CDCl_3$): δ 7.41–7.09 (m, 5H, H-Ar), 5.04 (t, *J* = 1.6 Hz, 1H, H-C_1), 4.92 (t, *J* = 1.3 Hz, 1H, H-C_1), 3.58–3.38 (s, 4H, H-C_6, H-C_4), 2.85 (dd, *J* = 8.3, 6.5 Hz, 2H, H-C_7), 2.41–2.30 (m, 2H, H-C_3). ^{13}C{^{1}H}-APT NMR (75 MHz, $CDCl_3$): δ 149.3 (s, 1C, C_5), 140.9 (s, 1C, C_2), 140.8 (s, 1C, C_8), 129.6 (s, 2C, C_{10}), 129.1 (s, 2C, C_9), 126.8 (s, 1C, C_{11}), 107.8 (s, 1C, C_1), 47.2 (s, 1C, C_4), 44.4 (s, 1C, C_7), 36.1 (s, 1C, C_3), 31.1 (s, 1C, C_6). HRMS (ESI+) calculated for $C_{13}H_{17}N_2S$ 233.1107; found 233.1101 $[M+H]^+$.

***N*-Cyclohexyl-6-methylene-5,6-dihydro-4*H*-1,3-thiazin-2-amine (2p')**

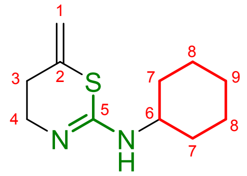

2p'

Following the general procedure, compound **2p'** was obtained after 20 hours of reaction, at 60 ºC, as a yellow solid in 99 % yield (20.8 mg). ^{1}H NMR (400 MHz, CD_3COCD_3): 5.21 (br s, 1H, NH), 5.01 (t, *J* = 1.6 Hz, 1H, H-C_1), 4.89 (t, *J* = 1.3 Hz, 1H, H-C_1), 3.67 (tt, *J* = 10.4, 3.8 Hz, 1H, H-C_6), 3.53–3.41 (m, 2H, H-C_4), 2.38–2.26 (m, 2H, H-C_3), 2.01–1.89 (m, 2H, H-C_y), 1.76–1.51 (m, 3H, H-C_y), 1.42–1.05 (m, 5H, H-C_y). ^{13}C{^{1}H}-APT NMR (101 MHz, CD_3COCD_3): δ 148.4 (s, 1C, C_5), 141.2 (s, 1C, C_2), 107.5 (s, 1C, C_1), 51.2 (s, 1C, C_6), 47.2 (s, 1C, C_4), 33.7 (s, 2C, C_7), 31.1 (s, 1C, C_3), 26.6 (s, 2C, C_8), 25.9 (s, 1C, C_9). HRMS (ESI+) calculated for $C_{11}H_{19}N_2S$ 211.1263; found 211.1267 $[M+H]^+$.

***N*-cyclohexyl-*N*-methyl-6-methylene-5,6-dihydro-4*H*-1,3-thiazin-2-amine (2q')**

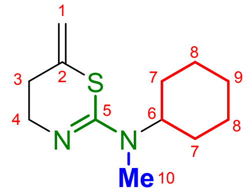

2r'

To a mixture of **2p'** (0.1 mmol, 21.0 mg, 0.2 M) and K_2CO_3 (2 equiv.) in acetone, MeI (0.2 mmol) was added. The reaction was followed at room temperature without stirring by NMR. After 24 h, the reaction was obtained as a yellow liquid in a quantitative conversion. ^{1}H NMR (300 MHz, CD_3COCD_3): 5.05 (t, *J* = 1.6 Hz, 1H, H-C_1), 5.03 (t, *J* = 1.6 Hz, 1H, H-C_1), 3.39–3.27 (m, 3H, H-C_4 and H-C_6), 2.97 (s, 3H, H-C_{10}), 2.75 (ddt, *J* = 7.2, 4.6, 1.6 Hz, 2H, H-C_3), 1.81–1.50 (m, 5H, H-C_y), 1.41–1.14 (m, 5H, H-C_y). ^{13}C{^{1}H}-APT NMR (101 MHz, CD_3COCD_3): δ 153.8 (s, 1C, C_5), 133.7 (s, 1C, C_2), 109.4 (s, 1C, C_1), 57.9 (s, 1C, C_6), 50.6 (s, 1C, C_4), 40.0 (s, 1C, C_{10}), 35.4 (s, 2C, C_7), 34.1 (s, 1C, C_3), 26.6 (s, 2C, C_8), 25.4 (s, 1C, C_9). HRMS (ESI+) calculated for $C_{12}H_{21}N_2S$ 225.1420; found 225.1416 $[M+H]^+$.

4. Synthesis of catalyst [Au(NCMe)(CyJohnPhos)]SbF_6 (IC)

To a solution of [AuCl(tht)] (1 equiv., 1.0 mmol, 321.2 mg, 0.1 M) in CH_2Cl_2 (10 mL), CyJohnPhos (1 equiv., 1.0 mmol, 357.9 mg, 0.1 M) is added. The reaction was carried out at room temperature during 30 min. After the indicated reaction time, the solvent was evaporated, precipitated with hexane, and filtered to give [AuCl(CyJohnPhos)] as a white solid. Then, to a solution of [AuCl(CyJohnPhos)] (1 equiv., 0.52 mmol, 303.0 mg, 0.035 M) in MeCN (15 mL), $AgSbF_6$ (1 equiv., 0.52 mmol, 182.7 mg, 0.035 M) was added to remove Cl^- and coordinate an NCMe ligand to the complex. The reaction was carried out at room temperature during 1 h. The generated AgCl is filtered by diatomaceous earth and washed with MeCN. The solvent was evaporated under vacuum, dissolved in CH_2Cl_2, precipitated with hexane and filtered, in a nº4 plate, to give a white solid in 82 % yield (351.5 mg).

The characterizations are performed by ^{1}H-NMR, ^{13}C{^{1}H}-APT, COSY, HSQC and HRMS in solution.

[Au(NCMe)(CyJohnPhos)]SbF_6 (**IC**)

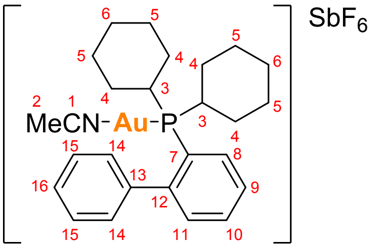

^{1}H NMR (400 MHz, CD_2Cl_2): δ 7.66–7.57 (m, 3H, H-$C_{15,16}$), 7.55–7.49 (m, 3H, H-Ar), 7.41–7.31 (m, 1H, H-Ar), 7.27–7.18 (m, 2H, H-C_{14}), 2.38 (m, 3H, H-C_2), 2.34–2.20 (m, 2H, H-Cy), 2.05–1.95 (m, 2H, H-Cy), 1.91– 1.83 (m, 2H, H-Cy), 1.83–1.75 (m, 2H, H-Cy), 1.74–1.68 (m, 1H, H-Cy), 1.46–1.11 (m, 12H, H-Cy), 0.93–0.78 (m, 1H, H-Cy). ^{31}P-NMR (162 MHz, CD_2Cl_2) δ 35.10 (s, 1P). ^{13}C{^{1}H}-APT NMR (101 MHz, CD_2Cl_2): δ 149.3 (d, J = 11.8 Hz, 1C, C_7), 142.4 (d, J = 6.9 Hz, 1C, C_{12}), 132.98 (d, J = 7.6 Hz, 1C, C_8), 132.6 (d, J = 4.8 Hz, 1C, C_{11}), 131.9 (d, J = 2.4 Hz, 1C, C_{10}), 130.2 (s, 2C, C_{15}), 129.4 (s, 2C, C_{14}), 128.7 (d, J = 8.5 Hz, 1C, C_9), 128.3 (s, 1C, C_{16}), 122.75 (s, 1C, C_{13}), 119.3 (s, 1C, C_1), 36.4 (d, J = 35.8 Hz, 2C, C_3), 31.5 (d, J = 2.8 Hz, 4C, C_4), 26.8 (d, J = 9.8 Hz, 2C, C_5), 26.7 (d, J = 11.4 Hz, 2C, C_5), 26.0 (d, J = 1.8 Hz, 2C, C_6), 2.8 (s, 1C, C_2). HRMS (ESI+) calculated for $C_{26}H_{34}AuNP$ 588.2089; found 588.2089 [M-SbF_6]$^+$.

5. ^{1}H, ^{13}C{^{1}H}-APT, COSY, HSQC and HMBC NMR spectra

5.1. Spectra of thioureas 1a-q

Figure S1. ^{1}H NMR (400 MHz, $CDCl_3$) spectrum of 1-(but-3-yn-1-yl)-3-phenylthiourea (**1a**)

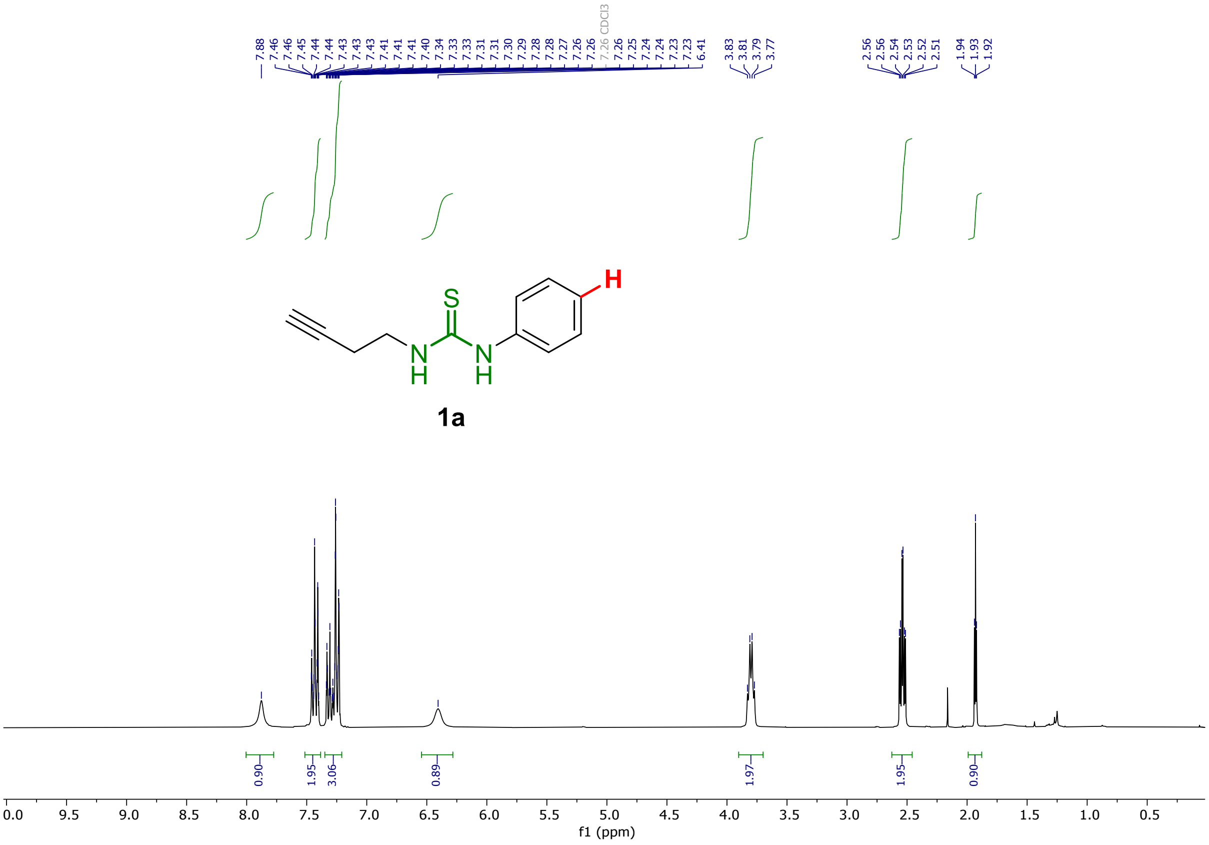

Figure S2. ^{13}C{^{1}H}-APT NMR (75 MHz, $CDCl_3$) spectrum of thiourea **1a**

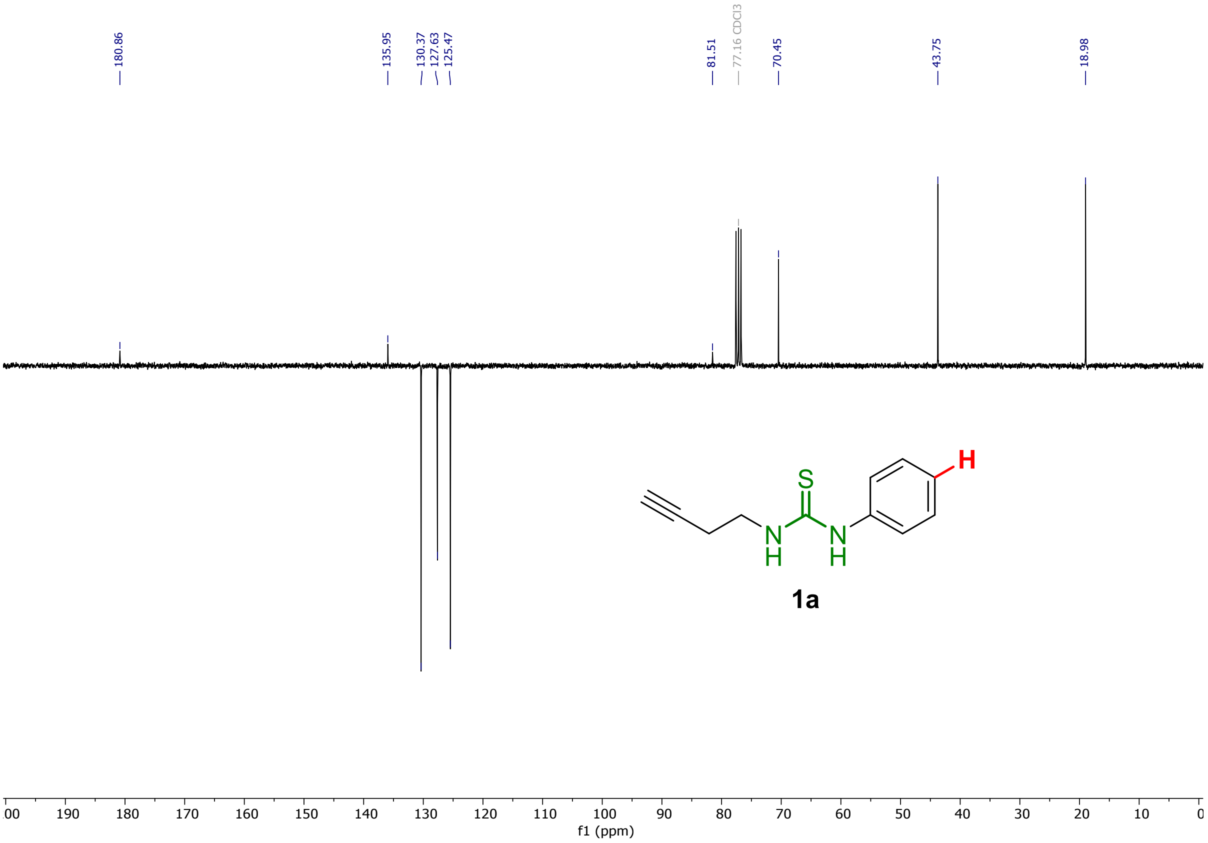

Figure S3. ^{1}H NMR (400 MHz, $CDCl_3$) spectrum of 1-(but-3-yn-1-yl)-3-(4-fluorophenyl)thiourea (**1b**)

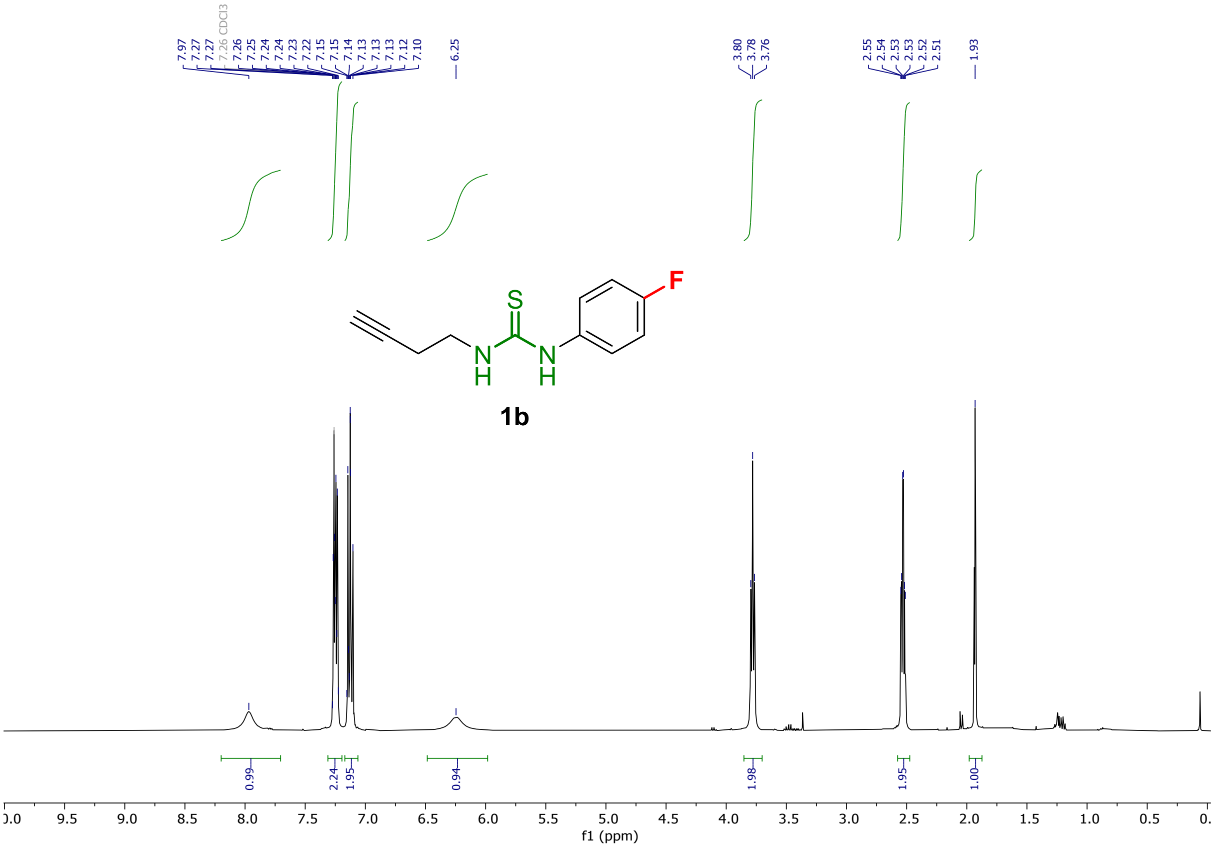

Figure S4. $^{13}C\{^{1}H\}$-APT NMR (101 MHz, $CDCl_3$) spectrum of thiourea **1b**

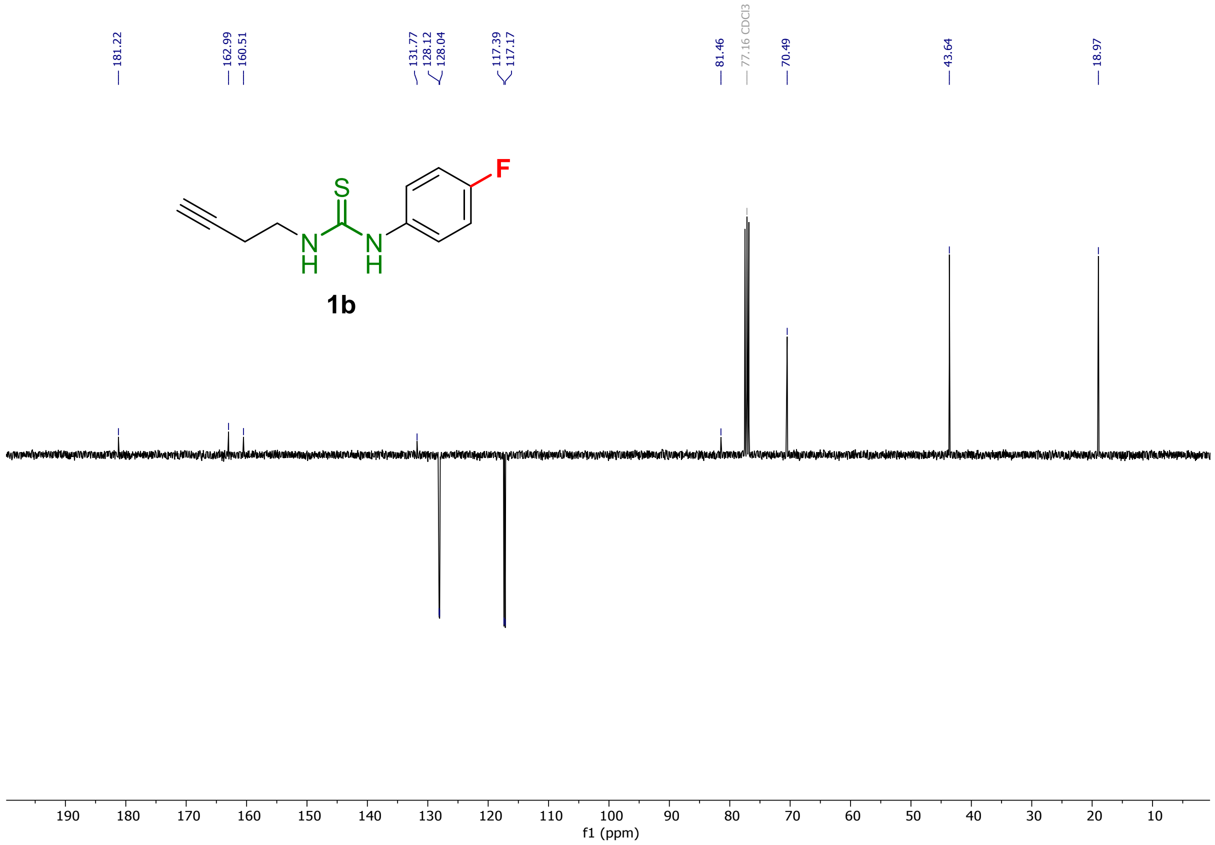

Figure S5. COSY NMR (400 MHz, $CDCl_3$) spectrum of thiourea **1b**

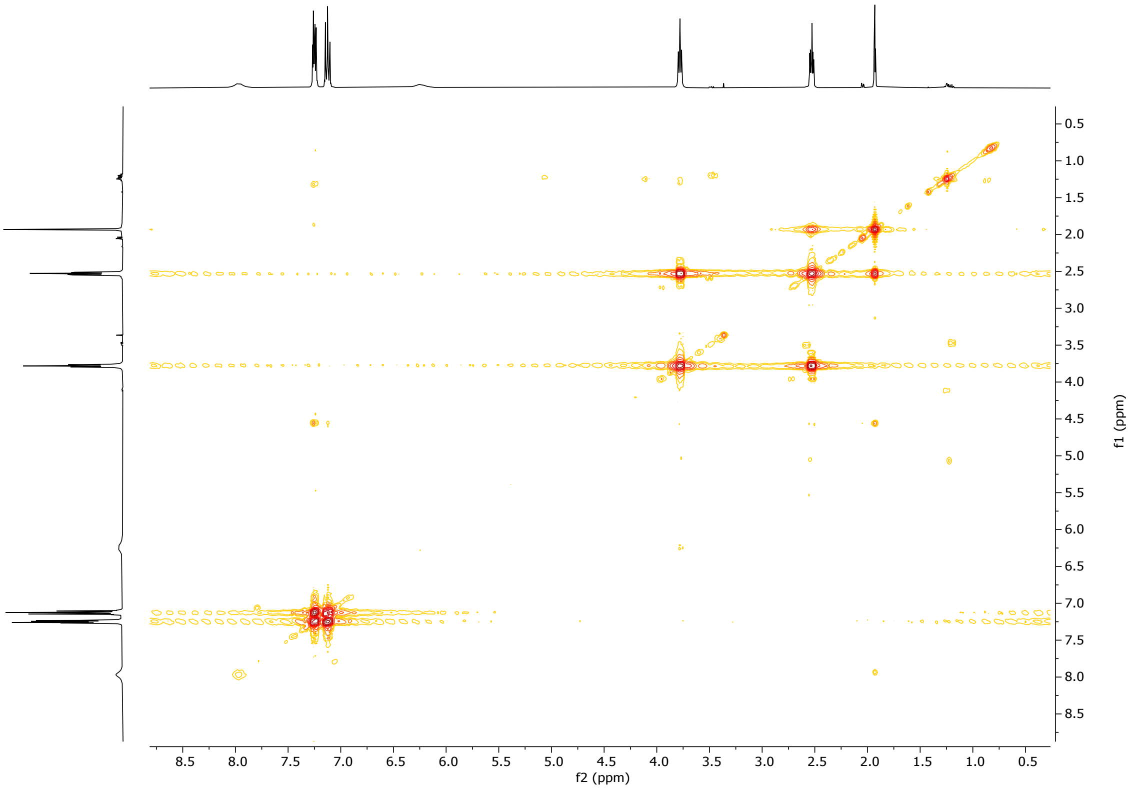

Figure S6. ^{1}H NMR (300 MHz, $CDCl_3$) spectrum of 1-(but-3-yn-1-yl)-3-(3-fluorophenyl)thiourea (**1c**)

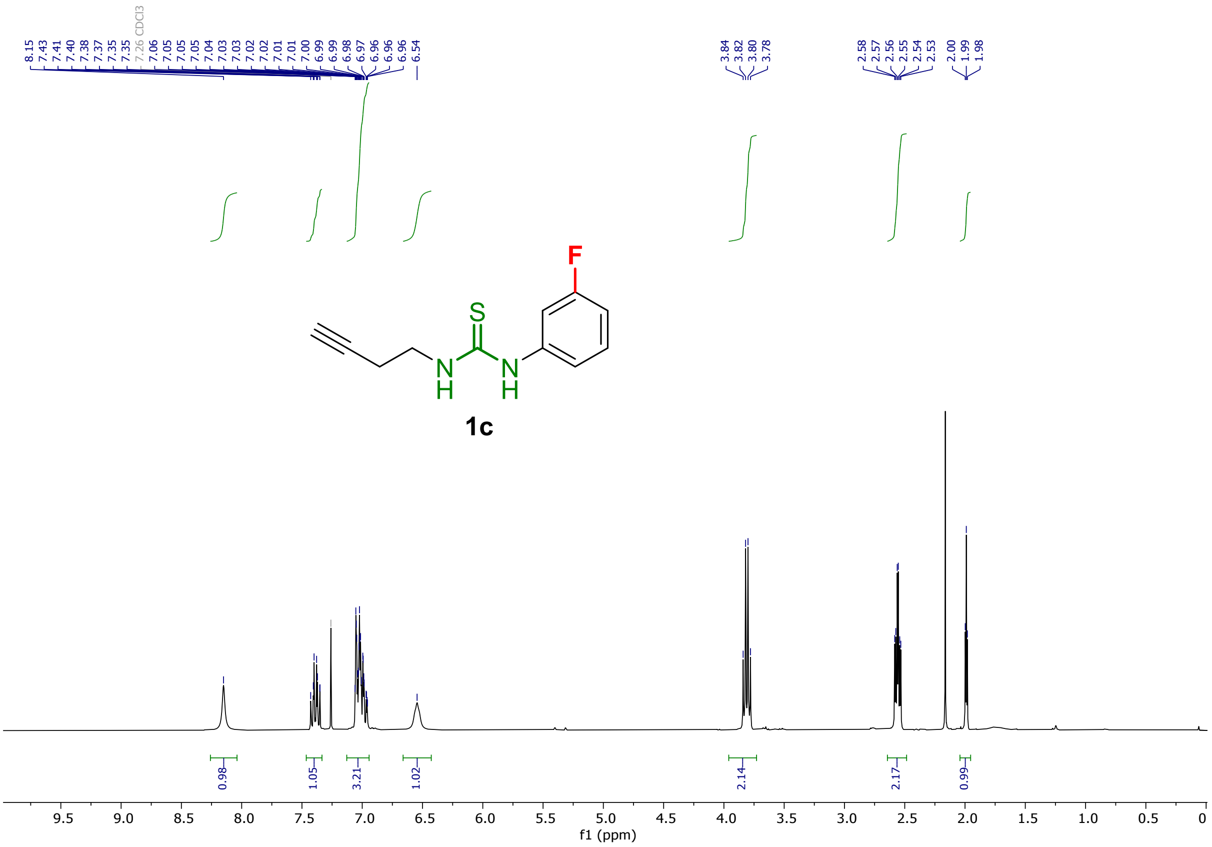

Figure S7. $^{13}C\{^{1}H\}$-APT NMR (75 MHz, $CDCl_3$) spectrum of thiourea **1c**

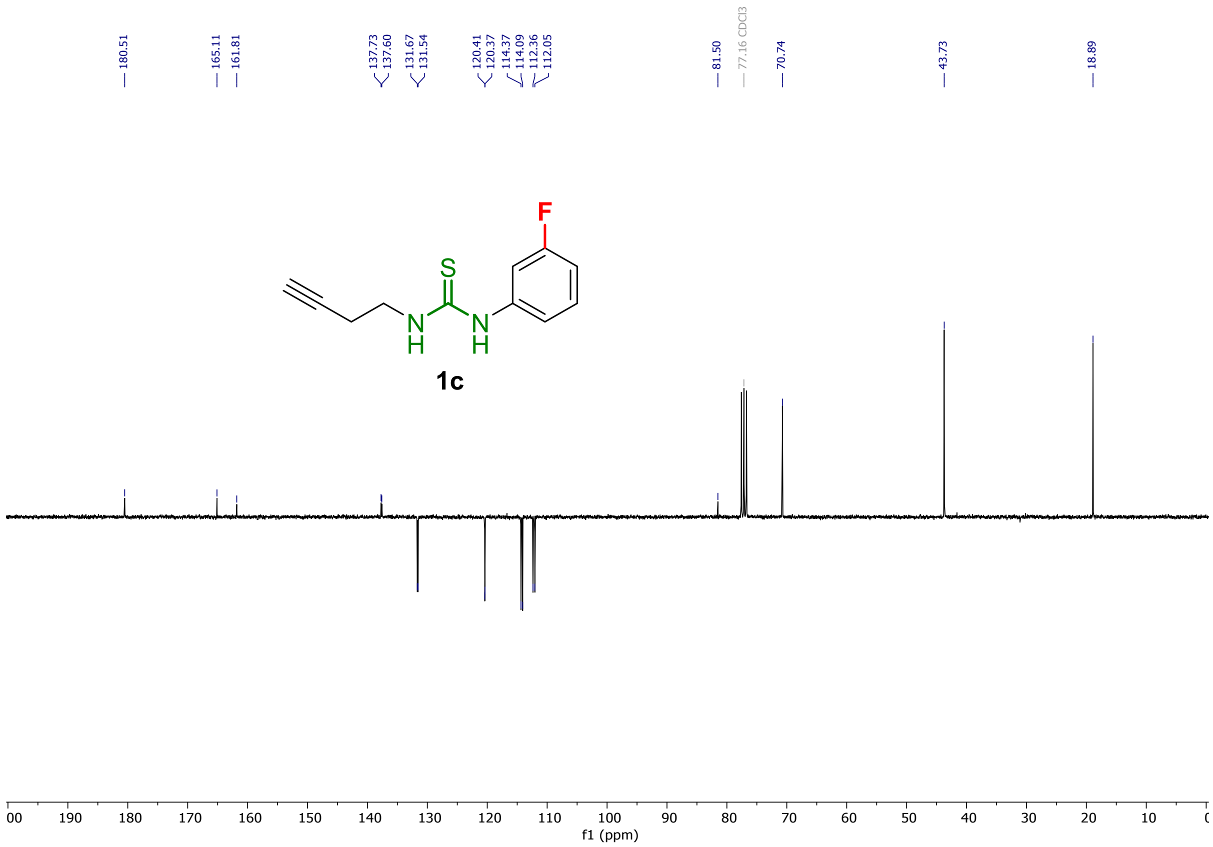

Figure S8. HSQC NMR (300 MHz, 75 MHz, $CDCl_3$) spectrum of thiourea **1c**

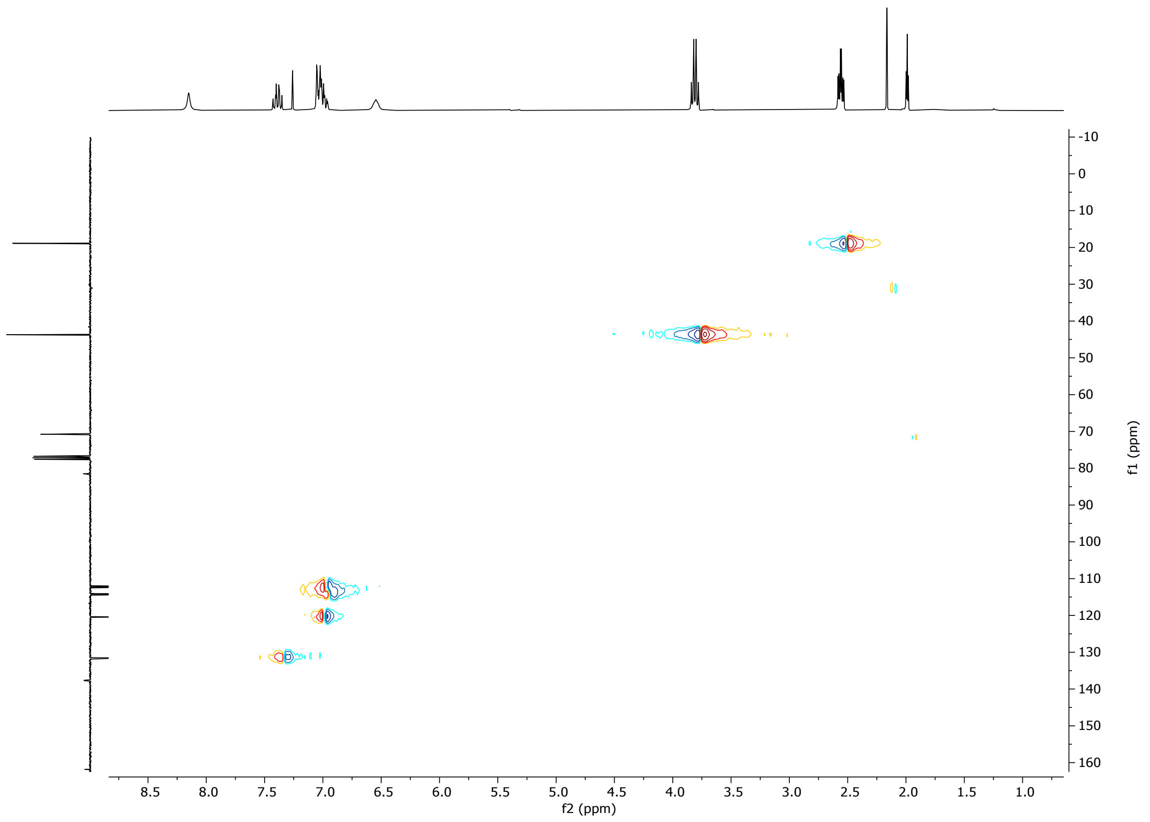

Figure S9. ^{1}H NMR (300 MHz, $CDCl_3$) spectrum of 1-(but-3-yn-1-yl)-3-(4-chlorophenyl)thiourea (**1d**)

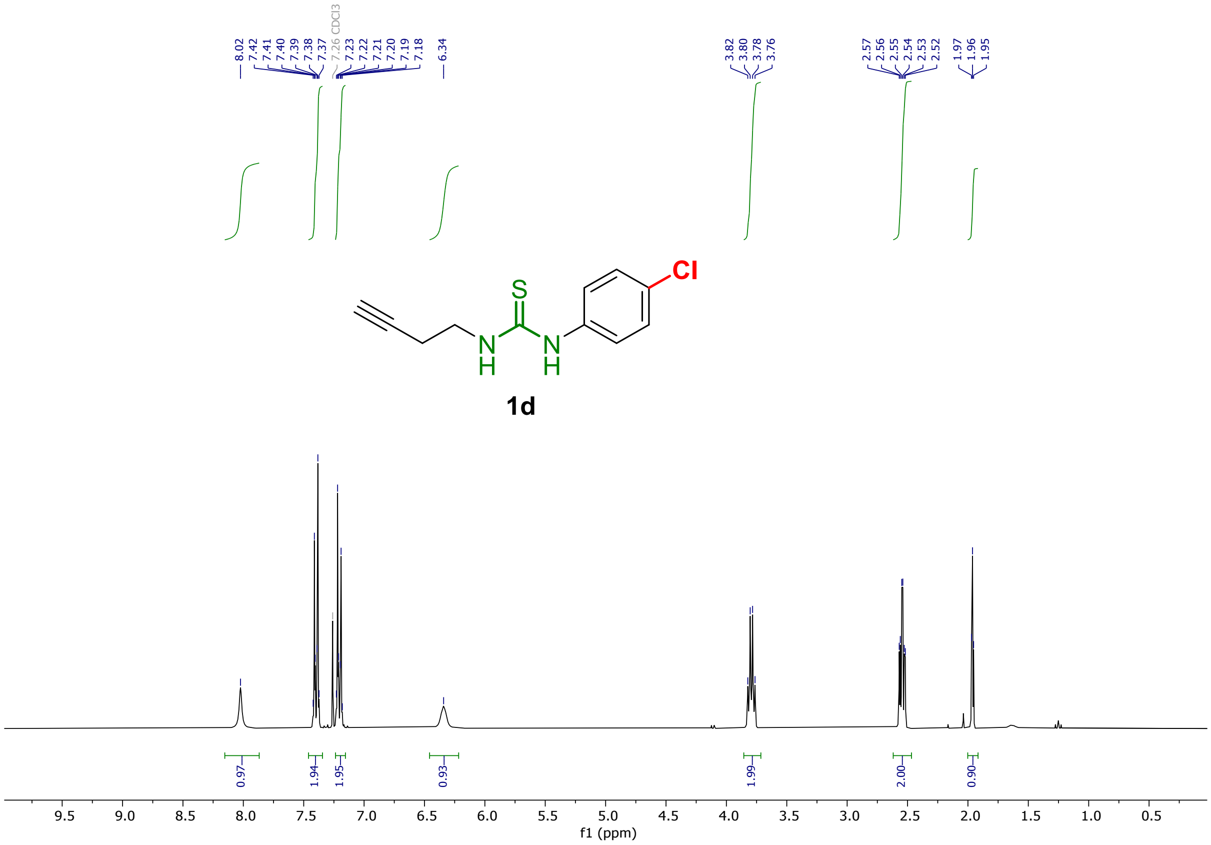

Figure S10. $^{13}C\{^{1}H\}$-APT NMR (101 MHz, $CDCl_3$) spectrum of thiourea **1d**

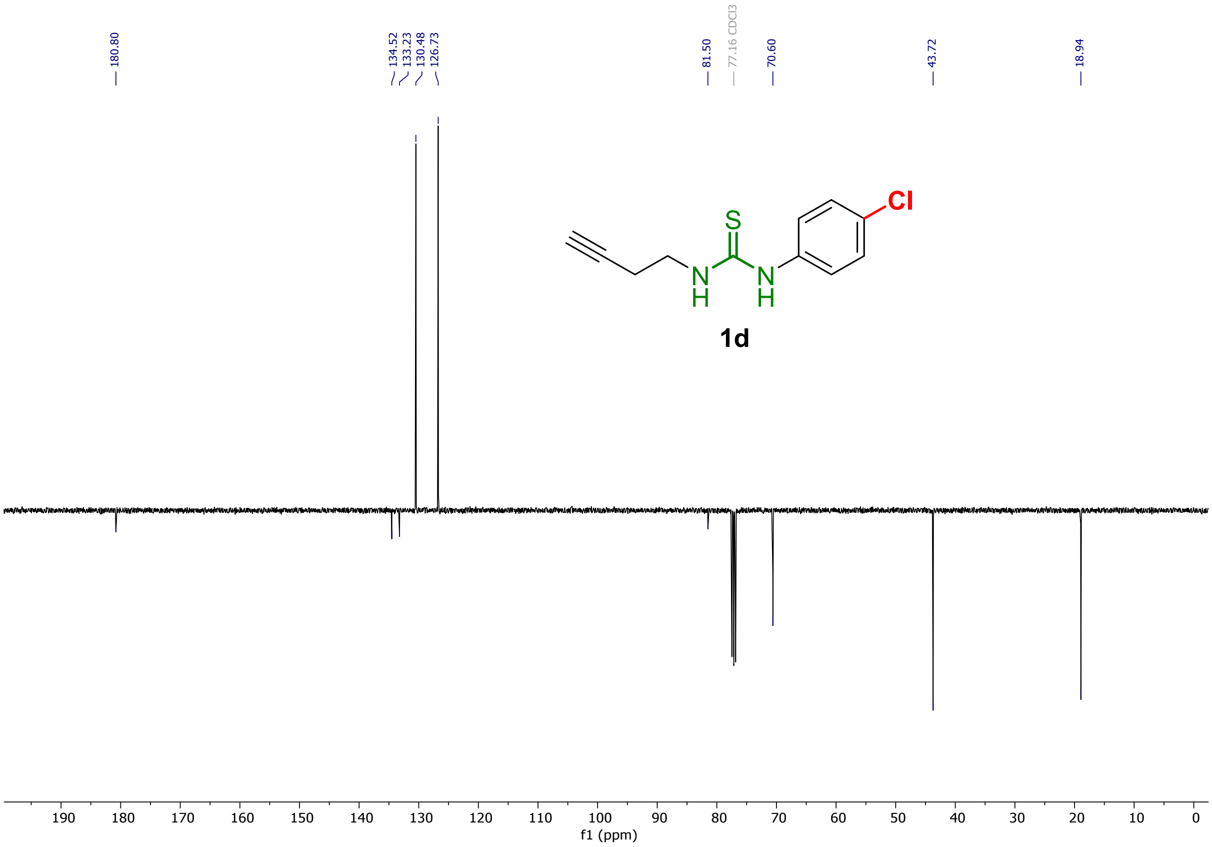

Figure S11. ^{1}H NMR (400 MHz, CD_3COCD_3) spectrum of 1-(but-3-yn-1-yl)-3-(4-bromophenyl)thiourea (**1e**)

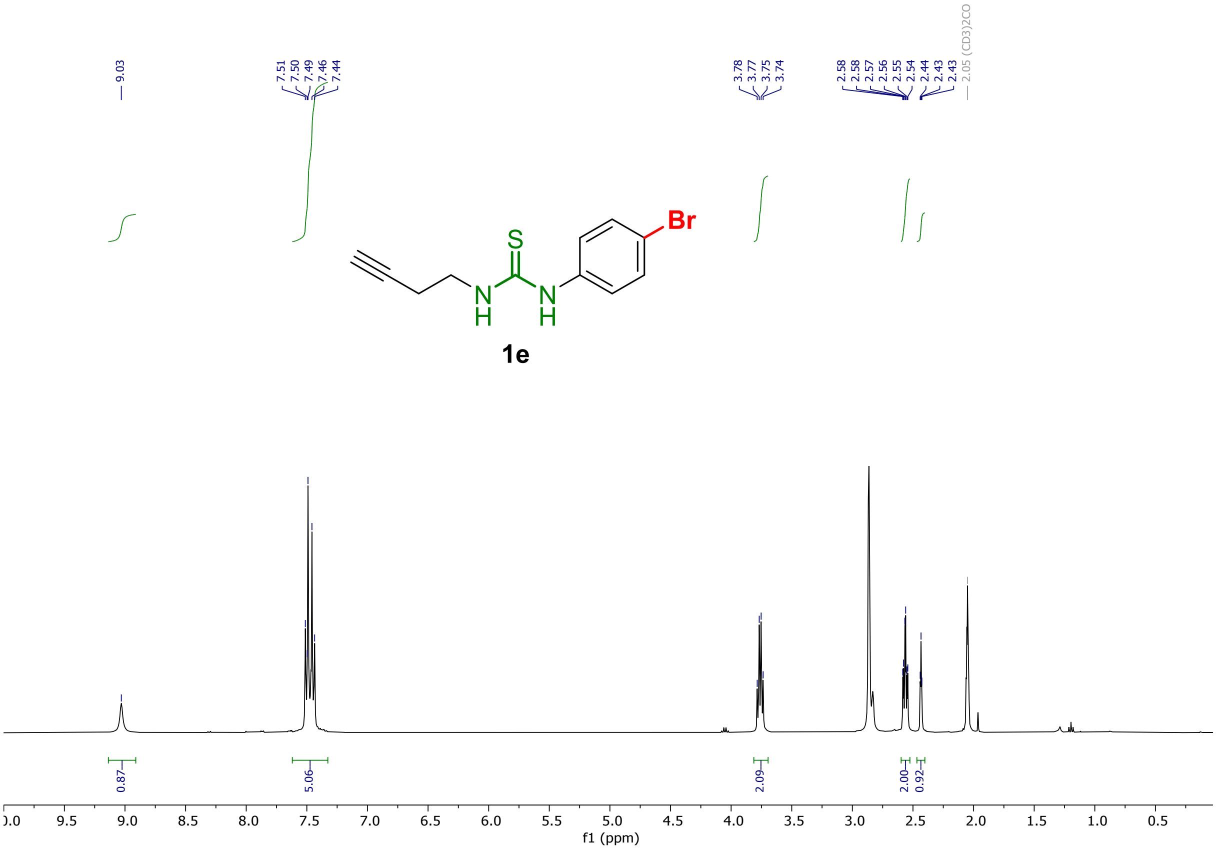

Figure S12. $^{13}C\{^{1}H\}$-APT NMR (101 MHz, CD_3COCD_3) spectrum of thiourea **1e**

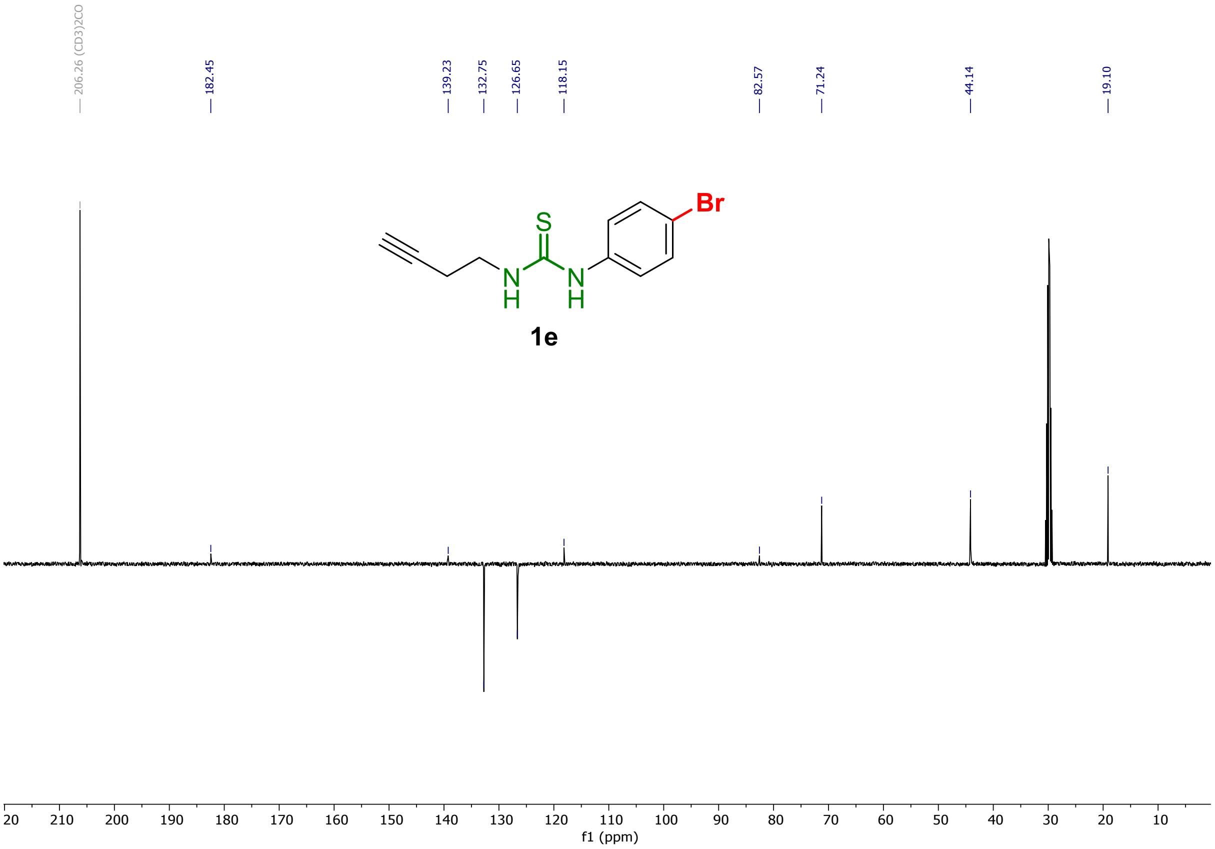

Figure S13. ^{1}H NMR (300 MHz, $CDCl_3$) spectrum of 1-(but-3-yn-1-yl)-3-(4-(trifluoromethyl)phenyl)thiourea (**1f**)

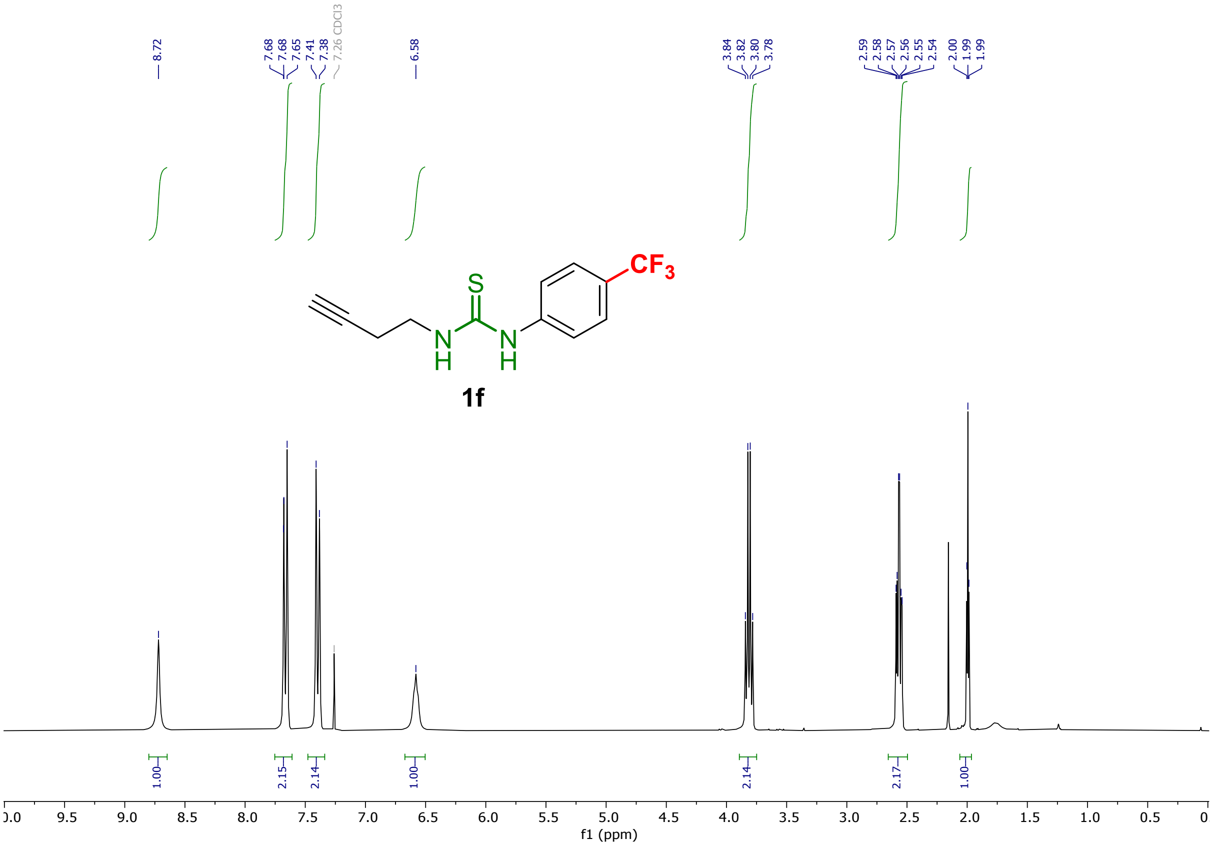

Figure S14. $^{13}C\{^{1}H\}$-APT NMR (75 MHz, $CDCl_3$) spectrum of thiourea **1f**

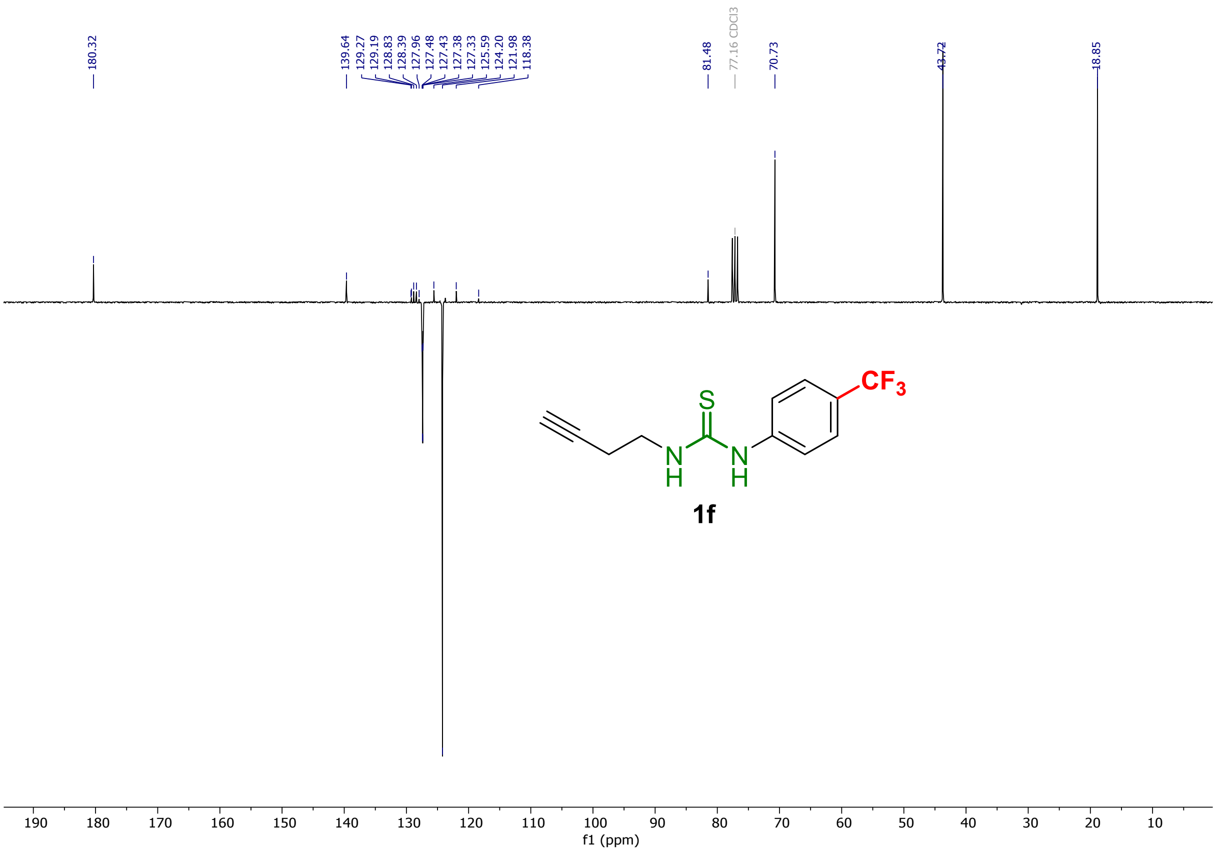

Figure S15. COSY NMR (300 MHz, $CDCl_3$) spectrum of thiourea **1f**

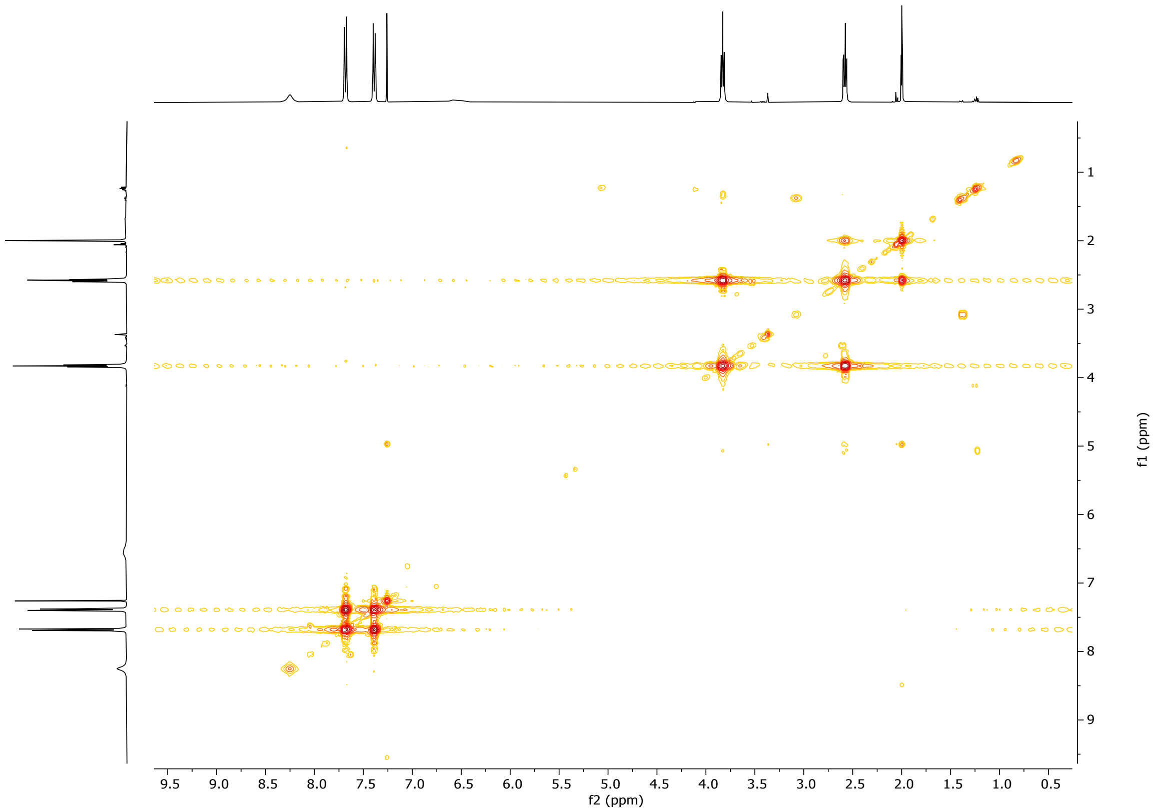

Figure S16. HSQC NMR (300 MHz, 75 MHz, $CDCl_3$) spectrum of thiourea **1f**

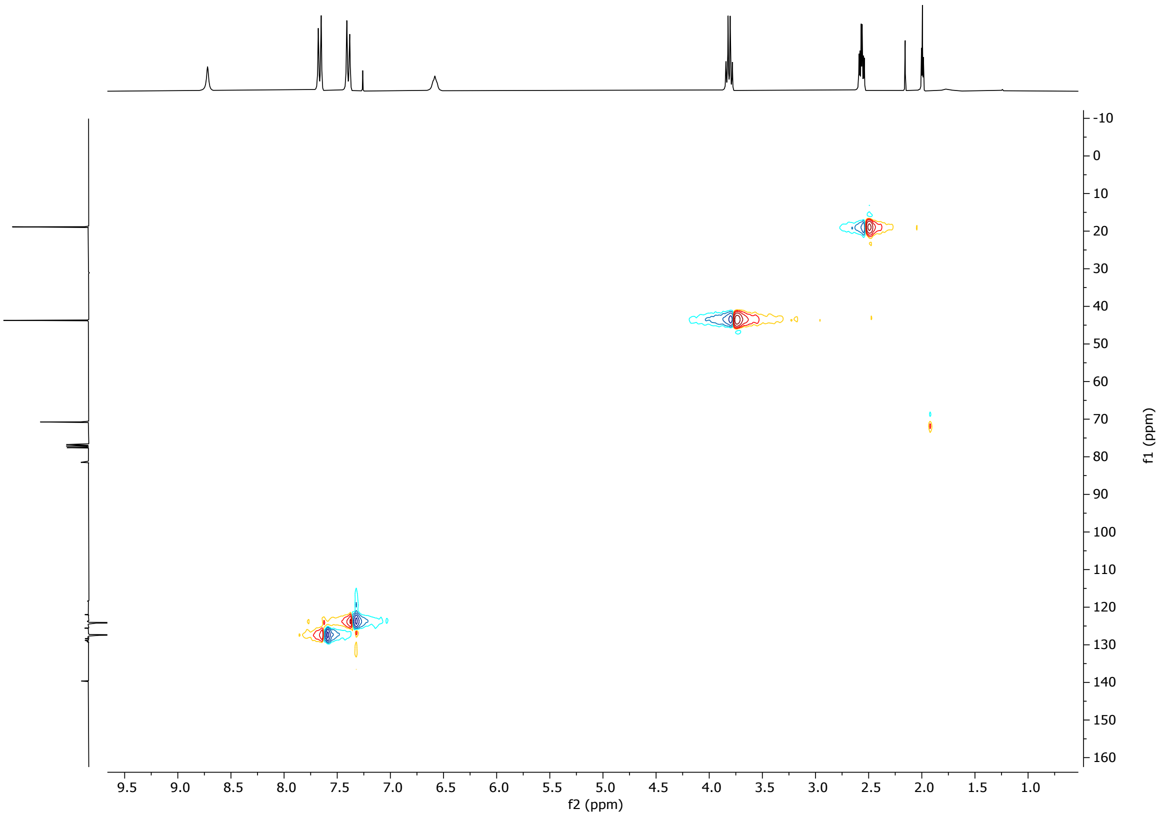

Figure S17. ^{1}H NMR (300 MHz, $CDCl_3$) spectrum of 1-(3,5-bis(trifluoromethyl)phenyl)-3-(but-3-yn-1-yl)thiourea (**1g**)

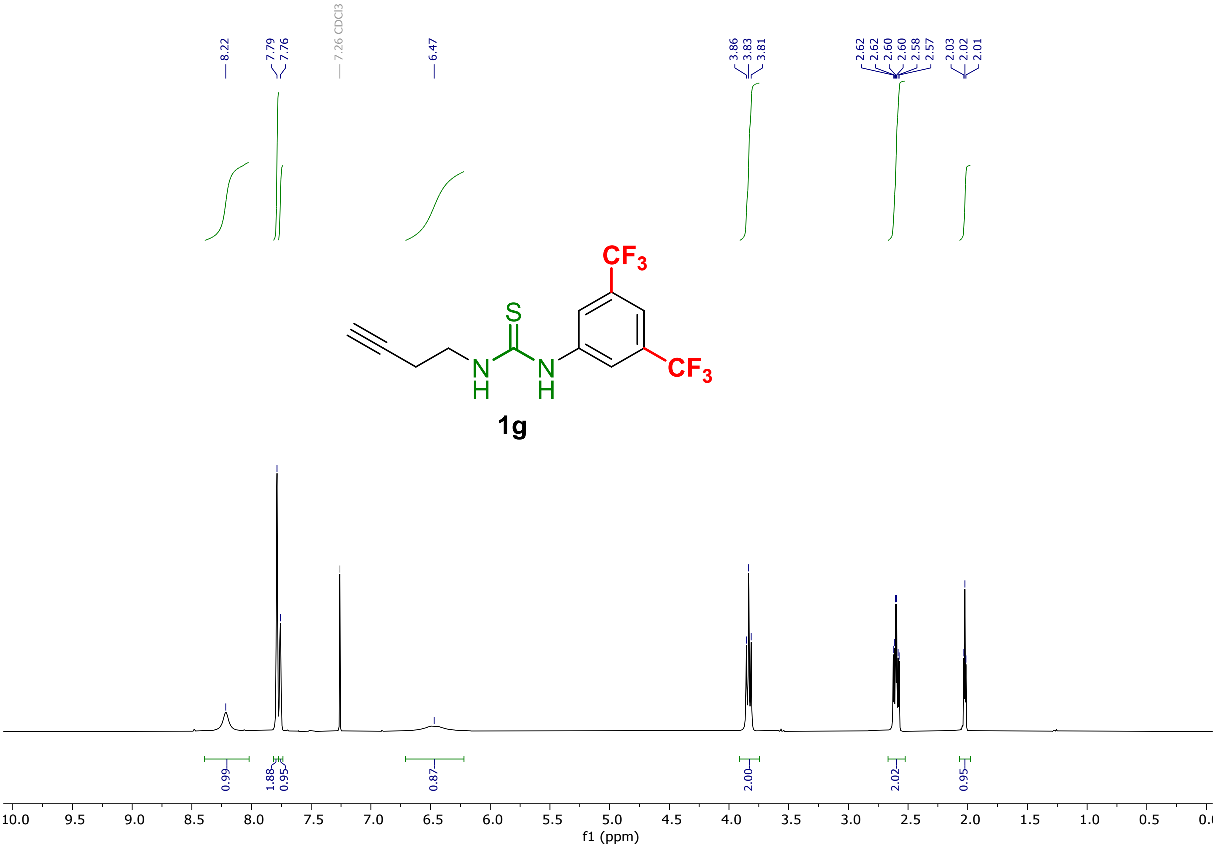

Figure S18. $^{13}C\{^{1}H\}$-APT NMR (75 MHz, $CDCl_3$) spectrum of thiourea **1g**

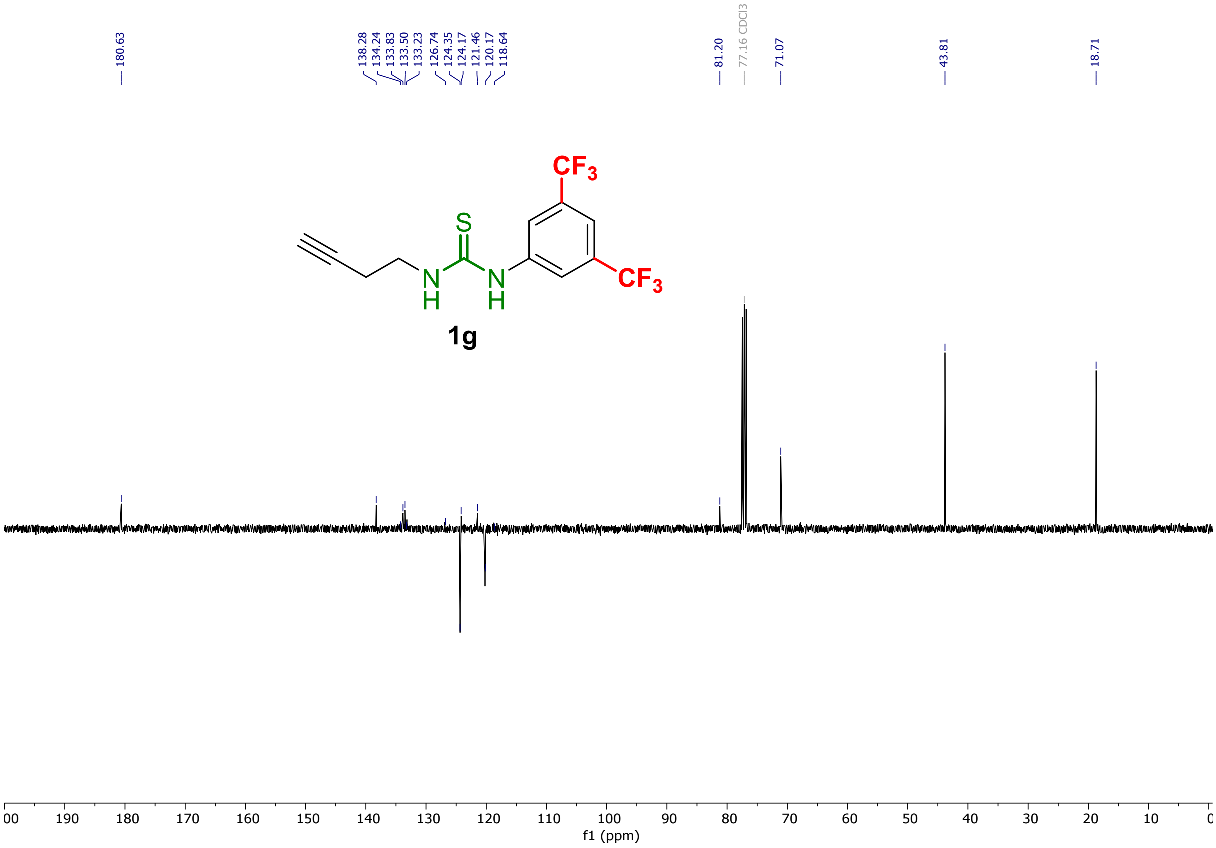

Figure S19. ^{1}H NMR (300 MHz, CD_3COCD_3) spectrum of 1-(but-3-yn-1-yl)-3-(4-nitrophenyl)thiourea (**1h**)

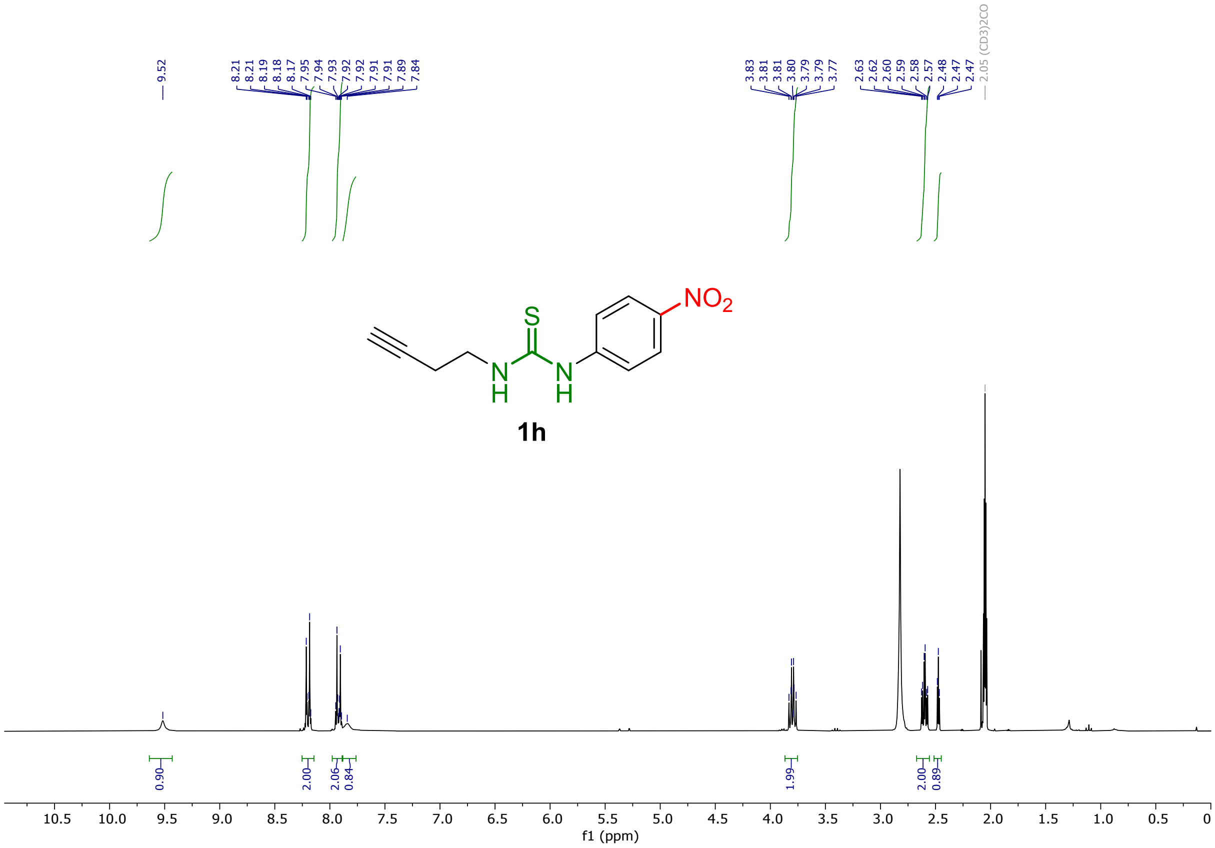

Figure S20. $^{13}C\{^1H\}$-APT NMR (75 MHz, CD_3COCD_3) spectrum of thiourea **1h**

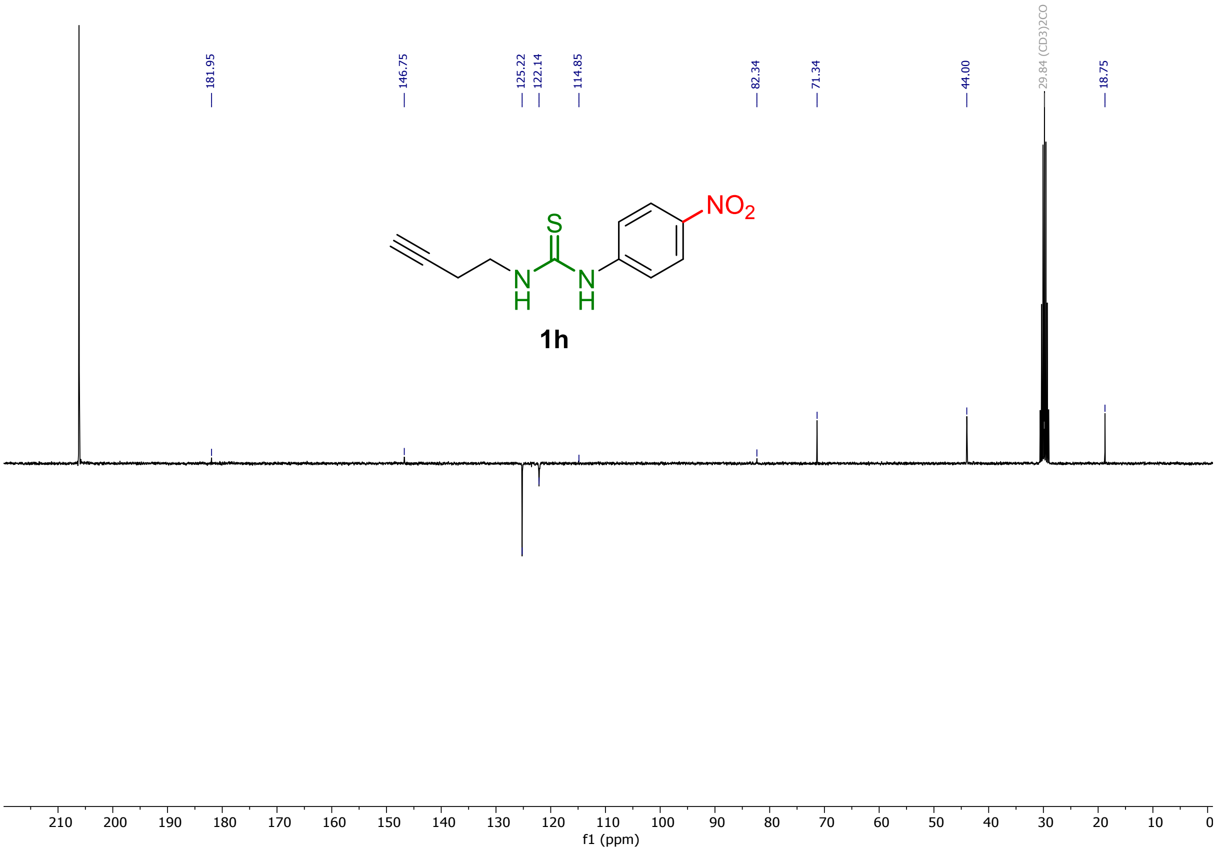

Figure S21. COSY NMR (300 MHz, CD_3COCD_3) spectrum of thiourea **1h**

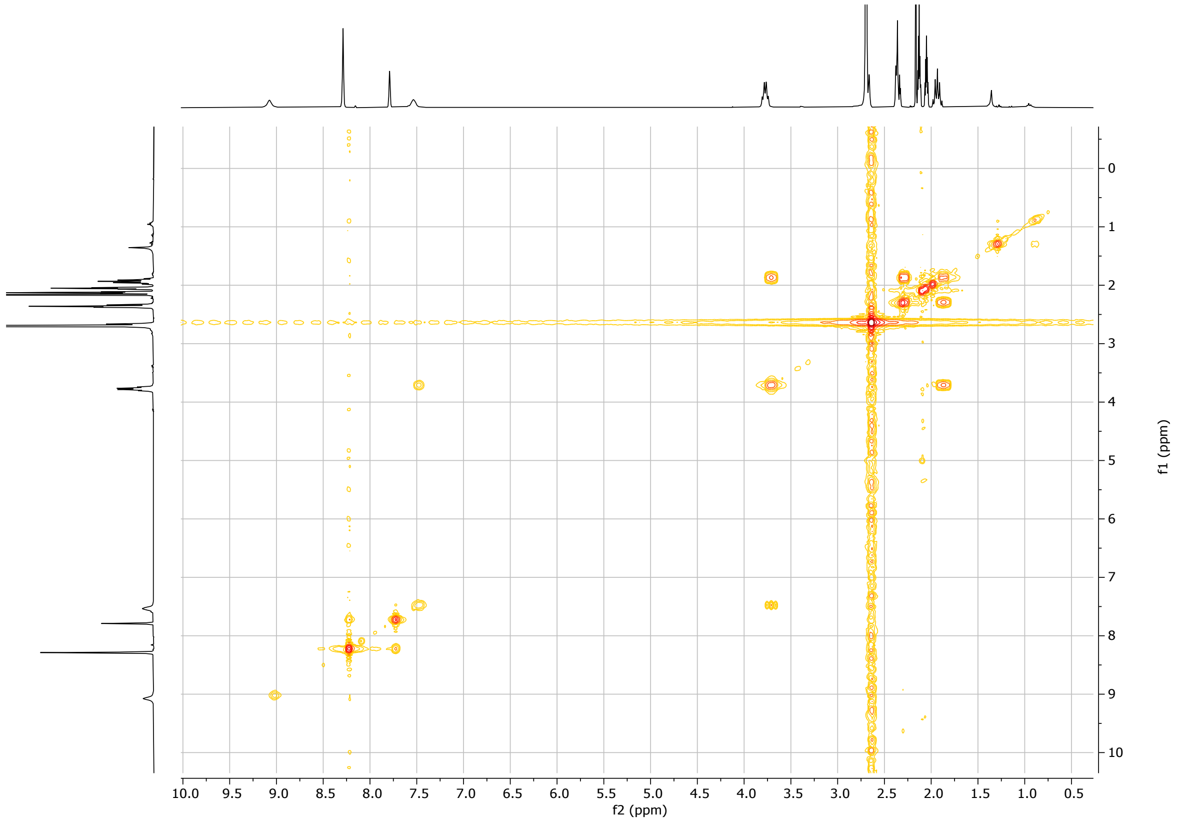

Figure S22. ^{1}H NMR (300 MHz, CD_3COCD_3) spectrum of 1-(but-3-yn-1-yl)-3-(4-cyanophenyl)thiourea (**1i**)

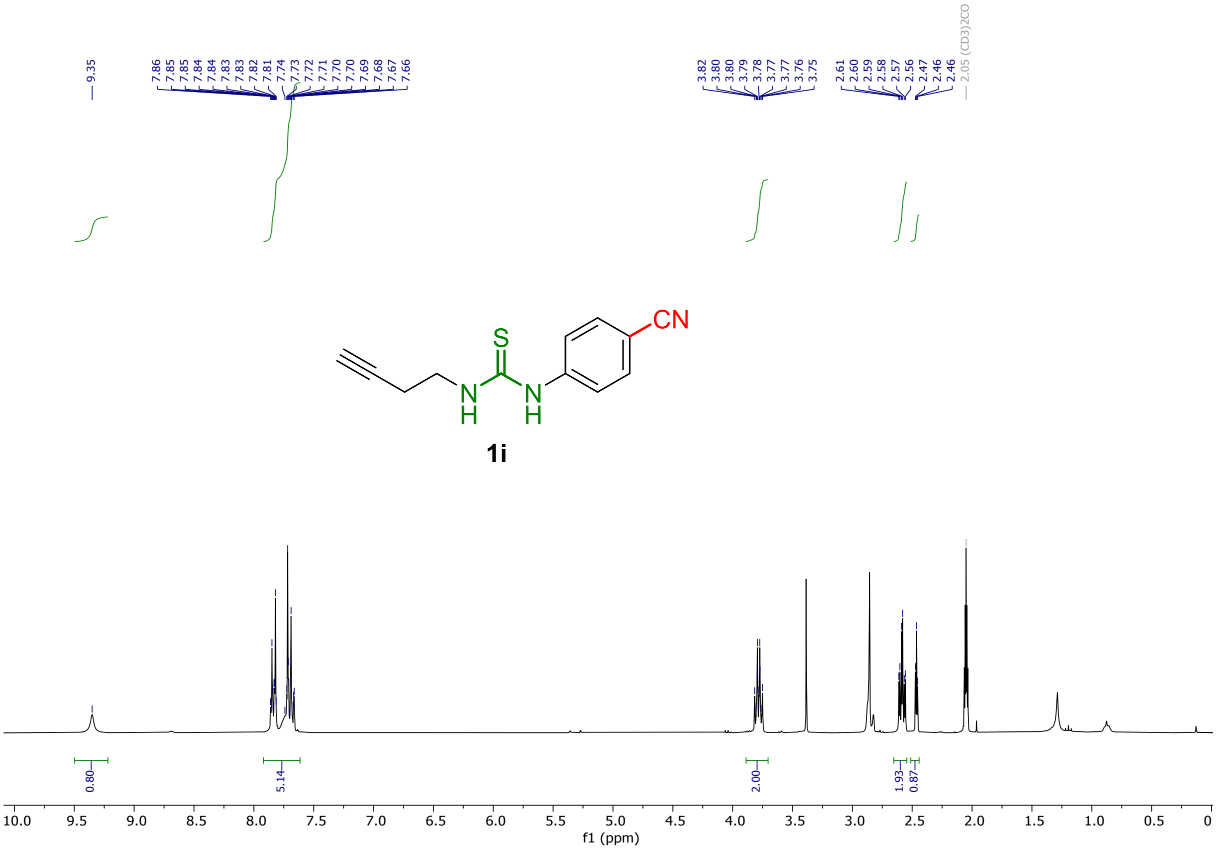

Figure S23. $^{13}C\{^{1}H\}$-APT NMR (75 MHz, CD_3COCD_3) spectrum of thiourea **1i**

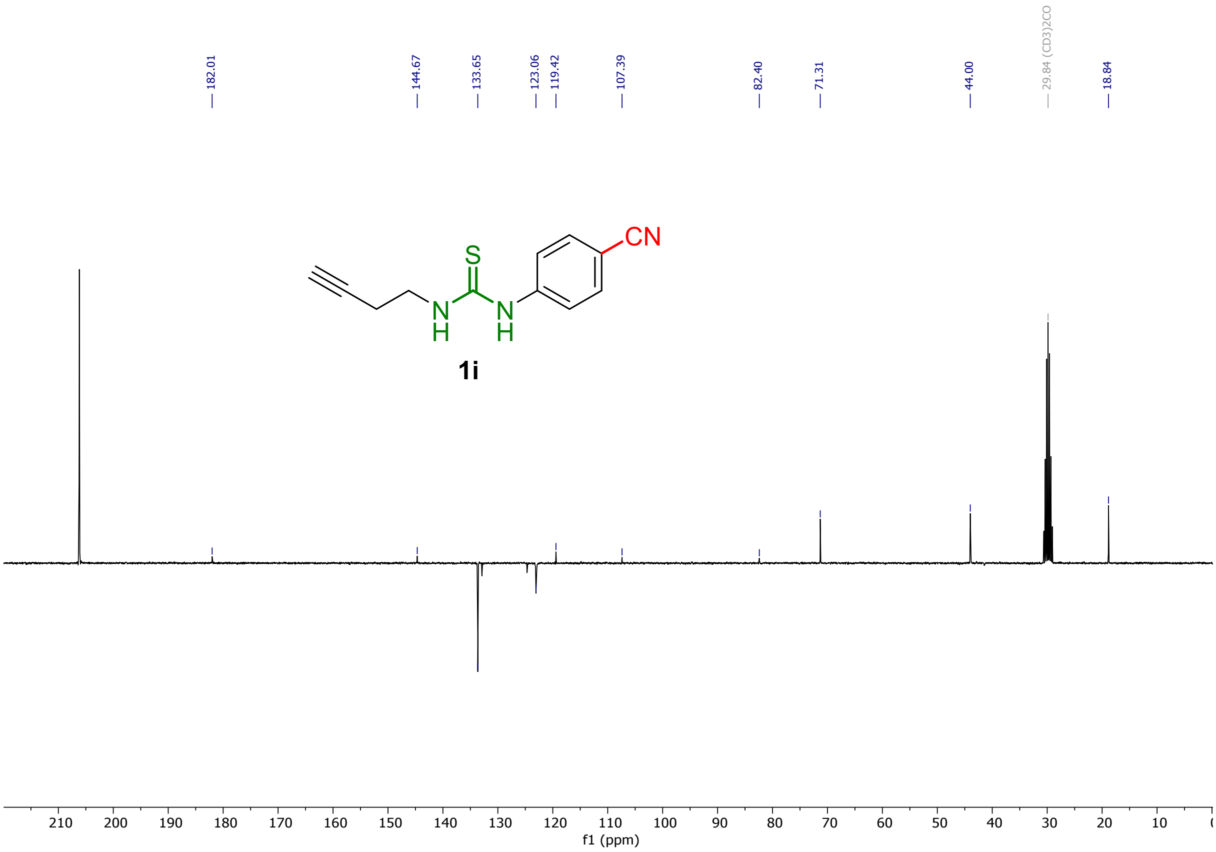

Figure S24. ^{1}H NMR (300 MHz, $CDCl_3$) spectrum of 1-(but-3-yn-1-yl)-3-(pyridin-3-yl)thiourea (**1j**)

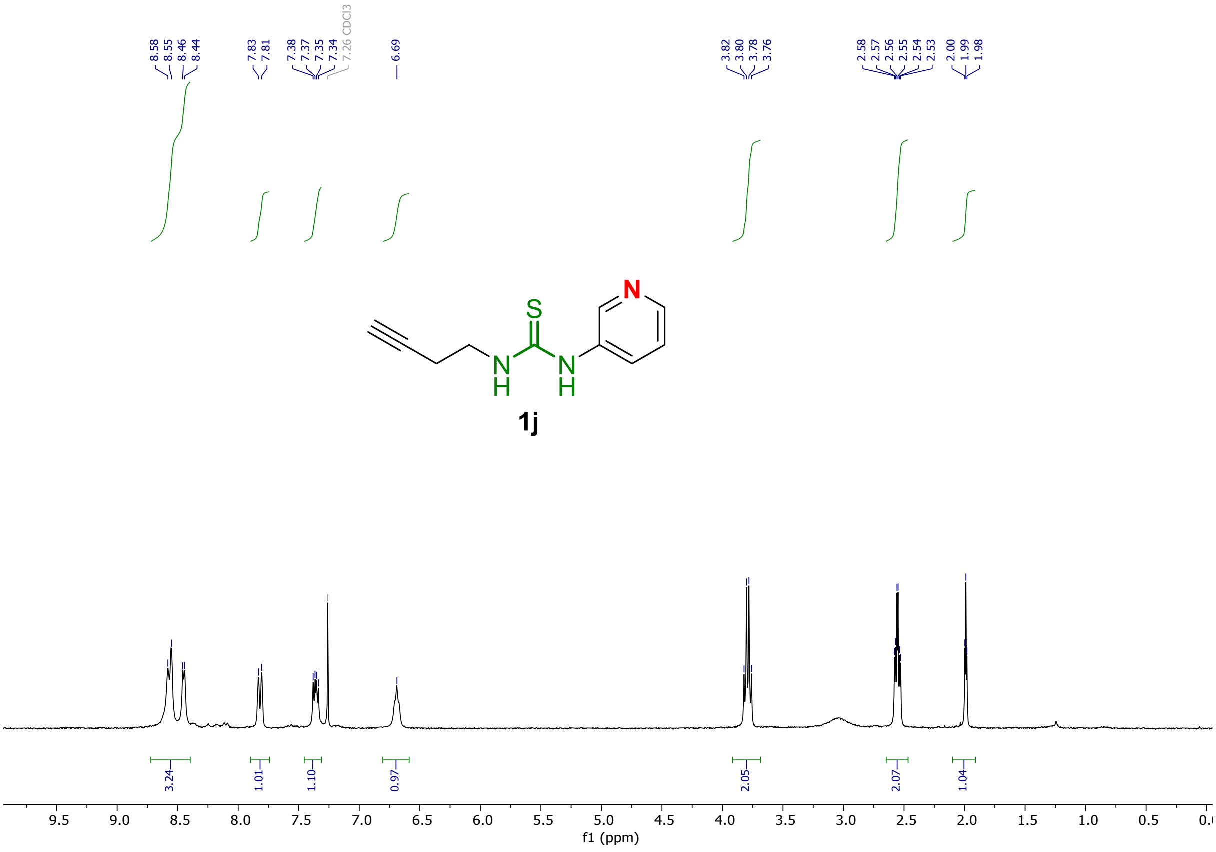

Figure S25. $^{13}C\{^{1}H\}$-APT NMR (75 MHz, $CDCl_3$) spectrum of thiourea **1j**

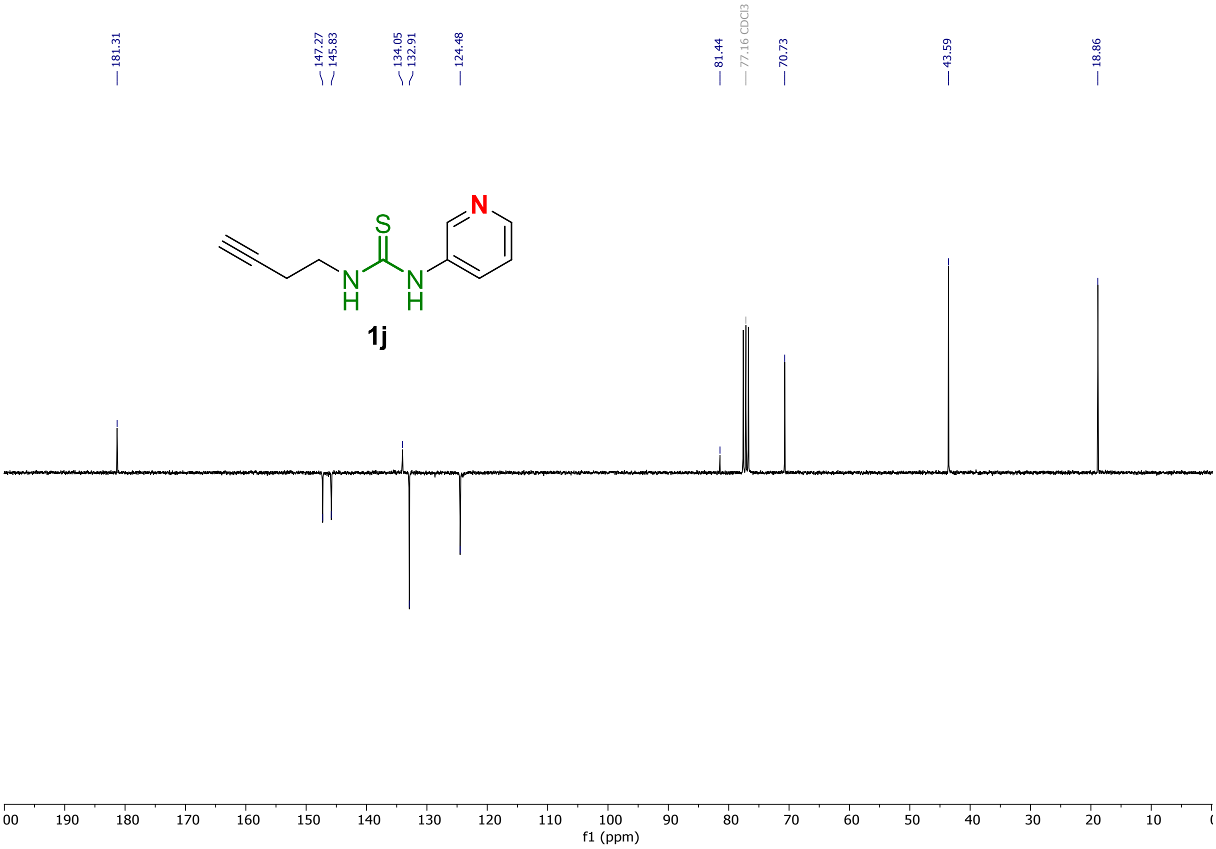

Figure S26. COSY NMR (300 MHz, $CDCl_3$) spectrum of thiourea **1j**

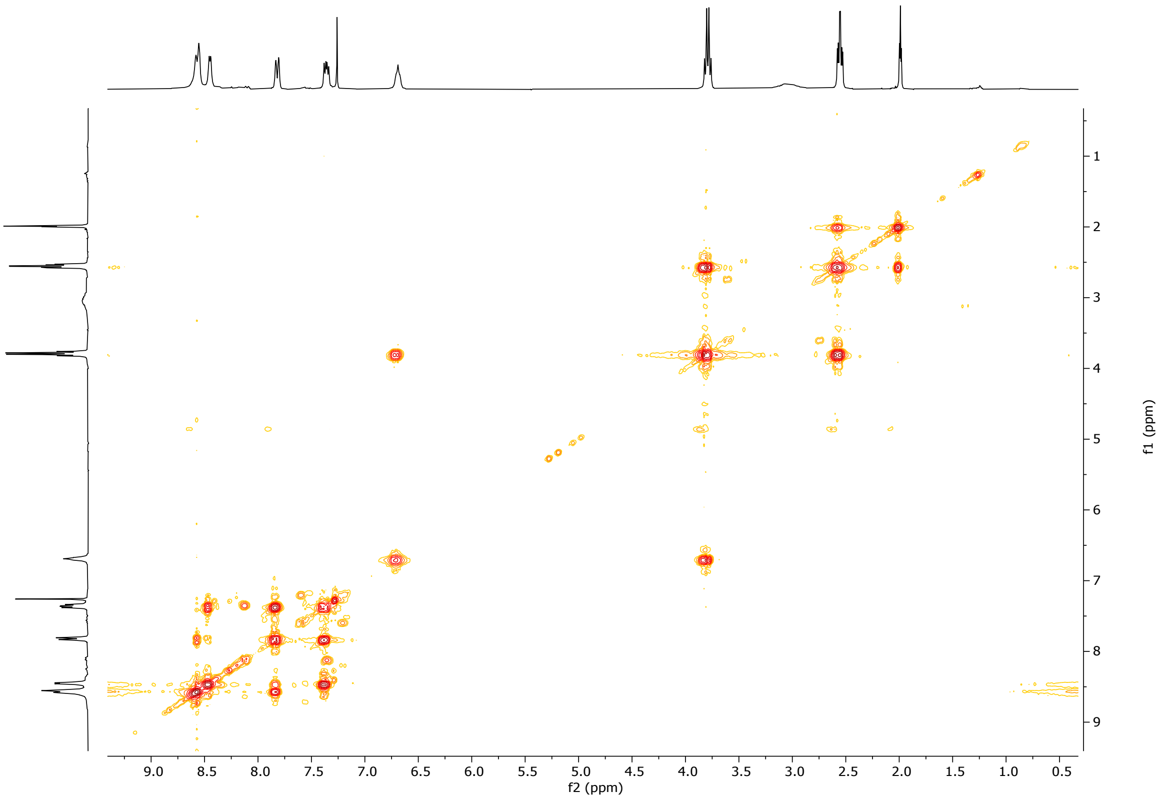

Figure S27. ^{1}H NMR (300 MHz, CD_3CN) spectrum of 1-(but-3-yn-1-yl)-3-(4-methylphenyl)thiourea (**1k**)

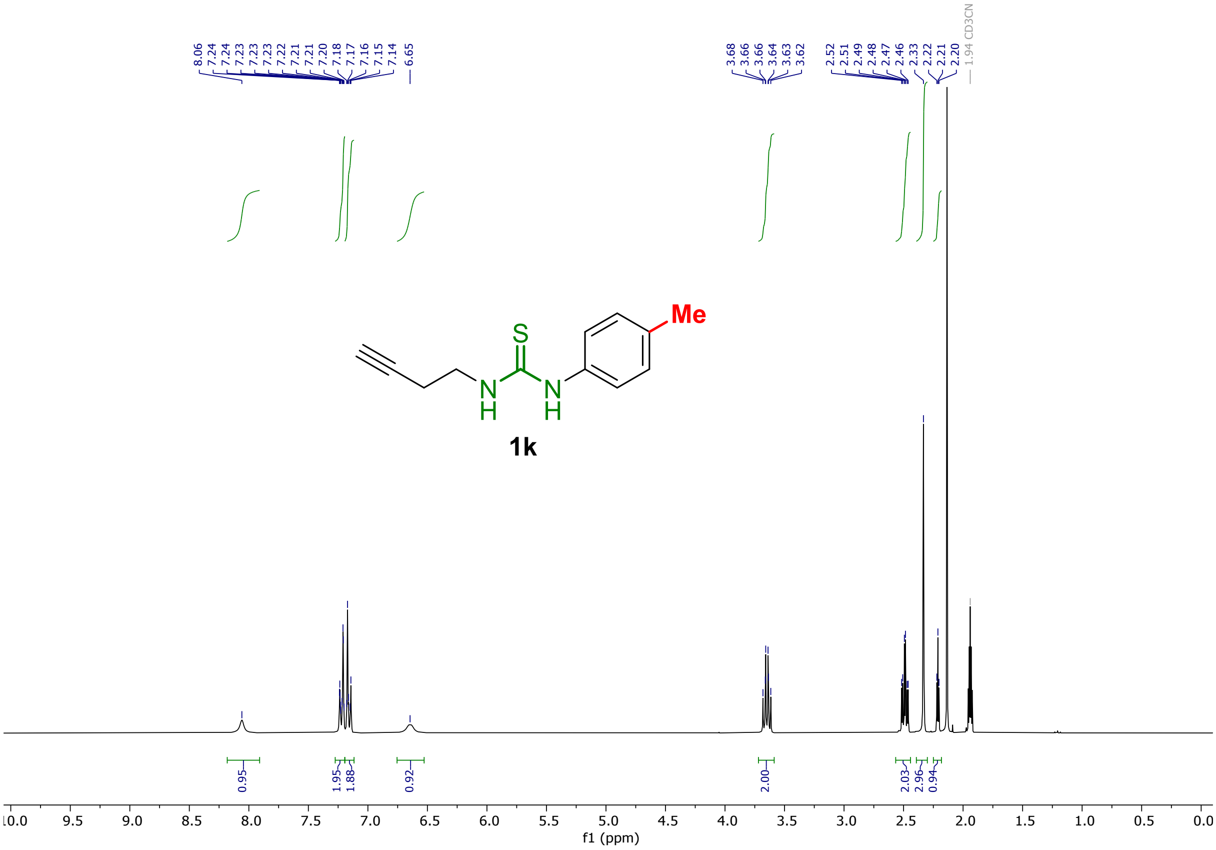

Figure S28. $^{13}C\{^1H\}$-APT NMR (75 MHz, CD_3CN) spectrum of thiourea **1k**

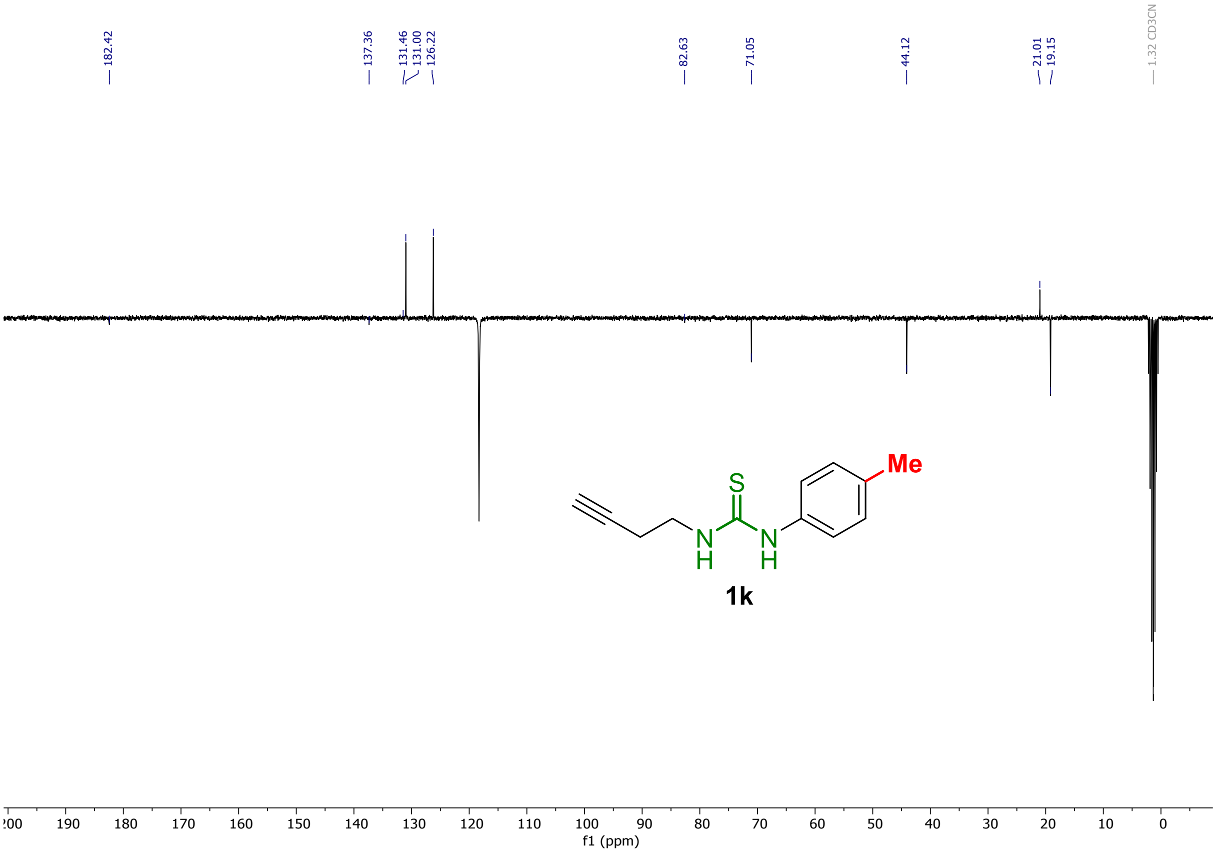

Figure S29. ^{1}H NMR (300 MHz, $CDCl_3$) spectrum of 1-(but-3-yn-1-yl)-3-(4-methoxyphenyl)thiourea (**1l**)

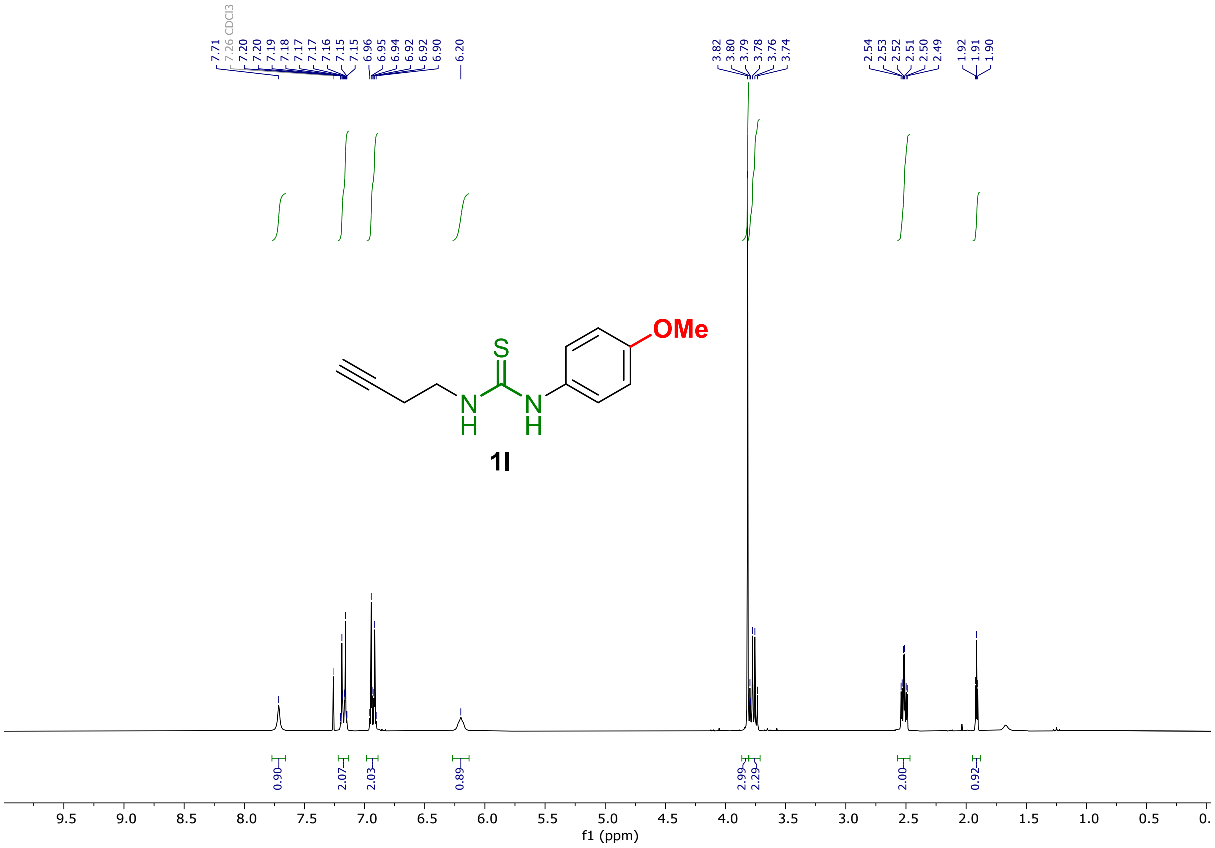

Figure S30. $^{13}C\{^{1}H\}$-APT NMR (75 MHz, $CDCl_3$) spectrum of thiourea **1l**

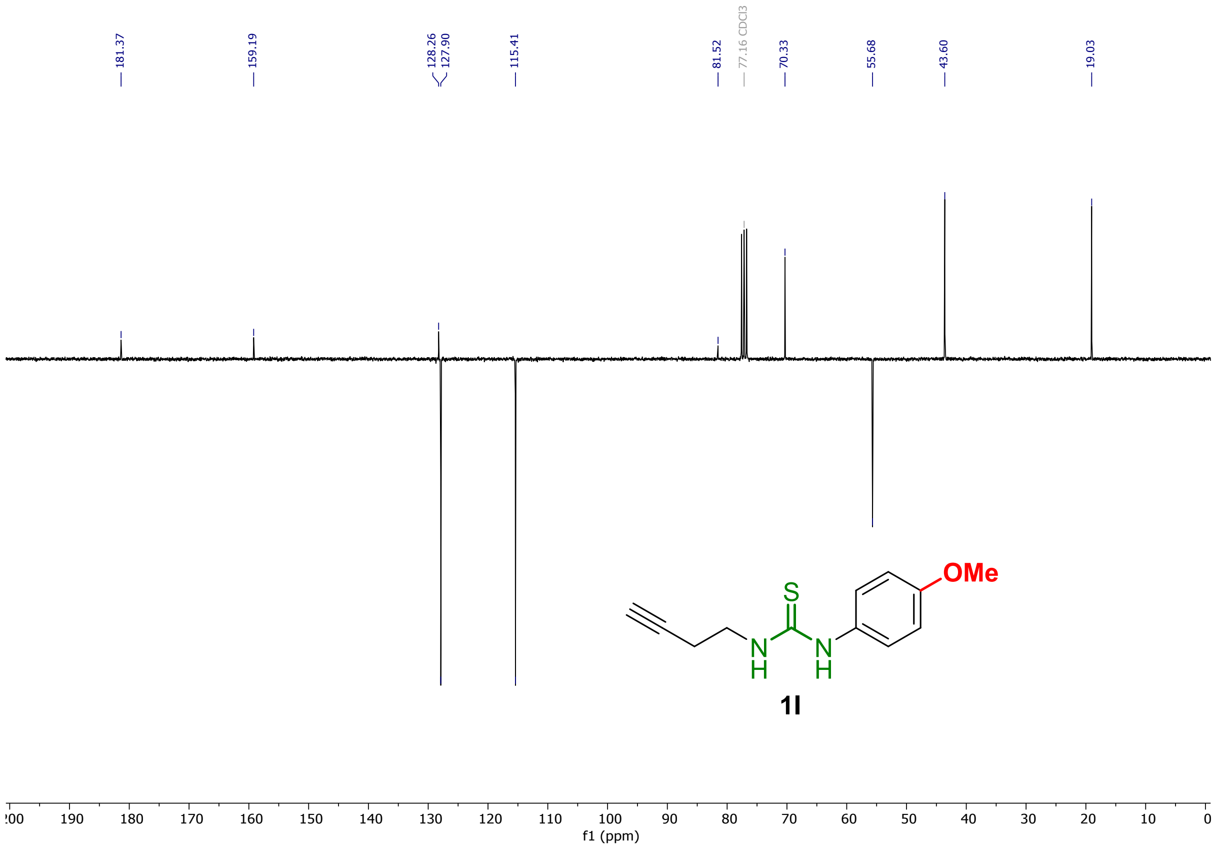

Figure S31. COSY NMR (300 MHz, $CDCl_3$) spectrum of thiourea **1l**

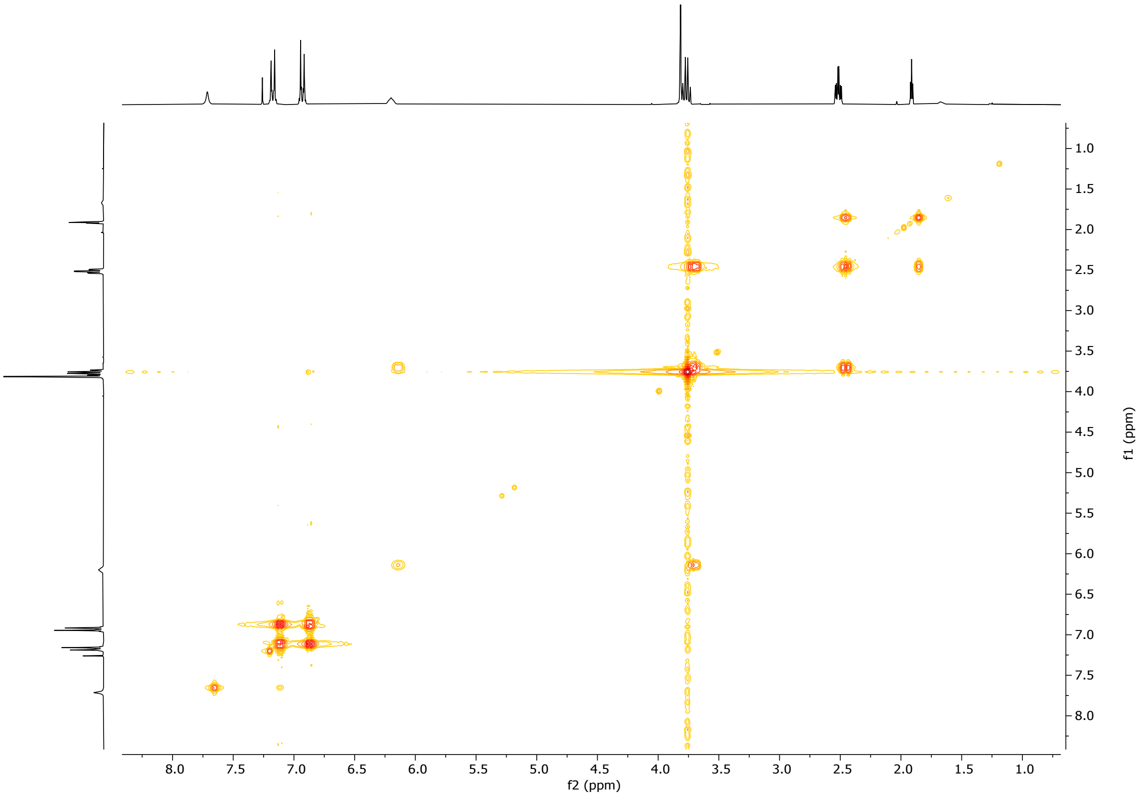

Figure S32. HSQC (300 MHz, 75 MHz, $CDCl_3$) NMR spectrum of thiourea **1l**

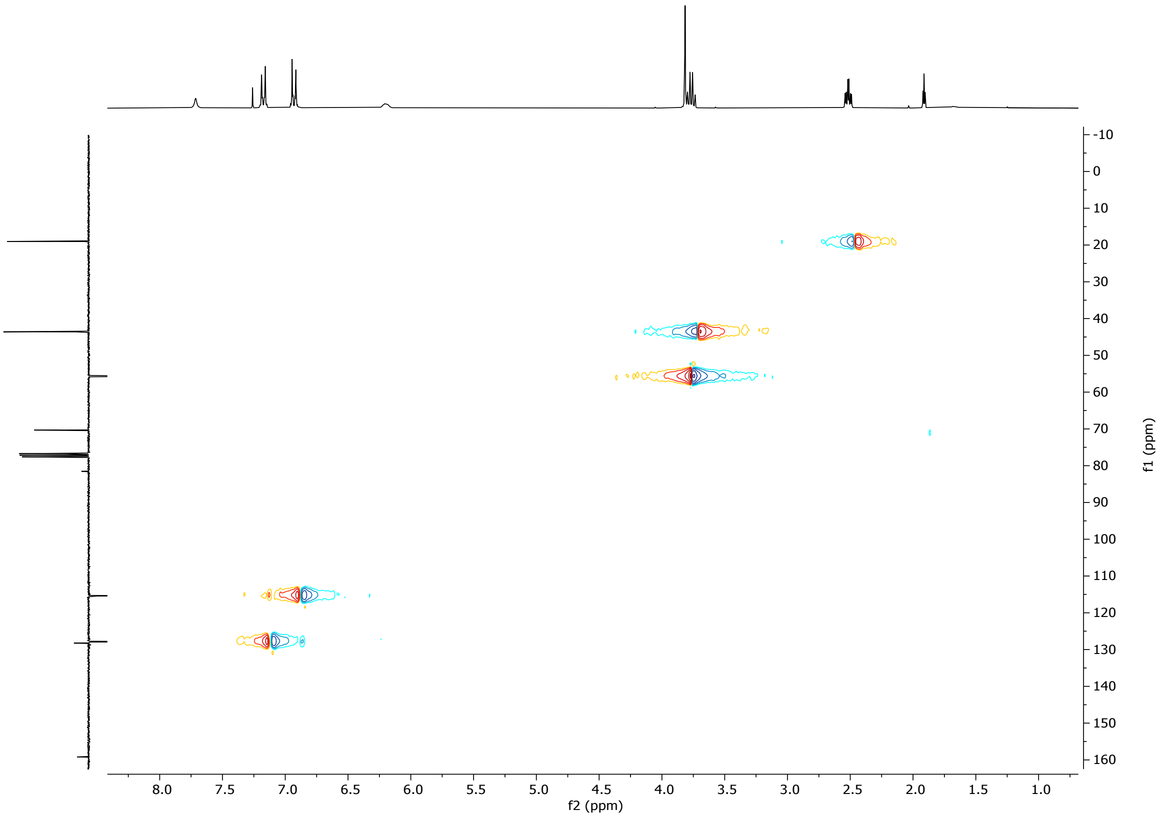

Figure S33. ^{1}H NMR (300 MHz, DMSO-d_6) spectrum of 1-(but-3-yn-1-yl)-3-(naphthalen-1-yl)thiourea (**1m**)

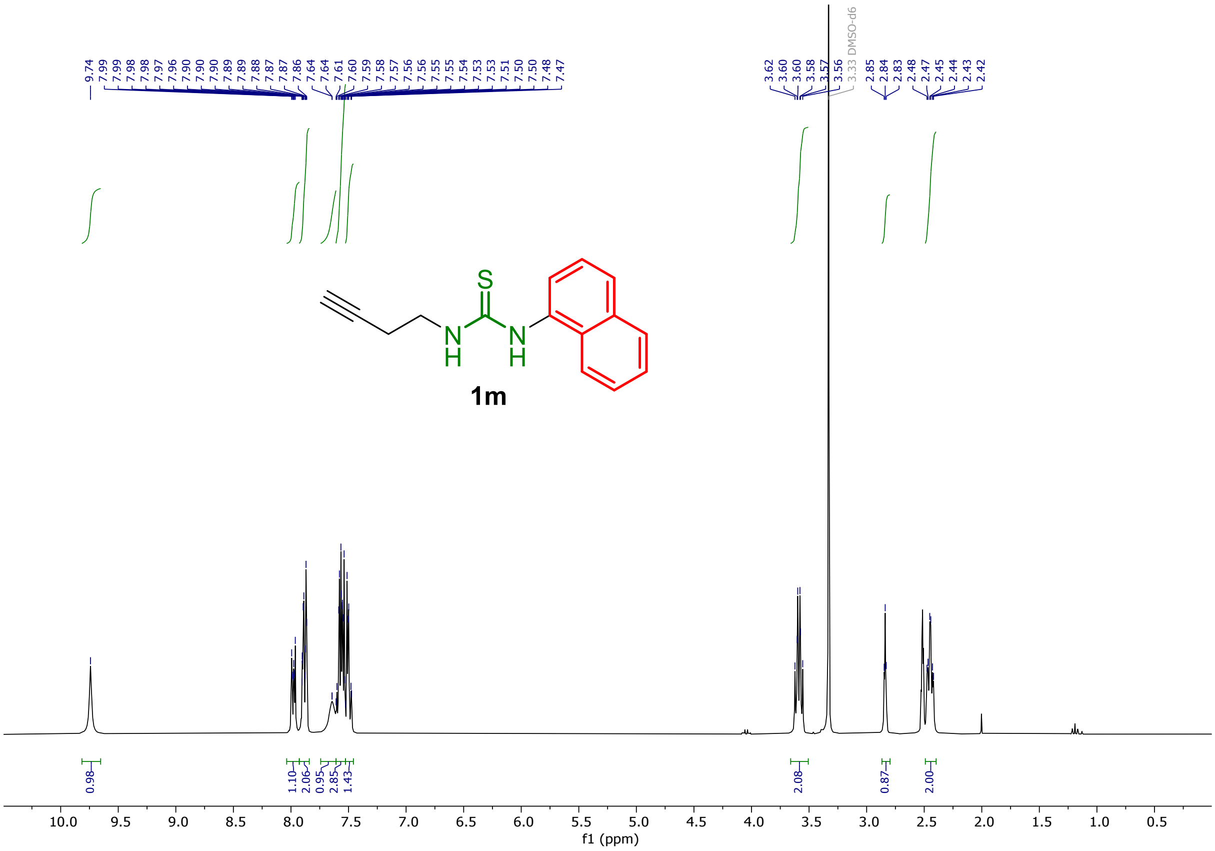

Figure S34. ^{13}C{^{1}H}-APT NMR (75 MHz, DMSO-d_6) spectrum of thiourea **1m**

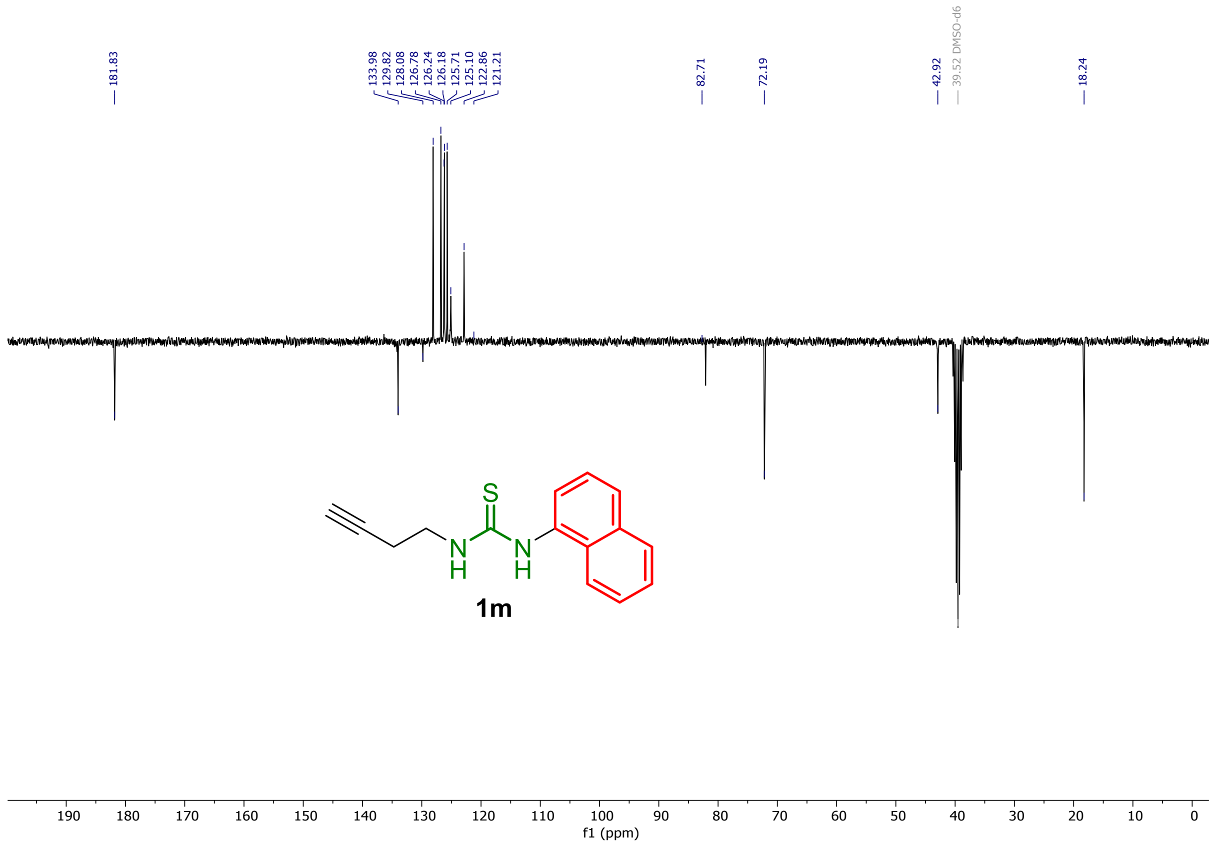

Figure S35. ^{1}H NMR (300 MHz, CD_3COCD_3) spectrum of 1-benzyl-3-(but-3-yn-1-yl)thiourea (**1n**)

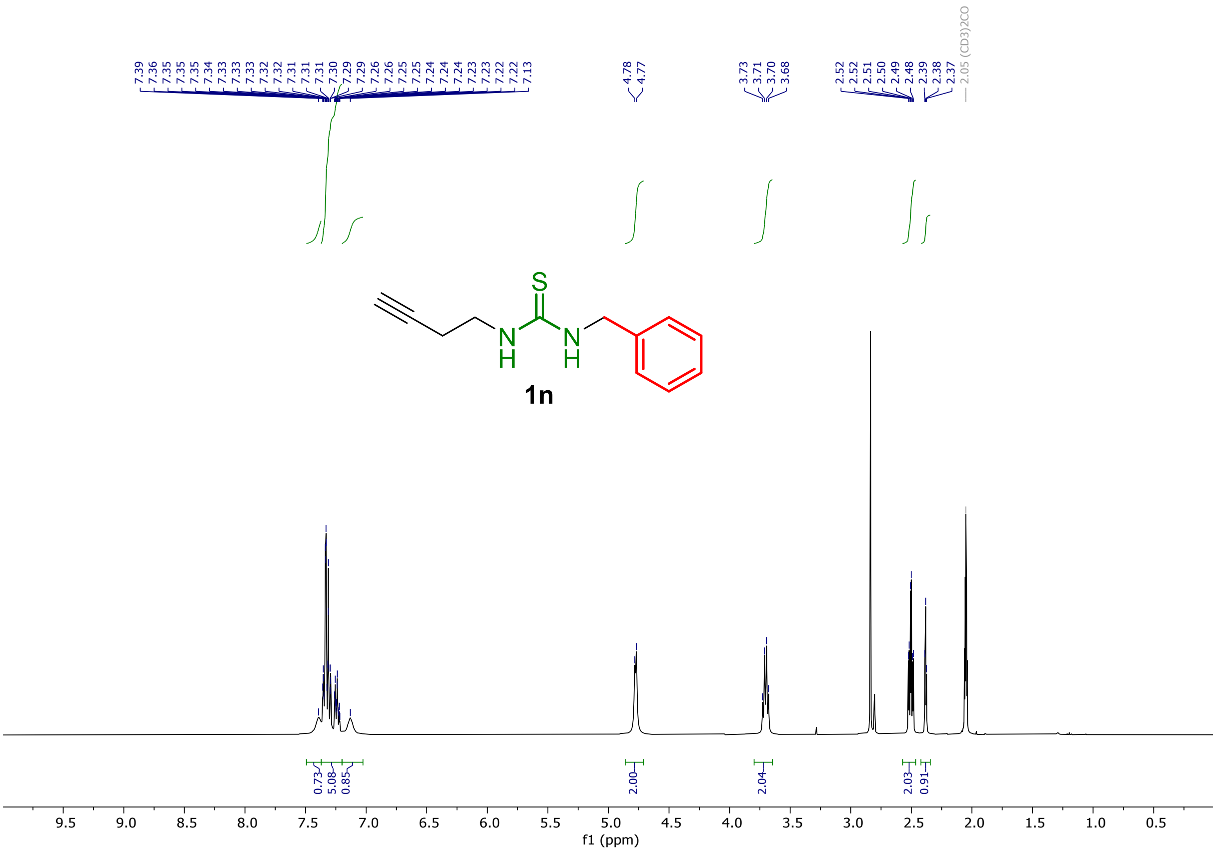

Figure S36. $^{13}C\{^{1}H\}$-APT NMR (75 MHz, $CDCl_3$) spectrum of 1-benzyl-3-(but-3-yn-1-yl)thiourea (**1n**)

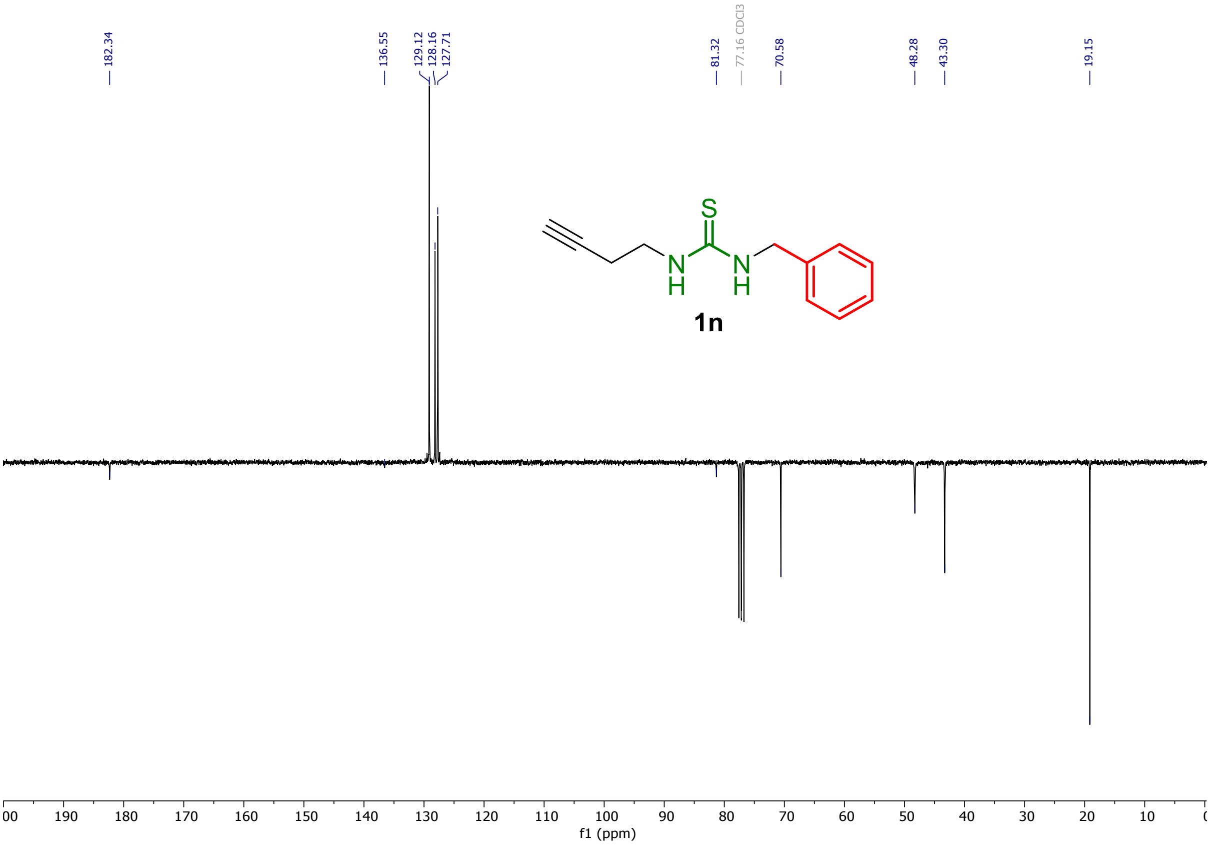

Figure S37. HSQC NMR (300 MHz, 75 MHz, $CDCl_3$) spectrum of thiourea **1n**

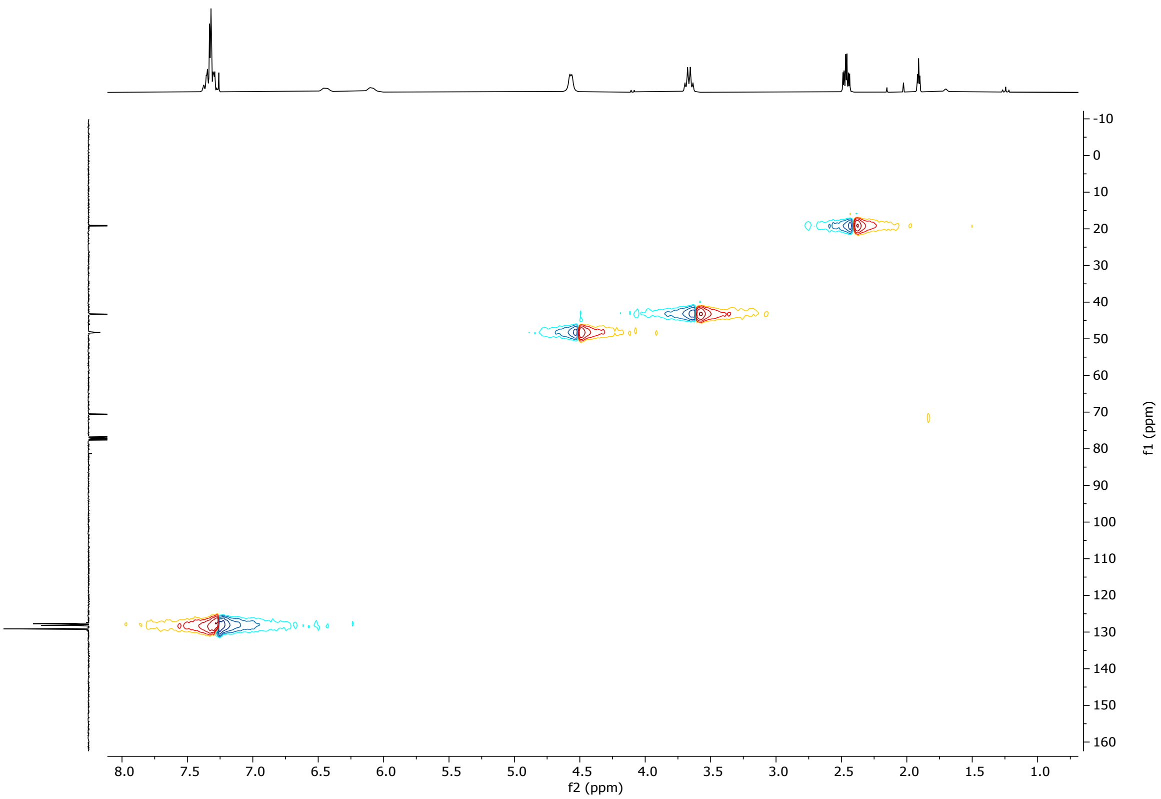

Figure S38. ^1H NMR (300 MHz, CD_3COCD_3) spectrum of 1-(but-3-yn-1-yl)-3-phenethylthiourea (**1o**)

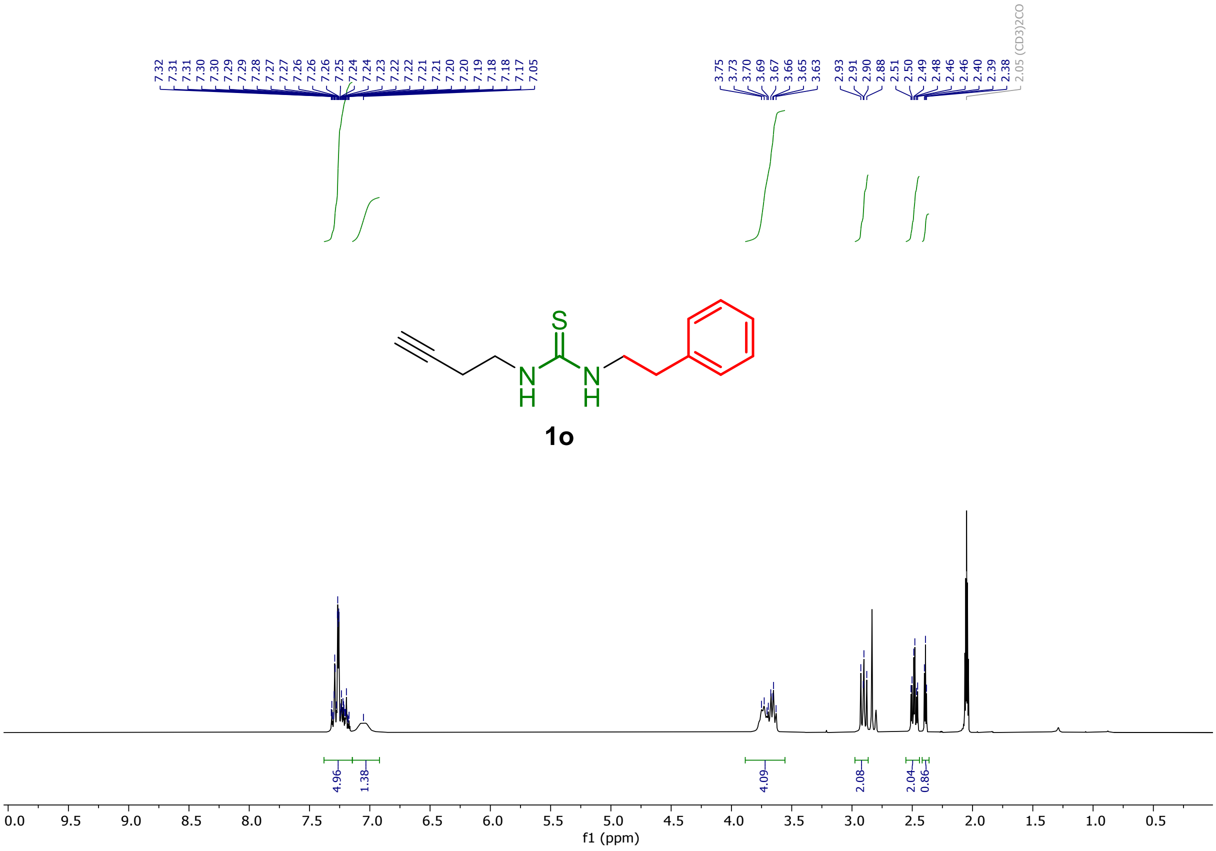

Figure S39. $^{13}C\{^1H\}$-APT NMR (75 MHz, CD_3COCD_3) spectrum of thiourea **1o**

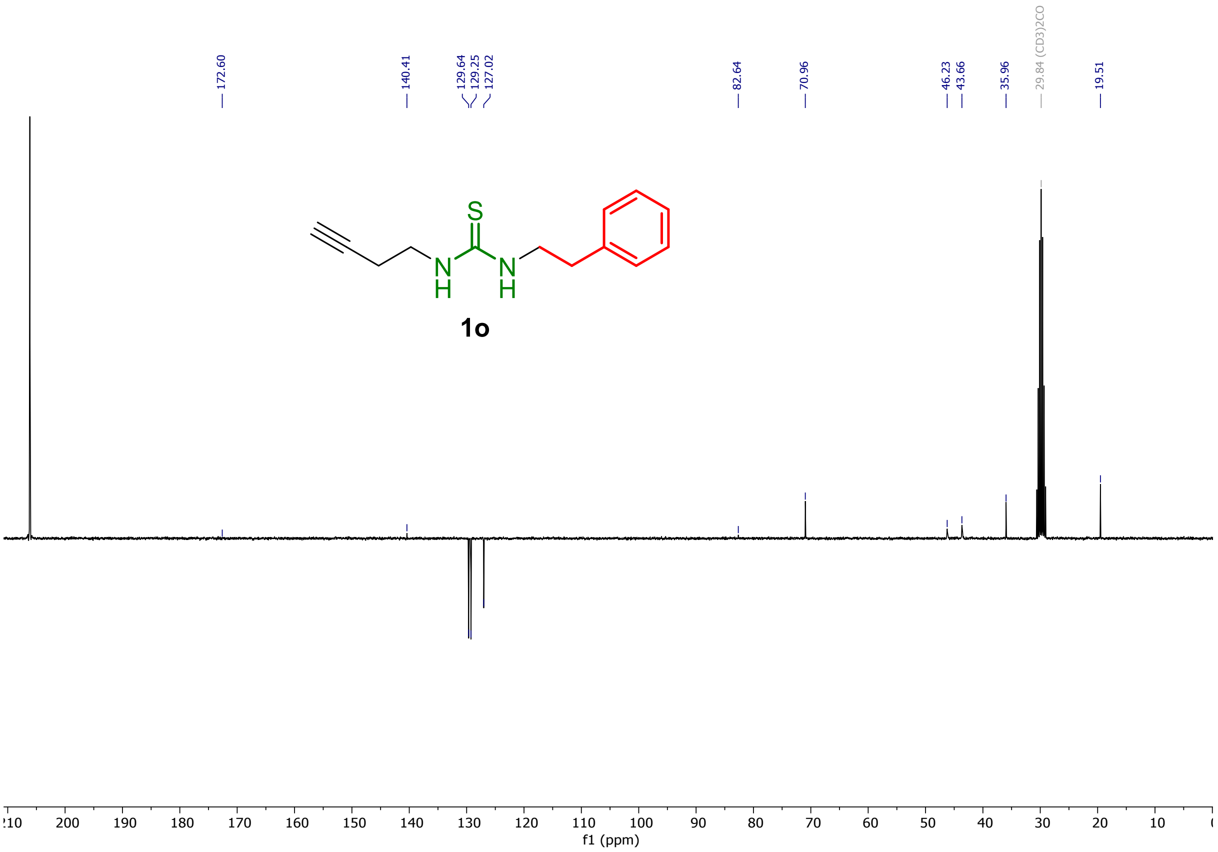

Figure S40. COSY NMR (400 MHz, CD_3COCD_3) spectrum of thiourea **1o**

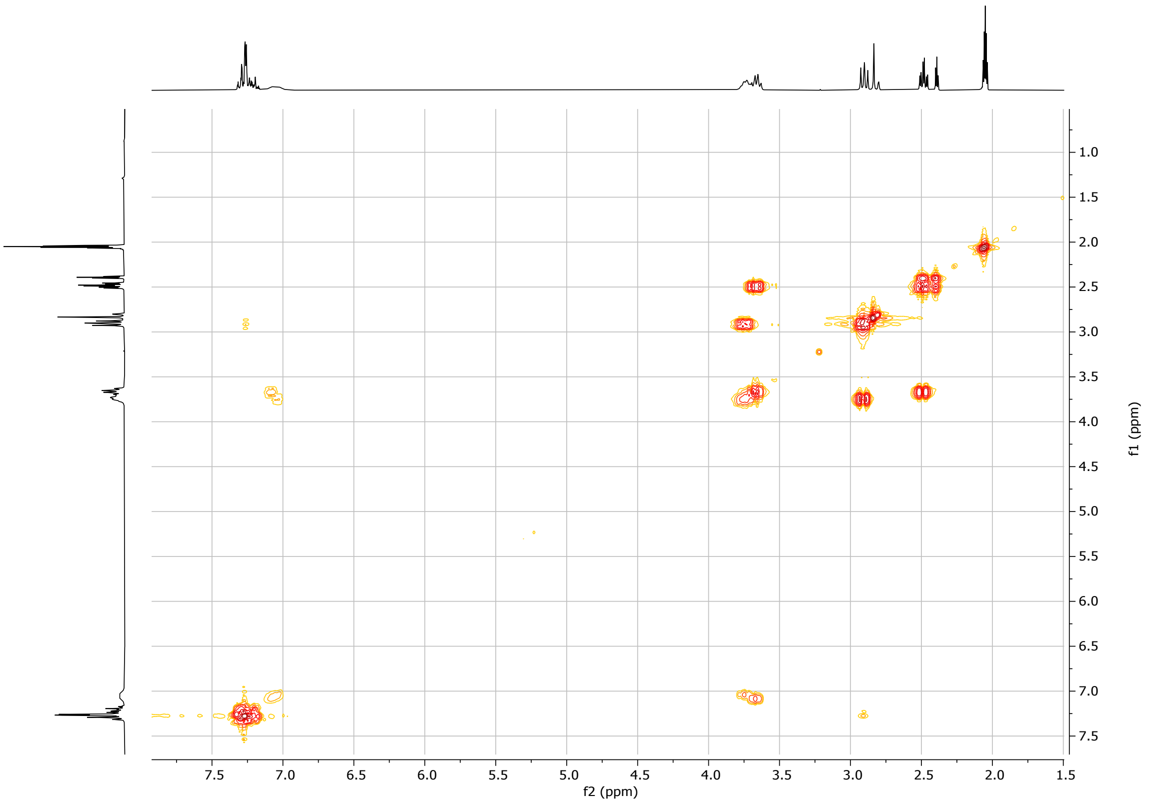

Figure S41. ^{1}H NMR (400 MHz, CD_3COCD_3) spectrum of 1-(but-3-yn-1-yl)-3-cyclohexylthiourea (**1p**)

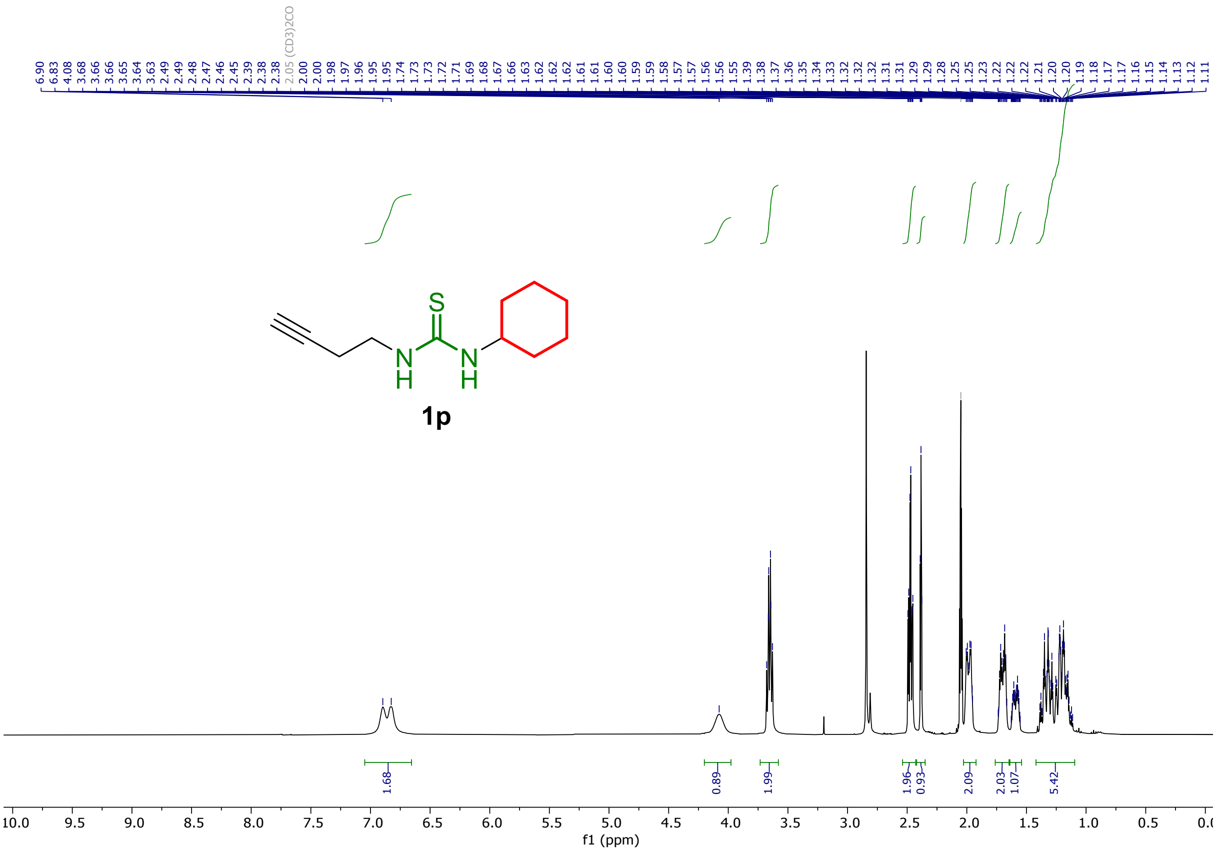

Figure S42. $^{13}C\{^{1}H\}$-APT NMR (101 MHz, CD_3COCD_3) spectrum of thiourea **1p**

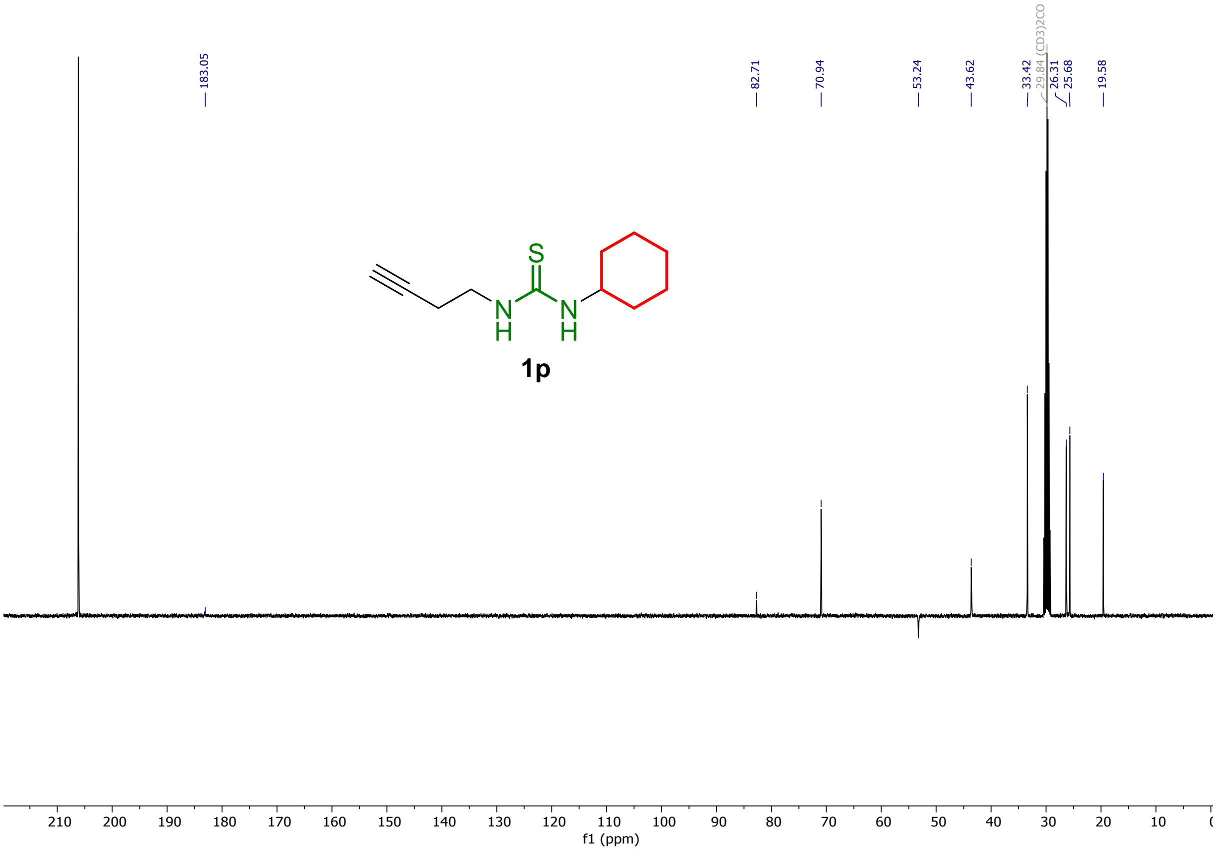

Figure S43. COSY NMR (400 MHz, CD_3COCD_3) spectrum of thiourea **1p**

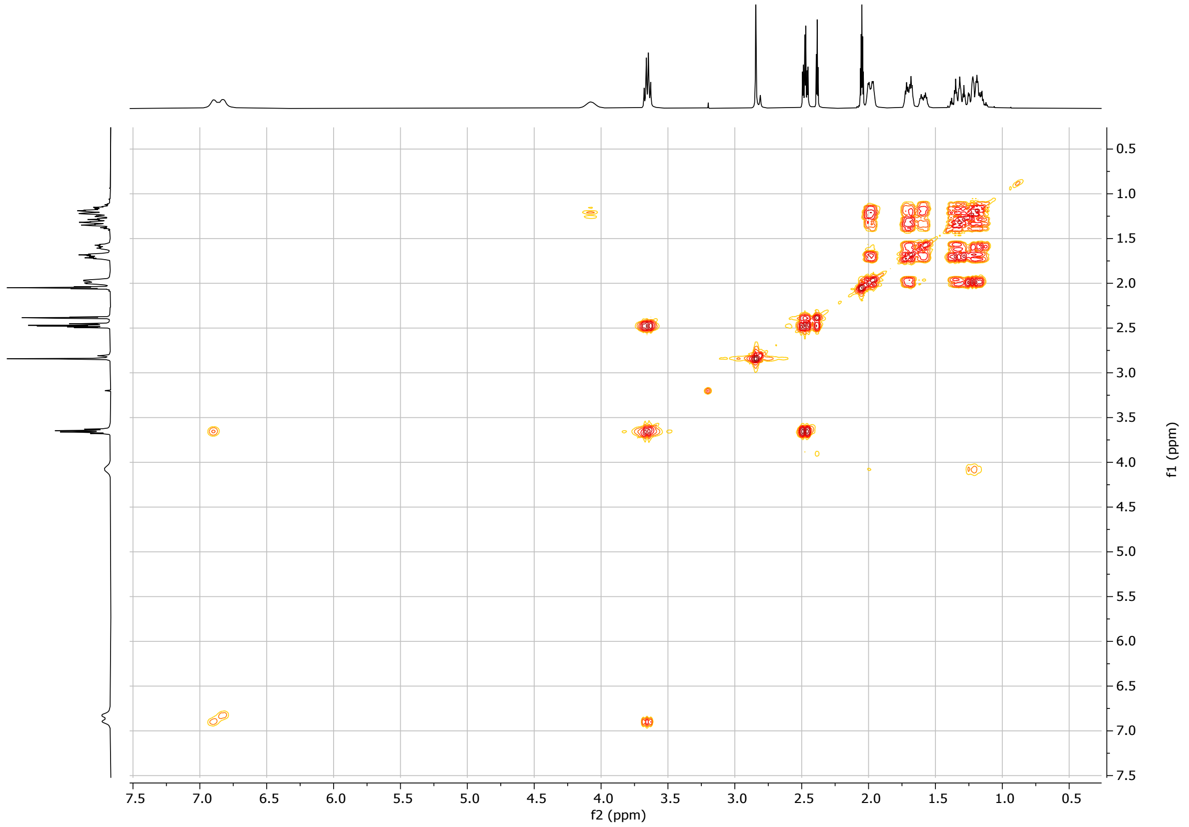

Figure S44. ^1H NMR (400 MHz, CD_3COCD_3) spectrum of 1-(pent-4-yn-1-yl)-3-phenylthiourea (**1q**)

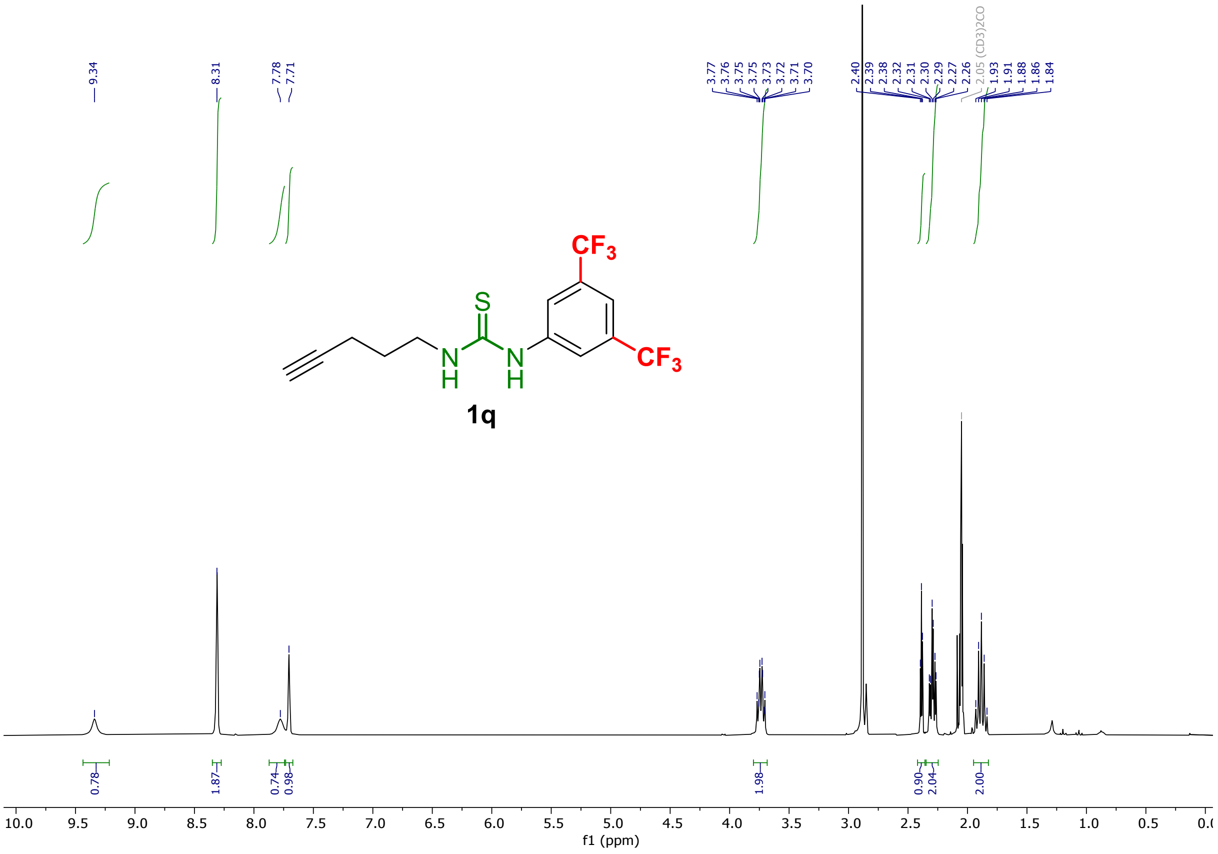

Figure S45. $^{13}C\{^1H\}$-APT NMR (101 MHz, CD_3COCD_3) spectrum of thiourea (**1q**)

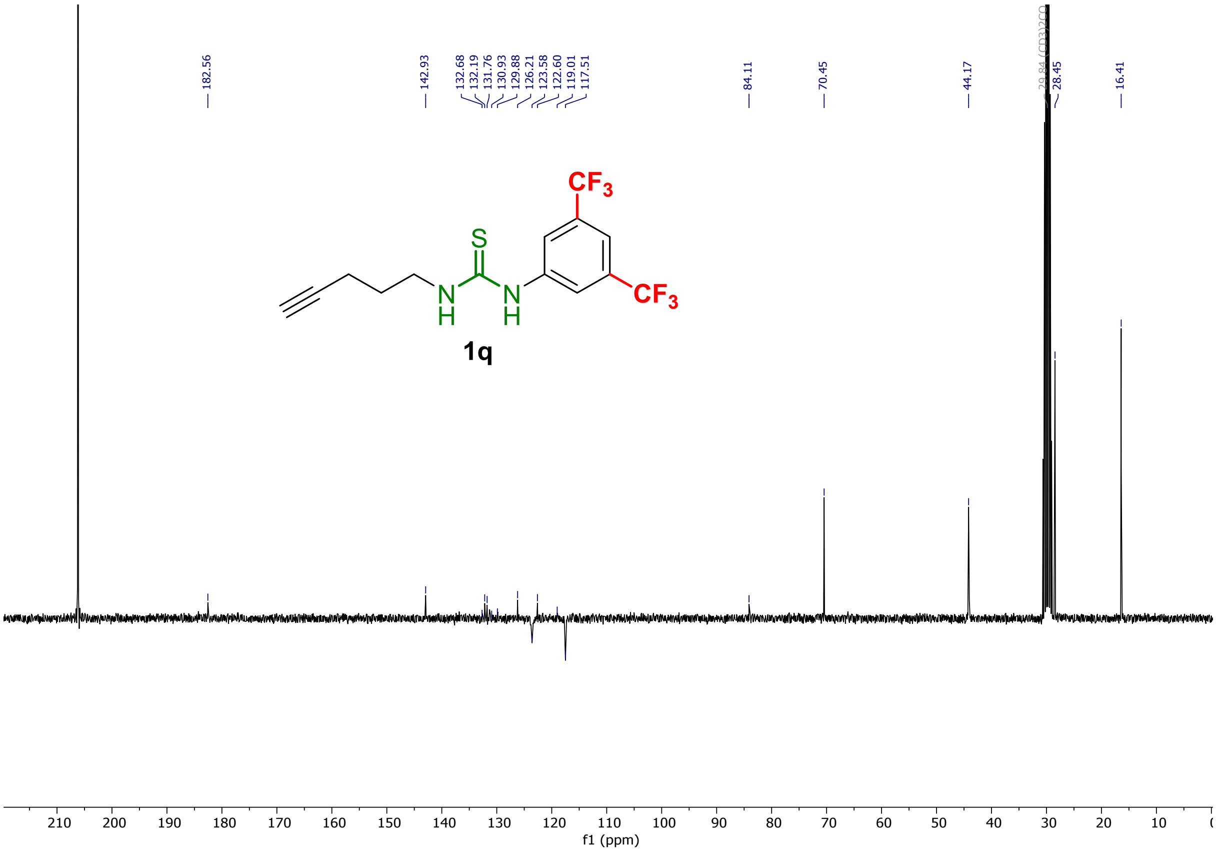

5.2. Spectra of 5,6-dihydro-4*H*-1,3-thiazines 2'

Figure S46. ^{1}H NMR (300 MHz, $CDCl_3$) spectrum of 6-methylene-*N*-phenyl-5,6-dihydro-4*H*-1,3-thiazin-2-amine (**2a'**)

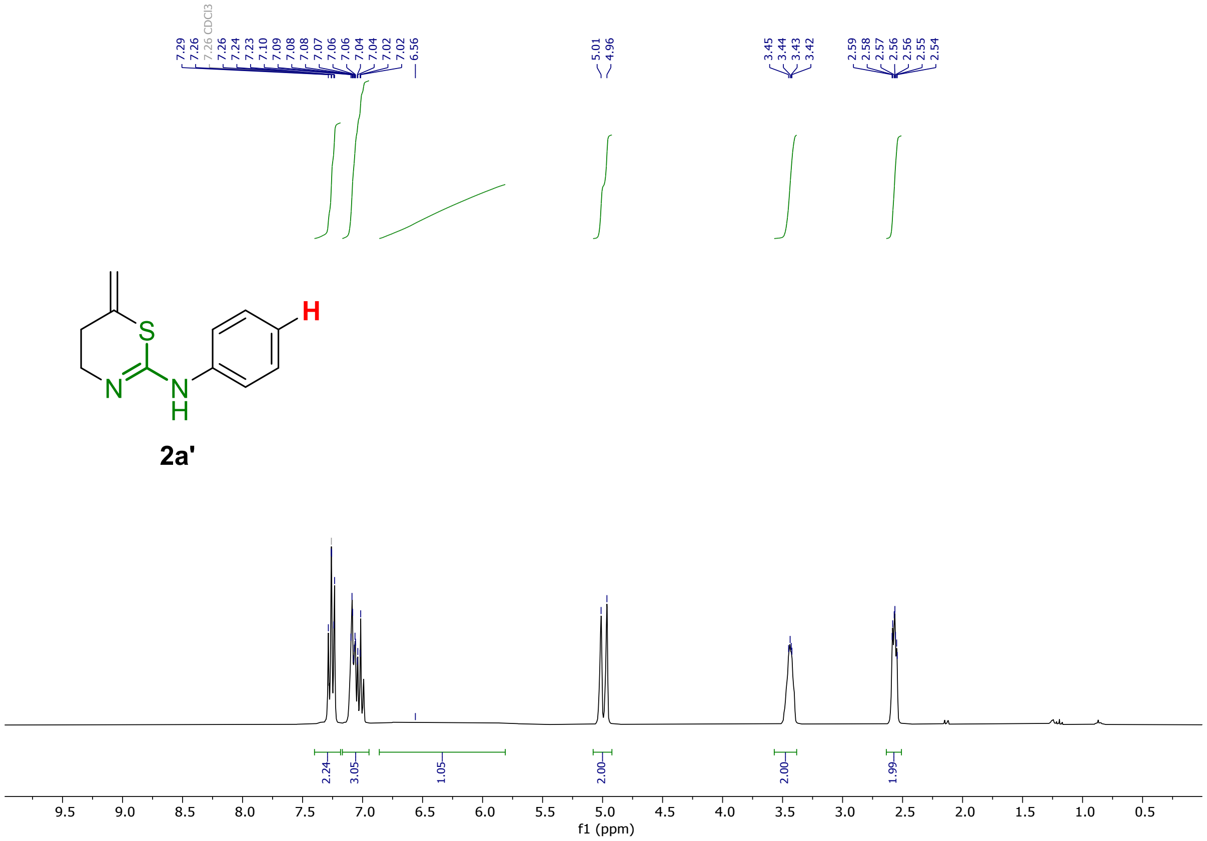

Figure S47. ^{13}C{^{1}H}-APT NMR (75 MHz, $CDCl_3$) spectrum of 1,3-thiazine **2a'**

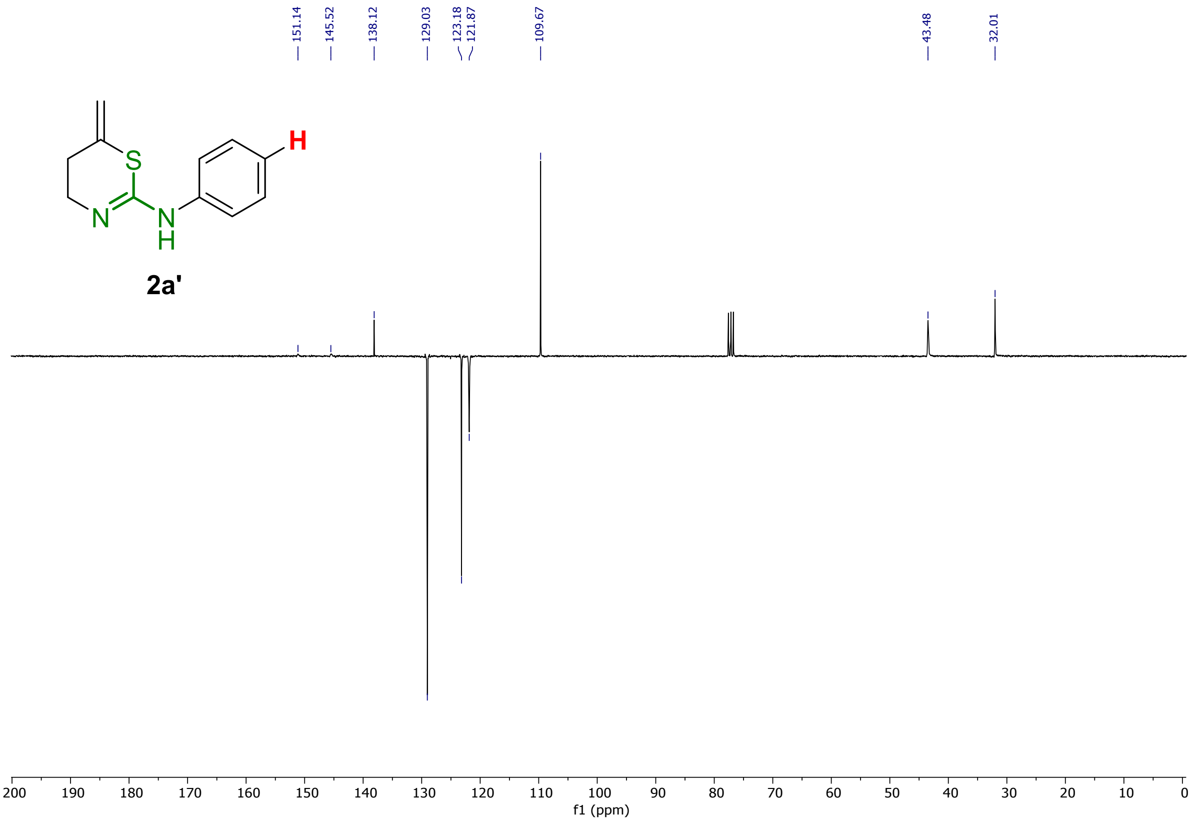

Figure S48. COSY NMR (300 MHz, CD_3COCD_3) spectrum of 1,3-thiazine **2a'**

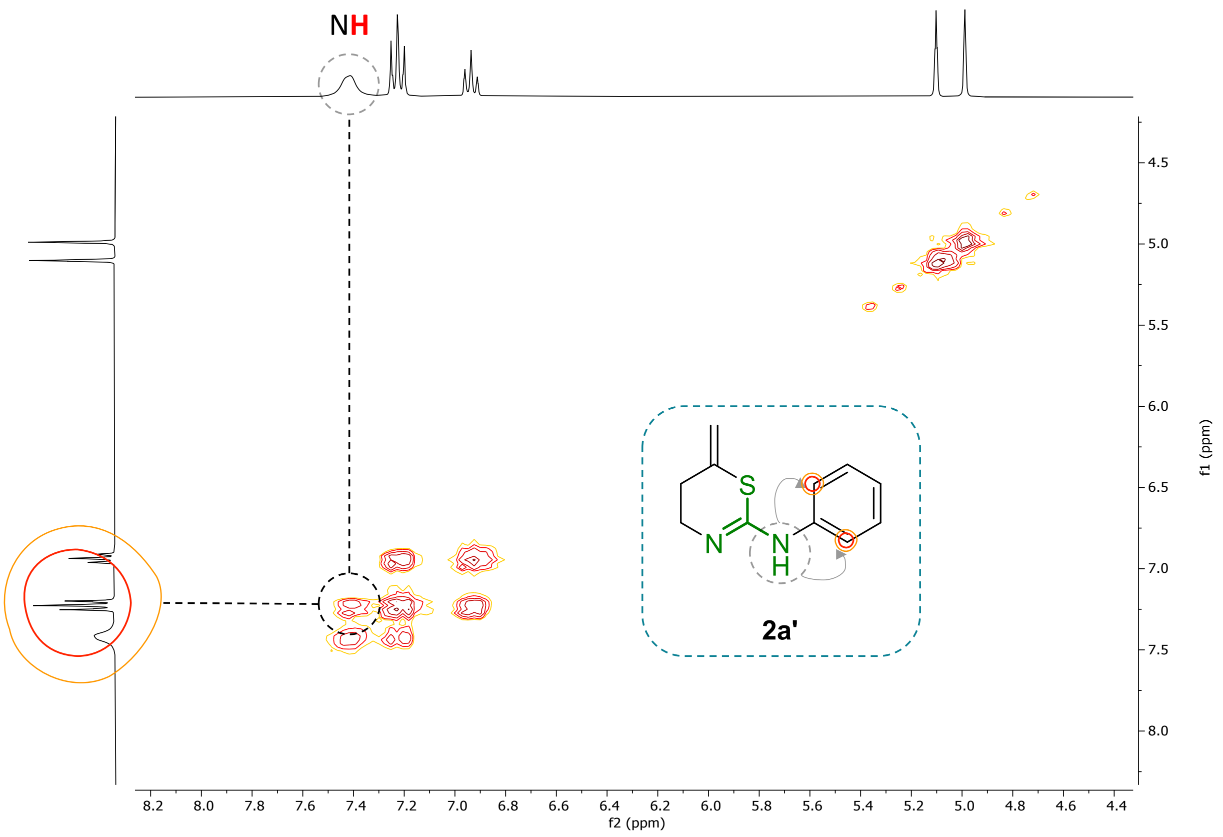

Figure S49. HSQC NMR (300 MHz, 75 MHz, $CDCl_3$) spectrum of 1,3-thiazine **2a'**

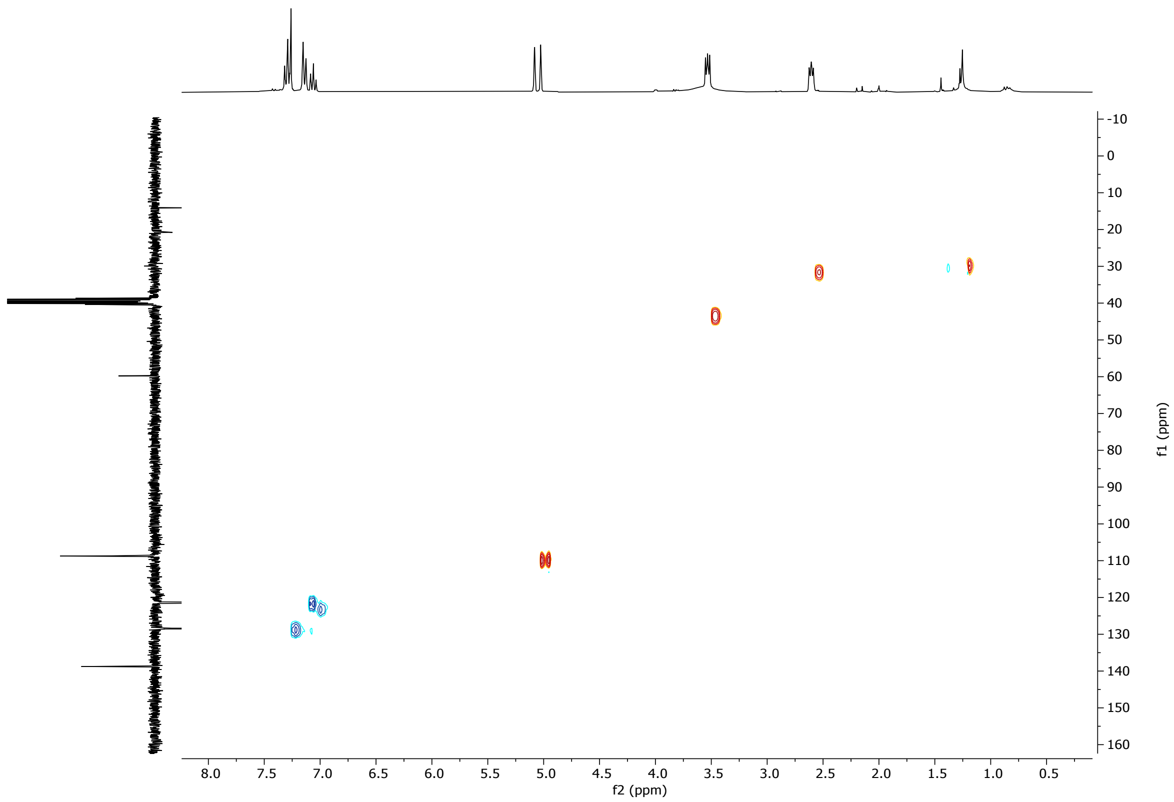

Figure S50. HMBC NMR (300 MHz, 75 MHz, CD_3COCD_3) spectrum of 1,3-thiazine **2a'**

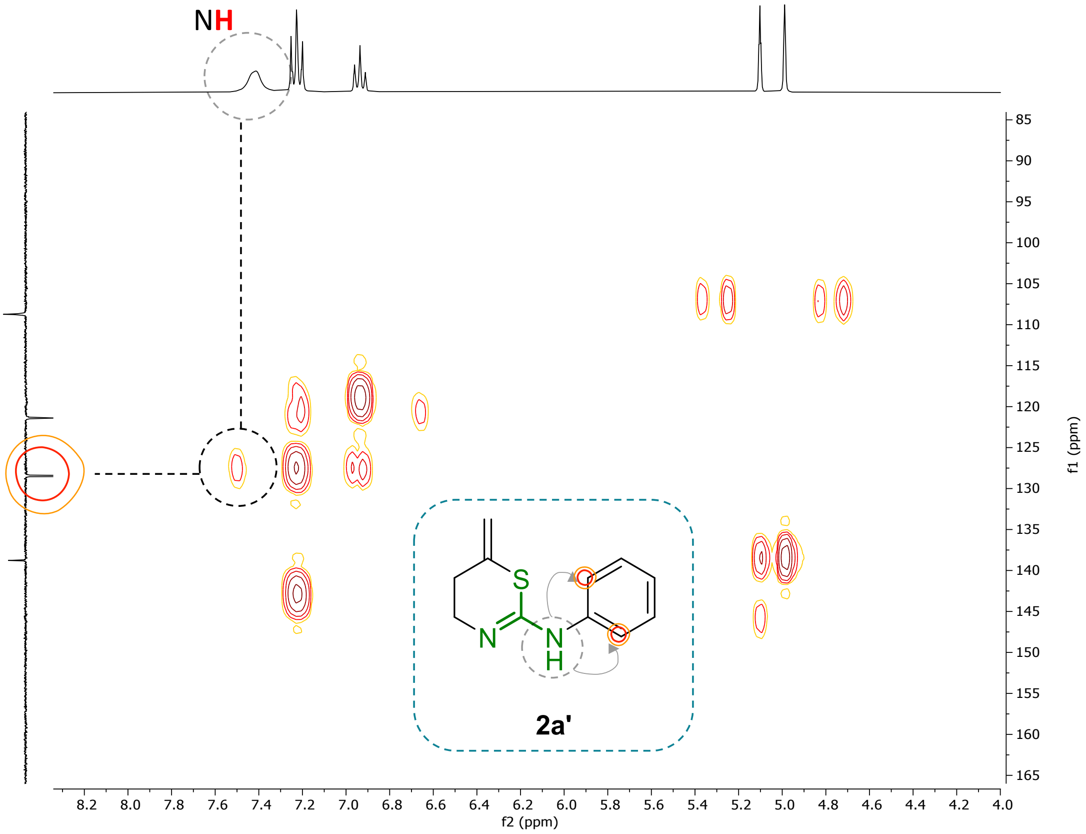

Figure S51. ^{1}H NMR (300 MHz, CD_3COCD_3) spectrum of *N*-(4-fluorophenyl)-6-methylene-5,6-dihydro-4*H*-1,3-thiazin-2-amine (**2b'**)

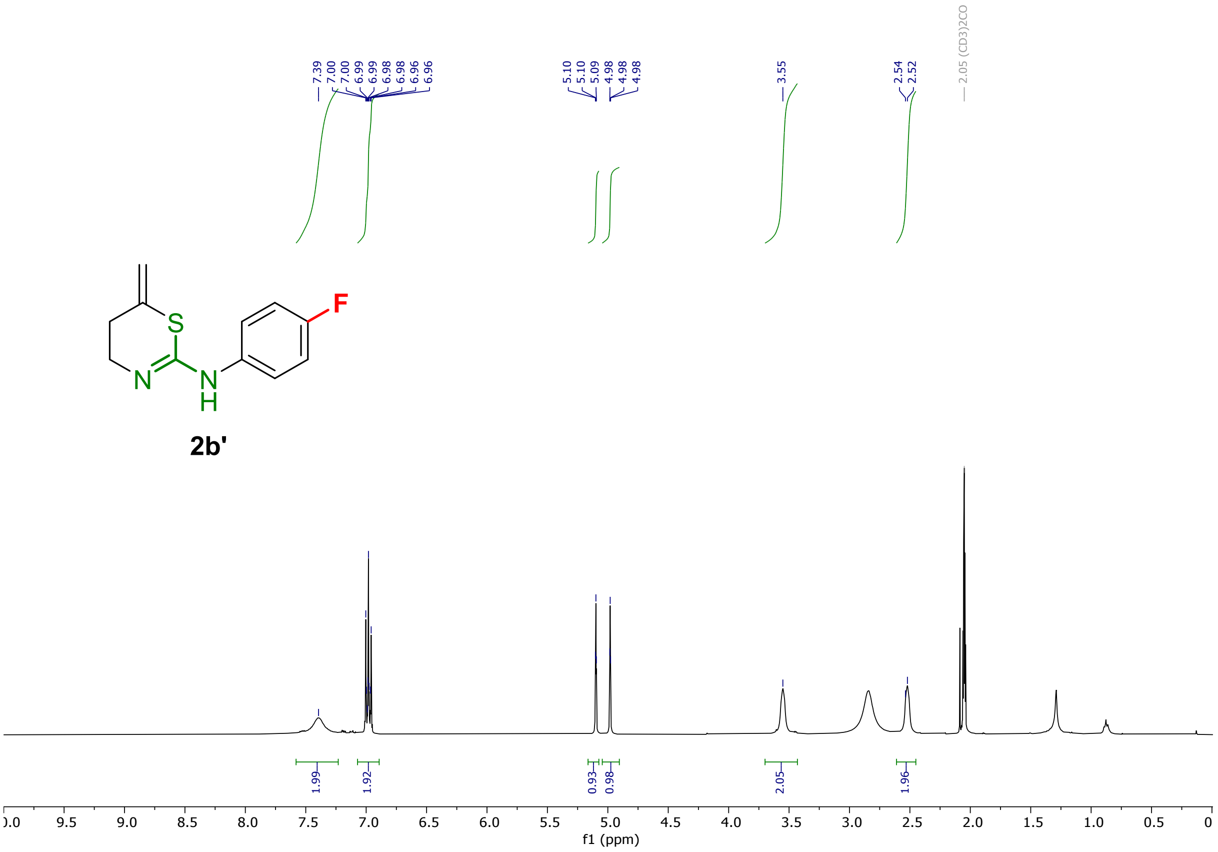

Figure S52. $^{13}C\{^{1}H\}$-APT NMR (75 MHz, $CDCl_3$) spectrum 1,3-thiazine **2b'**

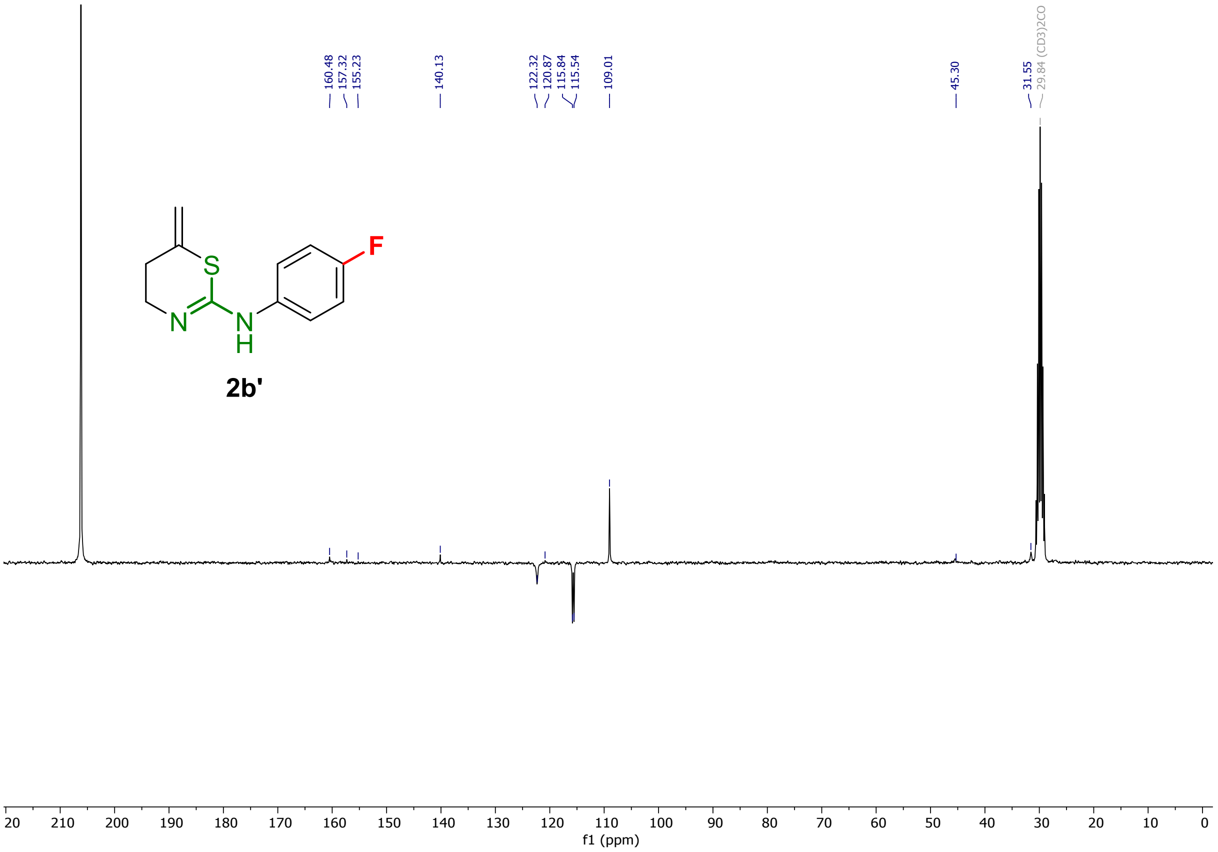

Figure S53. COSY NMR (300 MHz, $CDCl_3$) spectrum of 1,3-thiazine **2b'**

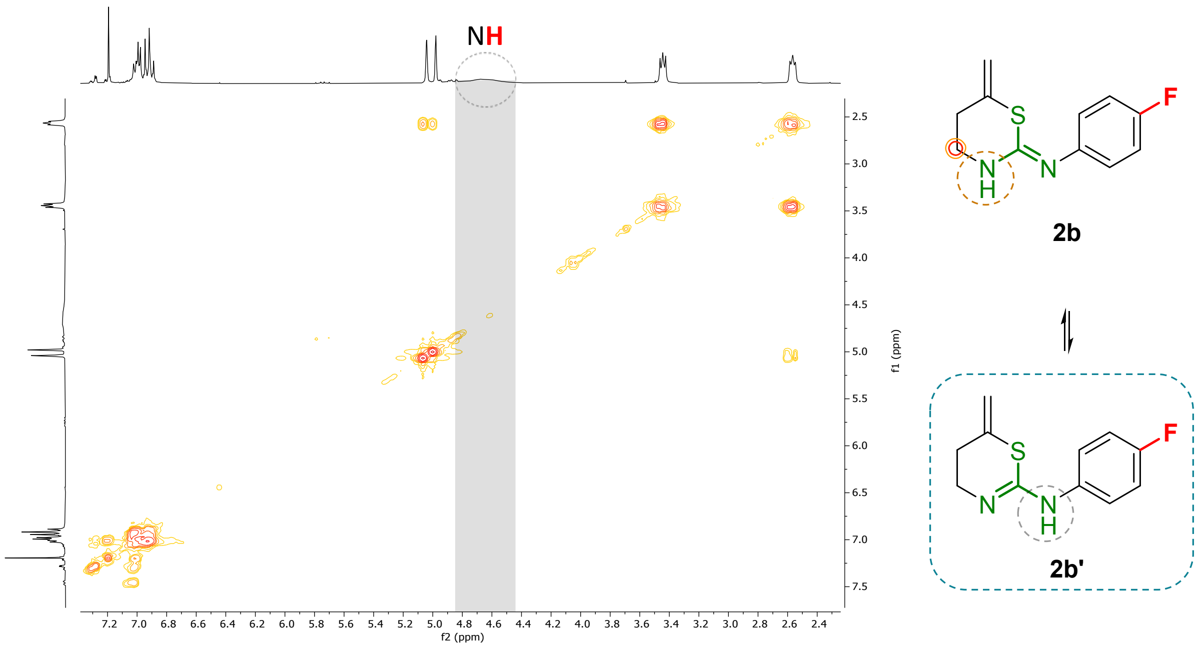

Figure S54. HSQC NMR (300 MHz, 75 MHz, $CDCl_3$) spectrum of 1,3-thiazine **2b'**

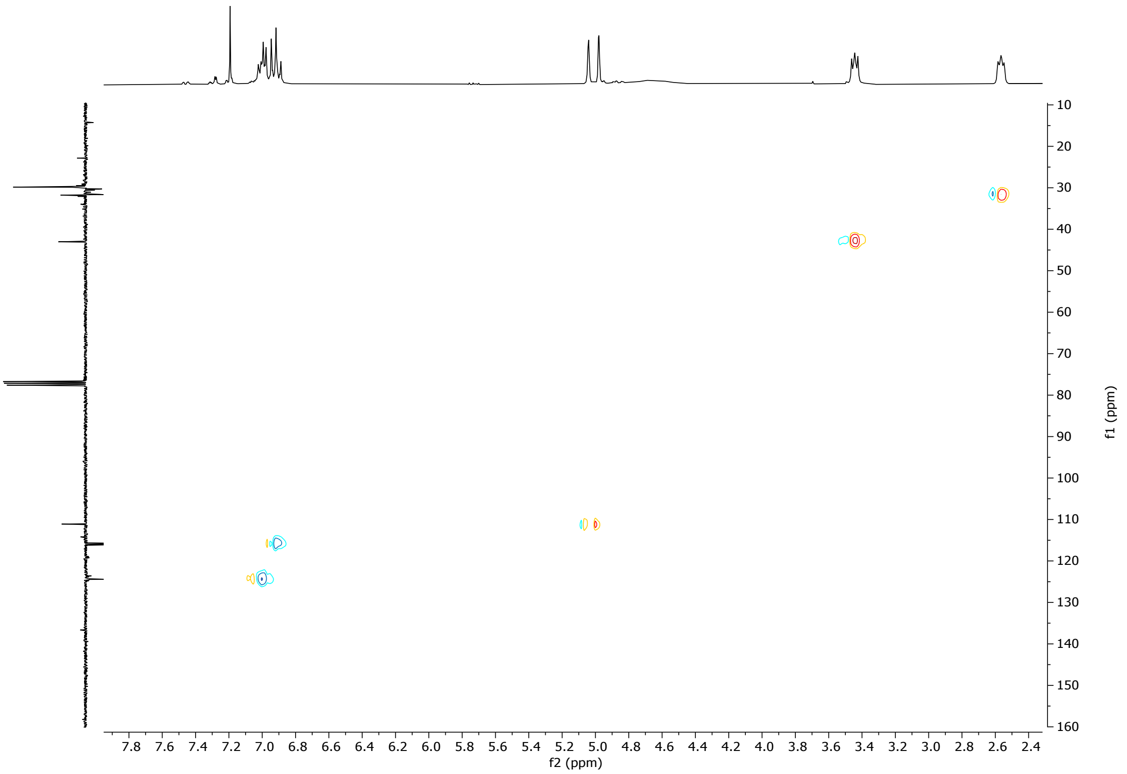

Figure S55. ^{1}H NMR (300 MHz, $CDCl_3$) spectrum of *N*-(3-fluorophenyl)-6-methylene-5,6-dihydro-4*H*-1,3-thiazin-2-amine (**2c'**)

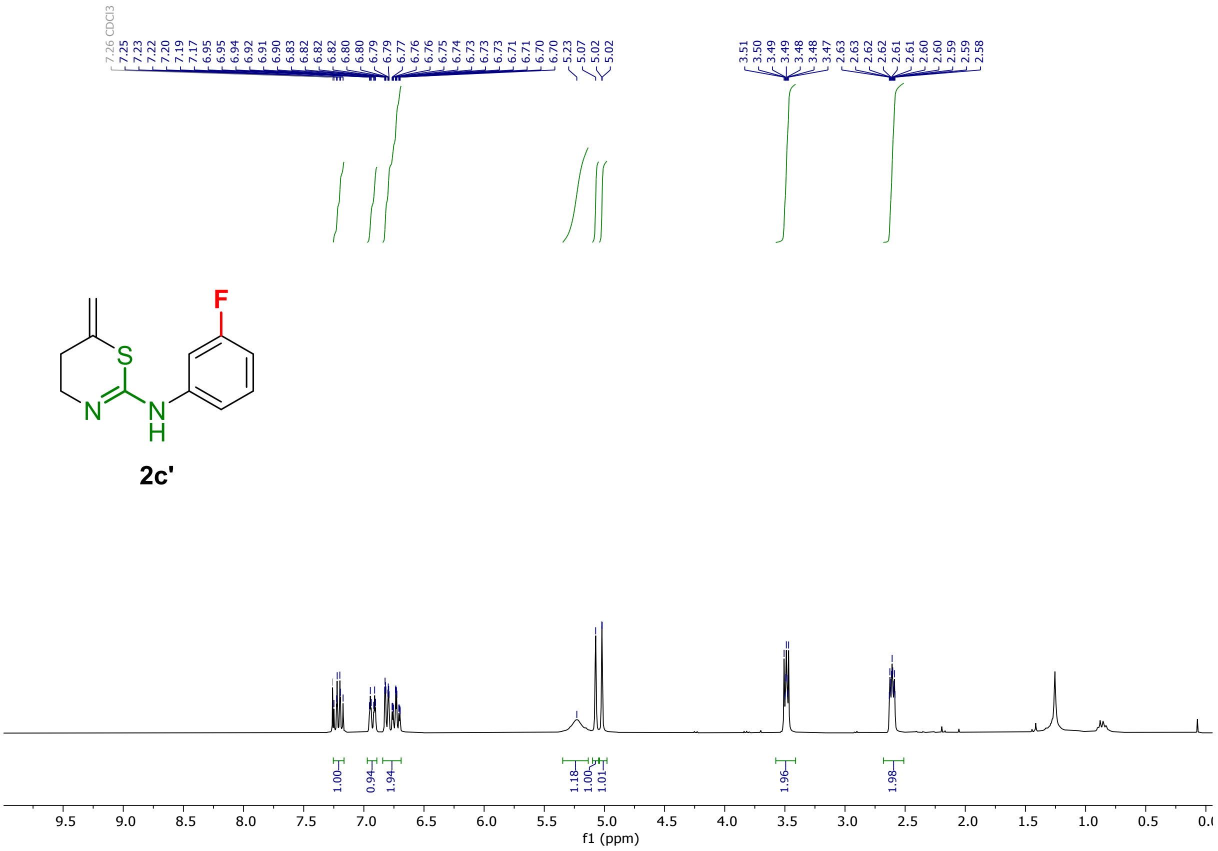

Figure S56. $^{13}C\{^{1}H\}$-APT NMR (75 MHz, CD_3COCD_3) spectrum of 1,3-thiazine **2c'**

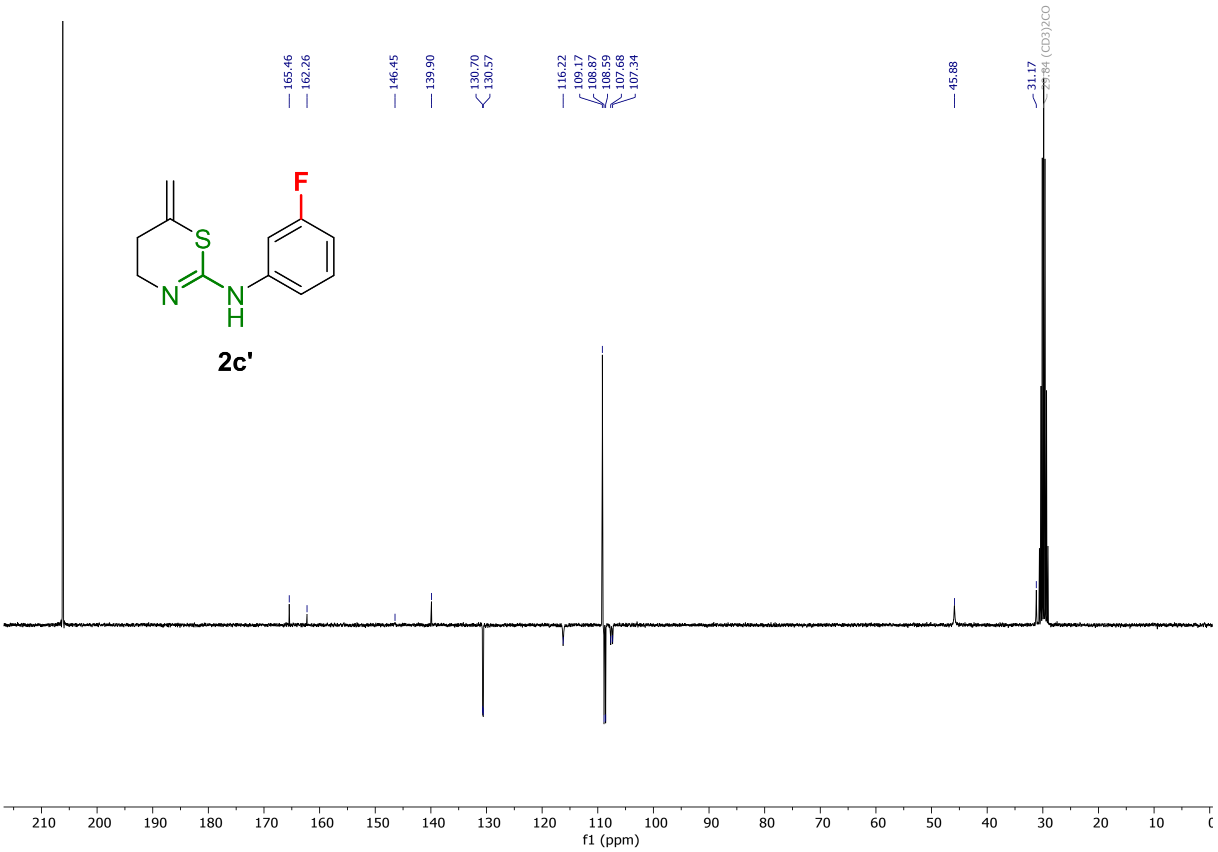

Figure S57. COSY NMR (300 MHz, CD_3COCD_3) spectrum of 1,3-thiazine **2c'**

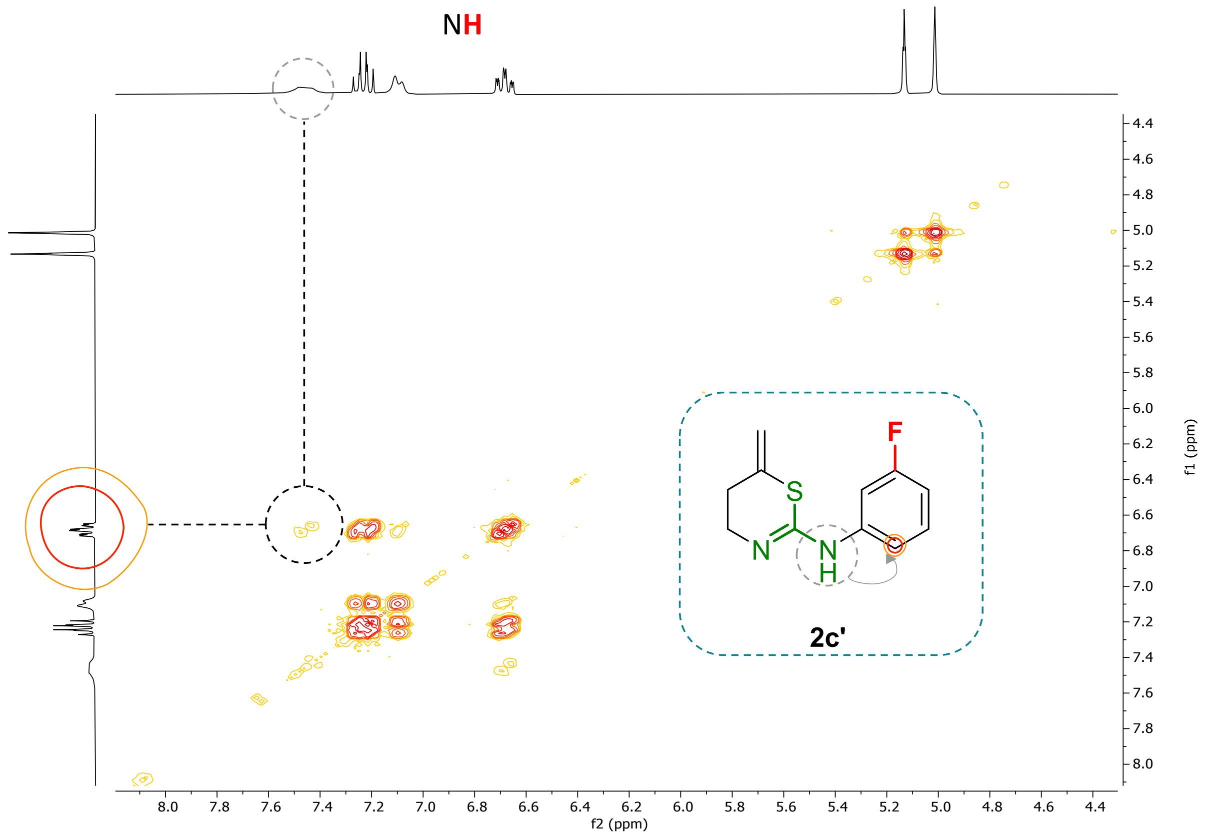

Figure S58. HSQC NMR (300 MHz, 75 MHz, $CDCl_3$) spectrum of 1,3-thiazine **2c'**

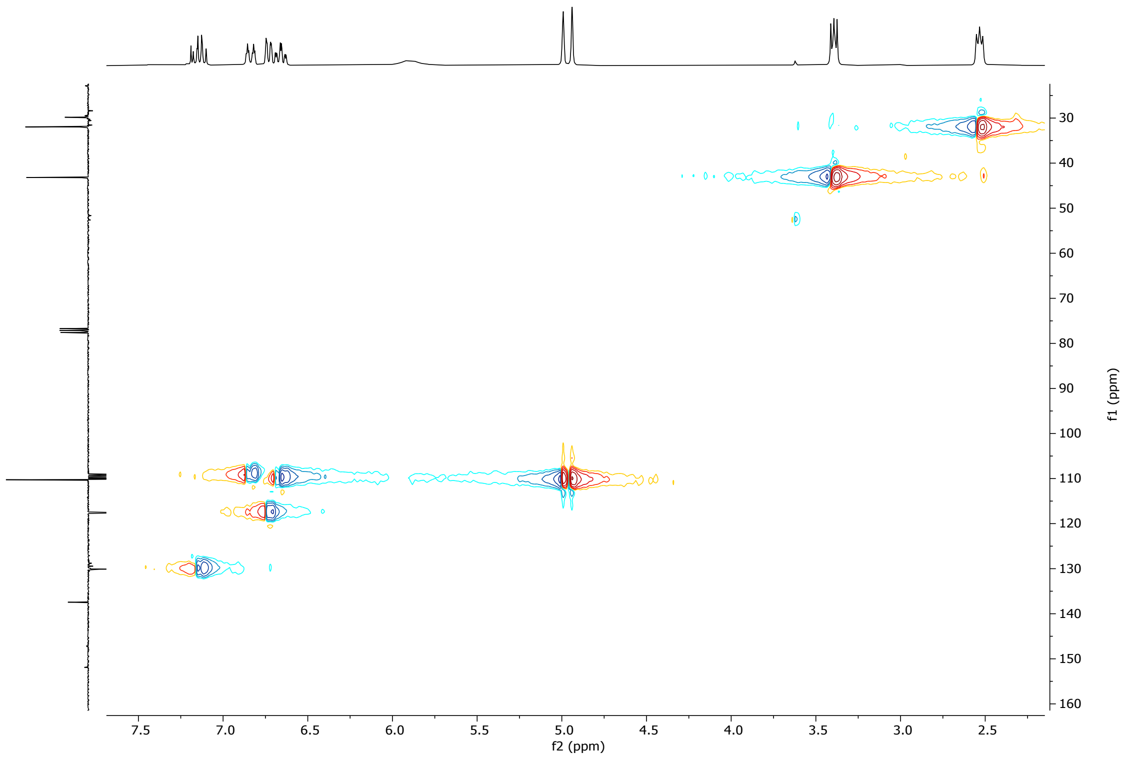

Figure S59. ^{1}H NMR (300 MHz, CD_3COCD_3) spectrum of *N*-(4-chlorophenyl)-6-methylene-5,6-dihydro-4*H*-1,3-thiazin-2-amine (**2d'**)

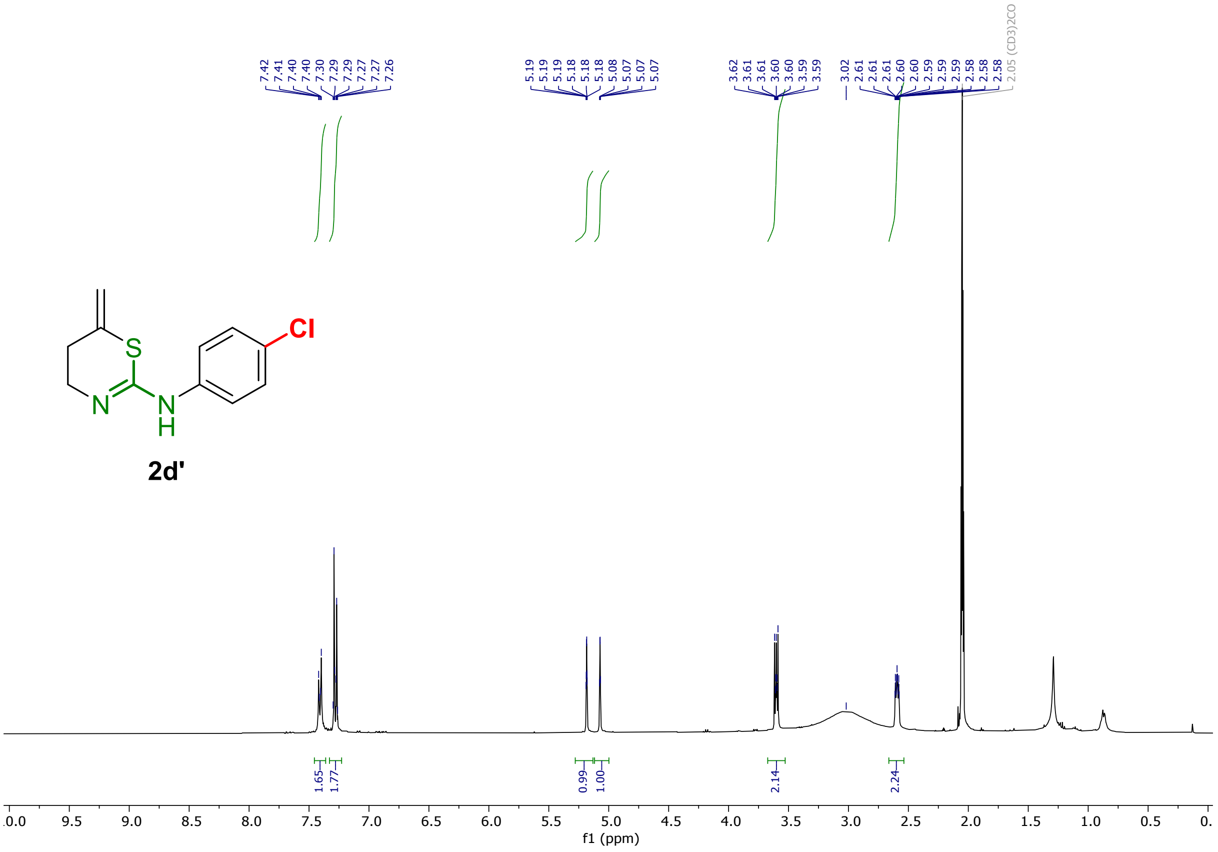

Figure S60. $^{13}C\{^{1}H\}$-APT NMR (75 MHz, $CDCl_3$) spectrum of 1,3-thiazine **2d'**

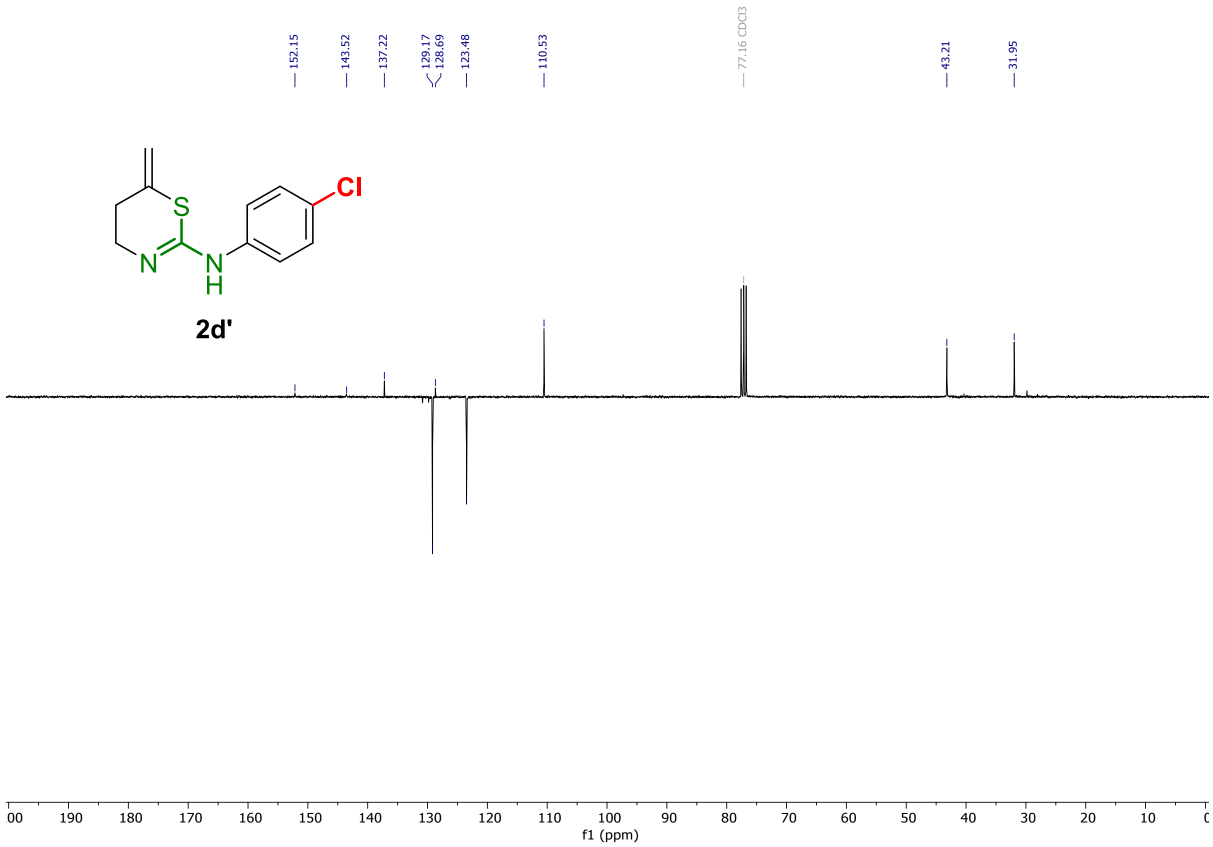

Figure S61. COSY NMR (300 MHz, $CDCl_3$) spectrum of thiazine **2d'**

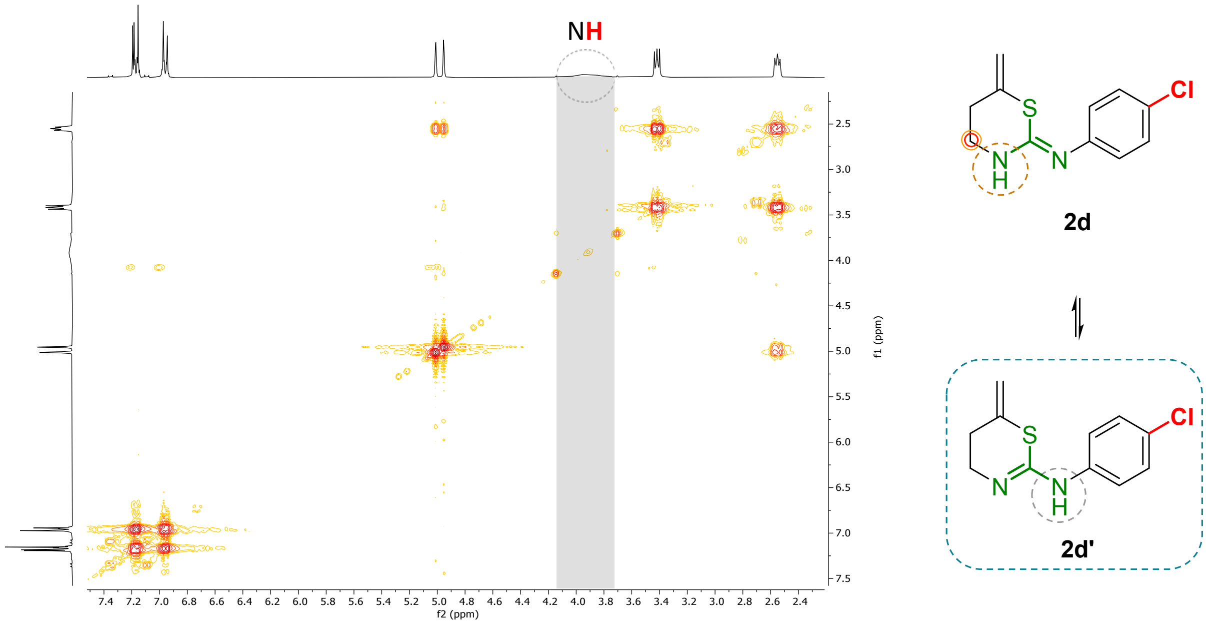

Figure S62. HSQC NMR (300 MHz, 75 MHz, $CDCl_3$) spectrum of 1,3-thiazine **2d'**

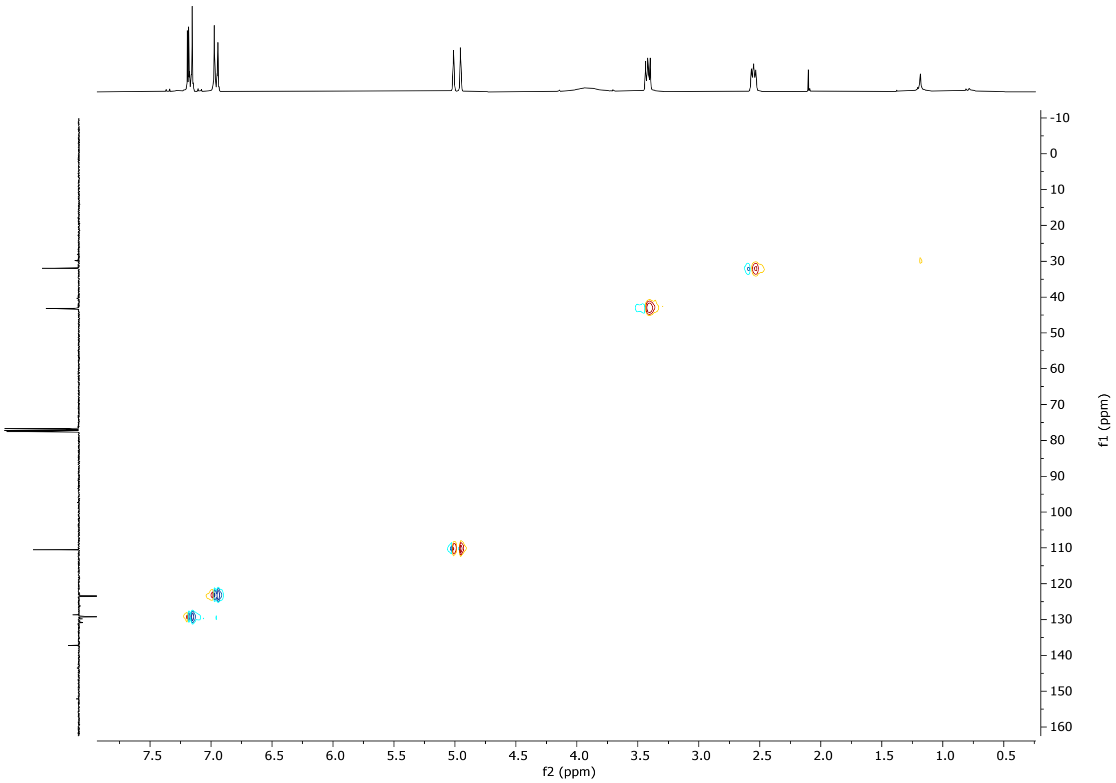

Figure S63. ^{1}H NMR (400 MHz, $CDCl_3$) spectrum of *N*-(4-bromophenyl)-6-methylene-5,6-dihydro-4*H*-1,3-thiazin-2-amine (**2e'**)

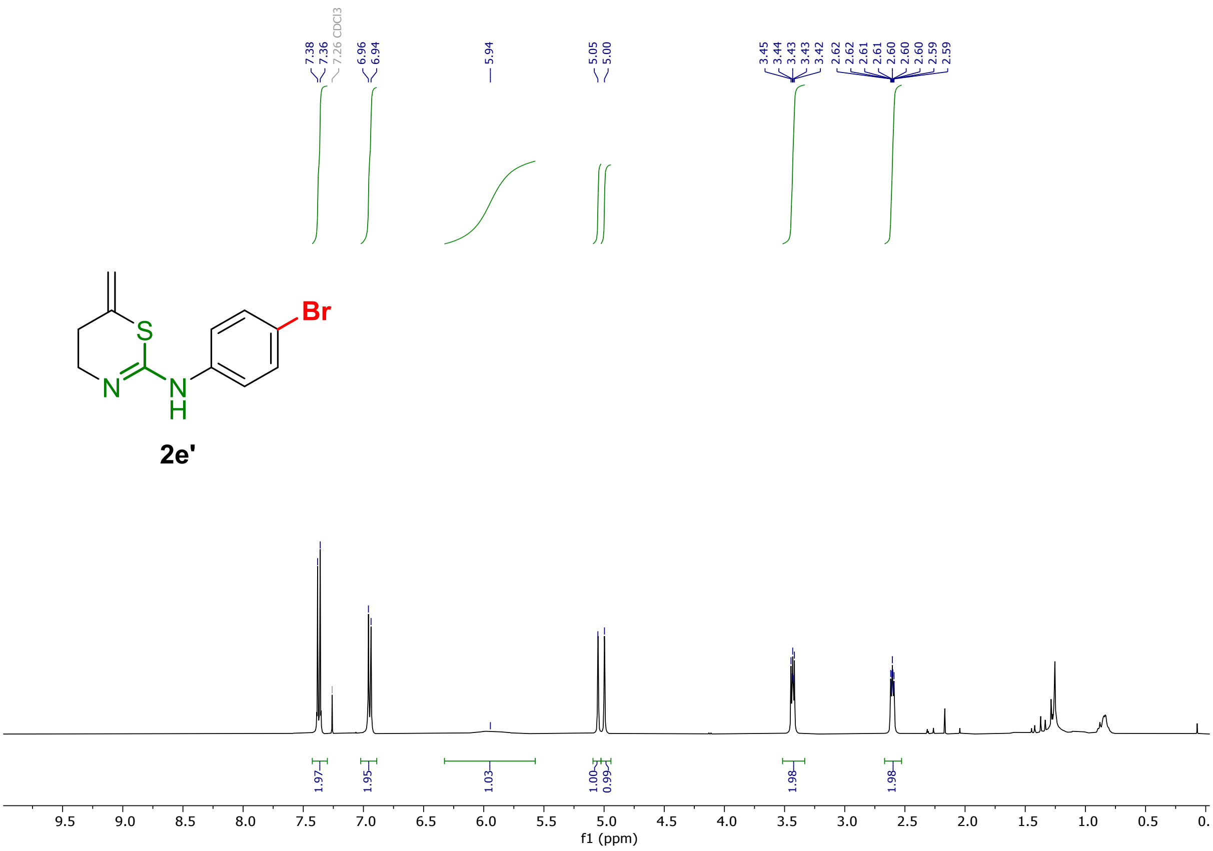

Figure S64. $^{13}C\{^{1}H\}$-APT NMR (101 MHz, $CDCl_3$) spectrum of 1,3-thiazine **2e'**

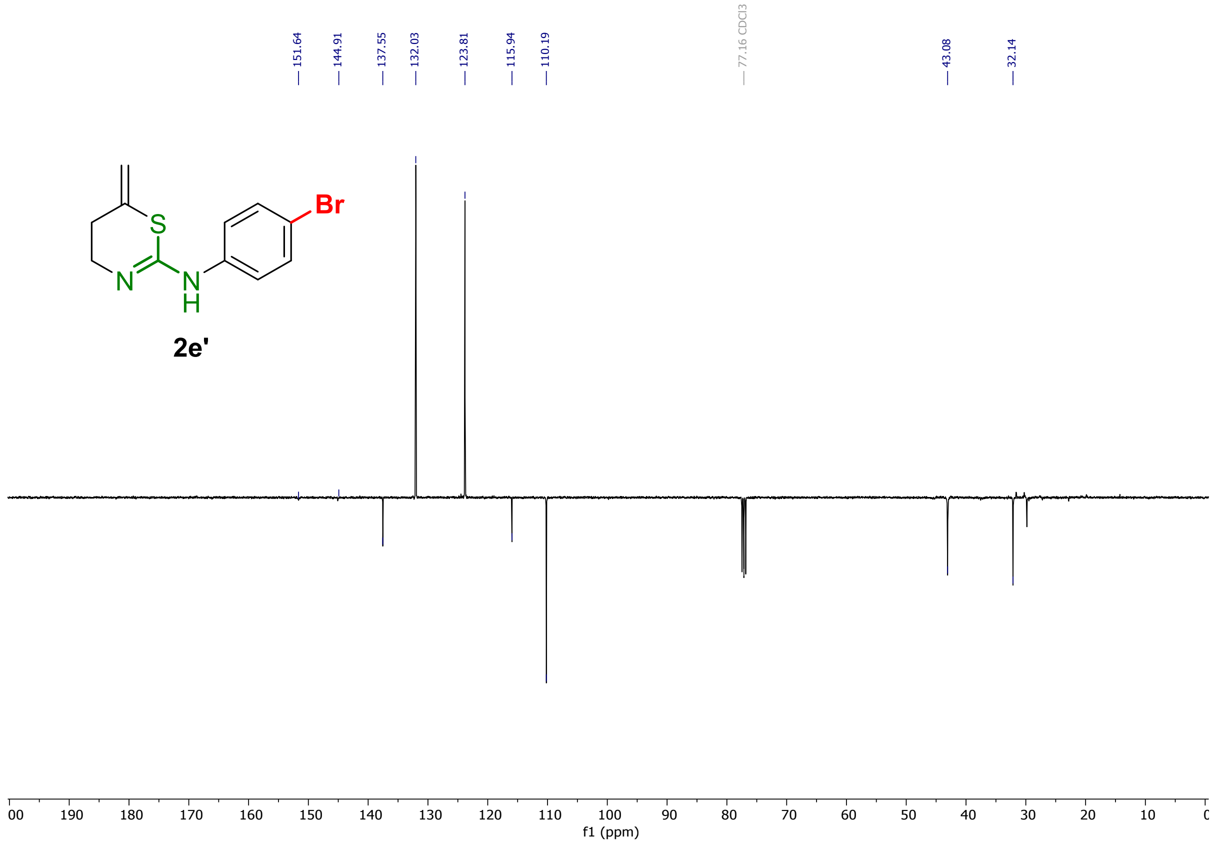

Figure S65. COSY NMR (400 MHz, $CDCl_3$) spectrum of 1,3-thiazine **2e'**

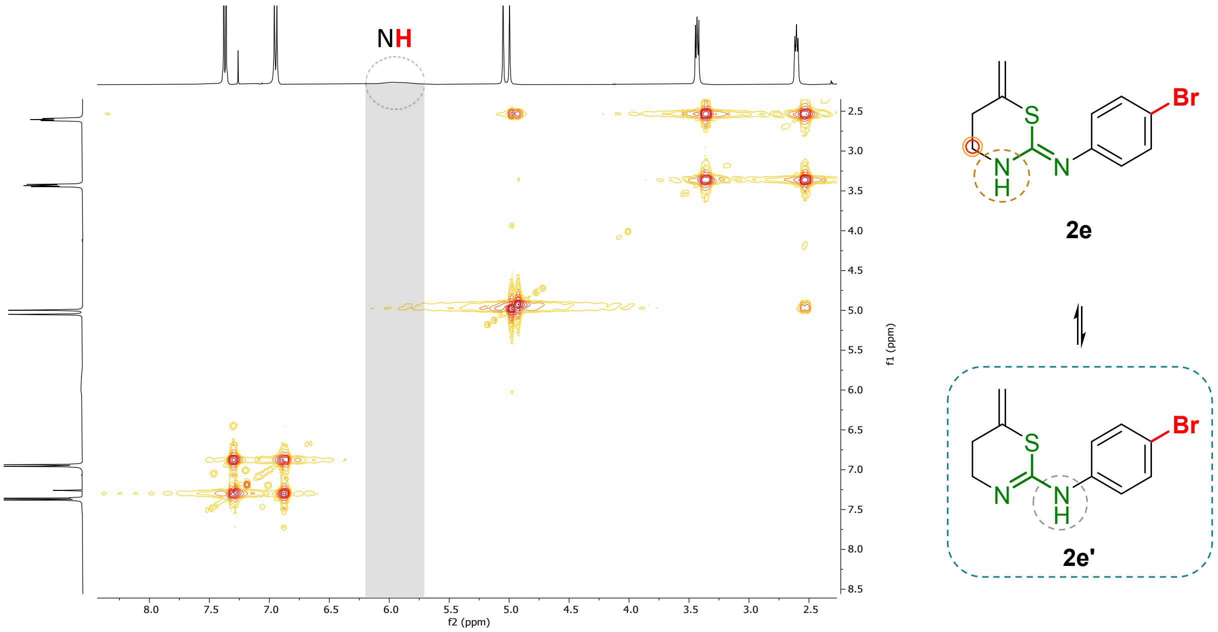

Figure S66. HSQC NMR (400 MHz, 101 MHz, $CDCl_3$) spectrum of 1,3-thiazine **2e'**

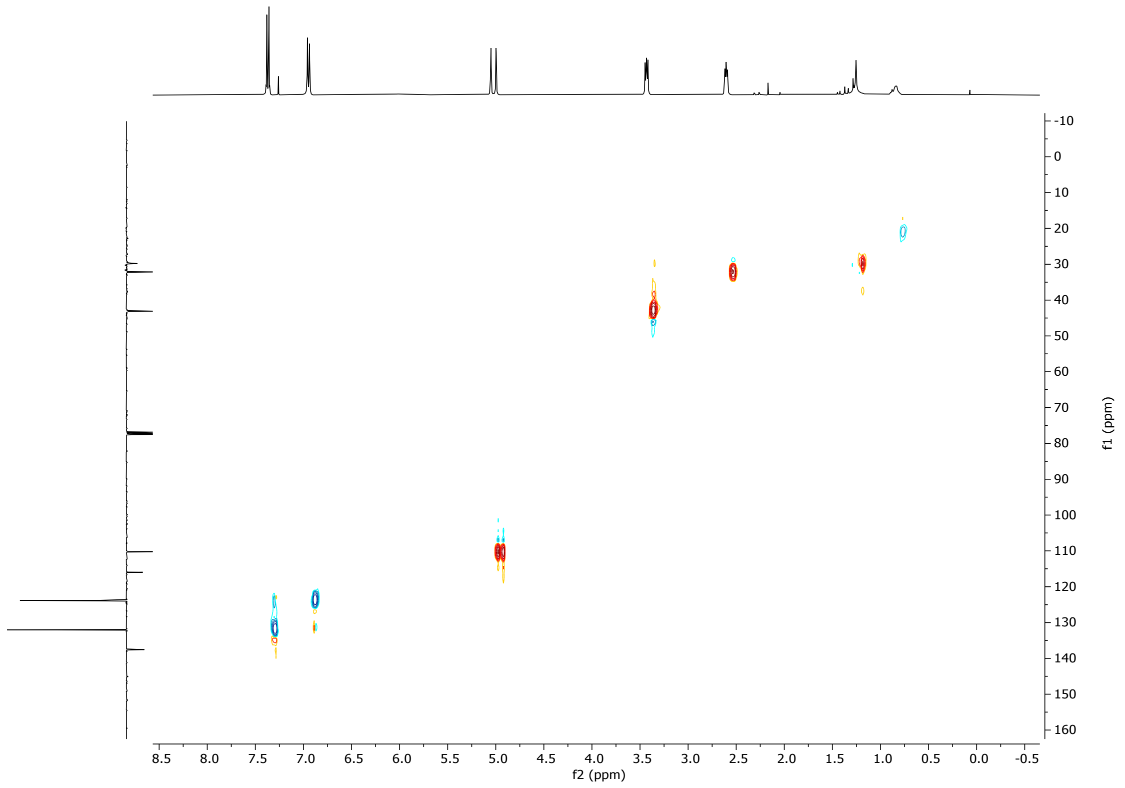

Figure S67. ^{1}H NMR (400 MHz, CD_3COCD_3) spectrum of 6-methylene-*N*-(4-(trifluoromethyl)phenyl)-5,6-dihydro-4*H*-1,3-thiazin-2-amine (**2f'**)

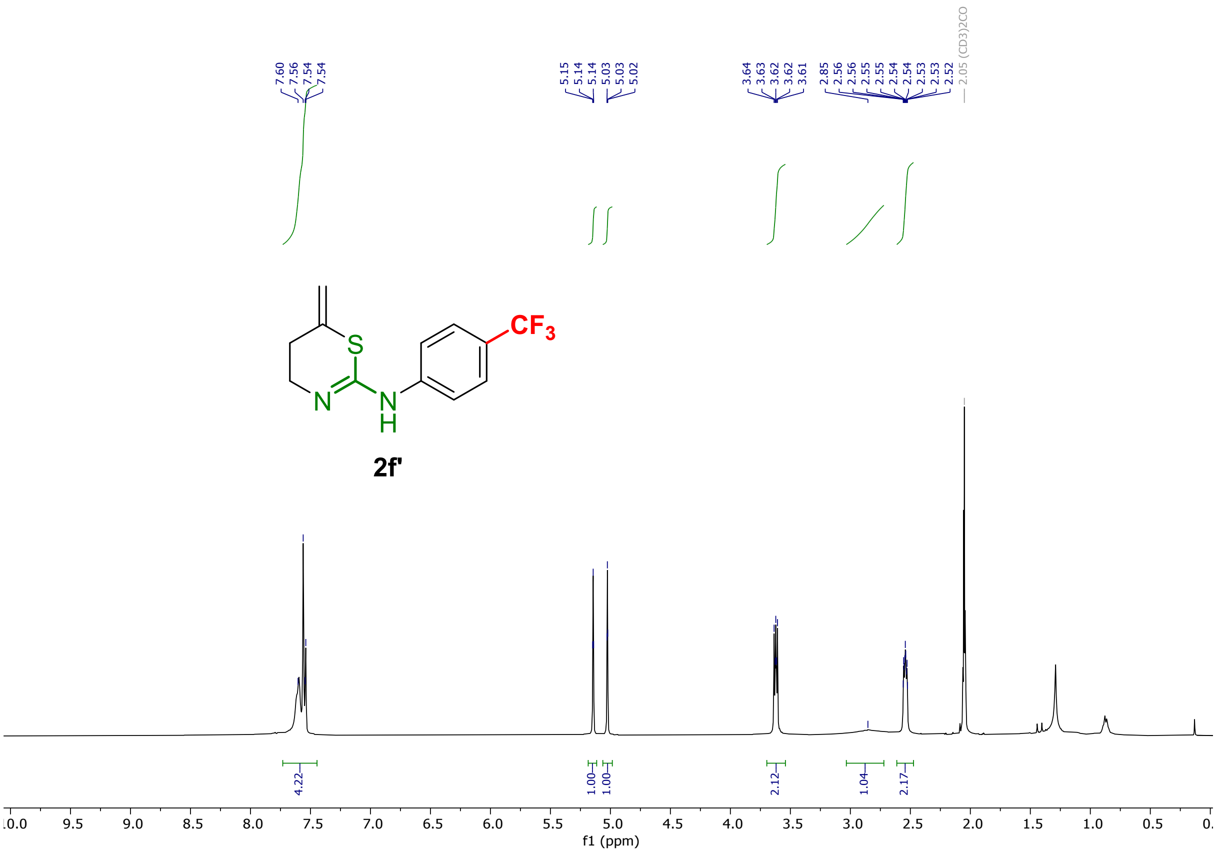

Figure S68. $^{13}C\{^1H\}$-APT NMR (75 MHz, $CDCl_3$) spectrum of 1,3-thiazine **2f'**

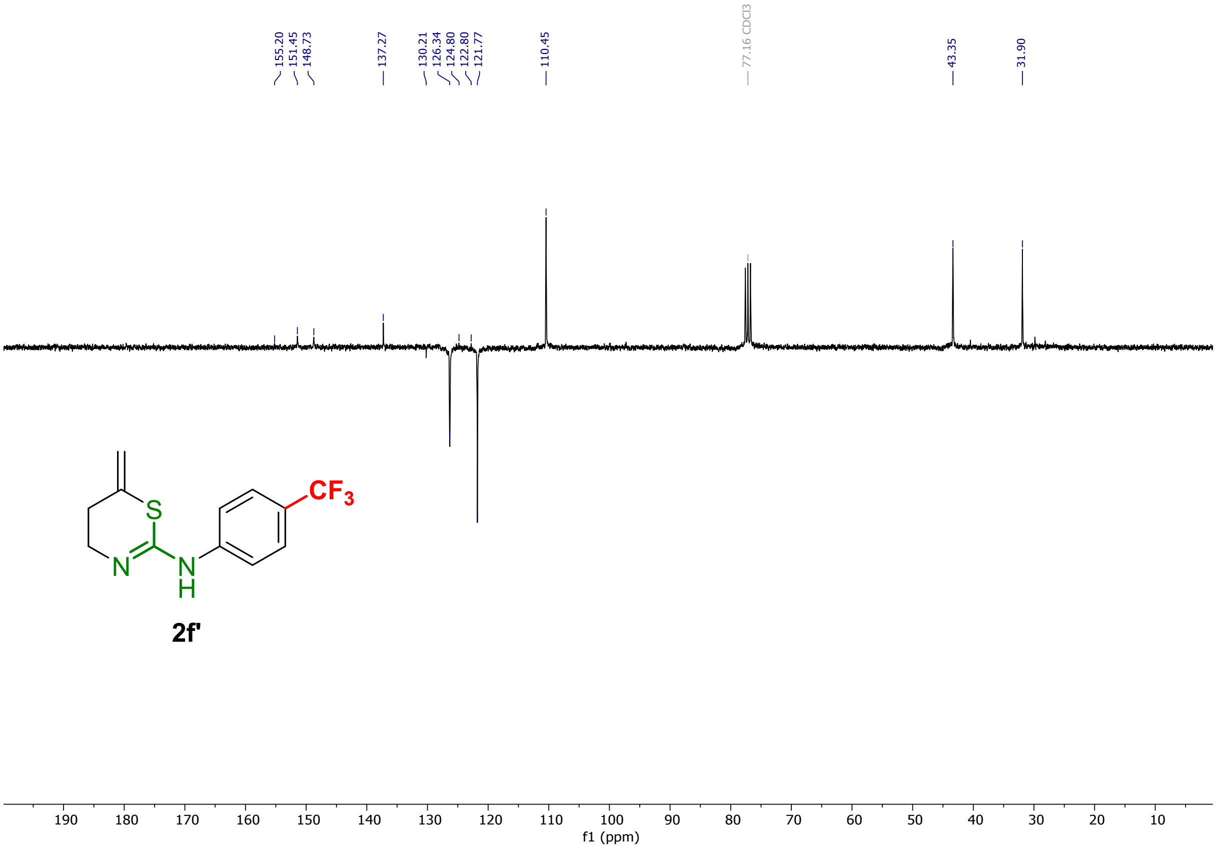

Figure S69. COSY NMR (300 MHz, $CDCl_3$) spectrum of 1,3-thiazine **2f'**

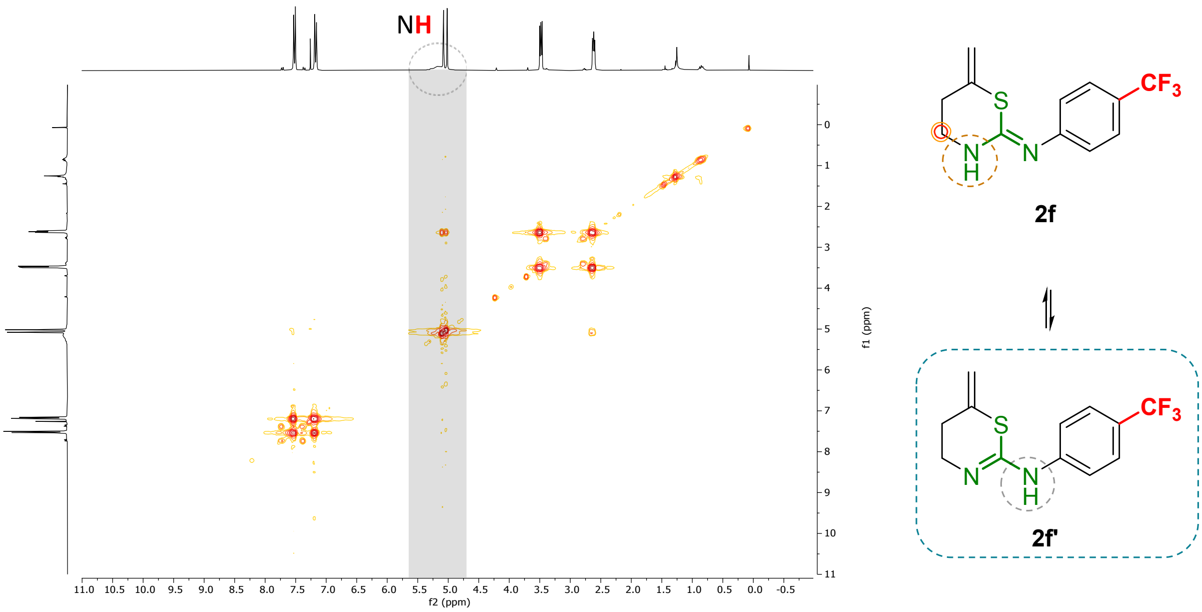

Figure S70. HSQC NMR (300 MHz, 75 MHz, $CDCl_3$) spectrum of 1,3-thiazine **2f'**

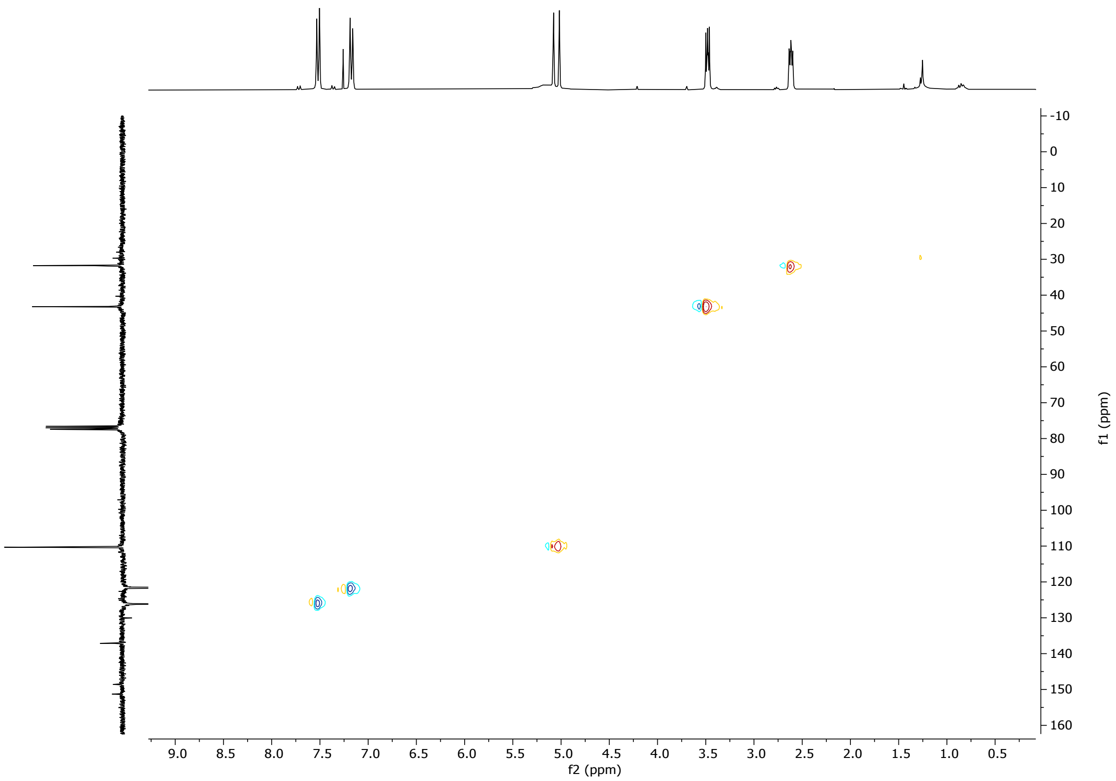

Figure S71. ^{1}H NMR (300 MHz, $CDCl_3$) spectrum of *N*-(3,5-bis(trifluoromethyl)phenyl)-6-methylene-5,6-dihydro-4*H*-1,3-thiazin-2-amine (**2g'**)

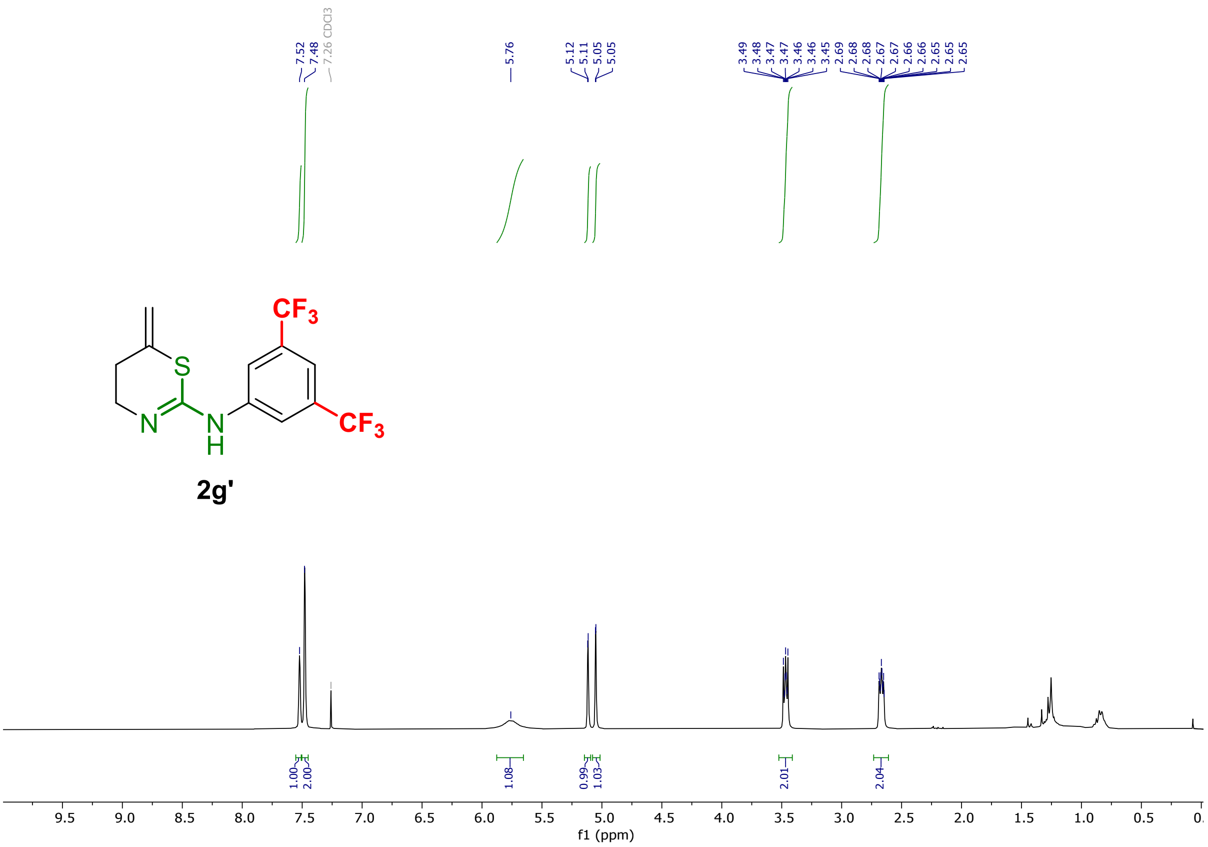

Figure S72. $^{13}C\{^{1}H\}$-APT NMR (75 MHz, $CDCl_3$) spectrum of 1,3-thiazine **2g'**

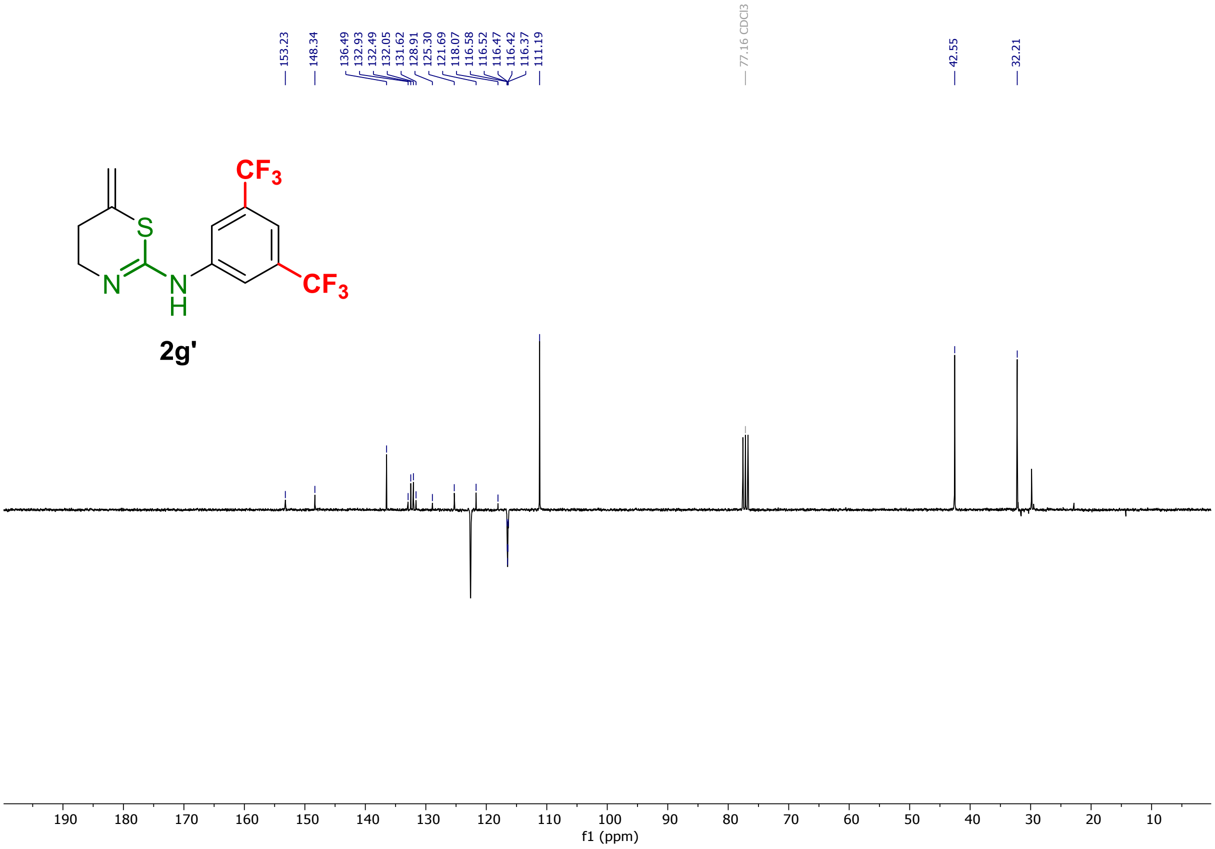

Figure S73. COSY NMR (300 MHz, $CDCl_3$) spectrum of 1,3-thiazine **2g'**

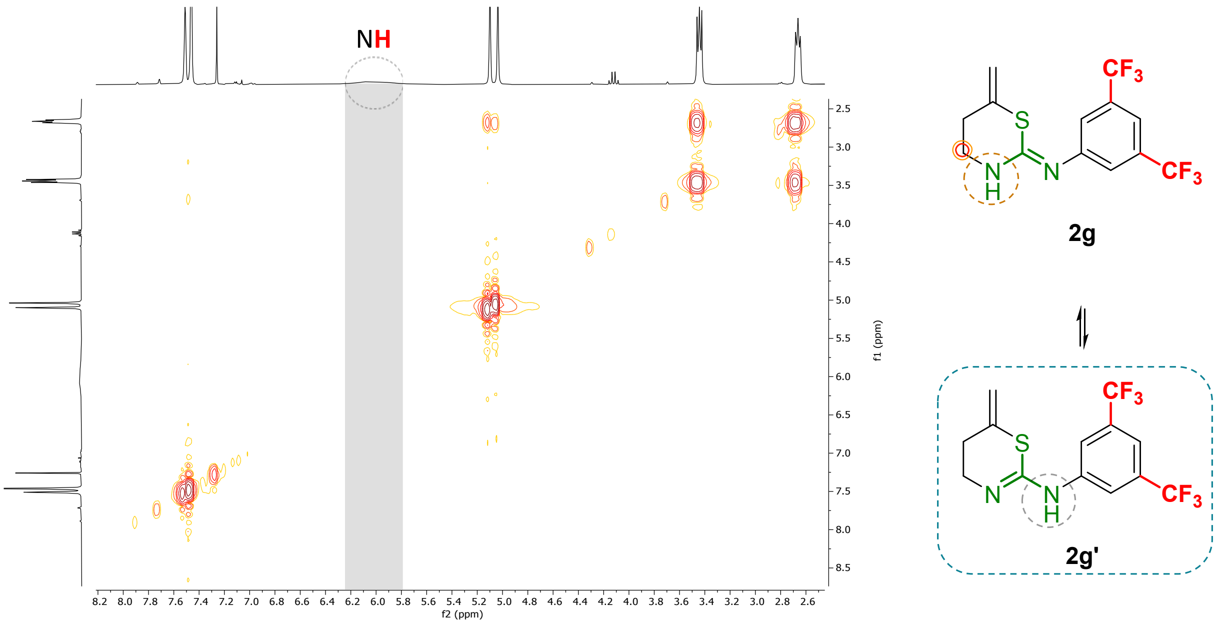

Figure S74. HSQC NMR (300 MHz, 75 MHz, $CDCl_3$) spectrum of 1,3-thiazine **2g'**

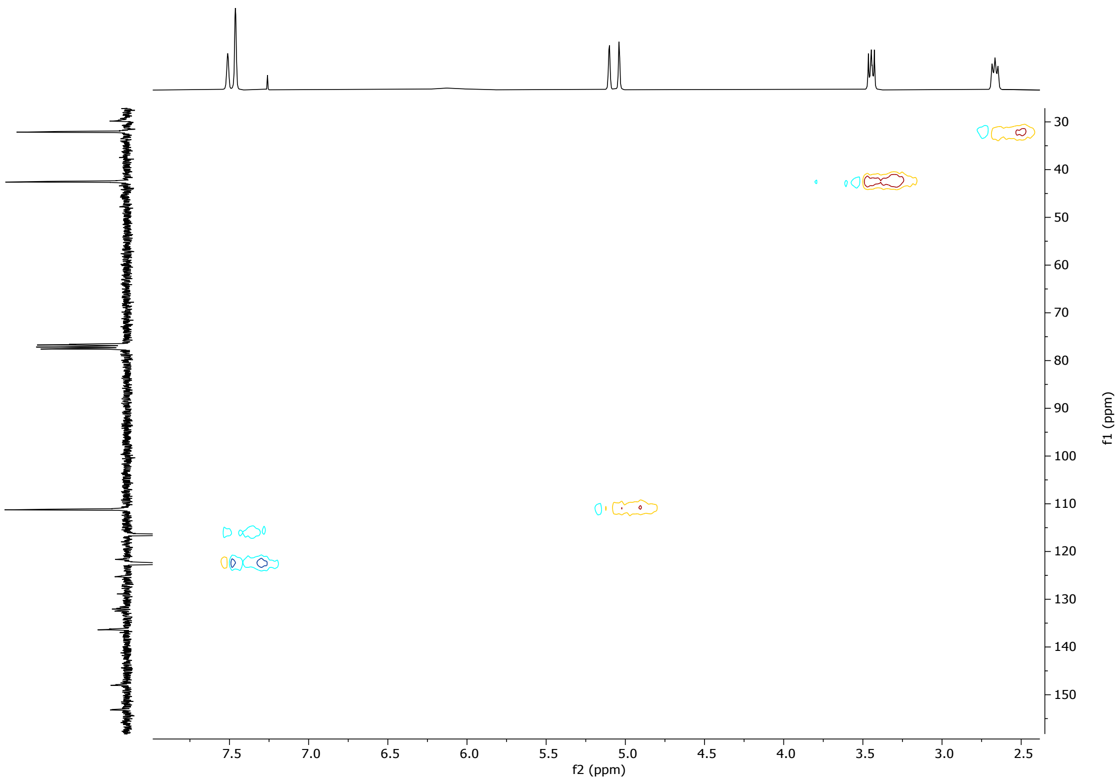

Figure S75. ^{1}H NMR (300 MHz, CD_3COCD_3) spectrum of 6-methylene-*N*-(4-nitrophenyl)-5,6-dihydro-4*H*-1,3-thiazin-2-amine (**2h'**)

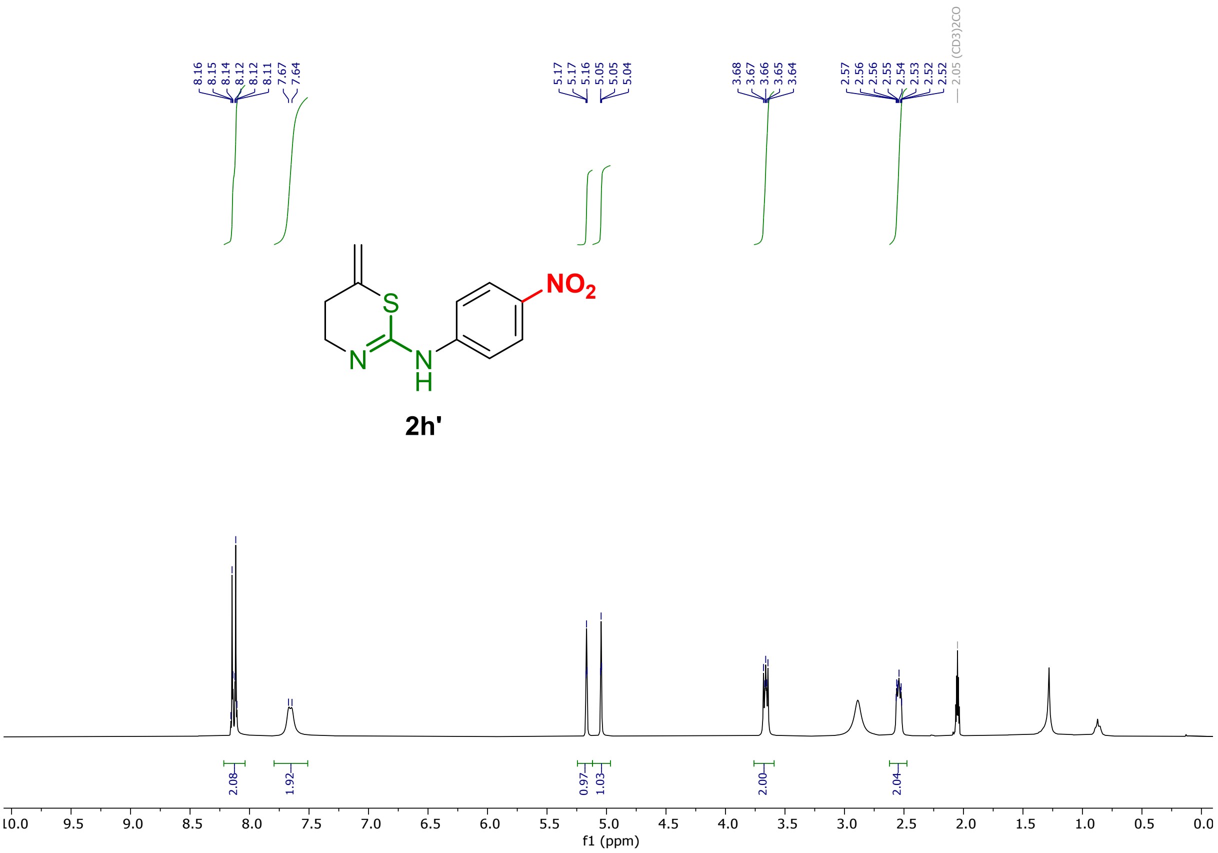

Figure S76. $^{13}C\{^{1}H\}$-APT NMR (75 MHz, CD_3COCD_3) spectrum of 1,3-thiazine **2h'**

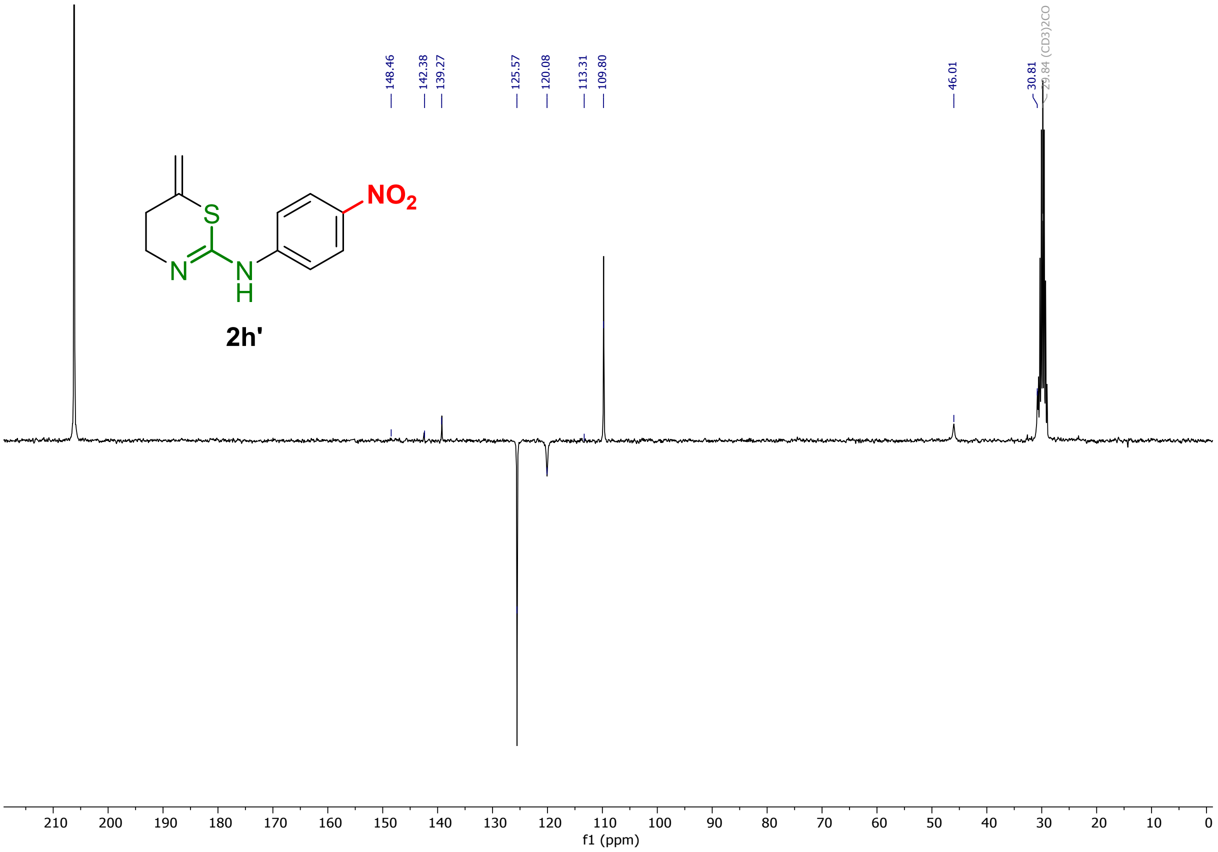

Figure S77. HMBC NMR (300 MHz, 75 MHz, CD_3COCD_3) spectrum of 1,3-thiazine **2h'**

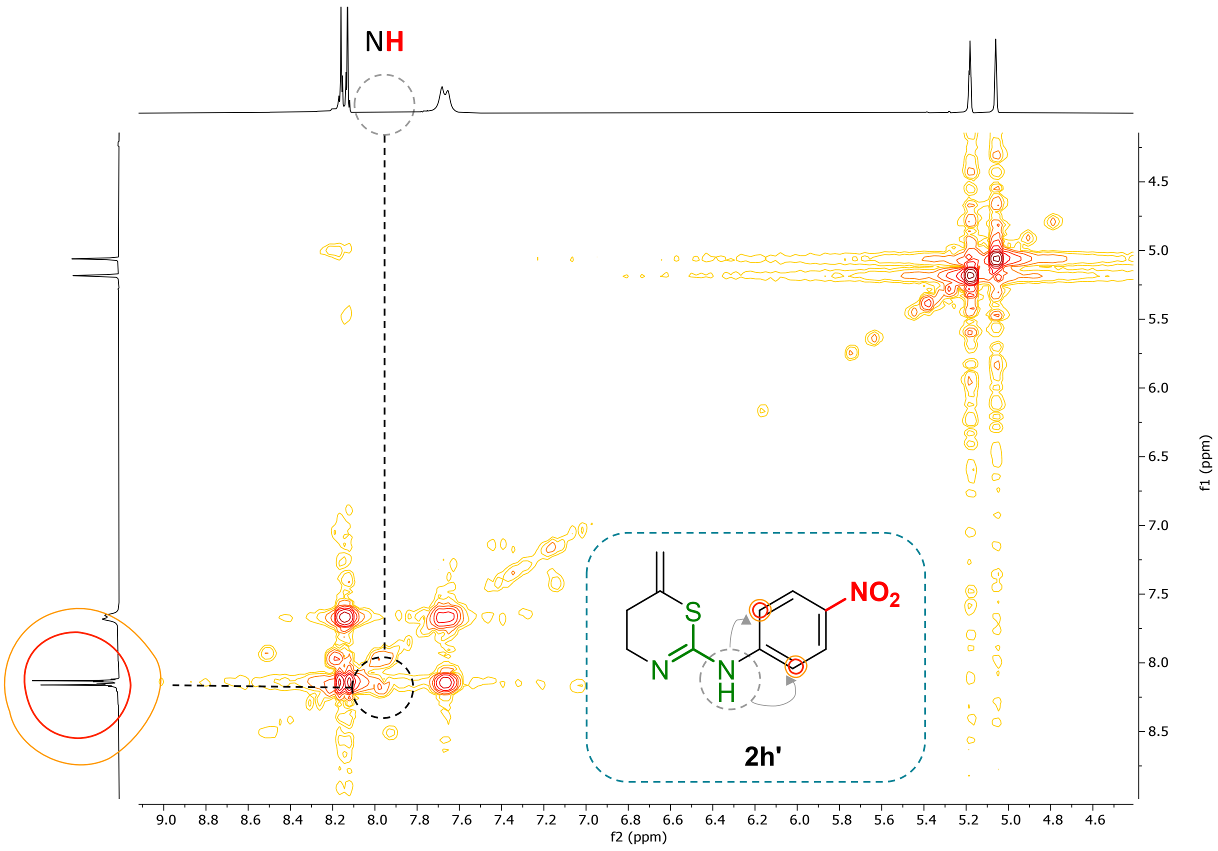

Figure S78. ^{1}H NMR (400 MHz, CD_3COCD_3) spectrum of 4-((6-methylene-5,6-dihydro-4*H*-1,3-thiazin-2-yl)amino)benzonitrile (**2i'**)

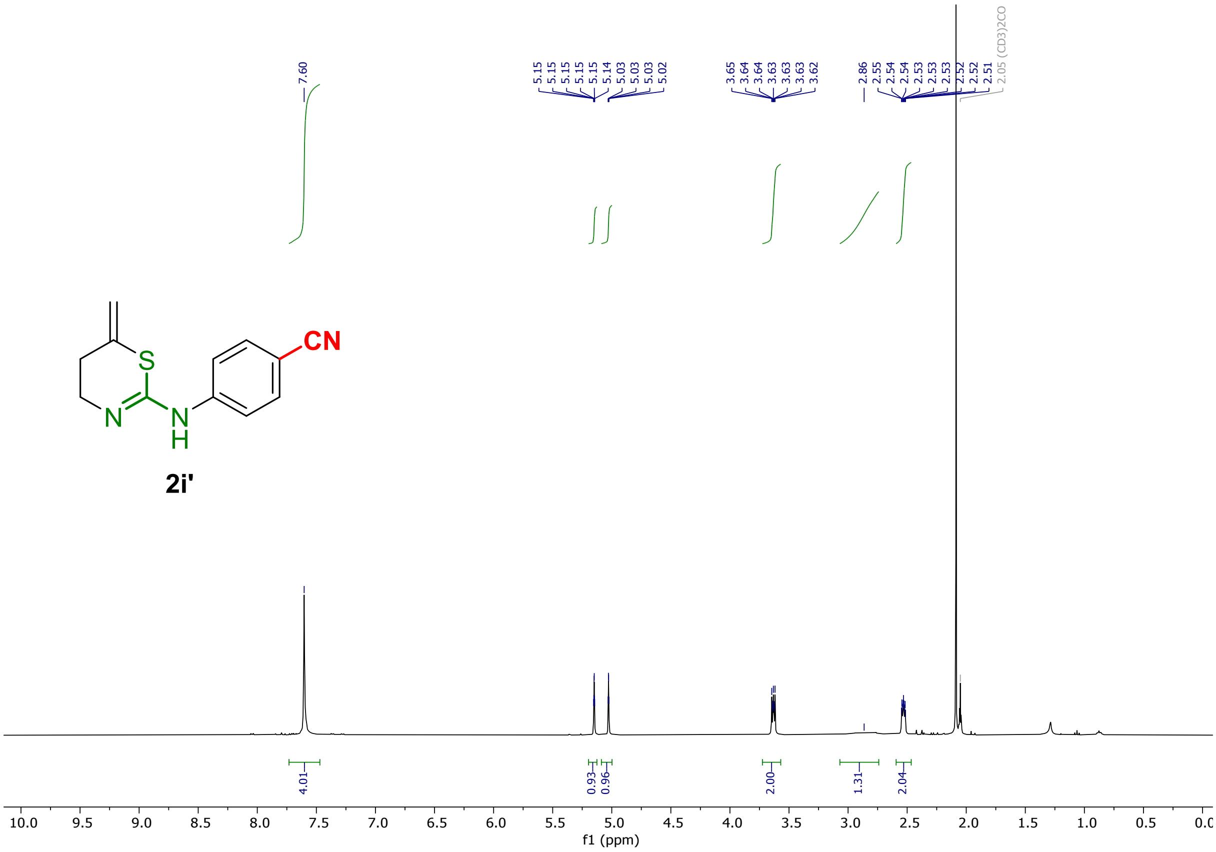

Figure S79. $^{13}C\{^1H\}$-APT NMR (101 MHz, CD_3COCD_3) spectrum of 1,3-thiazine **2i'**

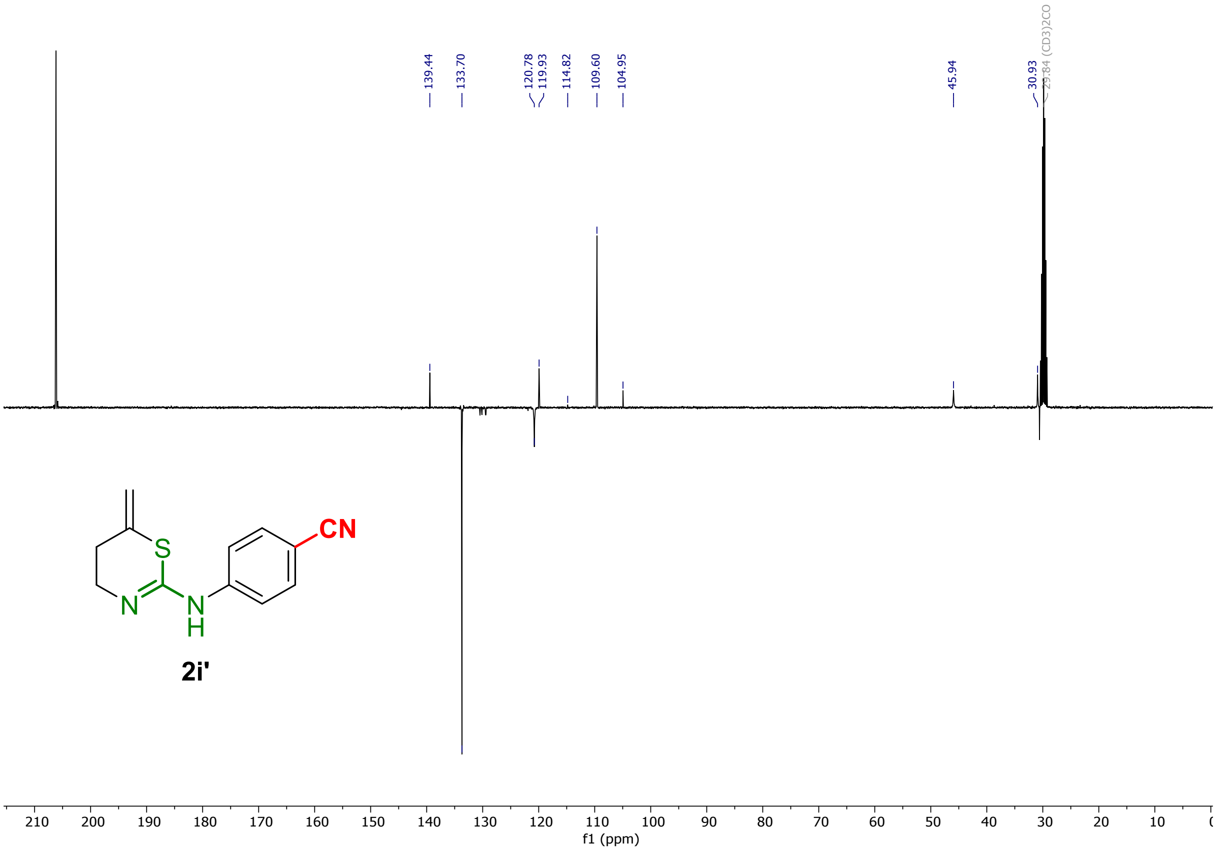

Figure S80. COSY NMR (300 MHz, $CDCl_3$) spectrum of 1,3-thiazine **2i'**

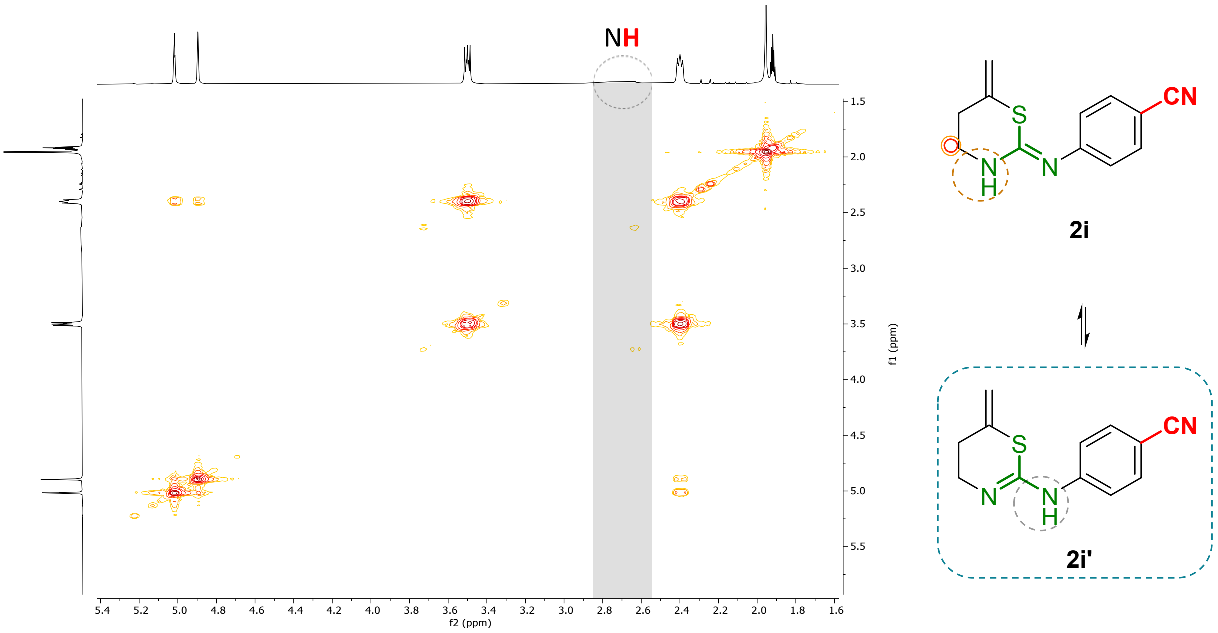

Figure S81. ^1H NMR (300 MHz, $CDCl_3$) spectrum of 6-methylene-*N*-(pyridin-3-yl)-5,6-dihydro-4*H*-1,3-thiazin-2-amine (**2j'**)

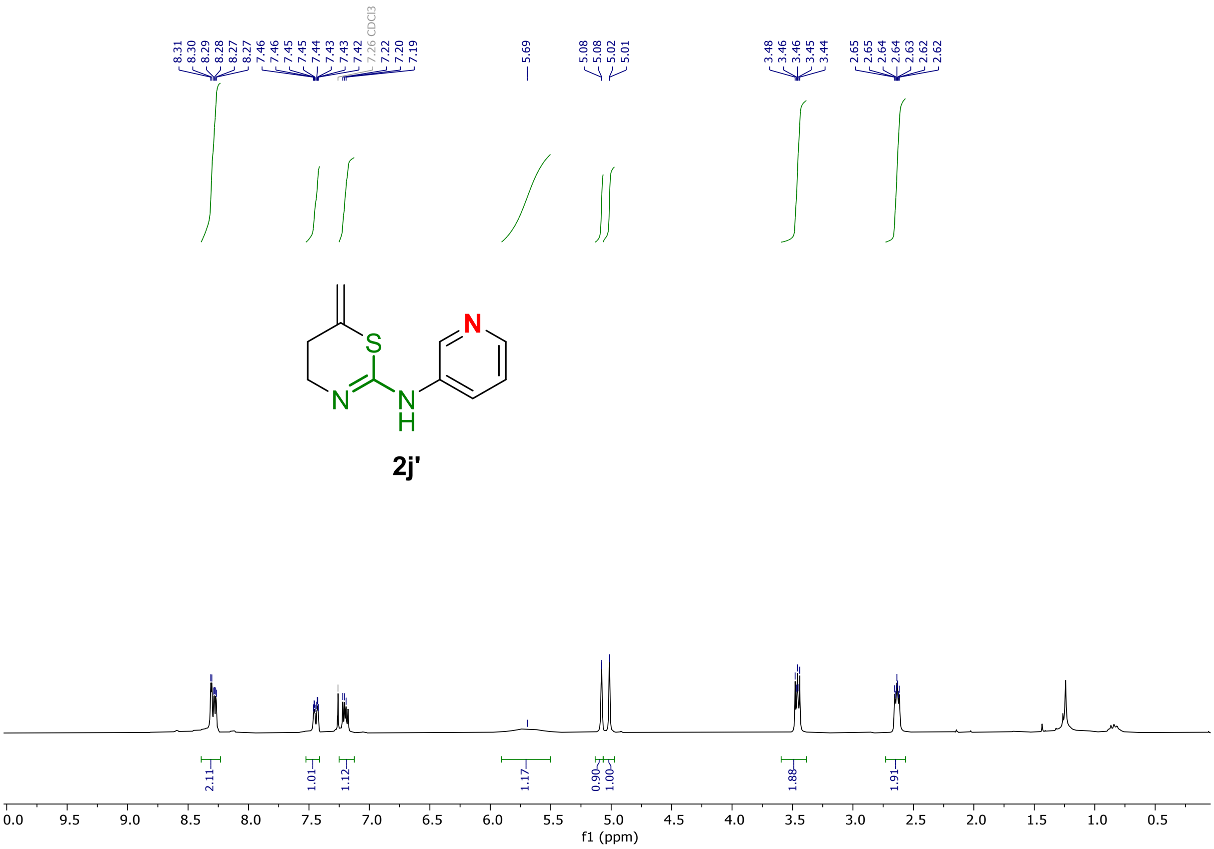

Figure S82. $^{13}C\{^{1}H\}$-APT NMR (75 MHz, $CDCl_3$) spectrum of 1,3-thiazine **2j'**

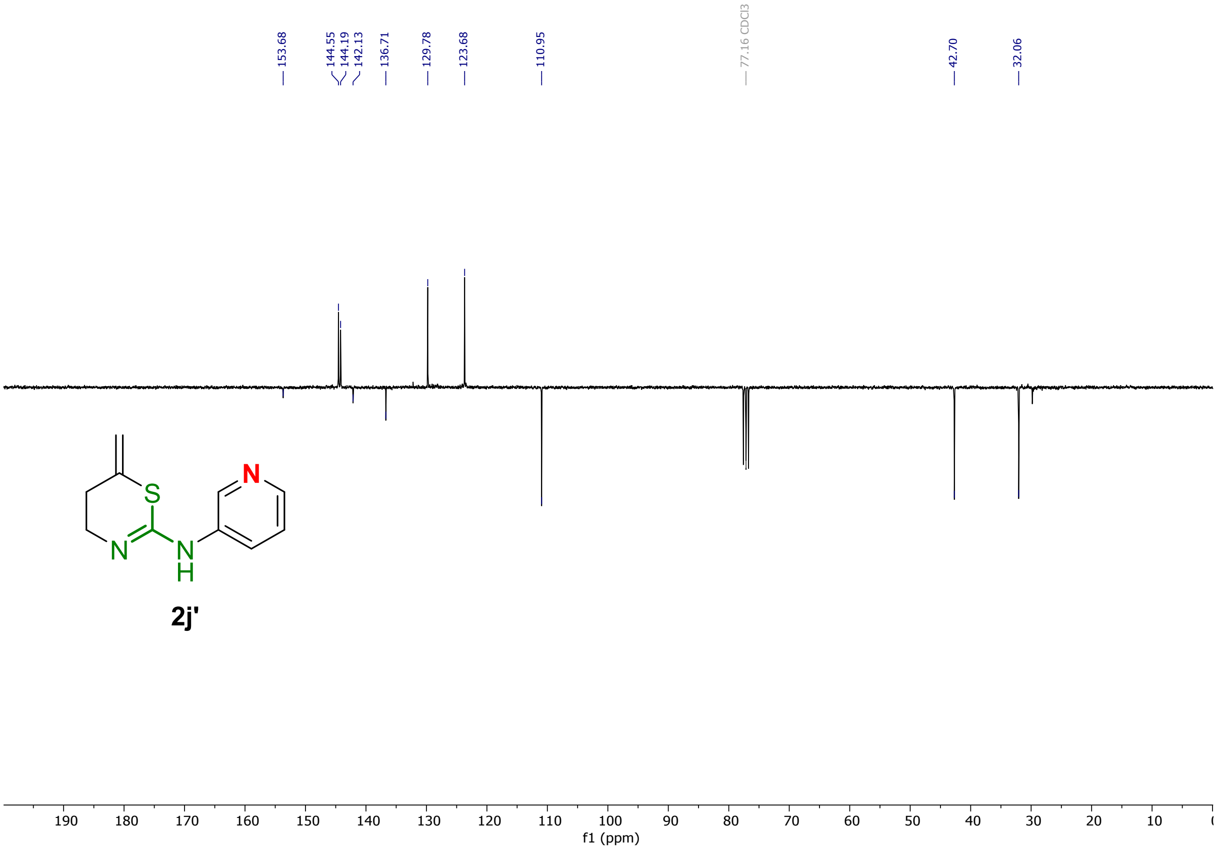

Figure S83. COSY NMR (300 MHz, $CDCl_3$) spectrum of 1,3-thiazine **2j'**

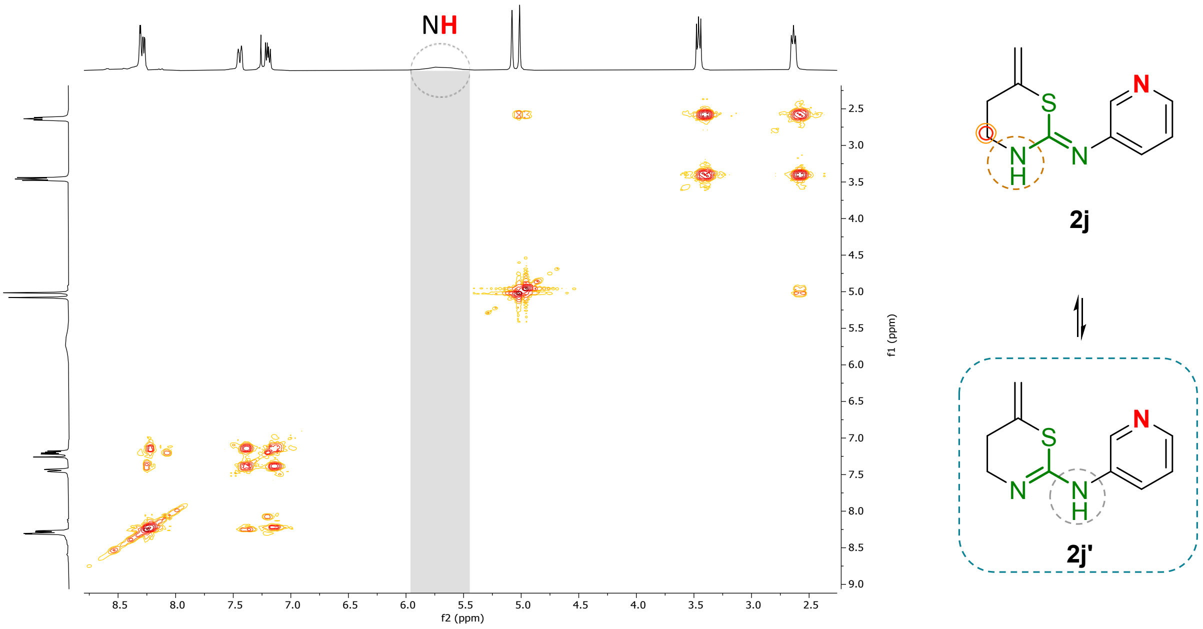

Figure S84. HSQC NMR (300 MHz, 75 MHz, $CDCl_3$) spectrum of 1,3-thiazine **2j'**

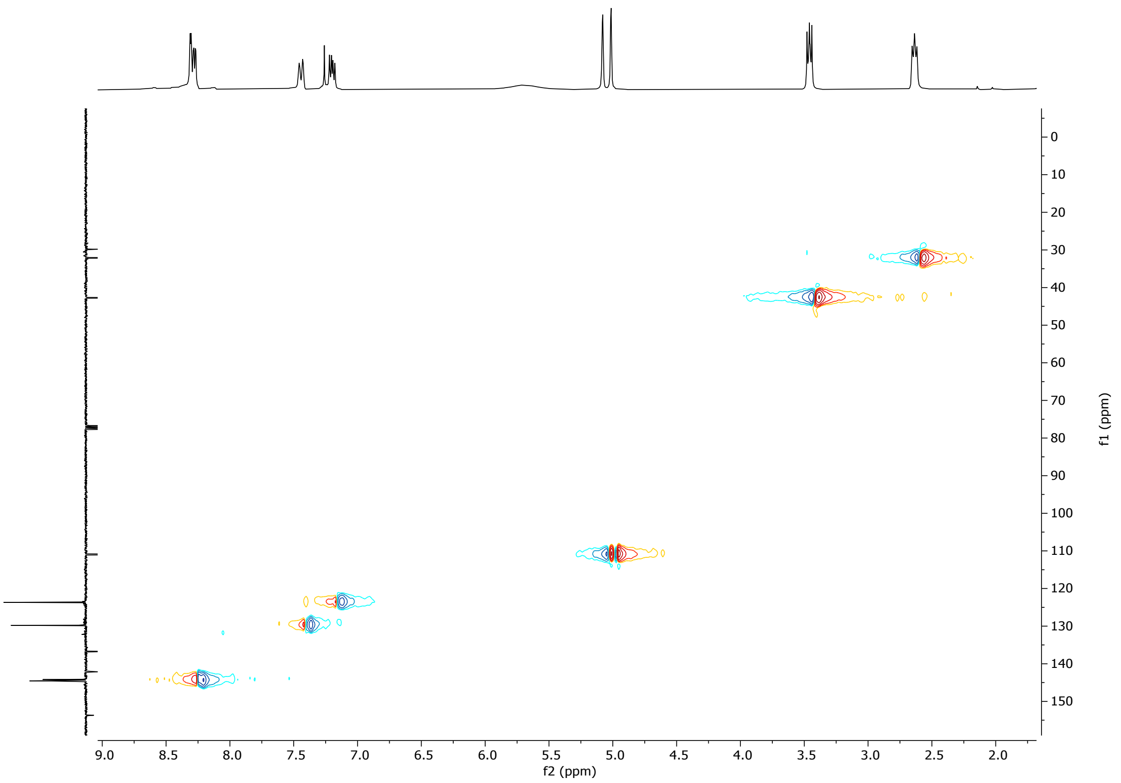

Figure S85. ^{1}H NMR (300 MHz, $CDCl_3$) spectrum of 6-methylene-*N*-(4-methylphenyl)-5,6-dihydro-4*H*-1,3-thiazin-2-amine (**2k'**)

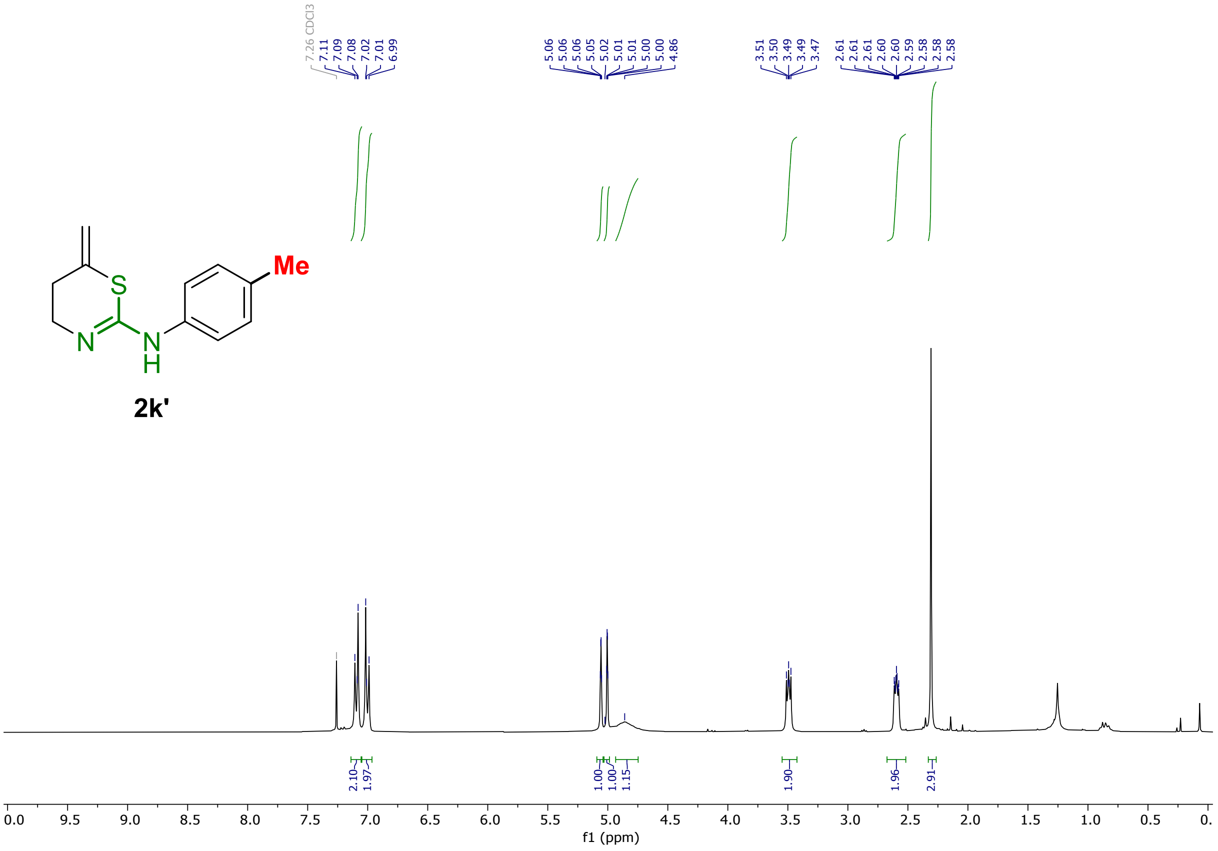

Figure S86. $^{13}C\{^{1}H\}$-APT NMR (75 MHz, $CDCl_3$) spectrum of 1,3-thiazine **2k'**

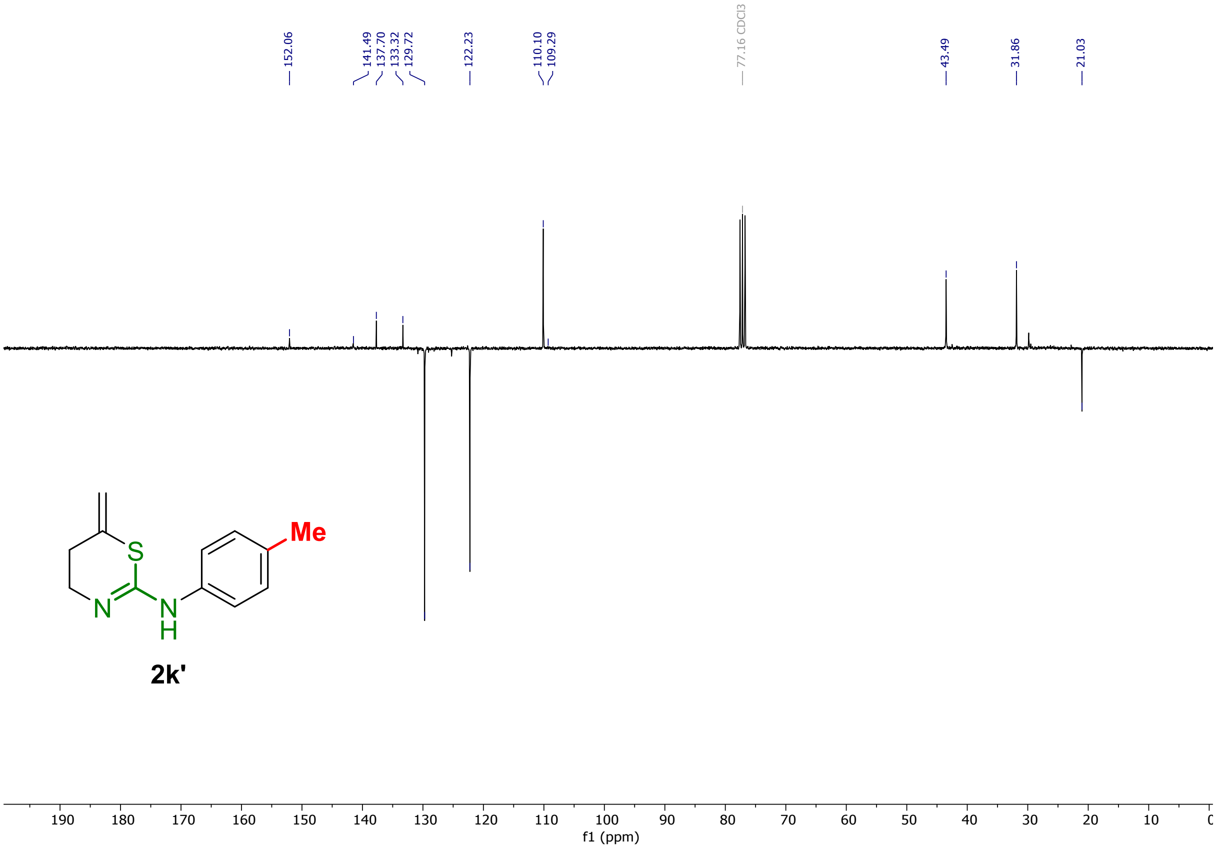

Figure S87. COSY NMR (300 MHz, $CDCl_3$) spectrum of 1,3-thiazine **2k'**

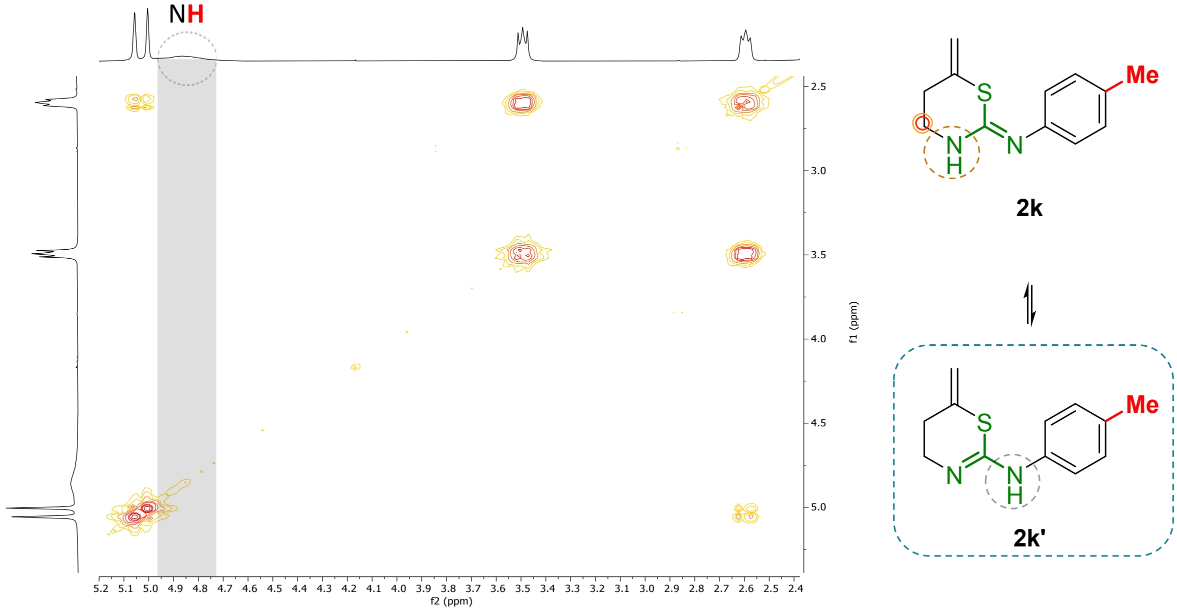

Figure S88. HSQC NMR (300 MHz, 75 MHz, $CDCl_3$) spectrum of 1,3-thiazine **2k'**

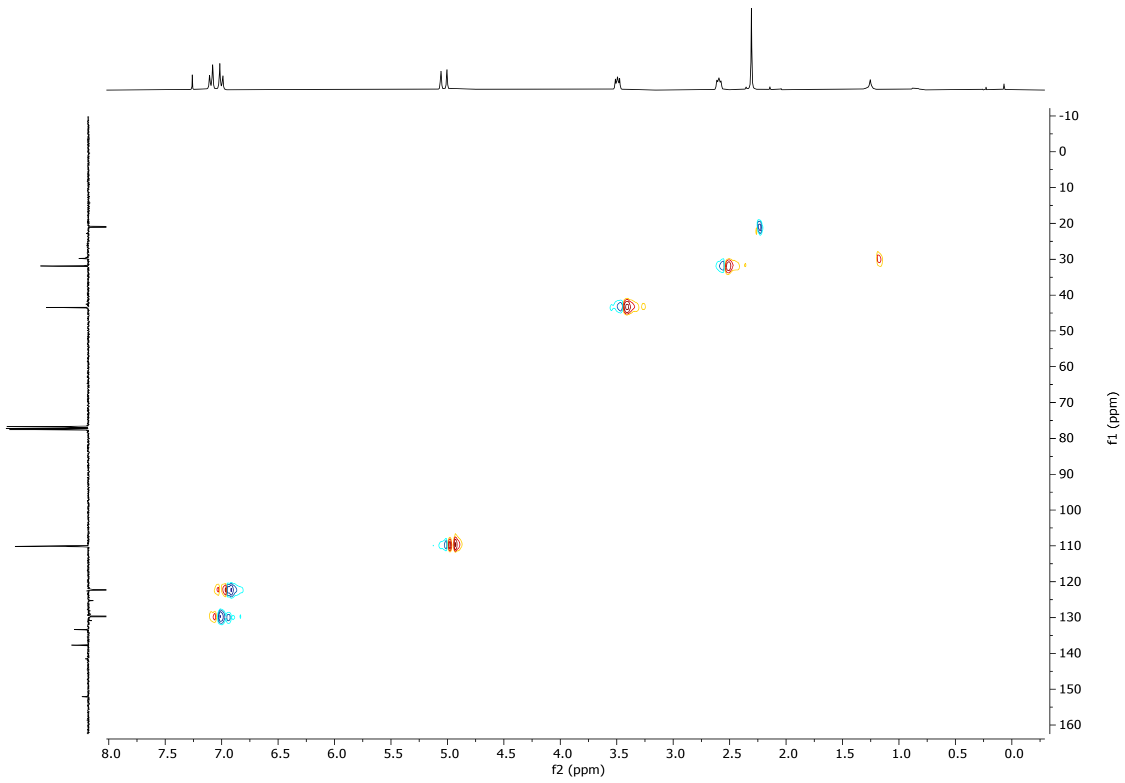

Figure S89. ^{1}H NMR (400 MHz, $CDCl_3$) spectrum of *N*-(4-methoxyphenyl)-6-methylene-5,6-dihydro-4*H*-1,3-thiazin-2-amine (**2l'**)

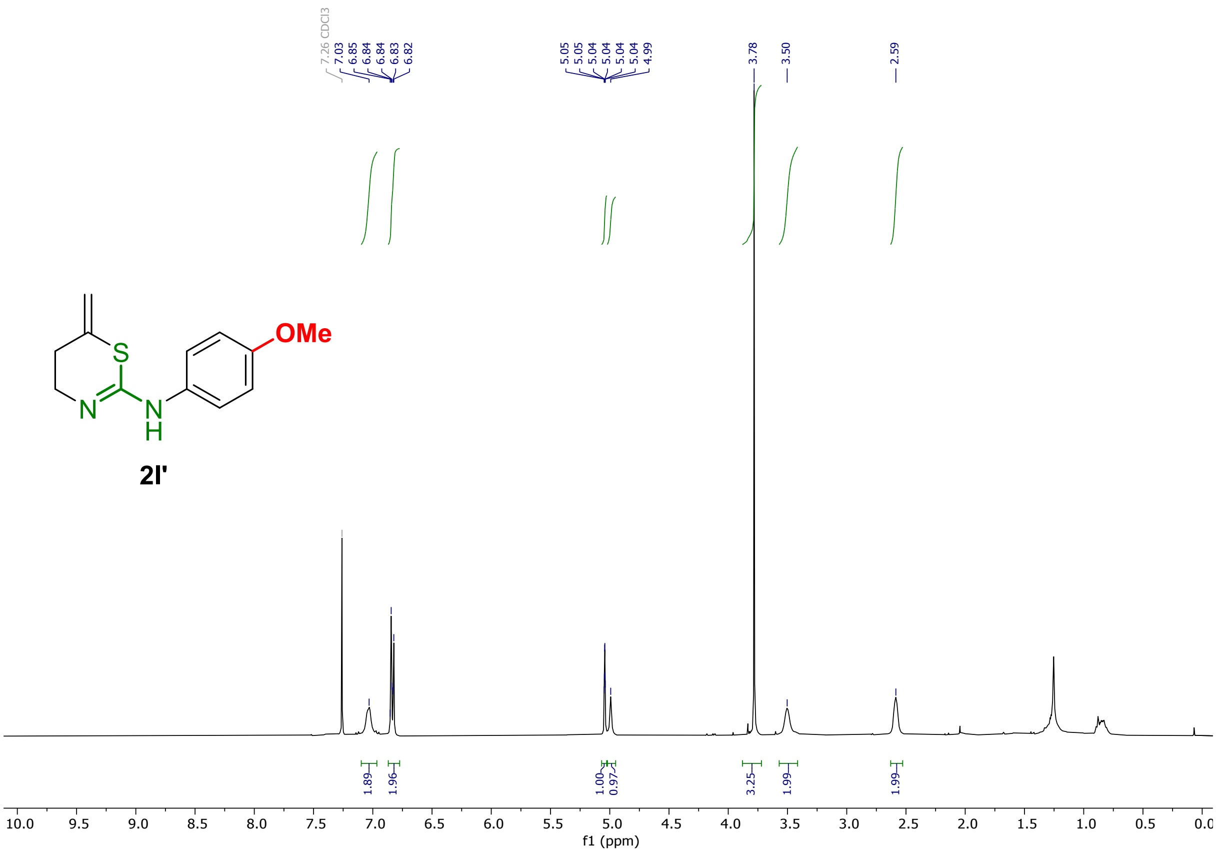

Figure S90. $^{13}C\{^{1}H\}$-APT NMR (101 MHz, $CDCl_3$) spectrum of 1,3-thiazine **2l'**

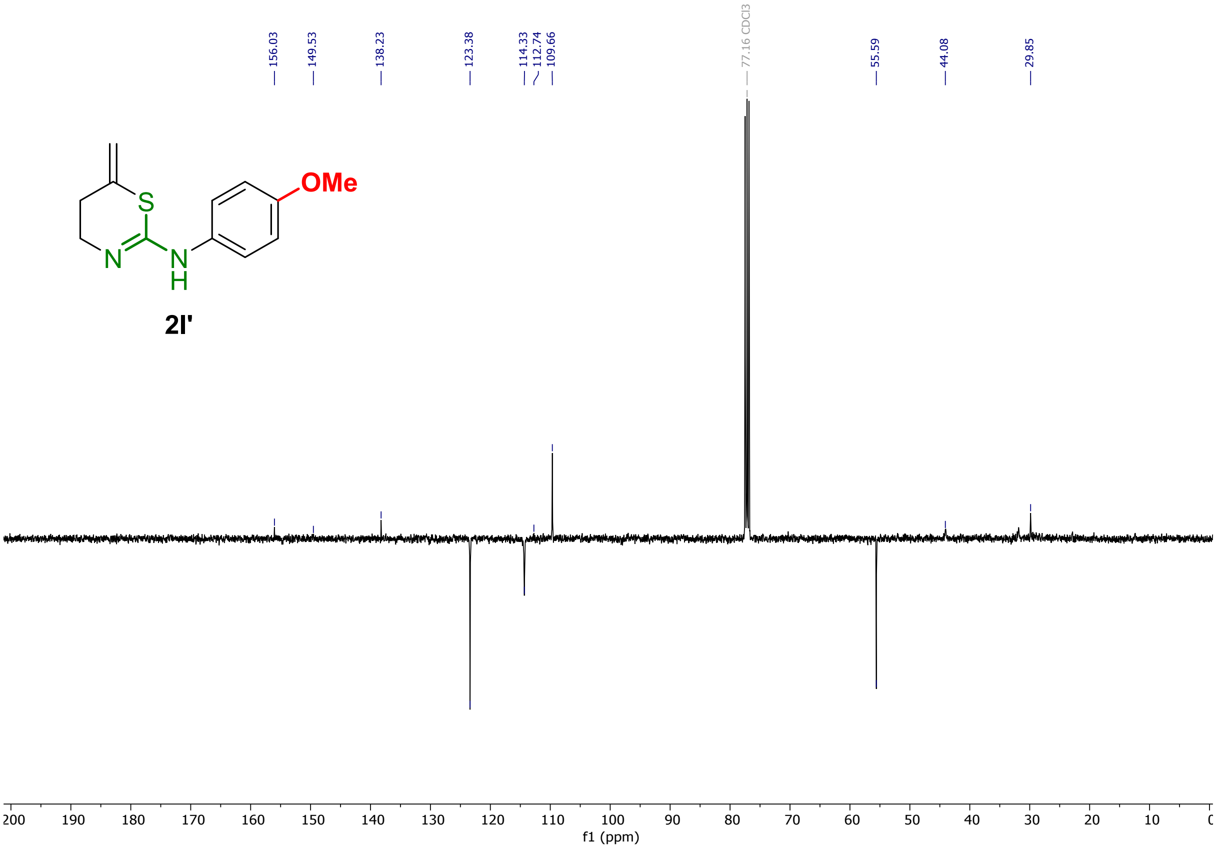

Figure S91. ^{1}H NMR (300 MHz, $CDCl_3$) spectrum of 6-methylene-*N*-(naphthalen-1-yl)-5,6-dihydro-4H-1,3-thiazin-2-amine (**2m'**)

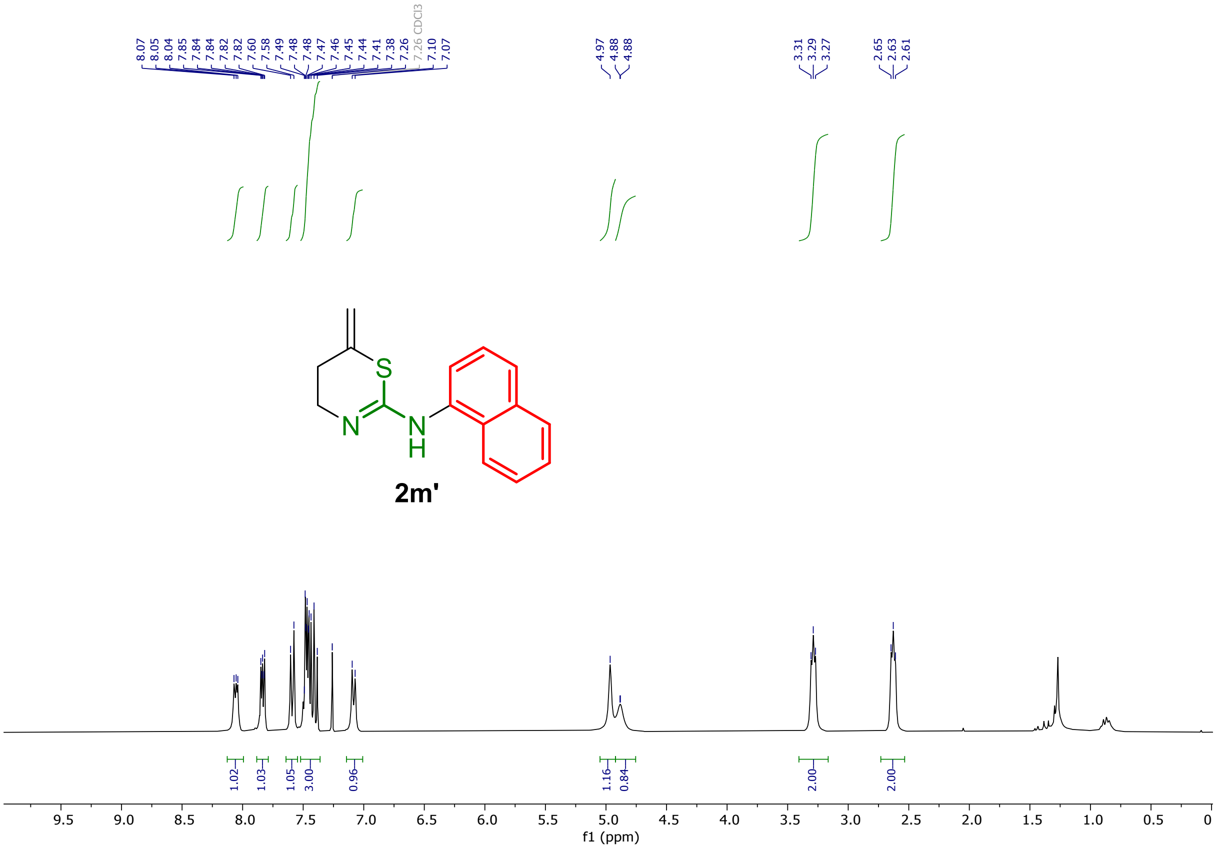

Figure S92. $^{13}C\{^1H\}$-APT NMR (101 MHz, $CDCl_3$) spectrum of 1,3-thiazine **2m'**

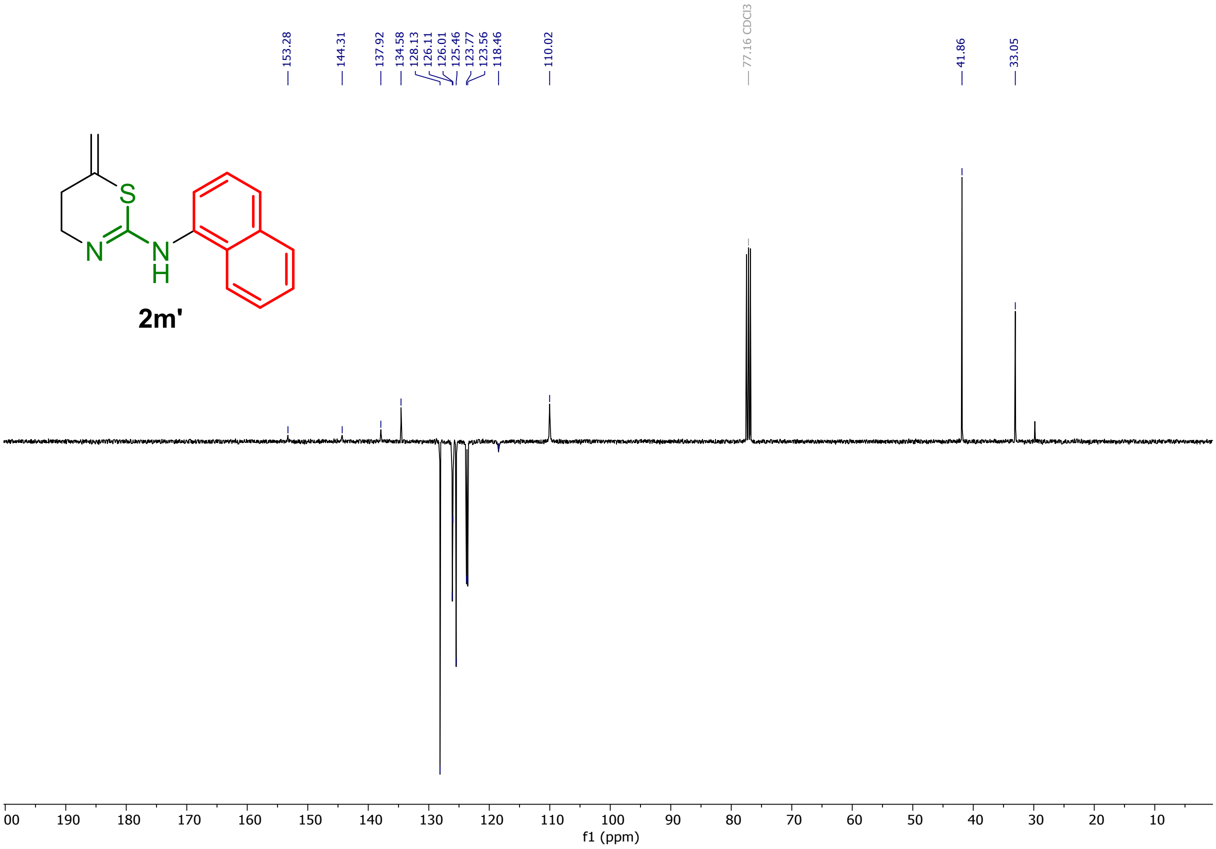

Figure S93. ^1H NMR (300 MHz, $CDCl_3$) spectrum of *N*-benzyl-6-methylene-5,6-dihydro-4*H*-1,3-thiazin-2-amine (**2n'**)

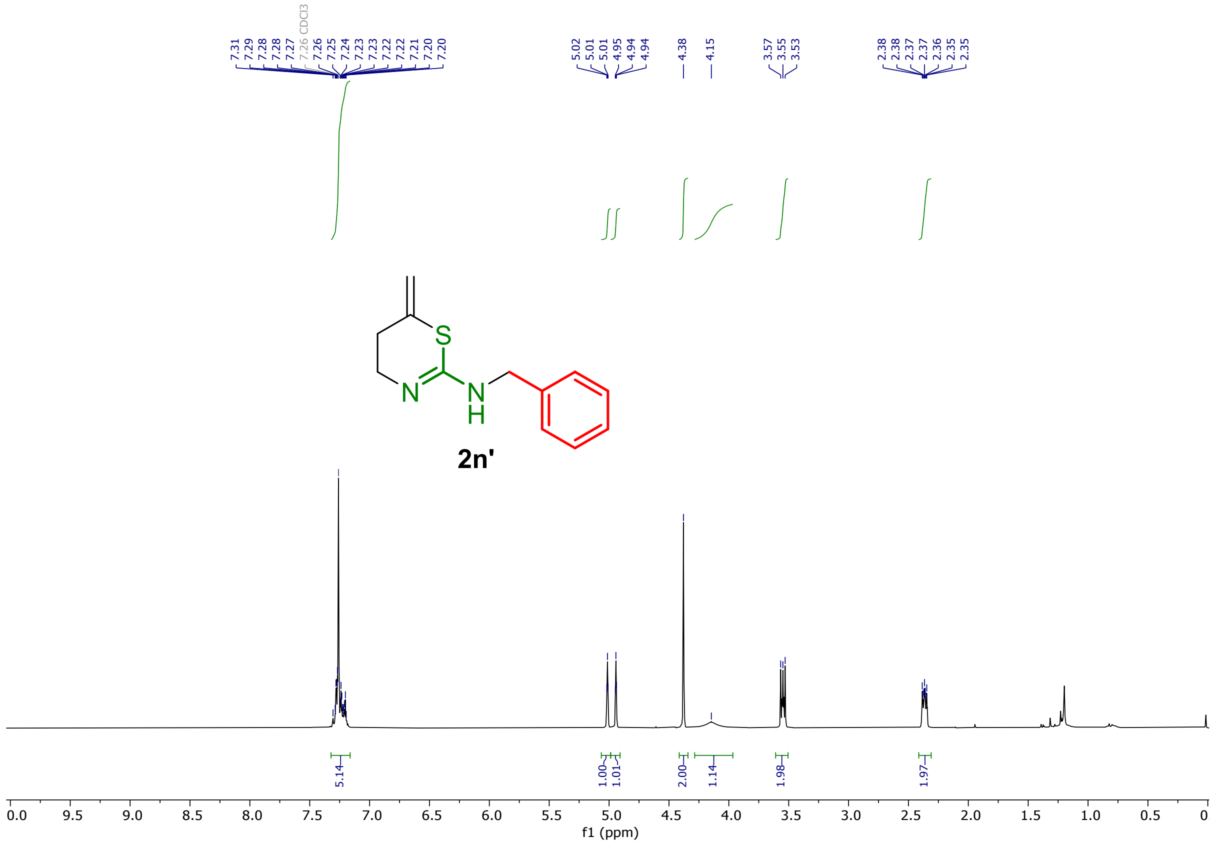

Figure S94. $^{13}C\{^{1}H\}$-APT NMR (75 MHz, $CDCl_3$) spectrum of 1,3-thiazine **2n'**

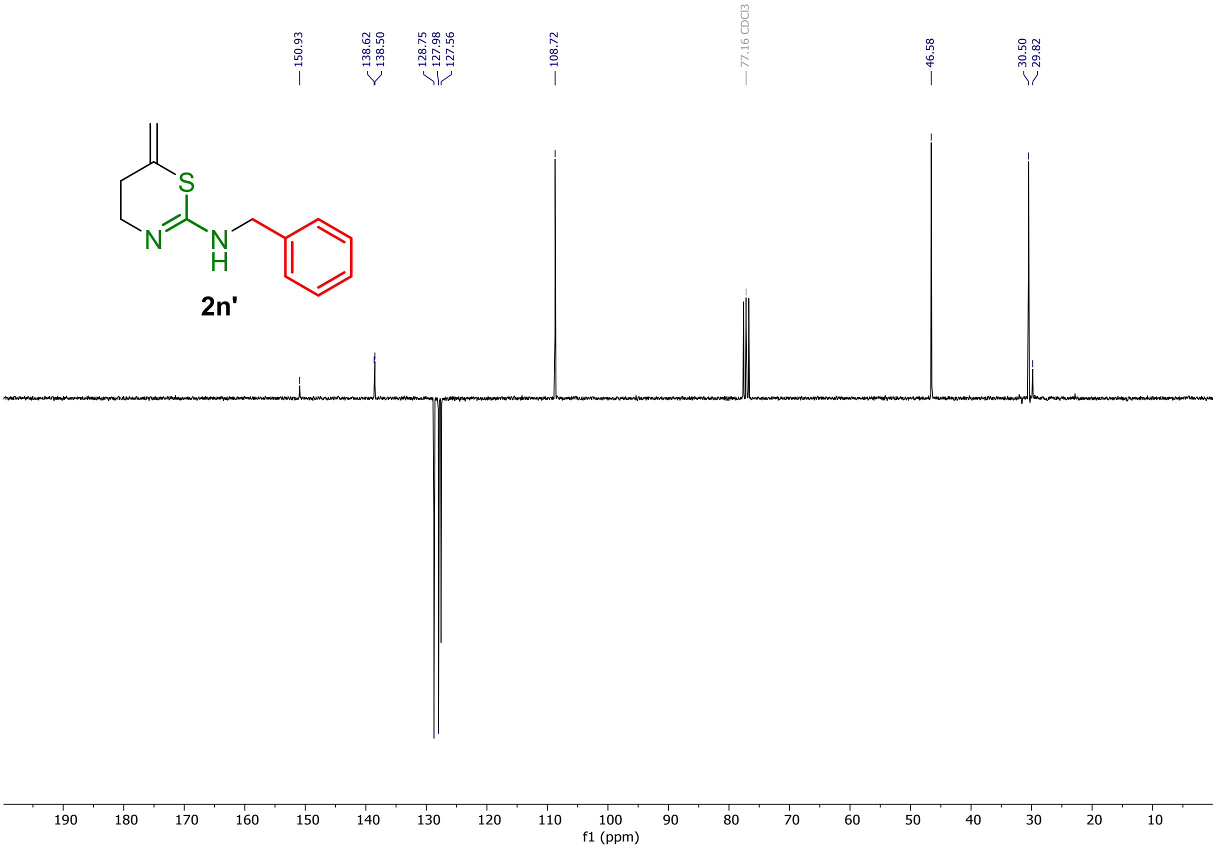

Figure S95. COSY NMR (300 MHz, $CDCl_3$) spectrum of 1,3-thiazine **2n'**

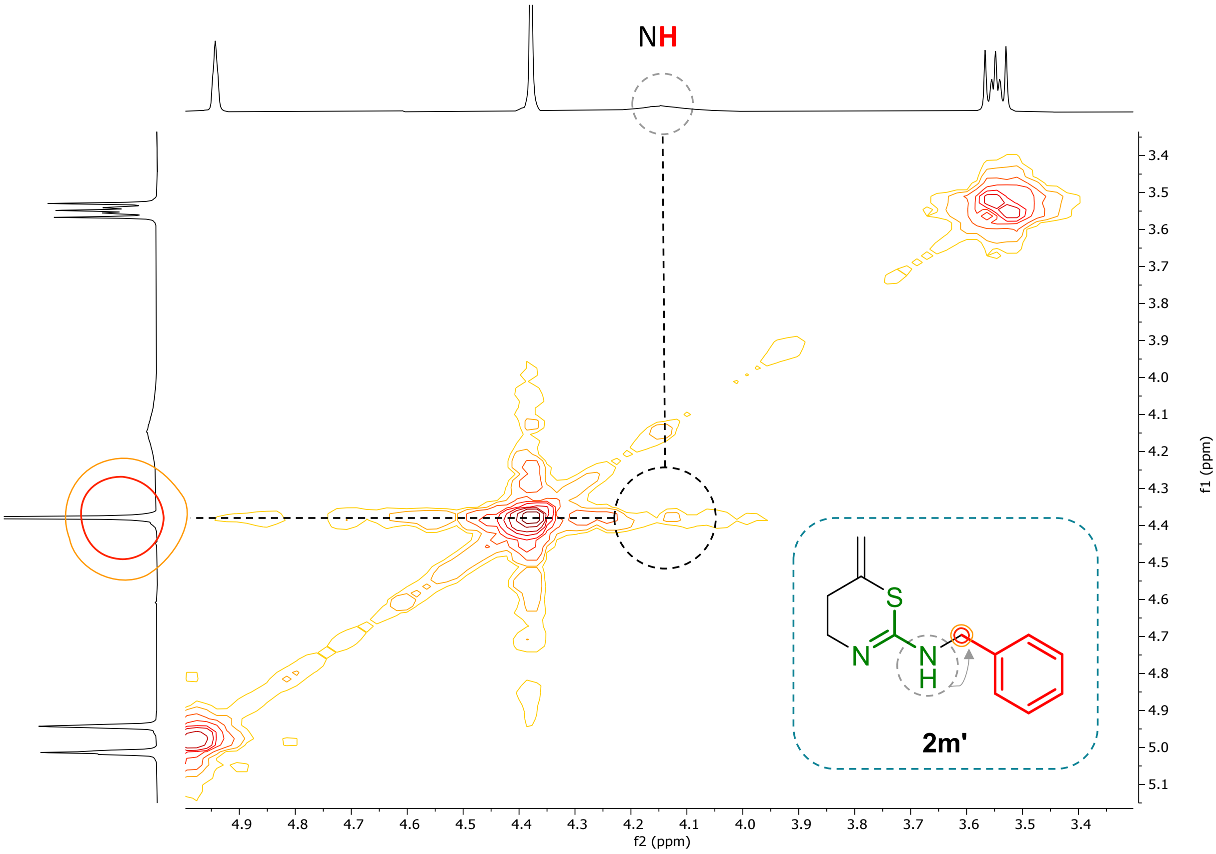

Figure S96. HSQC NMR (300 MHz, 75 MHz, $CDCl_3$) spectrum of 1,3-thiazine **2n'**

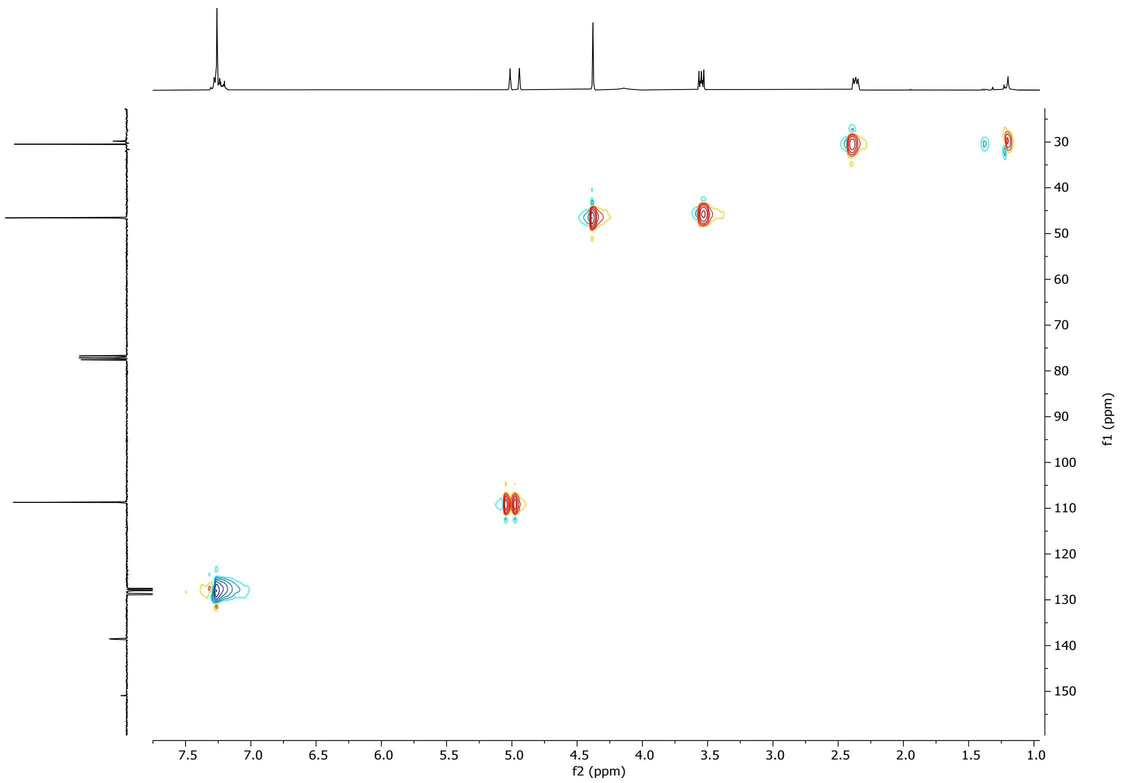

Figure S97. ^{1}H NMR (300 MHz, CD_3COCD_3) spectrum of 6-methylene-*N*-phenethyl-5,6-dihydro-4*H*-1,3-thiazin-2-amine (**2o'**)

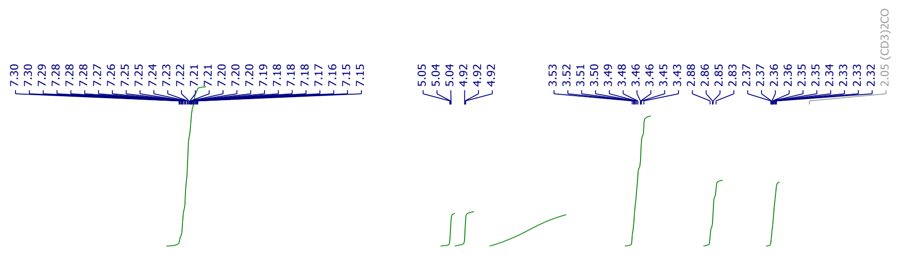

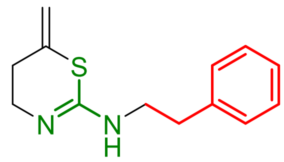

2o'

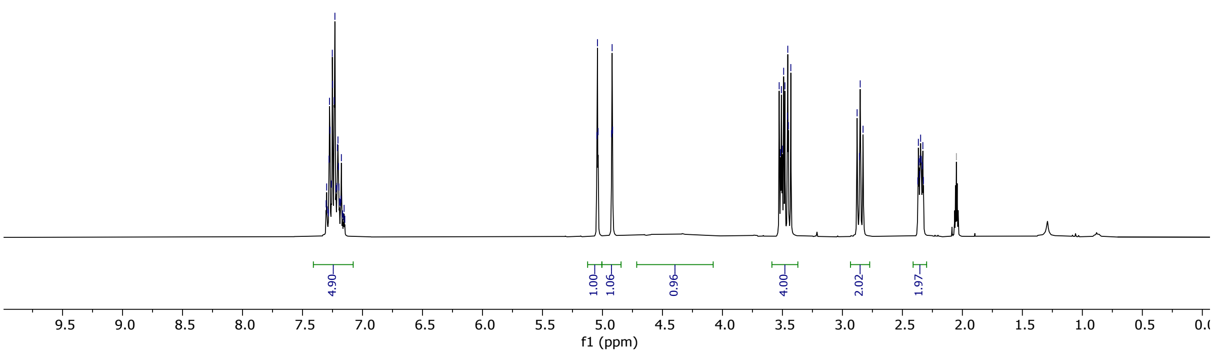

Figure S98. $^{13}C\{^1H\}$-APT NMR (75 MHz, CD_3COCD_3) spectrum of 1,3-thiazine **2o'**

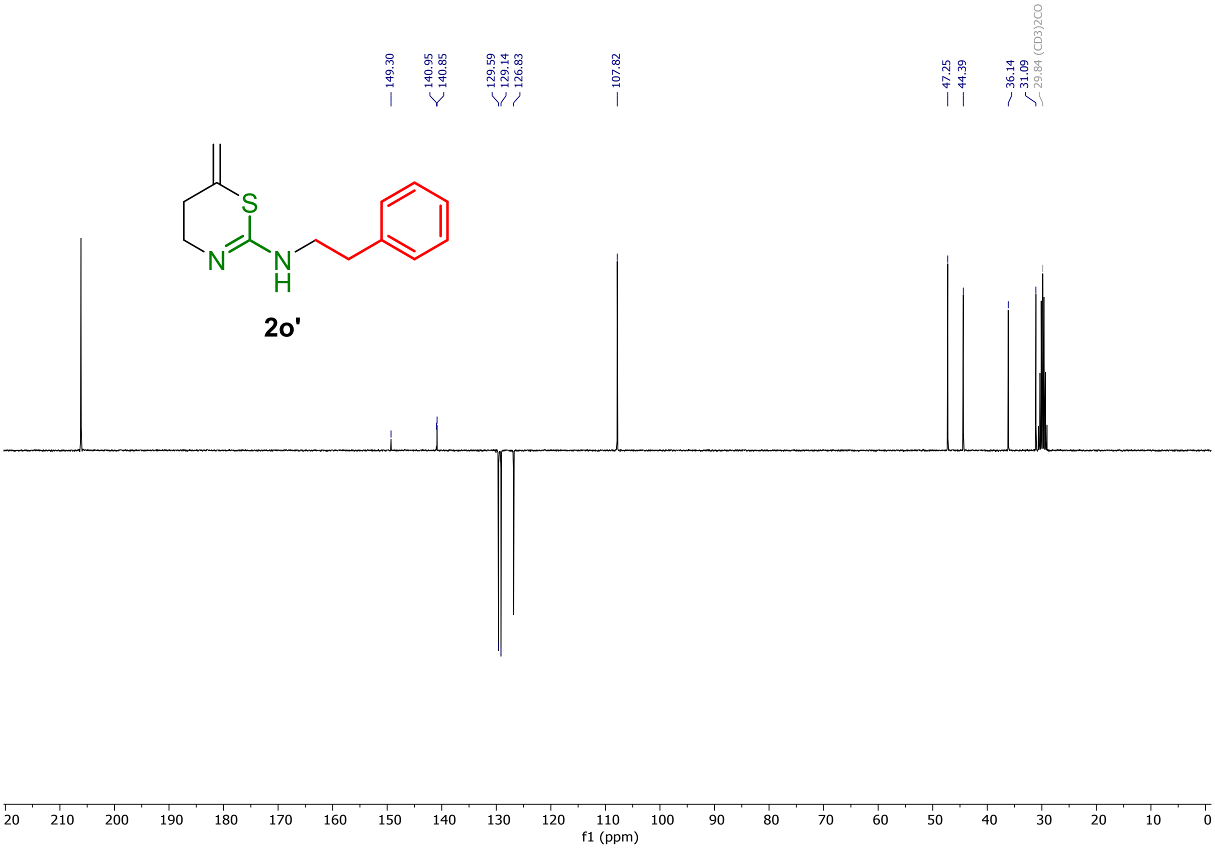

Figure S99. COSY NMR (300 MHz, CD_3COCD_3) spectrum of 1,3-thiazine **2o'**

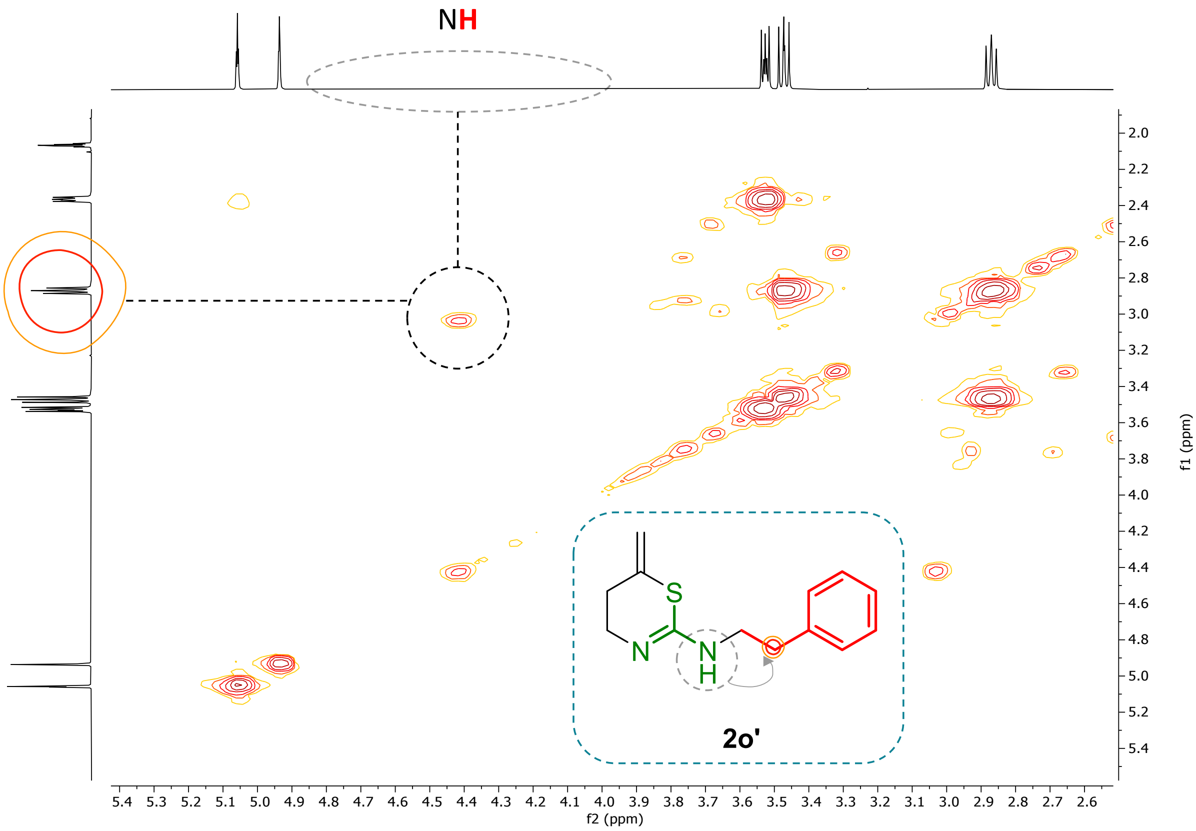

Figure S100. ^{1}H NMR (400 MHz, CD_3COCD_3) spectrum of *N*-cyclohexyl-6-methylene-5,6-dihydro-4*H*-1,3-thiazin-2-amine (**2p’**)

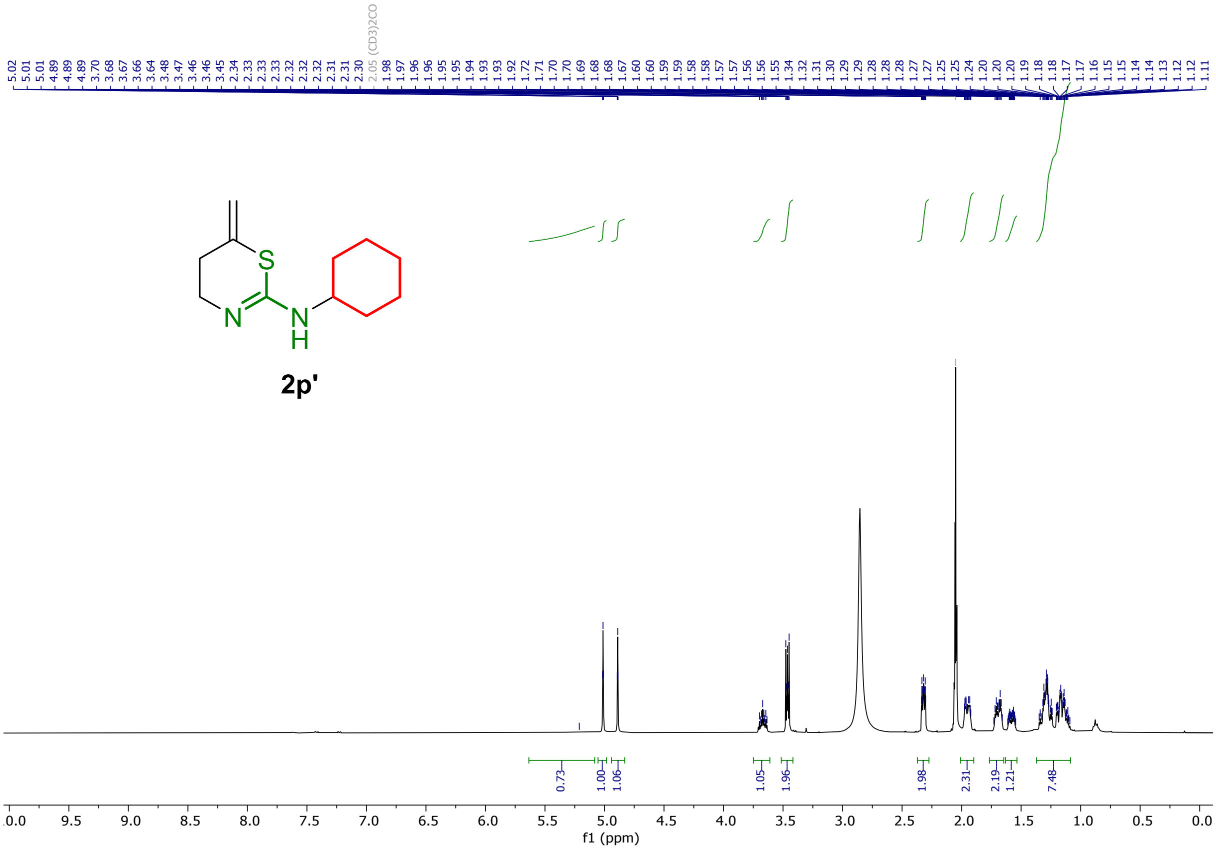

Figure S101. $^{13}C\{^{1}H\}$-APT NMR (101 MHz, CD_3COCD_3) spectrum of 1,3-thiazine **2p’**

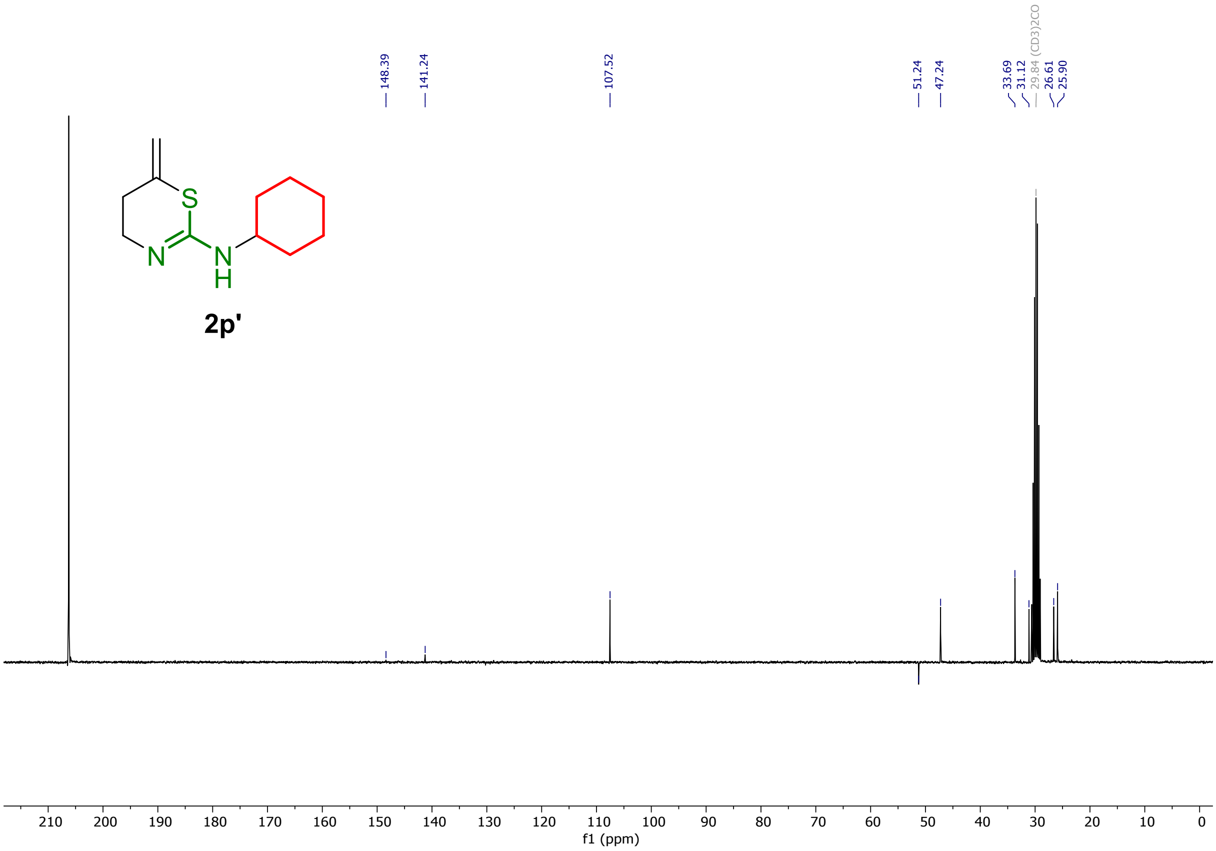

Figure S102. ^{1}H NMR (300 MHz, CD_3COCD_3) spectrum of *N*-cyclohexyl-*N*-methyl-6-methylene-5,6-dihydro-4*H*-1,3-thiazin-2-amine (**2r'**)

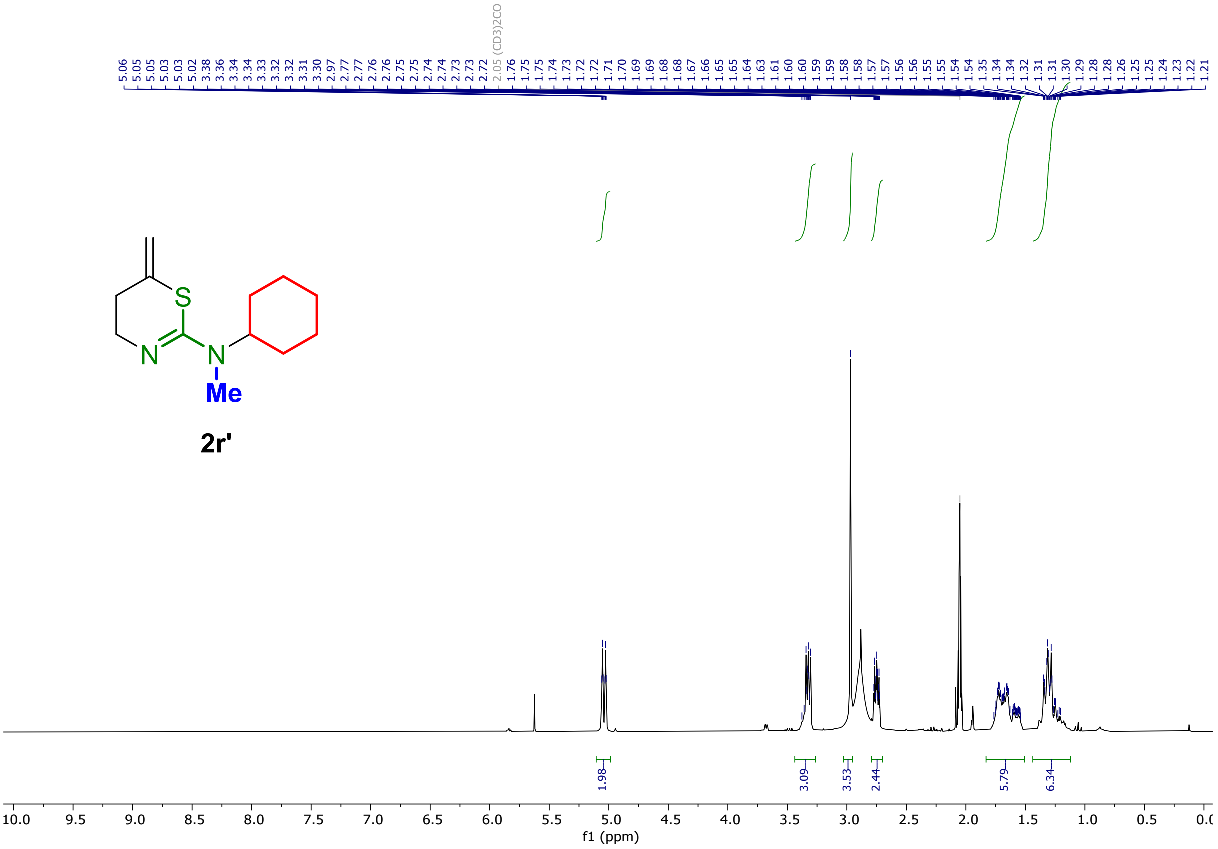

Figure S103. $^{13}C\{^{1}H\}$-APT NMR (75 MHz, CD_3COCD_3) spectrum of 1,3-thiazine **2r'**

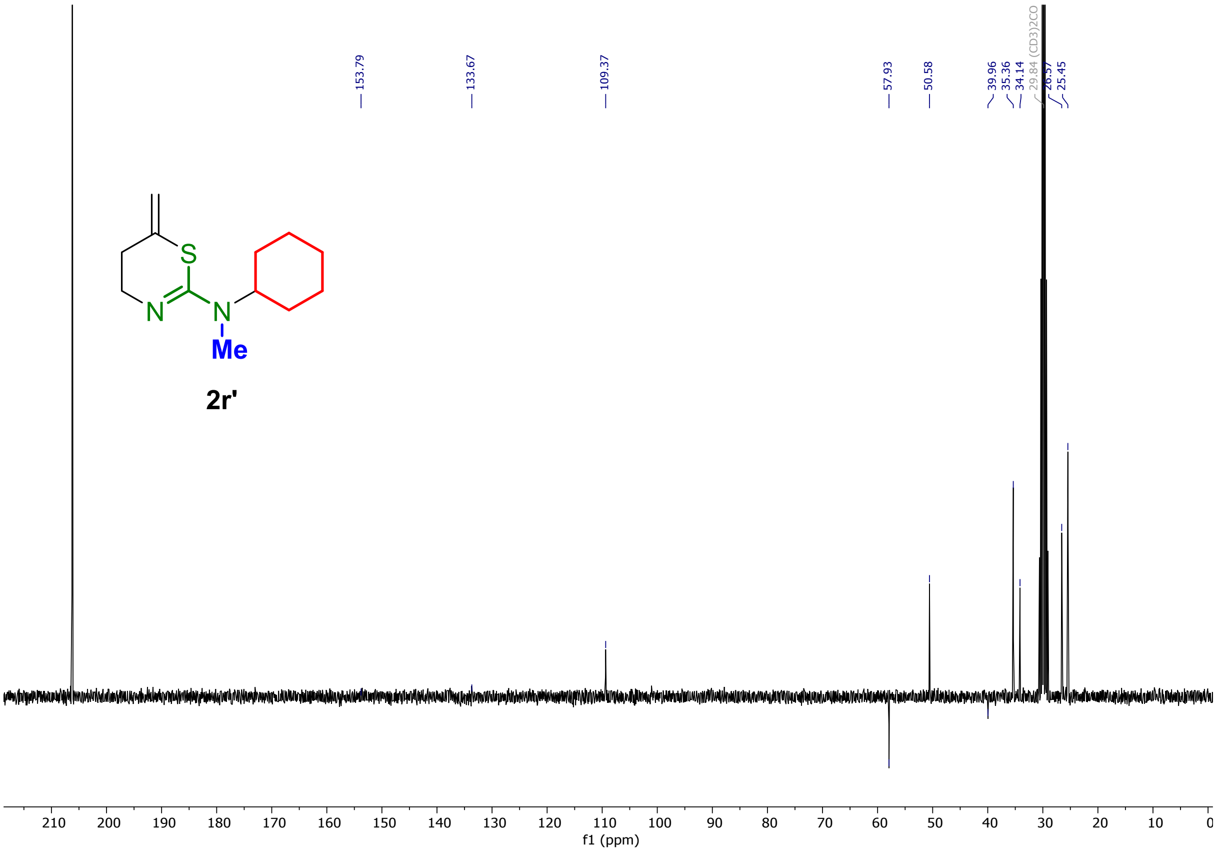

Figure S104. COSY NMR (300 MHz, CD_3COCD_3) spectrum of 1,3-thiazine **2r'**

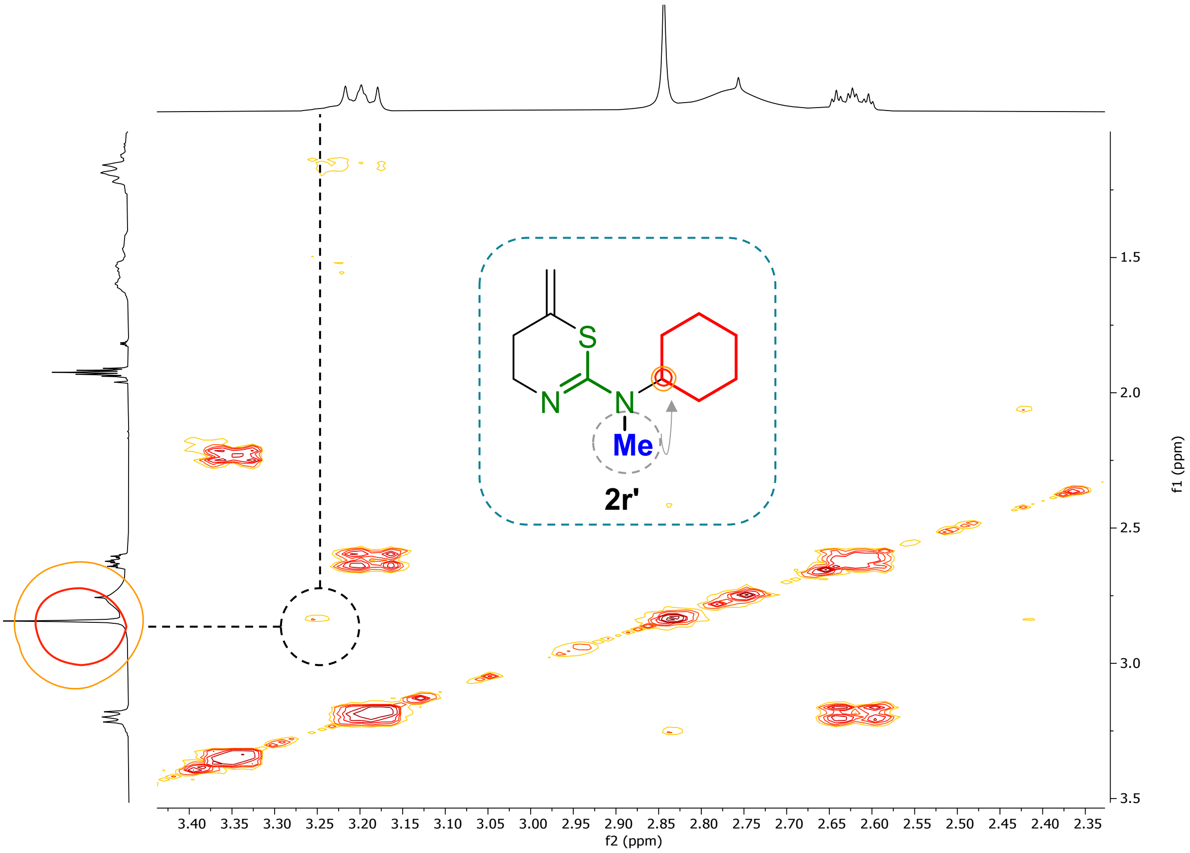

5.3. Spectra of gold(I) catalyst IC

Figure S105. ^{1}H NMR (400 MHz, CD_2Cl_2) spectrum of $[Au(NCMe)(CyJohnPhos)]SbF_6$ (**IC**)

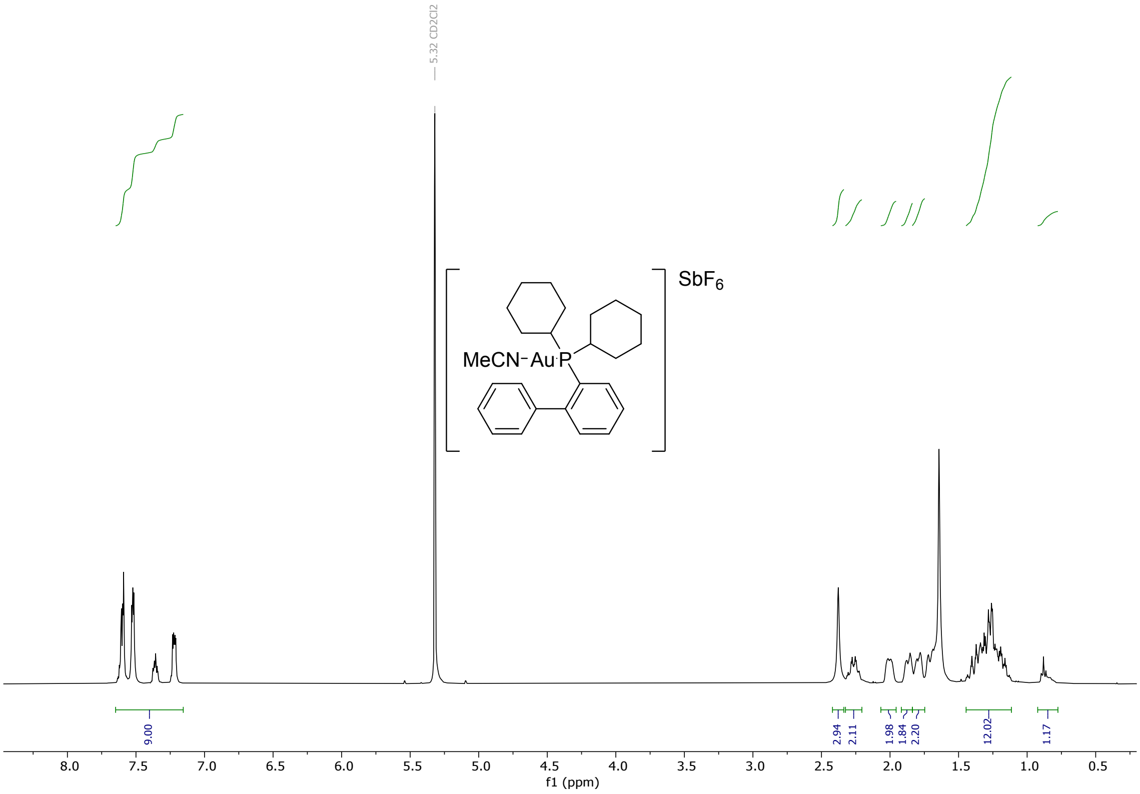

Figure S106. ^{13}C{^{1}H}-APT NMR (101 MHz, CD_2Cl_2) spectrum of **IC**

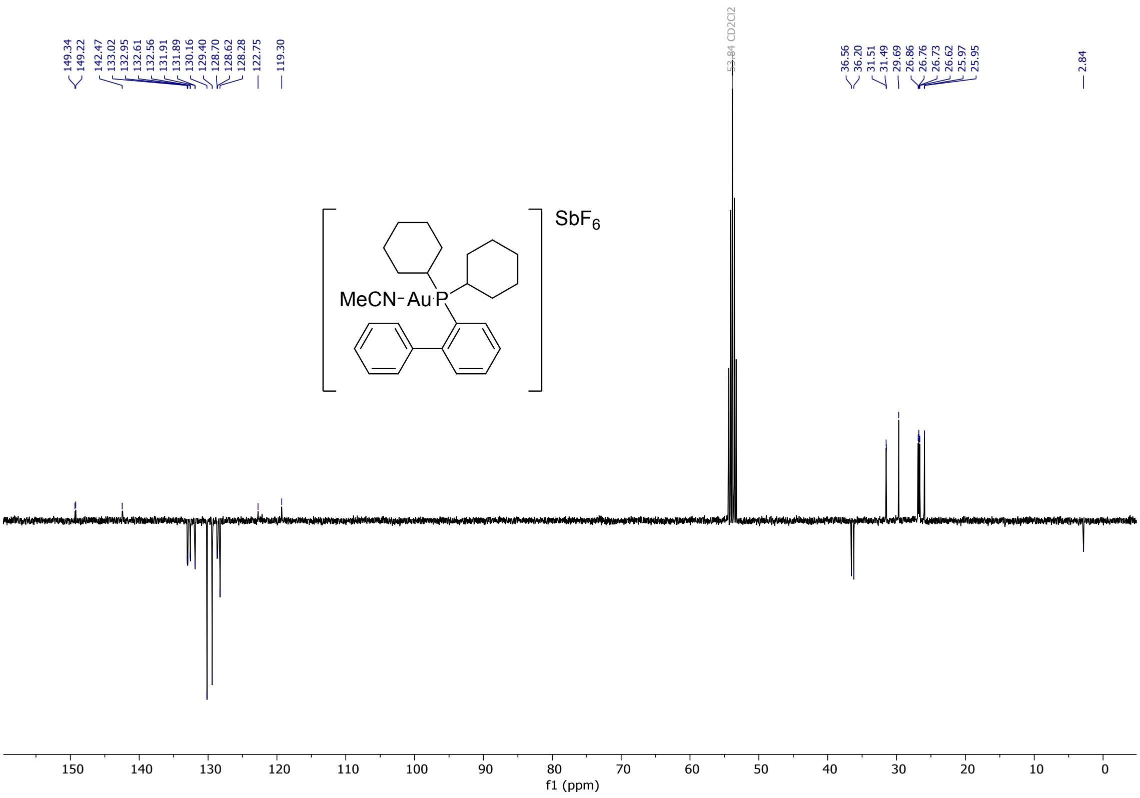

Figure S107. COSY NMR (400 MHz, CD_2Cl_2) spectrum of **IC**

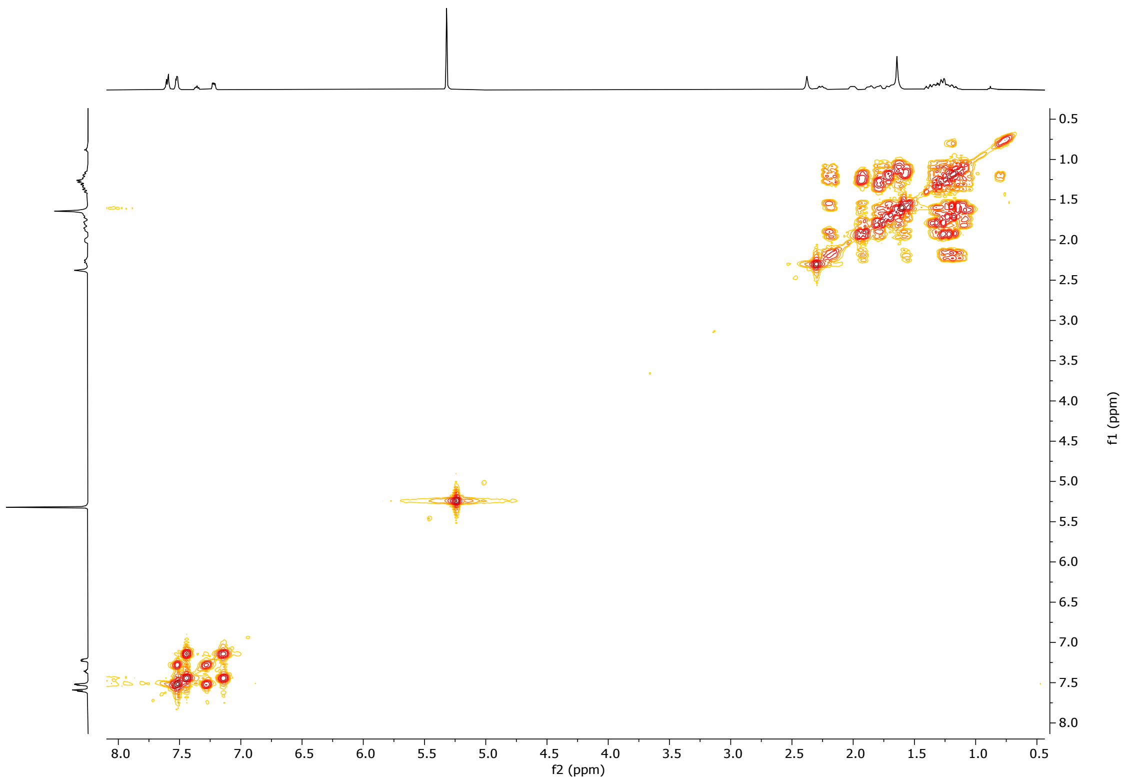

Figure S108. HSQC NMR (400 MHz,101 MHz, CD_2Cl_2) spectrum of **IC**

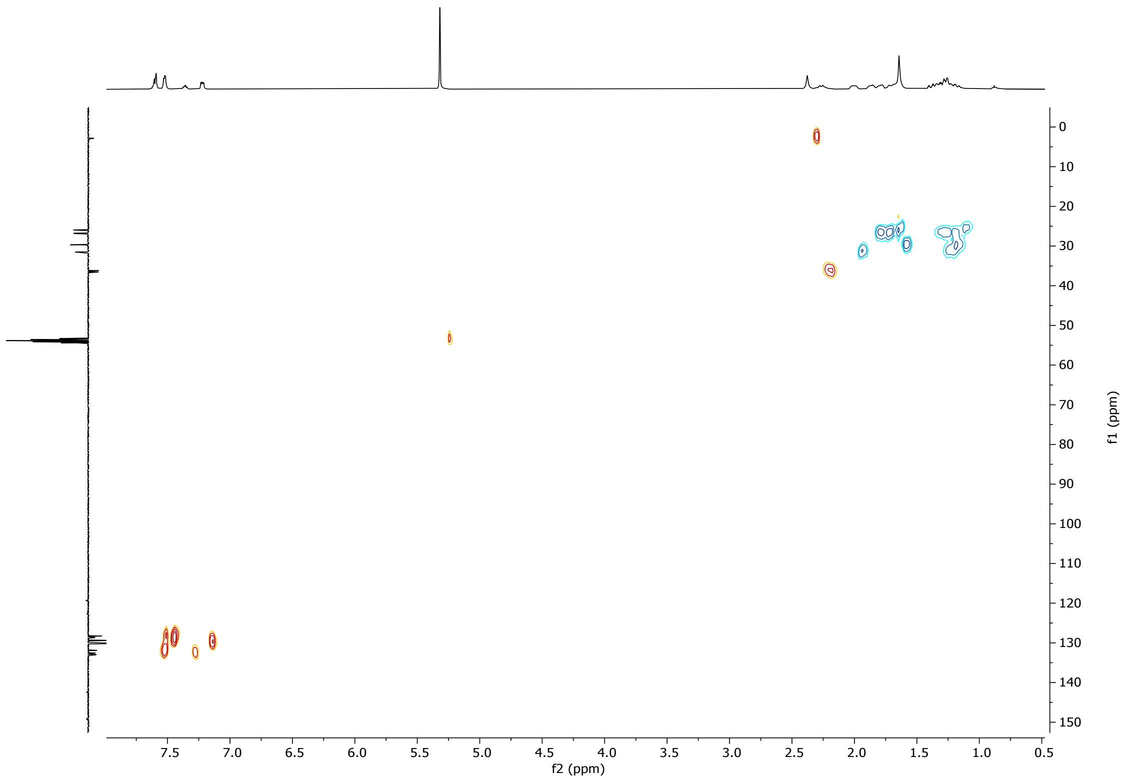

Supplement: Supplementary file 1 — jo2c00947_si_001.pdf [file jo2c00947_si_001.pdf]
